# Copy number variants from 4800 exomes contribute to ~7% of genetic diagnoses in movement disorders, muscle disorders and neuropathies

**DOI:** 10.1038/s41431-023-01312-0

**Published:** 2023-02-13

**Authors:** Maartje Pennings, Rowdy P. P. Meijer, Monique Gerrits, Jannie Janssen, Rolph Pfundt, Nicole de Leeuw, Christian Gilissen, Thatjana Gardeitchik, Meyke Schouten, Nicol Voermans, Bart van de Warrenburg, Erik-Jan Kamsteeg

**Affiliations:** 1 Department of Human Genetics, Radboud university medical center, Nijmegen, the Netherlands; 2 Department of Clinical Genetics, Maastricht University Medical Center, Maastricht, the Netherlands; 3 Department of Neurology, Donders Institute for Brain, Cognition and Behaviour, Radboud university medical Center, Nijmegen, the Netherlands

**Keywords:** Next-generation sequencing, Disease genetics

## Abstract

Various groups of neurological disorders, including movement disorders and neuromuscular diseases, are clinically and genetically heterogeneous. Diagnostic panel-based exome sequencing is a routine test for these disorders. Despite the success rates of exome sequencing, it results in the detection of causative sequence variants in ‘only’ 25–30% of cases. Copy number variants (CNVs), i.e. deletion or duplications, explain 10–20% of individuals with multisystemic phenotypes, such as co-existing intellectual disability, but may also have a role in disorders affecting a single system (organ), like neurological disorders with normal intelligence. In this study, CNVs were extracted from clinical exome sequencing reports of 4800 probands primarily with a movement disorder, myopathy or neuropathy. In 88 (~2%) probands, phenotype-matching CNVs were detected, representing ~7% of genetically confirmed cases. CNVs varied from involvement of over 100 genes to single exons and explained X-linked, autosomal dominant, or - recessive disorders, the latter due to either a homozygous CNV or a compound heterozygous CNV with a sequence variant on the other allele. CNVs were detected affecting genes where deletions or duplications are established as a common mechanism, like *PRKN* (in Parkinson’s disease), *DMD* (in Duchenne muscular dystrophy) and *PMP22* (in neuropathies), but also genes in which no intragenic CNVs have been reported to date. Analysis of CNVs as part of panel-based exome sequencing for genetically heterogeneous neurological diseases provides an additional diagnostic yield of ~2% without extra laboratory costs. Therefore it is recommended to perform CNV analysis for movement disorders, muscle disease, neuropathies, or any other single-system disorder.

## Introduction

Movement disorders (i.e., dystonia, ataxia, spastic paraplegia and Parkinson’s disease), muscle disease (i.e., myopathies, muscular dystrophies and myasthenic syndromes) and neuropathies (i.e., hereditary motor neuropathy, hereditary sensory and autonomic neuropathy, and polyneuropathies) are groups of neurological disorders that affect millions of people worldwide. The prevalence and age at onset of these disorders vary widely, and a monogenetic etiology plays a larger role in some of these (e.g., ataxia or muscular dystrophy) than in others (e.g., Parkinson’s disease). The prevalence of genetic forms of movement disorders, muscle disorders and neuropathies are estimated at up to 5 individuals per 100,000 [1–3].

Currently, diagnostic yields obtained by whole exome sequencing (WES) in individuals with these neurological disorders are approximately 25–30% [4–6]. Although, a part of these individuals will have an acquired cause, this percentage suggests that causative variants are not detected in the majority of these individuals. Copy Number Variants (CNVs) may be responsible for a part of the missing etiology in these disorders, but their contributions are unknown at the moment. CNVs are defined as deletions, insertions, duplications with a size larger than 50 base pairs [7]. While CNVs in different sizes are found as a cause of disease, these are also present in unaffected controls [7]. Therefore, CNVs can be classified from benign to pathogenic, depending on their gene content, breakpoints and occurrence in affected individuals versus controls [7–9]. The cumulative phenotypic effect of more than one Mendelian gene in large CNVs, known as contiguous gene deletion syndromes, are associated with multisystemic phenotypes often with co-existing intellectual disability and/or multiple congenital anomalies. Therefore, genome-wide analysis of CNVs by genomic microarray-based tests or cytogenetic tests such as karyotyping has become a standard procedure for these more multisystemic phenotypes and explains 10–20% of these cases [8, 10].

Single system phenotypes, such as movement disorders, muscle diseases or neuropathies, are generally not routinely subjected to genome-wide CNV analyses. The rationale has been that such phenotypes are likely caused by smaller events (i.e., affecting a single gene) that may not be detected by genome-wide copy number tests. Targeted tests for CNVs, such as multiplex ligation-dependent probe amplification (MLPA), are more commonly performed in these single system phenotypes, but dependent on availability and often based on an educated guess of which gene(s) to test.

The last decade, however, reliable tools have been developed to detect CNVs directly in WES data [11]. The expanded use of clinical exome sequencing, and these possibilities of performing CNV analyses with the same data, boosted the routine detection of (genome-wide) CNVs in individuals with single organ phenotypes.

Here, we retrospectively investigated the detection of CNVs from exome sequencing data in a cohort of 4800 individuals with movement disorders, muscle disease, and neuropathies by analyzing their clinical exome sequencing reports. We show that CNVs contribute to ~7% of the identified genetic causes of these neurological disorders.

## Subjects and methods

### Affected individuals

CNVs were extracted from 4800 clinical exome sequencing reports of the period 2012 to 2020. Only reports concerning individuals with a clinical suspicion of a hereditary movement disorder, muscle disorder or neuropathy were considered. Reports were from the Human Genetics department of the Radboud university medical center in Nijmegen or the Clinical Genetic department of the Maastricht University Medical Center. These hospitals are allocated as partners in European Reference Networks for neuromuscular and rare neurological disorders (Euro-NMD and Euro-RND, respectively). No restrictions on age at onset, clinical symptoms or suspected inheritance pattern were made, except for the cases with Parkinson disease, where only individuals with either an onset below age 45 years or an affected first degree relative were considered for testing. From each family, only one affected individual (proband) was included for exome sequencing, though in some cases, trio-sequencing (with healthy parents) was done.

### Exome sequencing and data analysis

Exome sequencing was performed as previously described [12]. To summarize, capture of exons was done using an Agilent SureSelect Human All Exon 50 Mb Kit (V4 / V5, Santa Clara, CA, USA) and sequencing was performed using an Illumina Hiseq 2000 or 4000 (San Diego, CA, USA). Read mapping and variant calling were done using BWA (mapping) and GATK (calling, version). A filter for one of the following gene panels was applied for analysis: movement disorders (current version 327 genes), muscle disorders (current version 164 genes), neuropathies (current version 149 genes) and/or Parkinson disease (current version 36 genes). The complete lists are available from our website (https://order.radboudumc.nl/genetics/rapid-exoom-sequencing) except for the Parkinson disease panel: Supplementary Table 1). The variants described in this paper were submitted to the LOVD (https://databases.lovd.nl); ID’s are present in the supplementary tables 2–4.

### CNV calling, annotation and interpretation

CNV analysis in WES data was performed by center as previously described [11] using the CoNIFER algorithm (http://conifer.sourceforge.net/) [13]. CNVs with an absolute Z-score greater than 1.7 were considered for analysis. To reduce false calls due to potential batch effects, analyses are performed using the most recent sex-matched samples as controls (*n* = 250). CNVs were annotated based on the number of RefSeq exons affected, the frequency of CNVs within the cohort, and filtered for disease genes from the respective disease panel(s).

The cut-off value for the CoNIFER variant list is restricted to three or more exons. Nevertheless, one or two exon CNVs are present in the raw data and visualized in Integrative Genome Viewer (IGV, Broad Institute, Massachusetts, USA) [14]. In the workflow, the full dataset (all chromosomes view) is analyzed in the IGV browser to detect these small CNVs. In some cases, genes with a high CNV count described in disease, such as *PRKN*, were visually inspected for small CNVs (one or two exons) in more detail. The same is true for recessive genes with one heterozygous SNV call. CNVs affecting only one or two exons, as well as CNVs that were just above the noise level on visual inspection, were validated using MLPA or SNP-based array analysis. MLPA was performed according to the manufacturer’s protocols, using the following kits: P052-D2 (*PRKN* gene), P279-B2 (*CACNA1A* gene), P165-C2 (*SPAST* gene), P099-C3 *(GCH1* gene), P306-B1 (*SPG11* gene), P213-B2 (*SPG7* gene), P1116-B1 (*SGCG* gene) and P033-B3 (*PMP22* gene) kit (MRC-Holland, Amsterdam, the Netherlands). Where applicable, CytoScan HD or CytoScan XON arrays (Thermo Fisher Scientific, Waltham, MA, USA) were used to validate events. Whole genome sequencing for the *TTN* case was performed by Beijing genome institute (China). Intragenic or single gene CNV’s were, similar to single nucleotide variants, classified using ACMG/AMP guidelines for the interpretation of sequence variants [15]. CNV’s affecting multiple genes were classified primarily using the ACMG/ClinGen guidelines for constitutional copy number variants [16].

A multidisciplinary team of (pediatric) neurologists, clinical geneticists and clinical laboratory geneticists was involved in determination of the plausibility of detected variants to be involved in the phenotypes of the affected indiviuals.

## Results

### Individual cohort and CNV analysis

A total of 4800 clinical exome sequencing reports of probands with a suspected genetic primary neurological disorder were searched for both CNVs and single nucleotide variants (SNVs). For these reports, exome data were filtered using one or more gene panels for movement disorders (including hereditary spastic paraplegia, ataxia, dystonia and Parkinson’s disease), muscle disease (e.g., myasthenic syndromes, myopathies and muscular dystrophies) or (poly)neuropathies. The analysis of variants in the coding sequence, including extended splice site variants (+/−8), resulted in the detection of (likely) causative variants in 1289 (~27%) of probands, comparable to previous reports by our group and others [4–6].

CNV analysis in WES data of the same cohort of 4800 affected individuals, using CoNIFER [13] or upon visual inspection, resulted in the detection of 88 disease-associated deletions or duplications (Supplementary Tables 2–4 and supplementary Fig. 2). Only CNVs compatible with the individual’s phenotypes were reported. Accordingly, ~2% of individuals in this cohort had a (likely) pathogenic CNV. The highest percentage of CNVs from the total of variants was detected in the individuals with neuropathies (3.8%), followed by movement disorders (2%), and muscle disease (1.2%) (Supplementary Fig. 1). In the entire cohort, CNVs constitute ~7% (88/ (1289 + 88)) of the obtained genetic diagnoses.

### CNVs detected in individuals with movement disorders

**Fig. 1:**
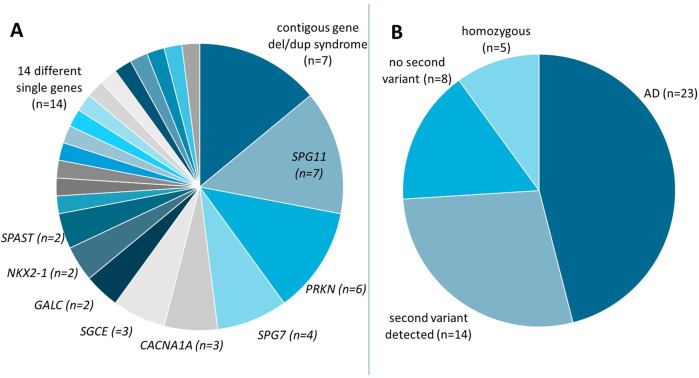
Copy number variants from probands with movement disorders. **A** 50 CNVs affect either multiple genes (*contiguous gene del/dup syndrome*) or single genes (indicated at the pie slices). Within the ‘14 different single genes’ are also two events in two probands each, affecting the adjacent COL4A1/COL4A2 and CACNB4/SCN1A/SCN2A genes, respectively. **B** CNVs were either detected in the heterozygous state in dominant disorders (*AD*) or in recessive disorders with or without second variants (indicated), or in the in the homozygous (indicated) state. The numbers of affected probands (*n*) are indicated.

**Fig. 2:**
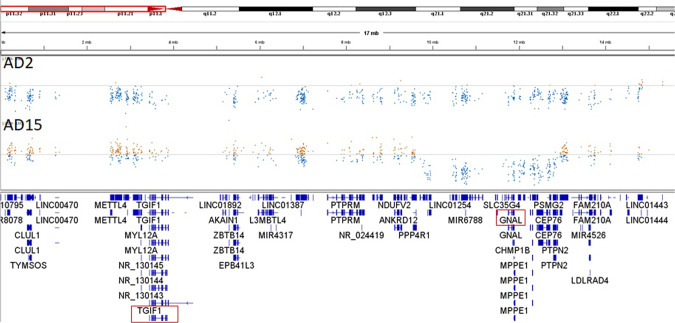
CNVs affecting the GNAL gene. Integrative genome viewer screenshot showing the normalized number of exome sequence reads of the p-arm of chromosome 18 from two probands (*AD2, AD15*). Data points below and above the normalized median are in blue and brown, respectively. The genes located in the p-arm of chromosome 18 are indicated in the lower panel at their respective positions. Genes involved in autosomal dominant Mendelian disorders are boxed in red.

The majority of 2,527 probands with a movement disorder had hereditary spastic paraplegia (42%), cerebellar ataxia (30%) or dystonia (16%), and a minority had early-onset or familial Parkinson’s disease (2.5%), or a combination of these movement disorders (9.5%). In the movement disorders cohort, 50 CNVs were detected (Fig. 1A, Supplementary Table 2 and Supplementary Fig. 2). Seven of the 50 CNVs were associated with established pathogenic contiguous gene deletions or duplication syndromes, including a recurrent deletion or duplication in 22q11.2, 16p11.2 or 15q11.2q13.1 (Prader-Willi / Angelman syndrome region). Two illustrative examples (AD2 and AD15, Supplementary Table 2) both contain the *GNAL* gene (Fig. 2), involved in dystonia type 25. Both individuals manifested dystonia. individual AD15 had dystonia and dysarthria, and a deletion of ~3 Mb (no known contiguous gene deletion syndrome, but within the chromosome 18p deletion syndrome region). AD2 has a ~14 Mb terminal deletion in the short arm of chromosome 18 and additional features of facial dysmorphisms and psychomotor delay compatible with the 18p deletion syndrome [17]. This, suggests that at least one other gene in the extended region of 9 Mb is responsible for the additional features of the 18p deletion syndrome.

**Fig. 3:**
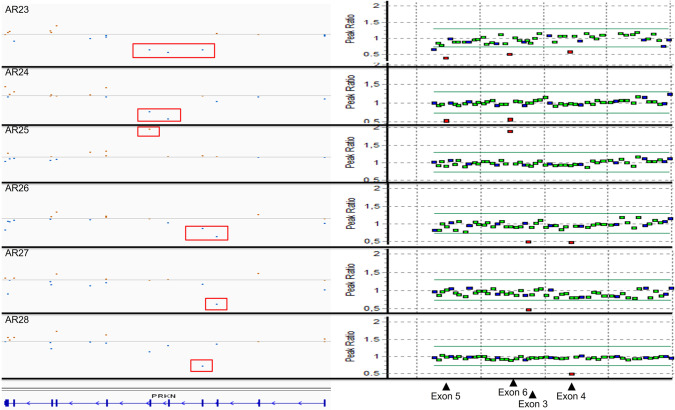
Small CNVs of the *PRKN* gene. Integrative genome viewer screenshot (left panel) showing the normalized number of exome sequence reads of the 11 exons of the *PRKN* gene (indicated in blue bars, bottom) of six individuals with Parkinson disease (*AR23-AR28*). Data points below and above the normalized median are in blue and brown, respectively. CNVs affecting one or multiple exons of the *PRKN* gene are indicated by red boxes. Fidelity of the CNVs was confirmed by Multiplex ligation-dependent probe amplification (MLPA, right panel). Data points within and beyond the thresholds of normal (diploid) are in green/blue and red, respectively. The MLPA probes corresponding to the exons 3–6 are indicated by arrowheads.

The remaining 43 CNVs detected in individuals with movement disorders affected single or multiple genes (up to 50). CNVs of exons of the *SPG11* gene were most common (*n* = 7), due to a founder deletion of the exons 31–34 [18–20]. In three individuals, this founder allele was detected without a second variant to explain the recessive inheritance despite thorough examination of MLPA and WES data. CNVs within the *PRKN* gene, either homozygous or compound heterozygous with sequence variants on the other allele, were a common cause of early-onset/familial Parkinson’s disease (n = 5) [21]. In contrast to the large contiguous gene deletions, *PRKN* variants in these individuals involved only one or a few exons of the gene (Fig. 3). These variants were mostly detected upon visual inspection of the CoNIFER bed file data, and confirmed by MLPA. Multiple CNVs affecting *SPG7* (*n* = 4), *CACNA1A* (*n* = 3), *SGCE* (*n* = 3), *NKX2-1* (*n* = 2) and *SPAST* (*n* = 2) were detected in individuals with episodic ataxia, dystonia, or spastic paraplegia, respectively (Supplementary Table 2). Two heterozygous, partial deletions of the *GALC* gene were detected, both detected in combination with a missense variant, in two unrelated individuals with variable phenotypes. Due to the clinical variability of Krabbe disease [22], a confirmation with a metabolic test is needed.

The remaining 14 CNVs were detected in a single proband each and affected the *B4GALNT1, COL4A1/COL4A2, CACNB4/SCN1A/SCN2A, CACNA1G, DDHD2, FARS2, GCH1, GNAL, ITPR1, KCTD7, PANK2, PDHX, SYNE1 or TANGO2* genes.

The 50 CNVs detected in the movement panel are classified as pathogenic (*n* = 42), or likely pathogenic (LP) (*n* = 6), or as variant of uncertain significance (VOUS) (*n* = 2) (Supplementary table 2).

Twenty-three of these CNVs were associated with autosomal dominant inheritance and 27 with autosomal recessive inheritance (Fig. 1B). CNVs in the 27 autosomal recessive genes were detected in the homozygous state in five probands or heterozygously present in combination with another variant in 14 probands, although we did not always have the opportunity to test whether the variants were in trans orientation. Eight affected individuals had only one heterozygous CNV, and no second variant detected, in a recessive gene. The presence of a second variant in each of these eight individuals is not excluded. Possibly, variants may not have been detected by WES (i.e., intron or promoter variants). Alternatively, these individuals may have symptoms of another origin (genetic or other).

Seven of the 50 CNVs are gains (Supplementary Table 2), of which the pathogenicity is generally more difficult to determine as it is more difficult to predict their effect on gene function, depending on the underlying mechanism (duplication, insertion, direct or inverted). However, the recurrent duplication in 22q11.2 (individual AD1) and the 15q11.2q13 region (individual AD5) are well-known microduplication syndromes involved in variable and incomplete penetrant neurological disorders [23, 24]. For the 15q11.2q13 region, cytogenetic follow up is needed to establish whether it is a duplication or a supernumerary marker chromosome [25]. Additionally, triplosensitivity (an extra copy) of *COL4A1/2* (AD8) and *SCN1A/2A* (AD9) and duplication of *PRKN* exons (AR24) are considered (likely) pathogenic as these were described in multiple affected individuals by others [26–28].

In total, 48 of the 50 variants in the movement disorders cohort were (likely) pathogenic and explain the phenotype in 38 individuals. eight variants (8/48), though (likely) pathogenic, did not directly result in a molecular diagnosis because a second variant in a recessive gene could not be identified (AR6–8, 13–16 and 27). Two individuals (2/48) had a pathogenic CNV with a possibly matching phenotype (two 16p11.2 deletions known to cause neurological phenotypes with marked reduced penetrance). Finally, two variants (2/50) were considered variants of uncertain significance (AD13, AR9).

### CNVs detected in individuals with a muscle disease

**Fig. 4:**
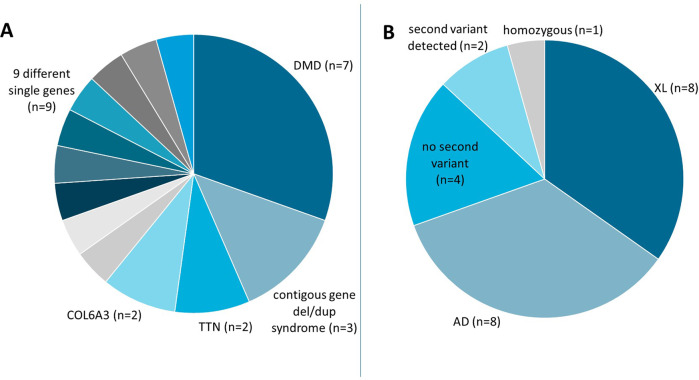
Copy number variants from probands with muscle disease. **A** 23 CNVs affect either multiple genes (*contiguous gene del/dup syndrome*) or single genes (indicated at the pie slices). Within the ‘9 different single genes’ is also one event affecting multiple genes (including *SLC16A3*, *CHAT*, *OGDHL*) in a single proband. **B** CNVs were either detected in X-linked disorders (*XL*), in the heterozygous state in dominant disorders (*AD*), in recessive disorders with or without second variants (indicated), or in the homozygous (indicated) state. The numbers of affected probands (*n*) are indicated.

A total of 1880 probands in the cohort of muscle disease individuals had (limb-girdle) muscular dystrophies (26%), myopathies (24%), exercise intolerance (19%) or hyperCKemia and/or rhabdomyolysis (12%), or other muscular problems (19%). In this group of 1880 individuals, 23 CNVs (1.2%) were detected (Fig. 4A, Supplementary table 3 and Supplementary Fig. 2).

Three of the 23 individuals had a recurrent contiguous gene deletion or duplication syndrome: 22q11.2 (BD1 and BD2) and 16p13.11 (BD3) involved in variable and incomplete penetrant neurological disorders [23, 29]. Three other CNVs affected multiple genes (probands BD4, BD8, and BX1). Each of these three CNVs contained one likely causative disease gene: *SMCHD1*, *PMP22*, or *MTM1*, involved in facioscapulohumeral muscular dystrophy-2 (digenic inheritence with the D4Z4 permissive haplotype), Charcot-Marie-Tooth disease 1A, and X-linked myotubular myopathy, respectively. In proband BR1, the heterozygous deletion did not contain dominant disease genes, but three recessive genes (*SLC38A1*, *CHAT* and *OGDHL*) with a muscle phenotype: congenital myasthenic syndrome type 21 or type 6, or Yoon-Bellen neurodevelopmental syndrome, respectively. It is uncertain whether any of these three genes is the cause of the phenotype of BR1.

**Fig. 5:**
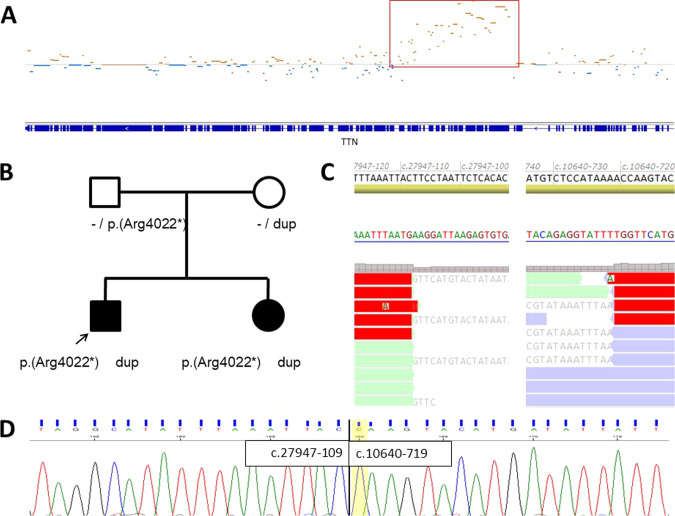
Tandem duplication of a part of the *TTN* gene in recessive myopathy. **A** Integrative genome viewer screenshot showing the normalized number of exome sequence reads of the *TTN* gene (indicated in blue bars, bottom). Data points below and above the normalized median are in blue and brown, respectively. The duplication of a large part of the *TTN* gene is indicated by a red box. **B** Pedigree of the family. Circles and squares indicate females and males, respectively. Solid symbols indicate affected individuals. Alleles without (-) variant, the duplication (dup) and a nonsense variant (p.Arg4022*) are indicated. **C** Part of the intron 122 and intron 50 reference sequences and their positions by coding DNA reference (top), their complement sequence (arrow head) and BAM files from whole genome sequencing data showing reads spanning the breakpoint of the tandem duplication in complement orientation. **D** Part of the intron 122 and intron 50 reference sequences (top) and Sanger sequencing electropherograms showing the breakpoint positions of the tandem duplication in forward orientation (NM_1333784.4).

The most CNVs affecting a single gene in the muscle disease cohort were detected in the *DMD* gene (*n* = 7, including manifesting females), related to X-linked Duchenne or Becker muscular dystrophy. This is not surprising given the fact that this is one of the largest genes in the human genome and pathogenic CNVs are common in *DMD* [30]. Two CNVs were detected in the *TTN* gene (that codes for the largest protein in the human body) in which a second variant was demonstrated in both individuals (BR6 and BR7). BR6 had a pathogenic deletion of 35 exons and a missense variant of uncertain significance, while BR7 and his sibling were compound heterozygous for a stop-gain variant and a duplication of the exons 51–122 (Fig. 5A, B, Supplementary Table 3)). Whole genome sequencing was performed showing that this variant was a tandem duplication within the *TTN* gene (Fig. 5C), which was confirmed by Sanger sequencing of the breakpoint (Fig. 5D). A tandem duplication of this size, though predicted to result in an in-frame transcript, is very likely to disturb the gene’s function and is thus considered to be likely pathogenic.

Two other intragenic CNVs were found in the *COL6A3* gene, one deletion and one duplication (BD6 and BD7). Parental testing showed that the deletion in individual BD6 occurred *de novo*. Since this is also predicted to be an in-frame deletion of the Von Willebrand factor domain of the protein, it is compatible with a dominant negative effect and thus considered pathogenic. No further follow-up analysis was possible for the CNV of a part of the *COL6A3* gene (BD7), so this variant remains classified as variant of uncertain significance despite the likely match with Ullrich / Bethlem myopathy. In another six probands (BD5, BD8, BR2, BR3, BR4, BR5) CNVs affected the *CAPN3, PMP22, GAA, LARGE1, NEB*, or *SGCG* gene, respectively.

Of the 23 CNVs detected in the muscle disease cohort, the vast majority (*n* = 17) were classified as pathogenic, followed by three likely pathogenic (LP) variants and 3 VOUS (Supplementary table 3).

In the muscle disease panel, many CNVs were associated with X-linked inheritance mainly due to variants in the *DMD* gene (*n* = 7/23). Eight (8/23) were involved in autosomal dominant inheritance and seven (7/23) with autosomal recessive inheritance (Fig. 4B). Of the seven CNVs associated with autosomal recessive inheritance, the deletion in the *SGCG* gene was detected homozygously and two other affected individuals with a CNV in *TTN* showed a second variant in the same gene. In four individuals, no second variant could be detected in recessive genes (Supplementary Table 3). Second variants may be present but remained undetected by WES (i.e. intron or promoter variants), or the individuals may be affected due to other (genetic) reasons.

Twenty of the 23 CNVs in the muscle disease cohort were (likely) pathogenic and explain the phenotype in 16 probands. The remaining four (likely) pathogenic variants (4/20) did not directly result in a molecular diagnosis because a second variant in a recessive gene was not detected, or is a variant of uncertain significance. Finally, three CNVs were of uncertain significance, even though they were found in individuals with matching phenotypes, because they constituted duplications (*COL6A3* and *LARGE1*) and one was an intragenic in-frame deletion of a gene with both dominant and recessive inheritance patterns (*CAPN3*).

### CNVs detected in neuropathies

From a total of 393 probands, the majority having a (poly)neuropathy, 15 (4%) had a CNV (Supplementary Table 4 and Supplementary Fig. 2).

**Fig. 6:**
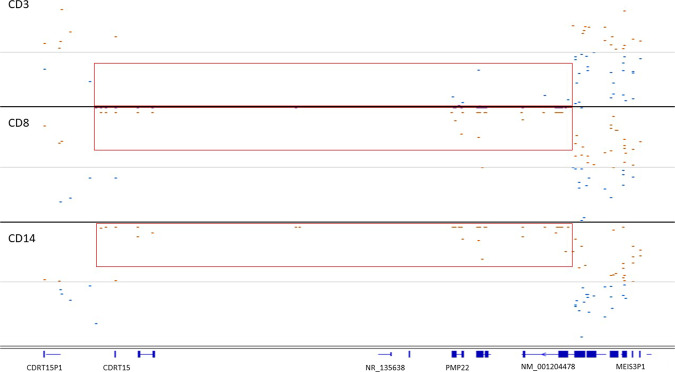
CNVs affecting the *PMP22* gene. Integrative genome viewer screenshot showing the normalized number of exome sequence reads of the *PMP22* locus (gene exons indicated in blue bars) gene. Data points below and above the normalized median are in blue and brown, respectively. The CNVs are indicated by a red box.

Pathogenic duplications and deletions of the *PMP22 gene* (*n* = 13) due to a recurrent duplication or deletion in 17p12, respectively, were most often detected (Supplementary table 4; Fig. 6 showing a representative duplication and a representative deletion). Duplications of *PMP22* are associated with Charcot-Marie-Tooth disease type 1A, whereas deletions cause distal hereditary motor neuronopathy type VIIB. Nonhomologous recombination between low copy repeats are a well-known mechanism causing *PMP22* CNVs [11, 31] and explains the similarity of the break points in the individuals DNA. Two other CNVs affected the genes *DCTN1* or *FARS2*. *DCTN1* variants described to date cause disease by diminishing microtubule binding and lead to intracytoplasmic inclusions [32]. Since haploinsufficiency due to a deletion would not have that effect, the CNV of *DCTN1* is regarded a variant of uncertain significance. *FARS2* variants cause autosomal recessive spastic paraplegia. As no second (possible) pathogenic *FARS2* variant was detected in this individual (CR1), the molecular diagnosis was not established, although we cannot rule out the presence of a second variant not detected by WES (Supplementary Table 4).

## Discussion

We detected CNVs in 88 affected individuals (1.8%) from our clinical cohort of 4800 probands with a likely genetic diseases in which a movement disorder, muscle disease or neuropathy were the dominant phenotypes. Previously, we detected 1289 single nucleotide variants (including variants of uncertain significance in matching genes; 26.9%) in this identical cohort. Thus, CNVs contribute to ~7% of the identified genetic diagnoses in this cohort. The fact that we detected multiple variants in genes where deletions or duplications are established as a common mechanism, like *PRKN, DMD* and *PMP22* [21, 30, 31] suggests that copy number detection from WES is a reliable tool.

These data reveal that CNV detection from WES is worthwhile for movement disorders, muscle disease, neuropathies, or any other single system disorder, like vision disorders or renal disease. Additionally, we have shown the additive value of CNV analysis from WES by detecting deletions in genes that have not or rarely been reported to contain CNVs (like *B4GALNT1* or *SYNE1)*. Moreover, these findings indicate that the analysis of copy numbers from WES is beneficial for affected individuals since it will increase the yield of variant detection without extra laboratory costs and will result in the detection of CNVs that otherwise would have remained obscure.

### Surprisingly many ‘large’ CNVs are identified in the neurological cohort

Since the cohort predominantly consists of individuals with an single system neurological disorder, we expected to mainly find CNVs affecting single genes. Nevertheless, we observed 41 gene deletions or duplications affecting more than one gene. From these, ten caused contiguous gene deletion/ duplication syndromes (i.e. syndromes caused by cumulative effects of more than one gene). The detection of contiguous gene deletion/duplication syndromes in individuals within the movement or muscle disorder cohort is partly explained by an initial onset of hypotonia in neonates or young children that have not presented the full phenotype yet. The initial symptoms prompted a request for one of the neurological disorders gene panels, but resulted in the detection of large CNVs, such as those causing the 22q11.2 duplication syndrome or Prader-Willi syndrome. These individuals have developed or will likely develop a multisystem phenotype over time [33, 34].

Other large deletions, however, do lead to more pure clinical phenotypes. For example, individual AD15 has a large contiguous gene deletion containing 28 genes, but only manifested dystonia and dysarthria due to haploinsufficiency of the dystonia-related *GNAL* gene located in this deletion (Fig. 2, Supplementary Table 2). The other 27 genes are thus less likely to cause a disorder due to haploinsufficiency. In fact, none of these 27 genes are listed as an OMIM dominant disease gene. Similar observations are made for three other contiguous gene deletions: the deletion of >20 genes, including *NKX2-1* in a proband with balance problems, myoclonus, chorea and dystonia; a deletion of *SGCE* among 15 other genes in a proband with myoclonic dystonia; and a deletion affecting seven genes including *SMCHD1* in a individual with muscular dystrophy only. Considering the fact that large CNVs are also detected as normal variation [7], it seems indeed a matter of gene content and the underlying mechanism of disease (i.e. haploinsufficiency vs. dominant negative or recessive) whether a CNV has no effect, causes an single system disorder, or a contiguous gene deletion / duplication syndrome due to the cumulative effect of multiple gene defects.

Another remarkable finding is the duplication of tandem copy genes in two cases. The first is the duplication of both the *COL4A1* and (partial) *COL4A2* genes in a affected individual (AD8) with multiple cerebral infarctions leading to neurological problems including movement disorders and speech problems. Haploinsufficiency of each gene, *COL4A1* or *COL4A2*, is sufficient to cause brain small vessel disease (OMIM 175780 and 614483). It is uncertain what mechanistic effect this specific duplication has, albeit simultaneous duplication of the complete *COL4A1* and *COL4A2* genes has been described in brain small vessel disease [26]. Similarly, individual AD9 has dystonia and seizures and a duplication encompassing three genes, *CACNB4*, *SCN1A* and *SCN2A*, each responsible for epilepsy syndromes with or without movement disorders. It is unclear whether the simultaneous duplication of these three genes causing similar or overlapping phenotypes will result in a more severe phenotype or earlier onset.

### CNVs in recessive disorders

CNVs in 35 probands affected recessive disease genes. Six of those were homozygous, while 16 probands had a CNV in combination with a single nucleotide variant compatible with recessive inheritance (Supplementary Tables 2–4). The remaining 13 had, however, no second (pathogenic) variant to complete a definite genetic diagnosis, despite extensive analysis using targeted resequencing, array CGH and analysis of rare coding variants. Possibly, some of those may have second variants that have remained undetected because they reside in introns or promotors and other regulating sequences [35], or represent more exotic variants like exon inversions or insertion of mobile elements [36]. Emerging techniques such as (long-read) whole genome sequencing or transcriptome sequencing may reveal such variants in the future [35, 37].

Alternatively, the heterozygous variants in recessive genes may not be related to the disorder in these individuals and, yet undetermined, causative variants in other genes or even nongenetic causes may exist.

### ‘Missing’ gene defects

The incorporation of CNV detection in our cohort has increased the diagnostic yield by 1.8% accumulating with single nucleotide variants to 30.5%. Given the fact that a genetic cause was suspected in all the individuals in this cohort, we are still missing the majority of causative variants. Despite the extra yield described here, smaller CNVs, especially those smaller than three exons, may be missed by CoNIFER. CNVs may also be missed in regions not covered either due to poor sequencing or mapping/calling difficulties, as is the case for the triplicated regions in the *NEB* and *TTN* genes. New callers may be helpful to better detect CNVs in the future. Other types of variants may also be missed, like variants affecting proper translation (like copy neutral structural variants or mobile element insertions). However, large scale genome sequencing efforts have not shown significant increases in diagnostic yields [38]. Other concepts, like multigenic inheritance [39], somatic variants that are not present in blood cells [40], or dominant inheritance in genes previously established as recessive, will be the future challenge to increase diagnostic yields.

In conclusion, we here show that CNV analysis from exome data is important to detect a part of the missing gene defects in neurogenetic disorders and a valuable tool for virtually any gene panel approach based on exome sequencing. Nevertheless, diagnostic labs will also have to strive to increase diagnostic yields by innovative approaches to find the genetic causes in the remaining group of unsolved cases.

## Supplementary information

Sup. Figure 1

Sup. Figure 2

Sup. Table 1

Sup. Table 2

Sup. Table 3

Sup. Table 4

Description of Supplementary information

## Data Availability

The datasets generated during and/or analysed during the current study are not publicly available due to privacy regulations but (partial) datasets are available from the corresponding author on reasonable request.

## References

[1] Hong JM, Choi YC, Shin S, Lee JH, Shin HY, Kim SM (2019). Prevalence and socioeconomic status of patients with genetic myopathy in Korea: A nationwide, population-based study. Neuroepidemiology.

[2] Eggermann K, Gess B, Hausler M, Weis J, Hahn A, Kurth I (2018). Hereditary neuropathies. Dtsch Arztebl Int.

[3] Matilla-Duenas A, Corral-Juan M, Rodriguez-Palmero Seuma A, Vilas D, Ispierto L, Morais S (2017). Rare neurodegenerative diseases: Clinical and genetic update. Adv Exp Med Biol.

[4] van de Warrenburg BP, Schouten MI, de Bot ST, Vermeer S, Meijer R, Pennings M (2016). Clinical exome sequencing for cerebellar ataxia and spastic paraplegia uncovers novel gene-disease associations and unanticipated rare disorders. Eur J Hum Genet.

[5] Westra D, Schouten MI, Stunnenberg BC, Kusters B, Saris CGJ, Erasmus CE (2019). Panel-based exome sequencing for neuromuscular disorders as a diagnostic service. J Neuromuscul Dis.

[6] Walsh M, Bell KM, Chong B, Creed E, Brett GR, Pope K (2017). Diagnostic and cost utility of whole exome sequencing in peripheral neuropathy. Ann Clin Transl Neurol.

[7] Zarrei M, MacDonald JR, Merico D, Scherer SW (2015). A copy number variation map of the human genome. Nat Rev Genet.

[8] Scionti F, Di Martino MT, Pensabene L, Bruni V, Concolino D (2018). The cytoscan HD array in the diagnosis of neurodevelopmental disorders. High Throughput.

[9] South ST, Lee C, Lamb AN, Higgins AW, Kearney HM (2013). Working Group for the American College of Medical G, et al. ACMG Standards and Guidelines for constitutional cytogenomic microarray analysis, including postnatal and prenatal applications: Revision 2013. Genet Med.

[10] Vulto-van Silfhout AT, Hehir-Kwa JY, van Bon BW, Schuurs-Hoeijmakers JH, Meader S, Hellebrekers CJ (2013). Clinical significance of de novo and inherited copy-number variation. Hum Mutat.

[11] Pfundt R, Del Rosario M, Vissers L, Kwint MP, Janssen IM, de Leeuw N (2017). Detection of clinically relevant copy-number variants by exome sequencing in a large cohort of genetic disorders. Genet Med.

[12] Neveling K, Feenstra I, Gilissen C, Hoefsloot LH, Kamsteeg EJ, Mensenkamp AR (2013). A post-hoc comparison of the utility of sanger sequencing and exome sequencing for the diagnosis of heterogeneous diseases. Hum Mutat.

[13] Krumm N, Sudmant PH, Ko A, O’Roak BJ, Malig M, Coe BP (2012). Copy number variation detection and genotyping from exome sequence data. Genome Res.

[14] Robinson JT, Thorvaldsdottir H, Winckler W, Guttman M, Lander ES, Getz G (2011). Integrative genomics viewer. Nat Biotechnol.

[15] Richards S, Aziz N, Bale S, Bick D, Das S, Gastier-Foster J (2015). Standards and guidelines for the interpretation of sequence variants: a joint consensus recommendation of the American College of Medical Genetics and Genomics and the Association for Molecular Pathology. Genet Med.

[16] Riggs ER, Andersen EF, Cherry AM, Kantarci S, Kearney H, Patel A (2020). Technical standards for the interpretation and reporting of constitutional copy-number variants: a joint consensus recommendation of the American College of Medical Genetics and Genomics (ACMG) and the Clinical Genome Resource (ClinGen). Genet Med.

[17] Wester U, Bondeson ML, Edeby C, Anneren G (2006). Clinical and molecular characterization of individuals with 18p deletion: a genotype-phenotype correlation. Am J Med Genet A.

[18] de Bot ST, Burggraaff RC, Herkert JC, Schelhaas HJ, Post B, Diekstra A (2013). Rapidly deteriorating course in Dutch hereditary spastic paraplegia type 11 patients. Eur J Hum Genet.

[19] Conceicao Pereira M, Loureiro JL, Pinto-Basto J, Brandao E, Margarida Lopes A, Neves G (2012). Alu elements mediate large SPG11 gene rearrangements: further spatacsin mutations. Genet Med.

[20] Bauer P, Winner B, Schule R, Bauer C, Hafele V, Hehr U (2009). Identification of a heterozygous genomic deletion in the spatacsin gene in SPG11 patients using high-resolution comparative genomic hybridization. Neurogenetics.

[21] Elfferich P, Verleun-Mooijman MC, Maat-Kievit JA, van de Warrenburg BP, Abdo WF, Eshuis SA (2011). Breakpoint mapping of 13 large parkin deletions/duplications reveals an exon 4 deletion and an exon 7 duplication as founder mutations. Neurogenetics.

[22] De Gasperi R, Gama Sosa MA, Sartorato EL, Battistini S, MacFarlane H, Gusella JF (1996). Molecular heterogeneity of late-onset forms of globoid-cell leukodystrophy. Am J Hum Genet.

[23] Alberti A, Romano C, Falco M, Cali F, Schinocca P, Galesi O (2007). 1.5 Mb de novo 22q11.21 microduplication in a patient with cognitive deficits and dysmorphic facial features. Clin Genet.

[24] Bundey S, Hardy C, Vickers S, Kilpatrick MW, Corbett JA (1994). Duplication of the 15q11-13 region in a patient with autism, epilepsy and ataxia. Dev Med Child Neurol.

[25] Kleefstra T, de Leeuw N, Wolf R, Nillesen WM, Schobers G, Mieloo H (2010). Phenotypic spectrum of 20 novel patients with molecularly defined supernumerary marker chromosomes 15 and a review of the literature. Am J Med Genet A.

[26] Saskin A, Sillon G, Palfreeman N, Buhas D (2018). COL4A1/2 CNVs and cerebral small vessel disease: Narrowing in on the critical chromosomal region. Neurology.

[27] Goeggel Simonetti B, Rieubland C, Courage C, Strozzi S, Tschumi S, Gallati S (2012). Duplication of the sodium channel gene cluster on 2q24 in children with early onset epilepsy. Epilepsia.

[28] Schule B, Hatchwell E, Eis PS, Langston JW (2015). Comparative genomic hybridization solves a 14-year-old PARKIN mystery. Ann Neurol.

[29] Allach El Khattabi L, Heide S, Caberg JH, Andrieux J, Doco Fenzy M, Vincent-Delorme C (2020). 16p13.11 microduplication in 45 new patients: refined clinical significance and genotype-phenotype correlations. J Med Genet.

[30] Selvatici R, Rossi R, Fortunato F, Trabanelli C, Sifi Y, Margutti A (2021). Ethnicity-related DMD Genotype Landscapes in European and Non-European Countries. Neurol Genet.

[31] Inoue K, Dewar K, Katsanis N, Reiter LT, Lander ES, Devon KL (2001). The 1.4-Mb CMT1A duplication/HNPP deletion genomic region reveals unique genome architectural features and provides insights into the recent evolution of new genes. Genome Res.

[32] Farrer MJ, Hulihan MM, Kachergus JM, Dachsel JC, Stoessl AJ, Grantier LL (2009). DCTN1 mutations in Perry syndrome. Nat Genet.

[33] Ensenauer RE, Adeyinka A, Flynn HC, Michels VV, Lindor NM, Dawson DB (2003). Microduplication 22q11.2, an emerging syndrome: clinical, cytogenetic, and molecular analysis of thirteen patients. Am J Hum Genet.

[34] Butler MG, Meaney FJ, Palmer CG (1986). Clinical and cytogenetic survey of 39 individuals with Prader-Labhart-Willi syndrome. Am J Med Genet.

[35] Lionel AC, Costain G, Monfared N, Walker S, Reuter MS, Hosseini SM (2018). Improved diagnostic yield compared with targeted gene sequencing panels suggests a role for whole-genome sequencing as a first-tier genetic test. Genet Med.

[36] Burns KH (2020). Our Conflict with Transposable Elements and Its Implications for Human Disease. Annu Rev Pathol.

[37] Pauper M, Kucuk E, Wenger AM, Chakraborty S, Baybayan P, Kwint M (2021). Correction: Long-read trio sequencing of individuals with unsolved intellectual disability. Eur J Hum Genet.

[38] Lee HF, Chi CS, Tsai CR (2021). Diagnostic yield and treatment impact of whole-genome sequencing in paediatric neurological disorders. Dev Med Child Neurol.

[39] Deltas C (2018). Digenic inheritance and genetic modifiers. Clin Genet.

[40] Gong T, Hayes VM, Chan EKF (2021). Detection of somatic structural variants from short-read next-generation sequencing data. Brief Bioinform.

