# Supplementary material for: Copy number variants from 4800 exomes contribute to ~7% of genetic diagnoses in movement disorders, muscle disorders and neuropathies
Source: Eur J Hum Genet. 2023 Feb 13;31(6):654–62. doi: 10.1038/s41431-023-01312-0 (PMC10250492; doi:10.1038/s41431-023-01312-0)
Supplement: Supplementary file 1 — Sup. Figure 1 [file 41431_2023_1312_MOESM1_ESM.pptx]

## Slide 1
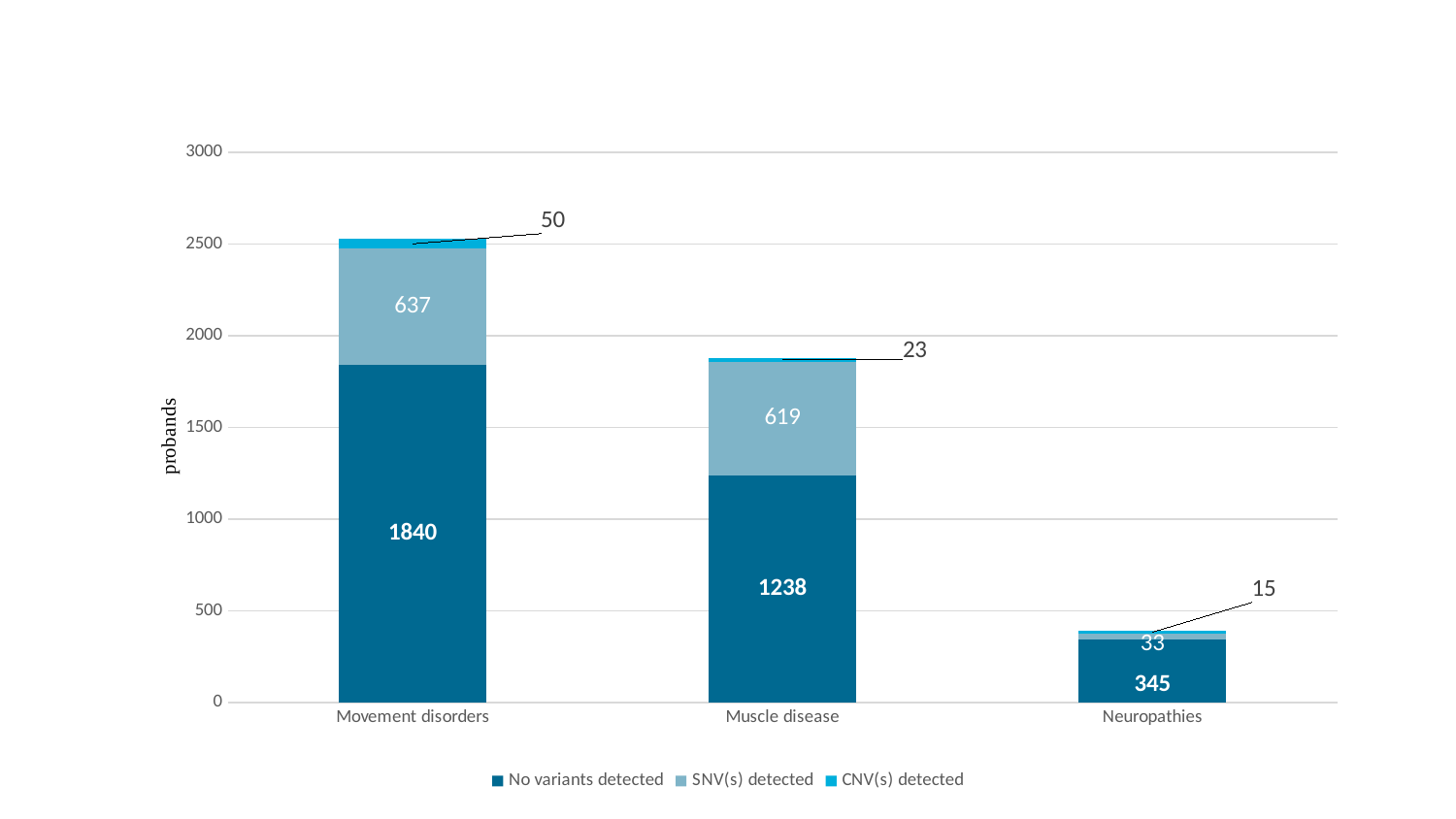

### Chart
| Category | No variants detected | SNV(s) detected | CNV(s) detected |
|---|---|---|---|
| Movement disorders | 1840.0 | 635.0 | 52.0 |
| Muscle disease | 1238.0 | 618.0 | 24.0 |
| Neuropathies | 345.0 | 32.0 | 16.0 |
