# Supplementary material for: Copy number variants from 4800 exomes contribute to ~7% of genetic diagnoses in movement disorders, muscle disorders and neuropathies
Source: Eur J Hum Genet. 2023 Feb 13;31(6):654–62. doi: 10.1038/s41431-023-01312-0 (PMC10250492; doi:10.1038/s41431-023-01312-0)
Supplement: Supplementary file 2 — Sup. Figure 2 [file 41431_2023_1312_MOESM2_ESM.pptx]

## Slide 1
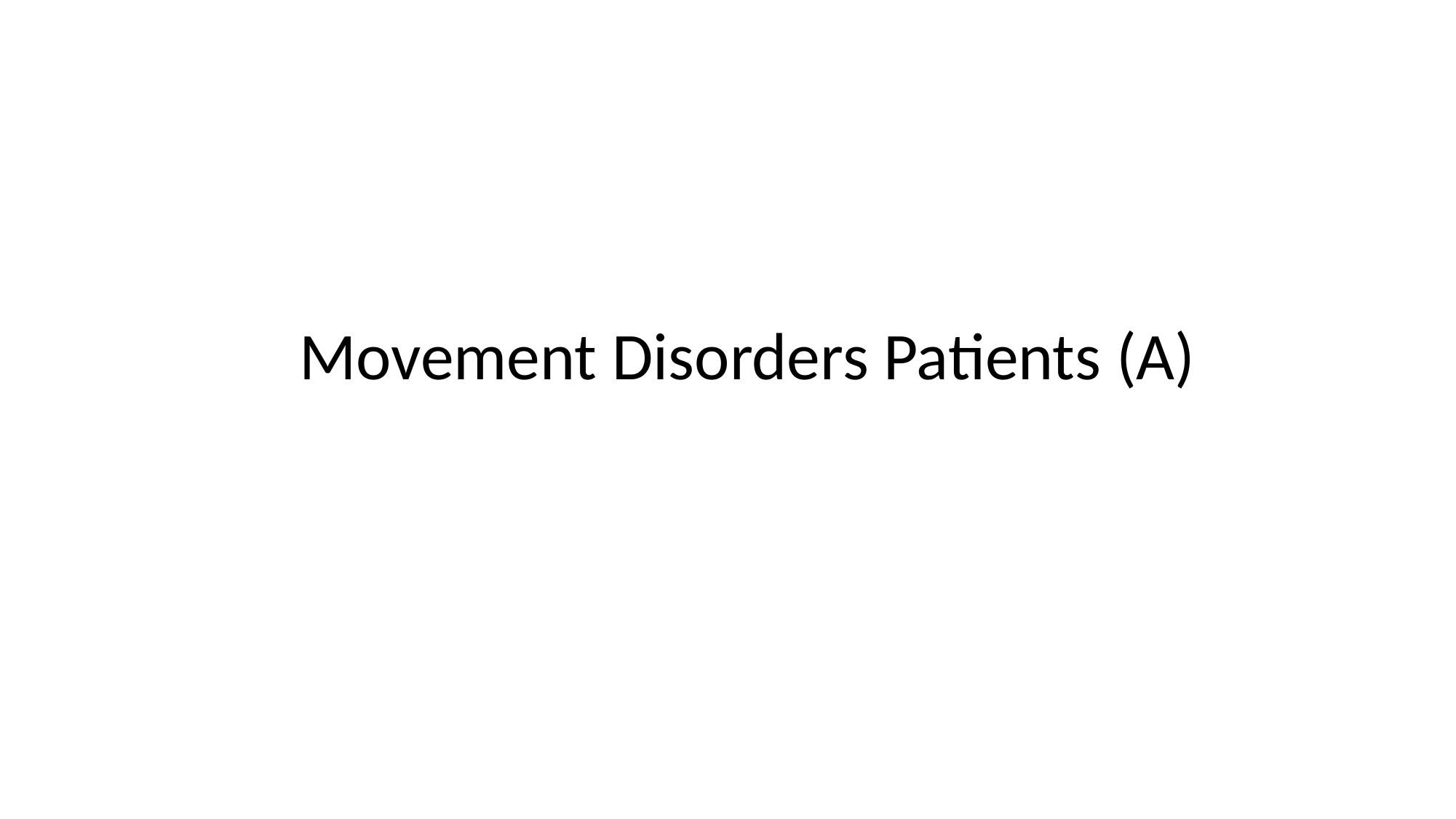

Movement Disorders Patients (A)

## Slide 2
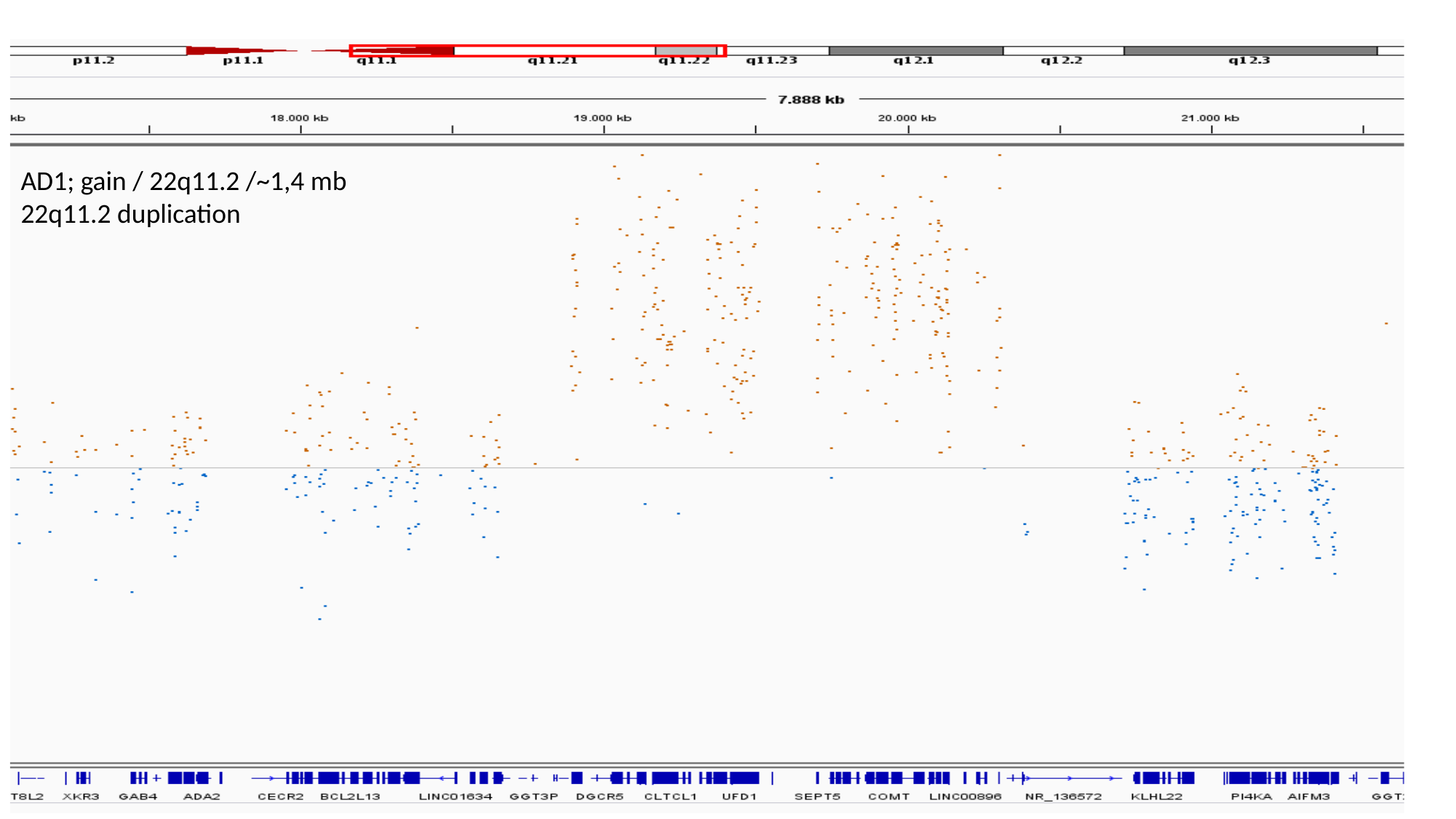

AD1; gain / 22q11.2 /~1,4 mb
22q11.2 duplication

## Slide 3
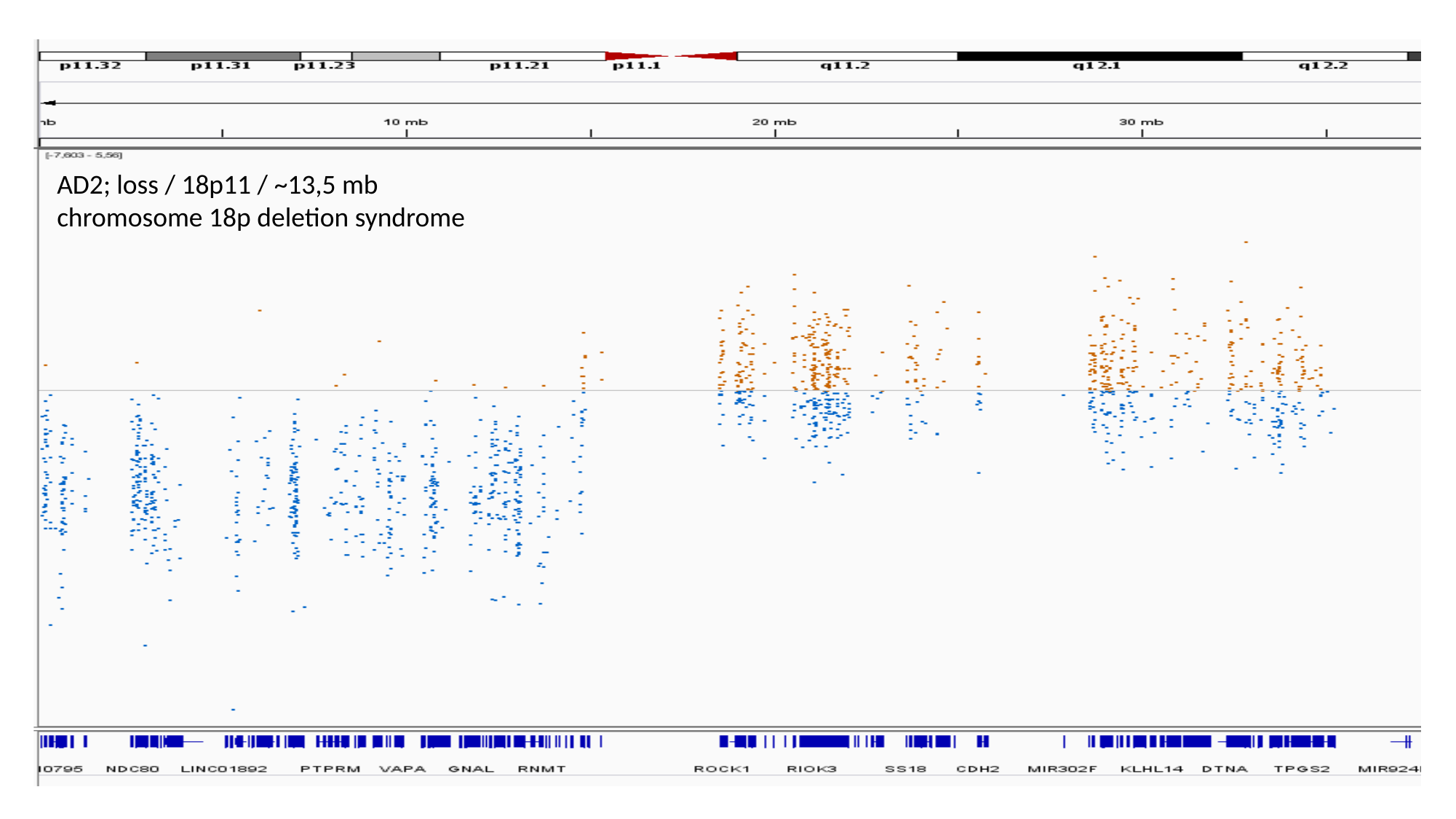

AD2; loss / 18p11 / ~13,5 mb
chromosome 18p deletion syndrome

## Slide 4
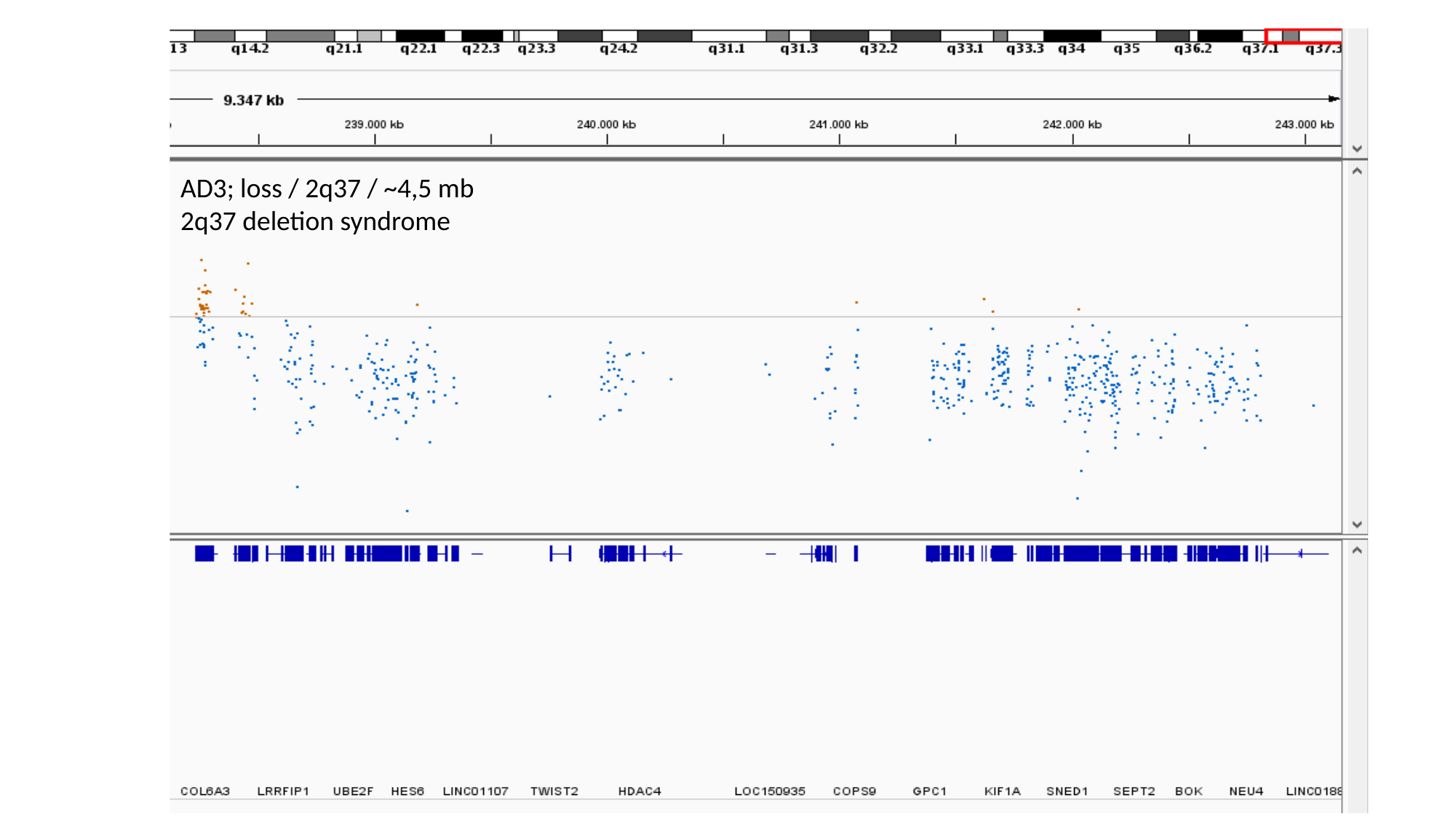

AD3; loss / 2q37 / ~4,5 mb
2q37 deletion syndrome

## Slide 5
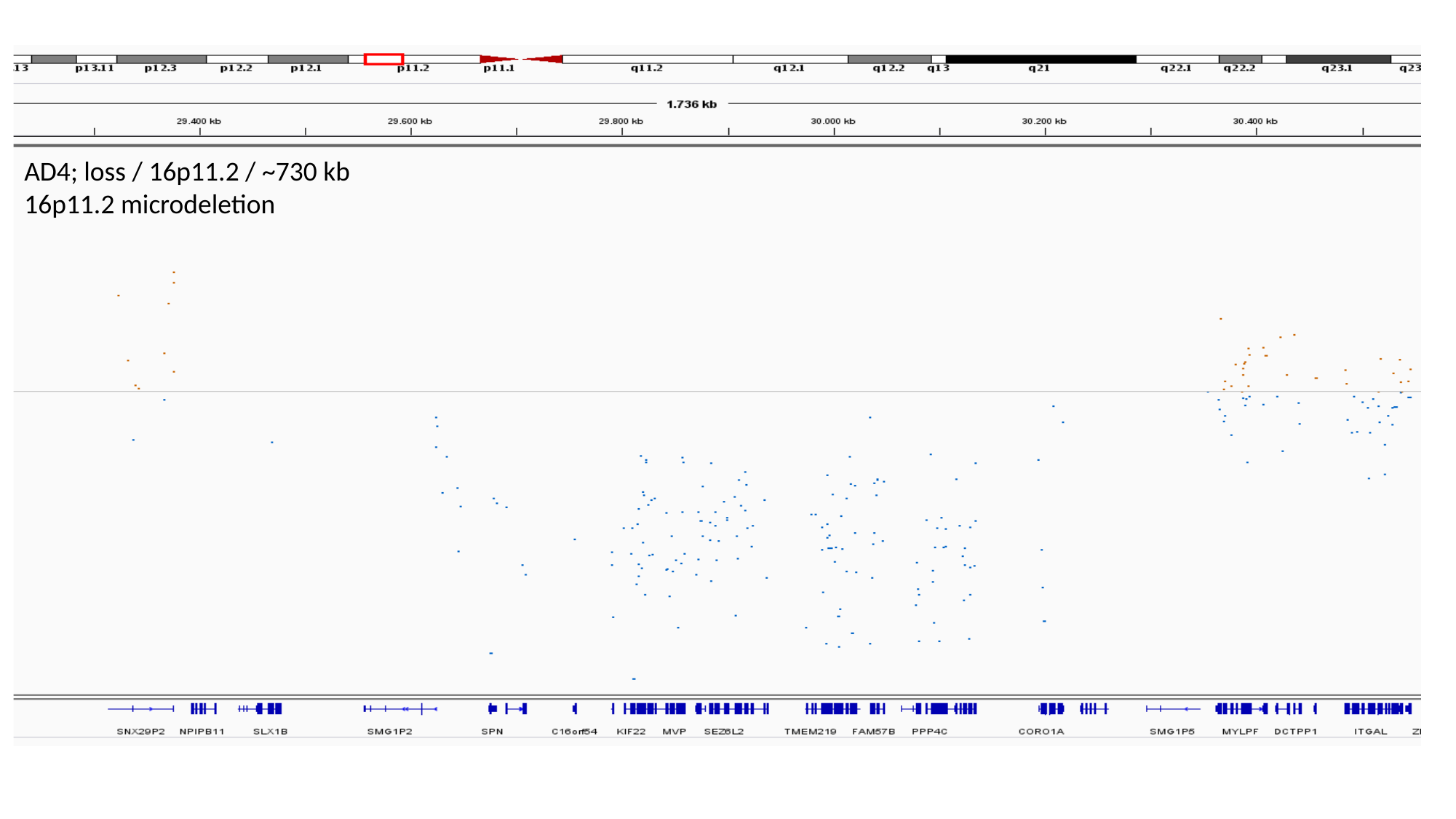

AD4; loss / 16p11.2 / ~730 kb
16p11.2 microdeletion

## Slide 6
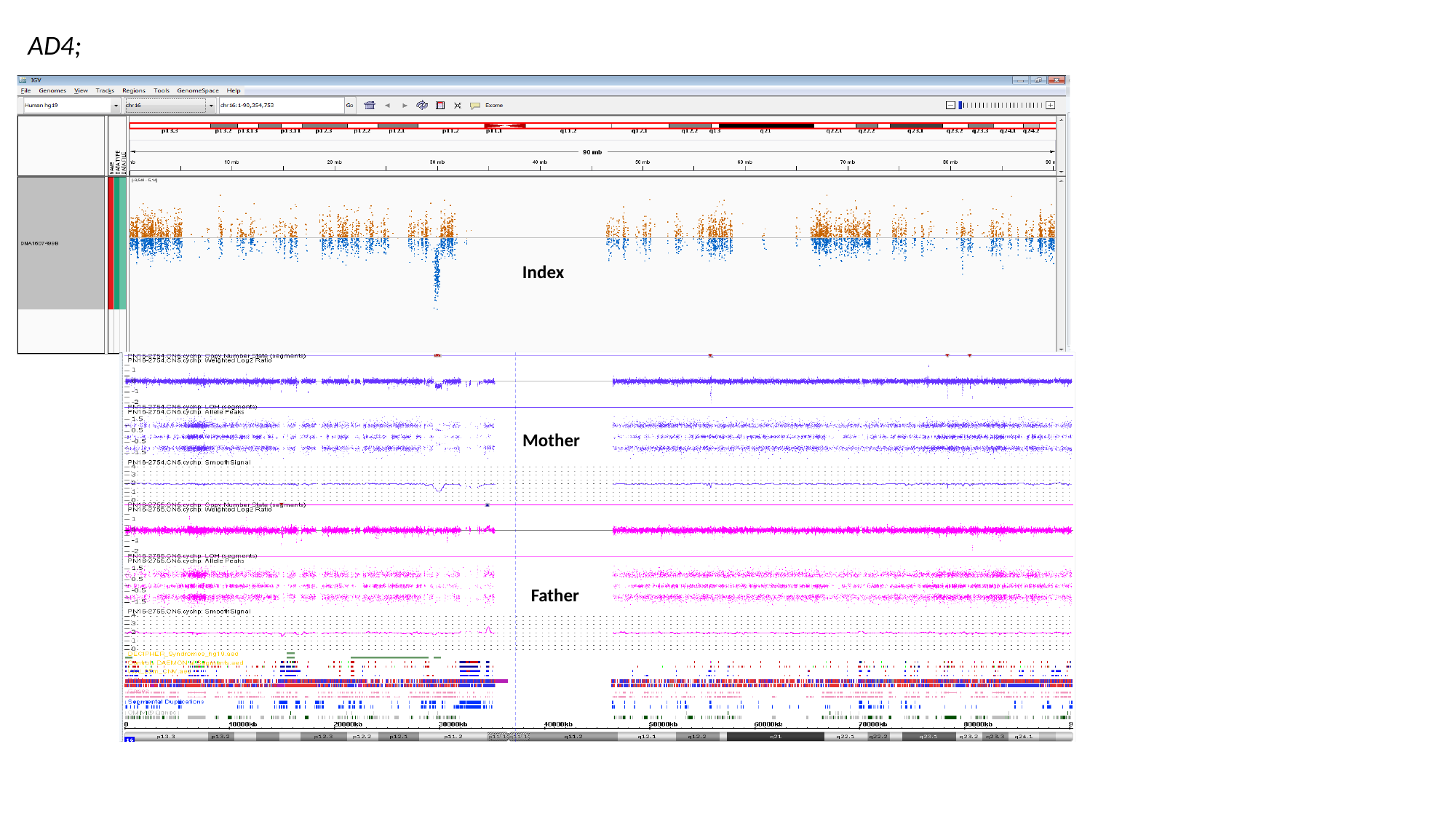

AD4;
Index
Mother
Father

## Slide 7
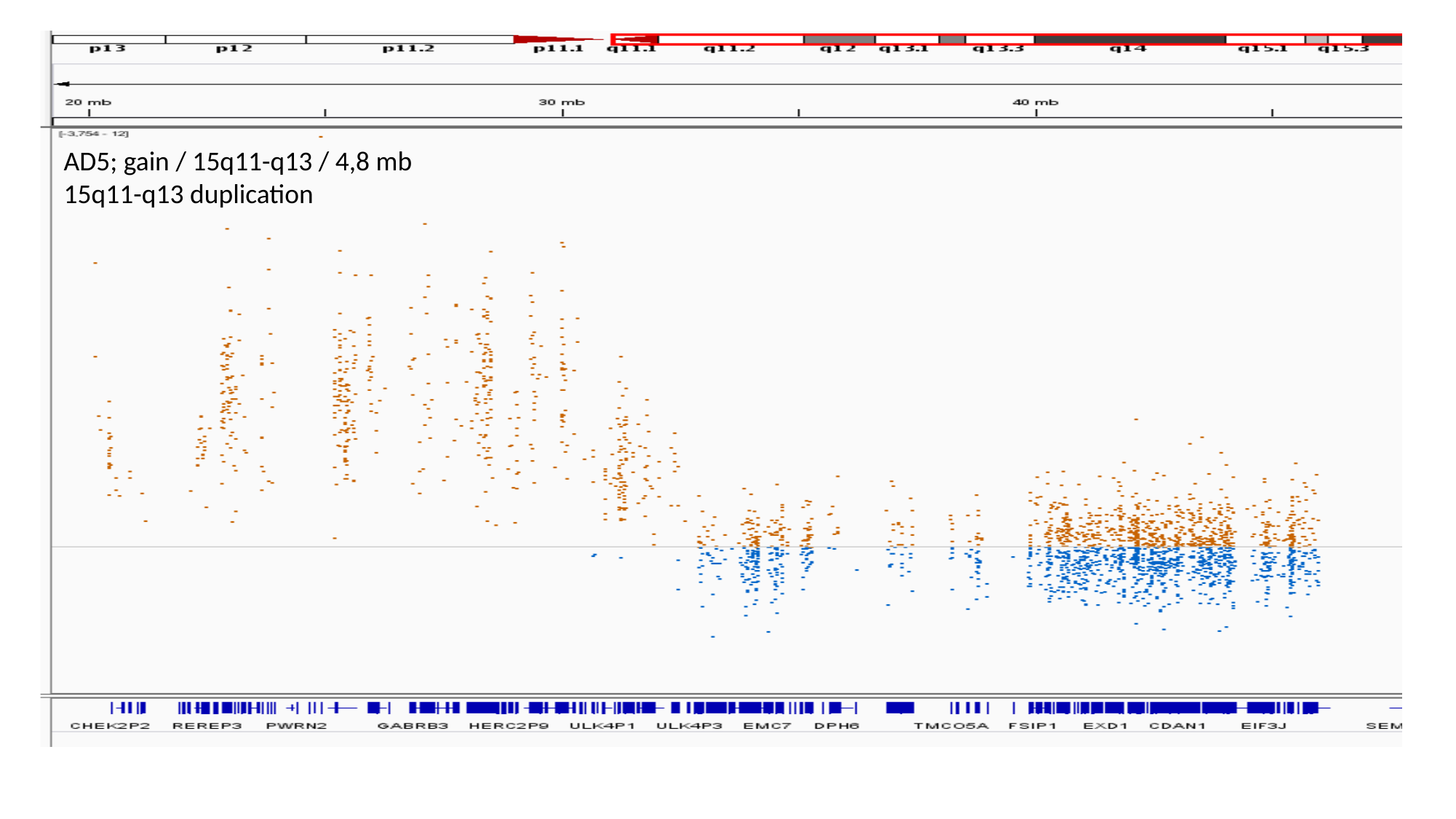

AD5; gain / 15q11-q13 / 4,8 mb
15q11-q13 duplication

## Slide 8
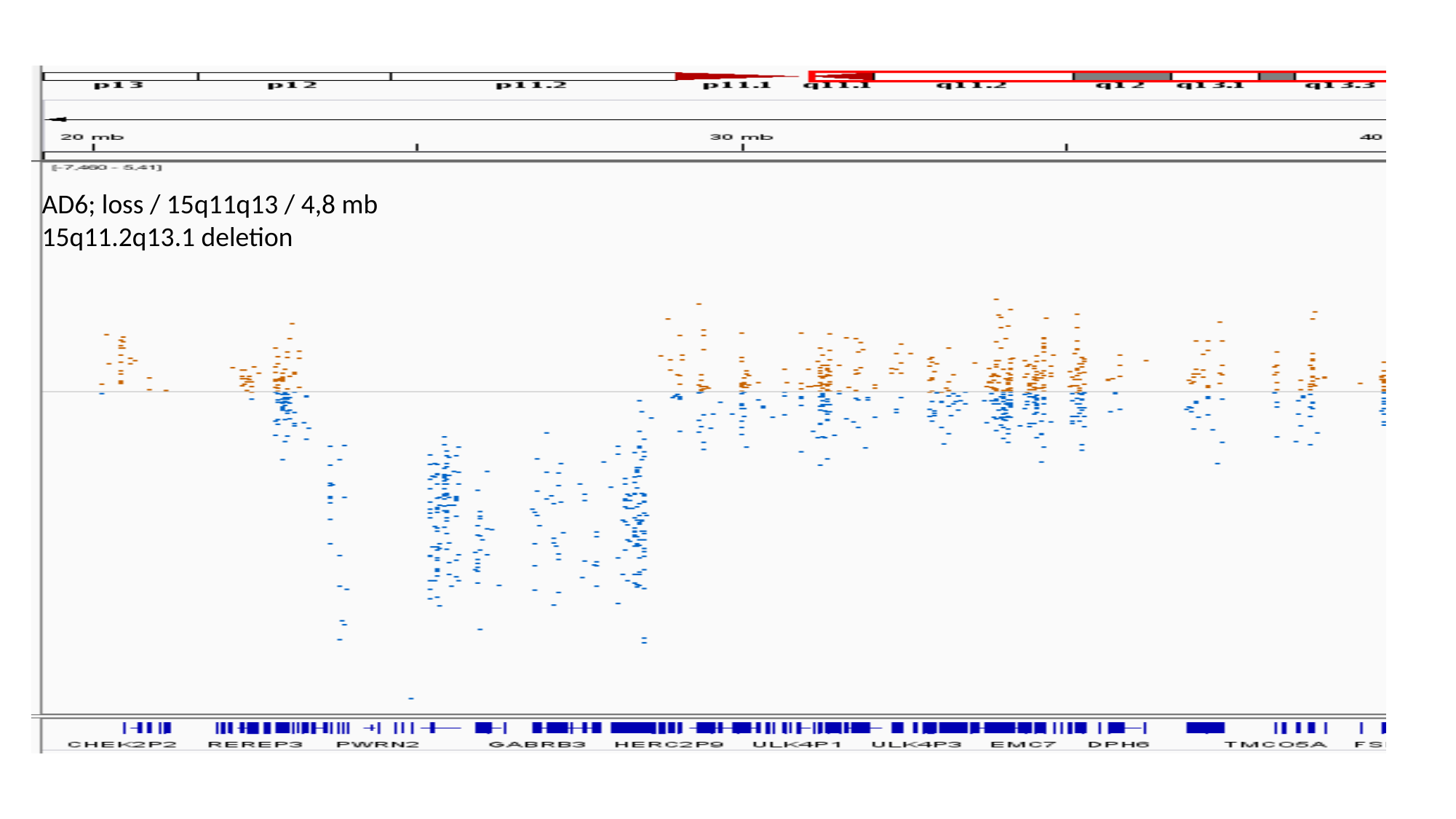

AD6; loss / 15q11q13 / 4,8 mb
15q11.2q13.1 deletion

## Slide 9
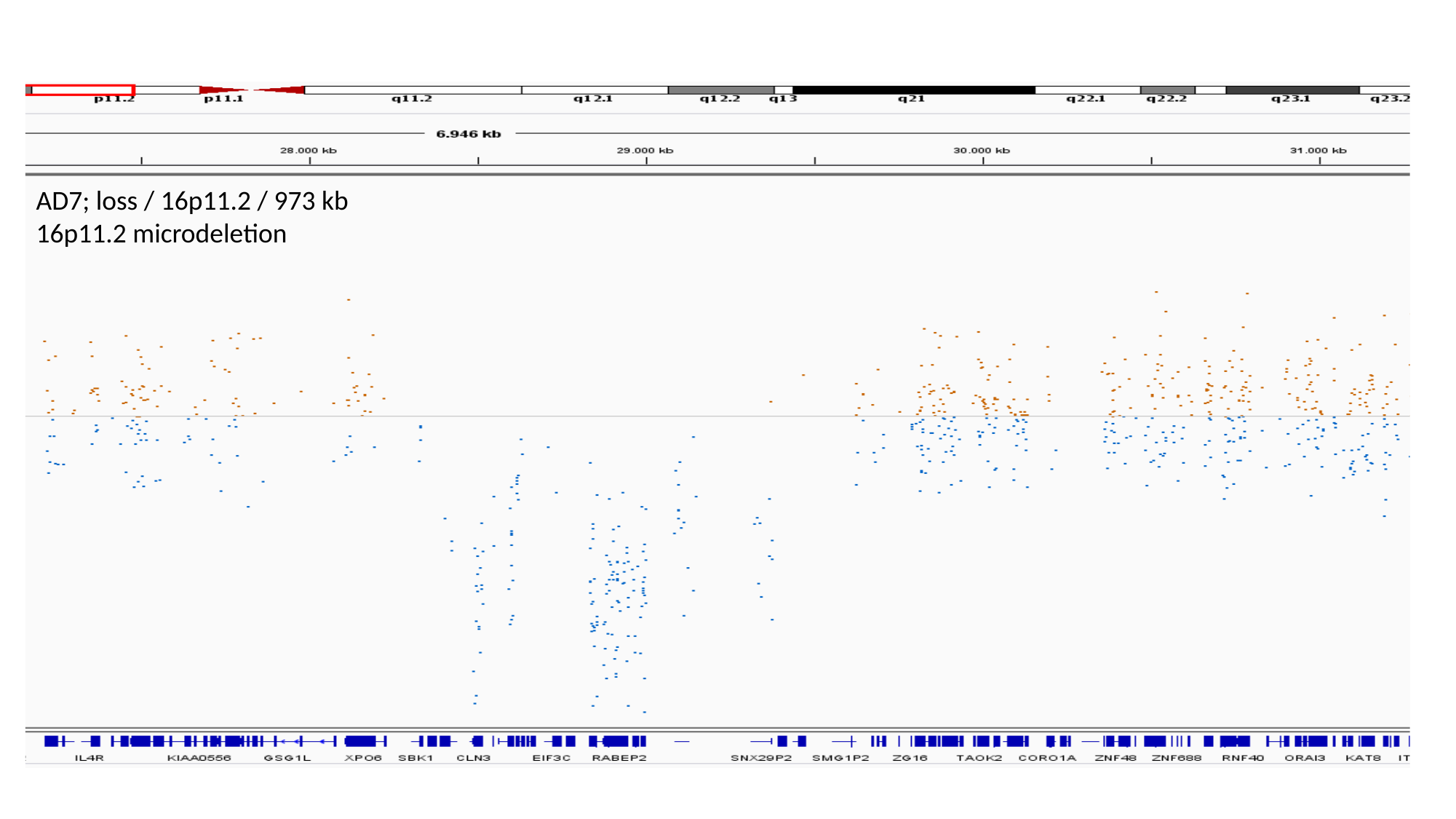

AD7; loss / 16p11.2 / 973 kb
16p11.2 microdeletion

## Slide 10
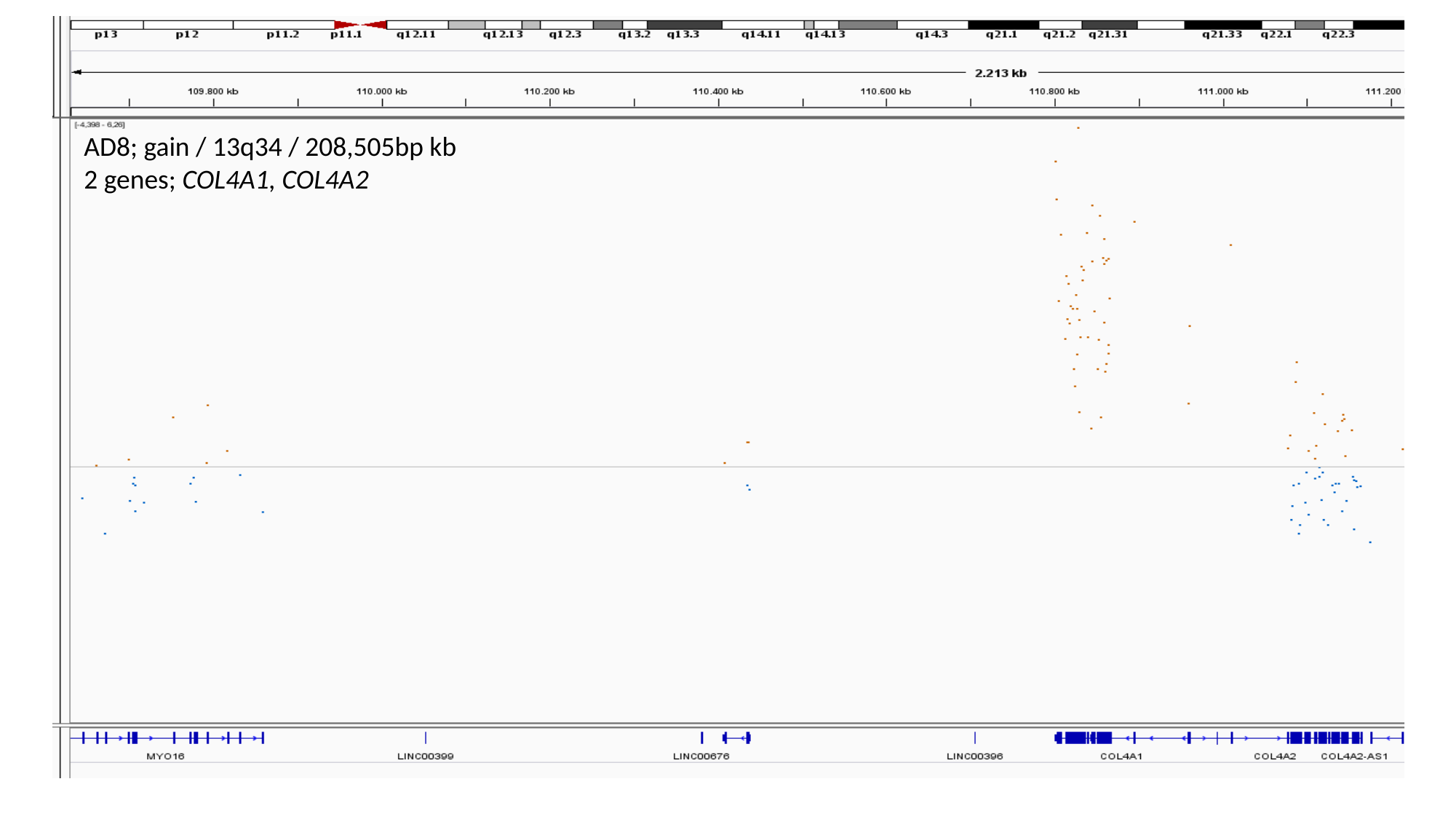

AD8; gain / 13q34 / 208,505bp kb
2 genes; COL4A1, COL4A2

## Slide 11
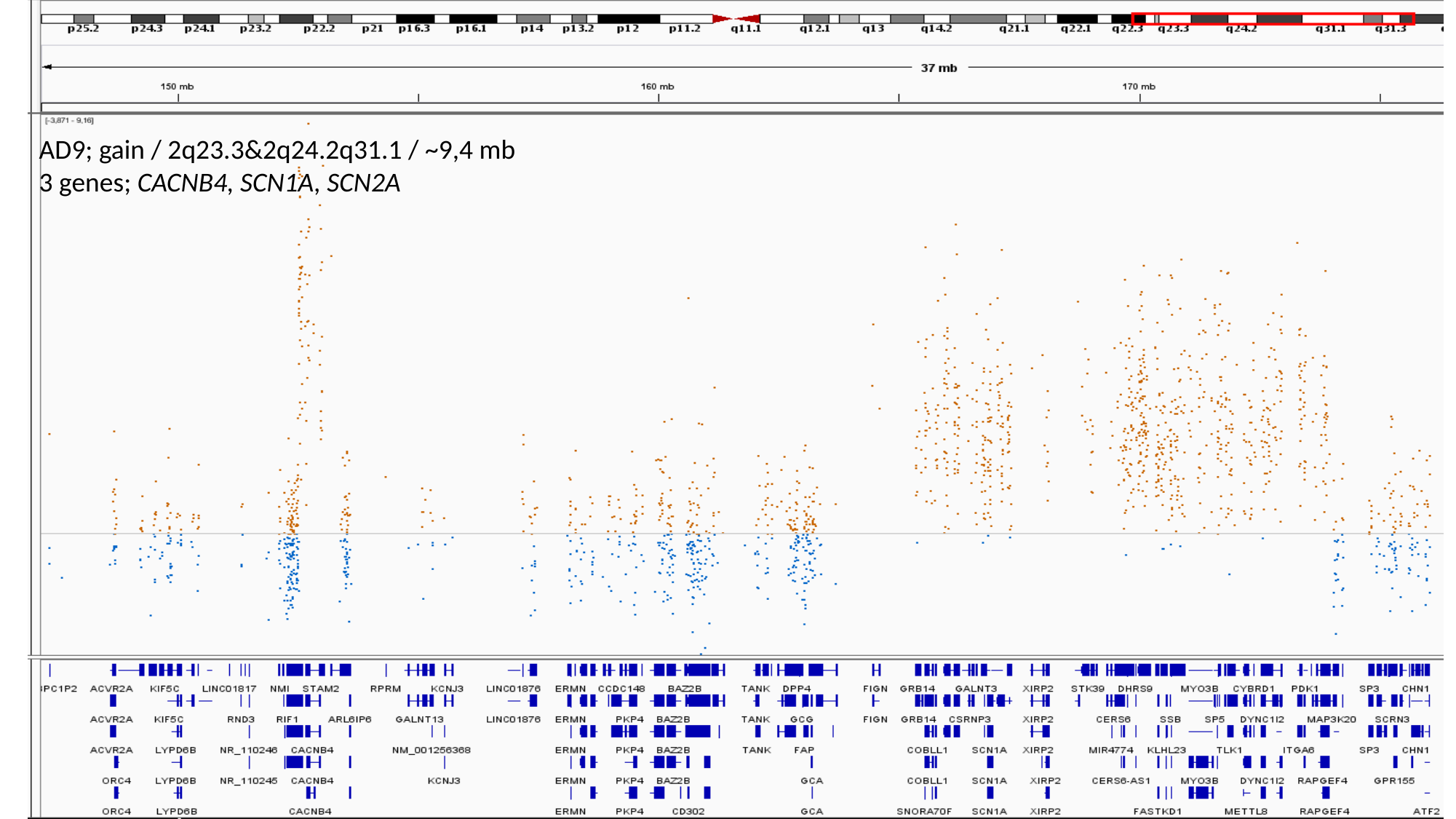

AD9; gain / 2q23.3&2q24.2q31.1 / ~9,4 mb
3 genes; CACNB4, SCN1A, SCN2A

## Slide 12
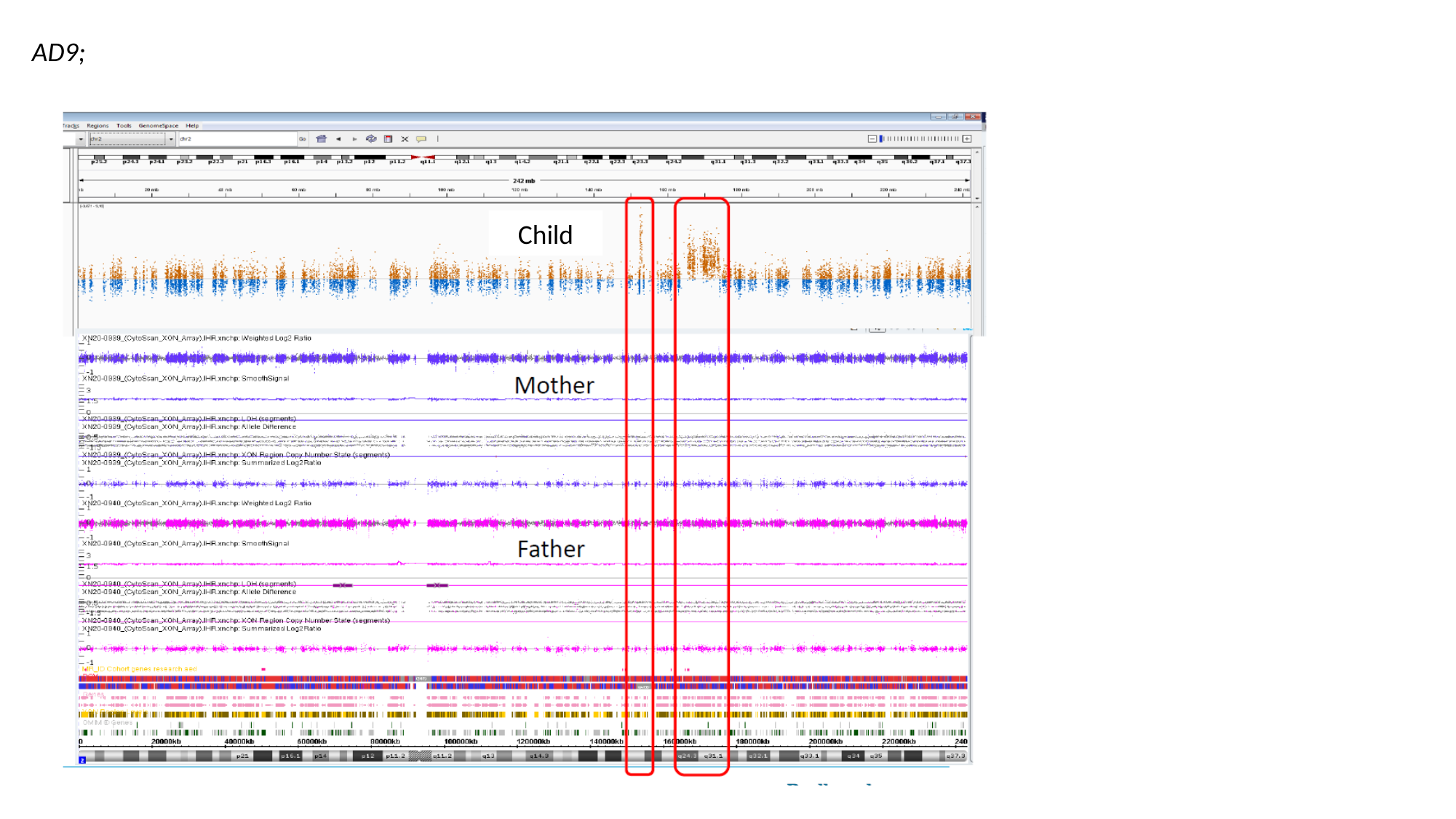

AD9;
Child

## Slide 13
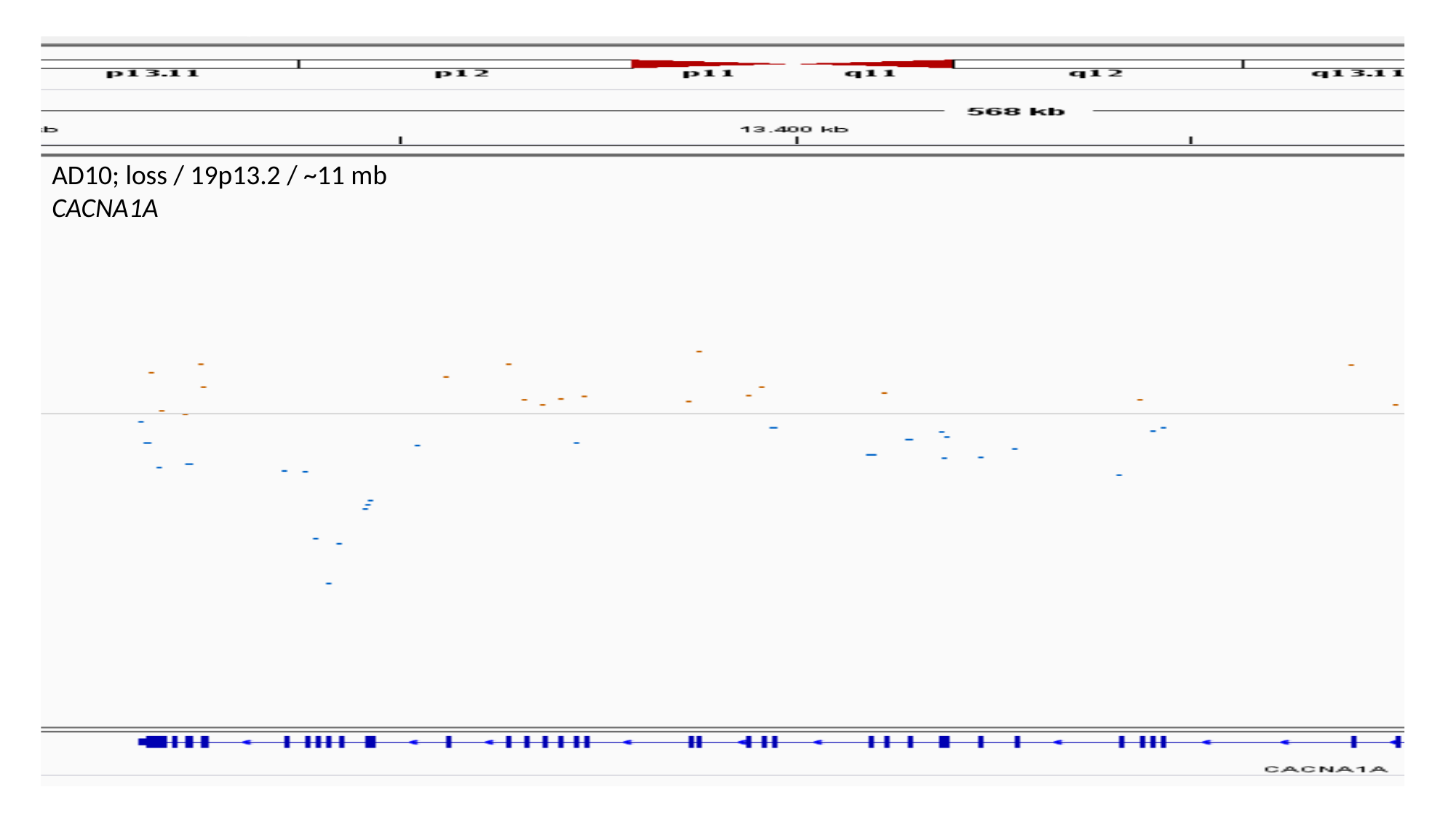

AD10; loss / 19p13.2 / ~11 mb
CACNA1A

## Slide 14
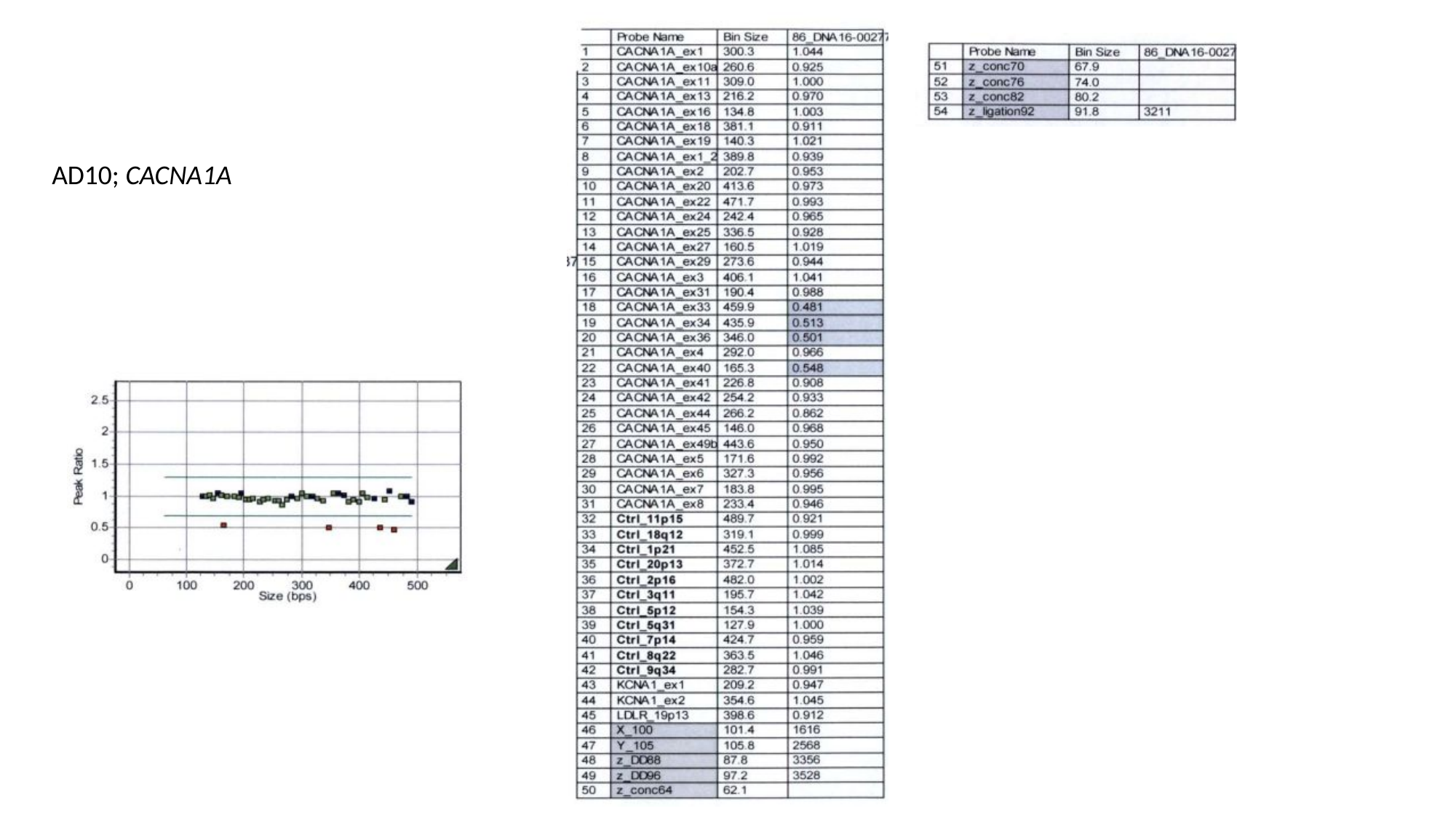

AD10; CACNA1A

## Slide 15
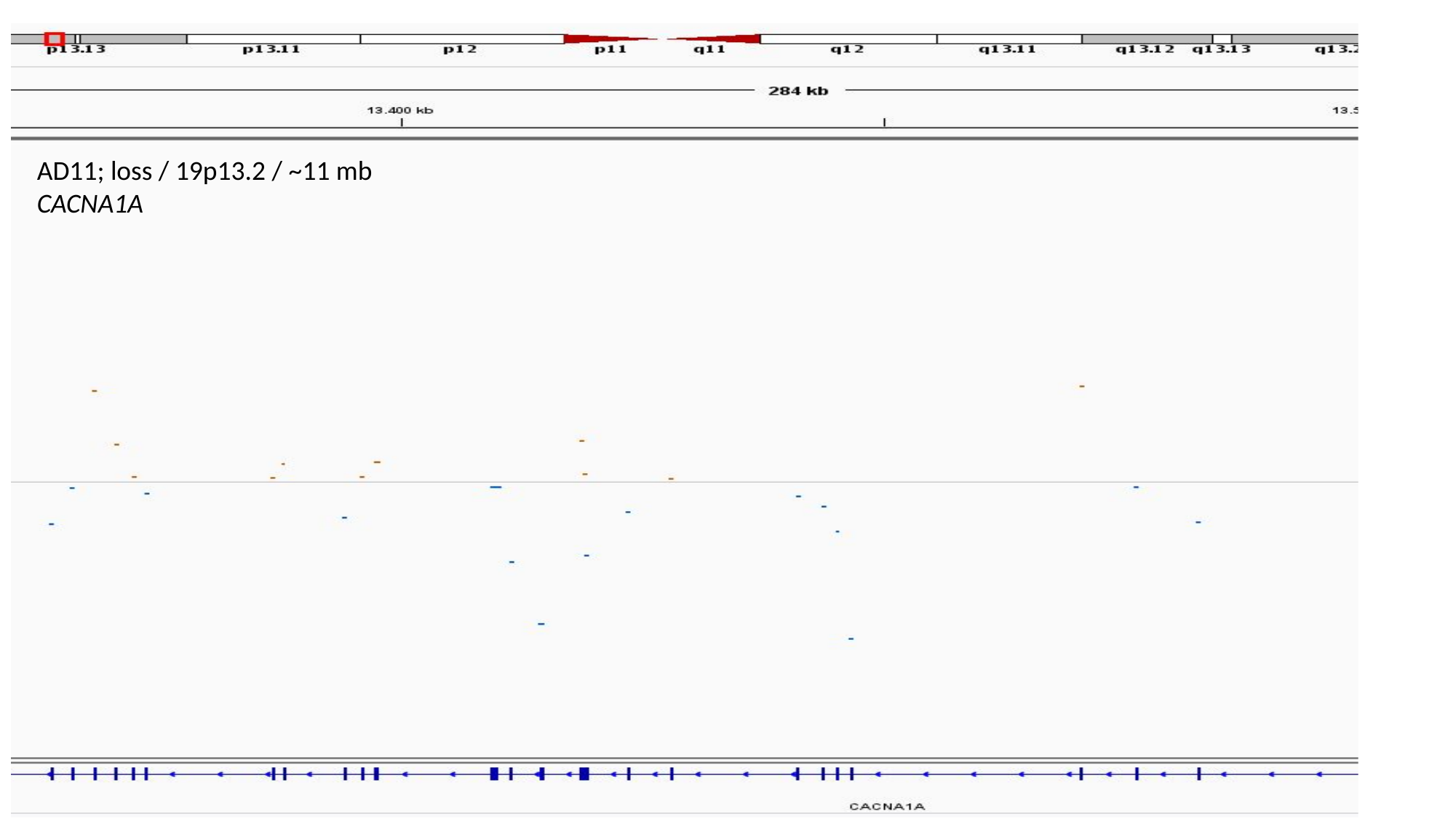

AD11; loss / 19p13.2 / ~11 mb
CACNA1A

## Slide 16
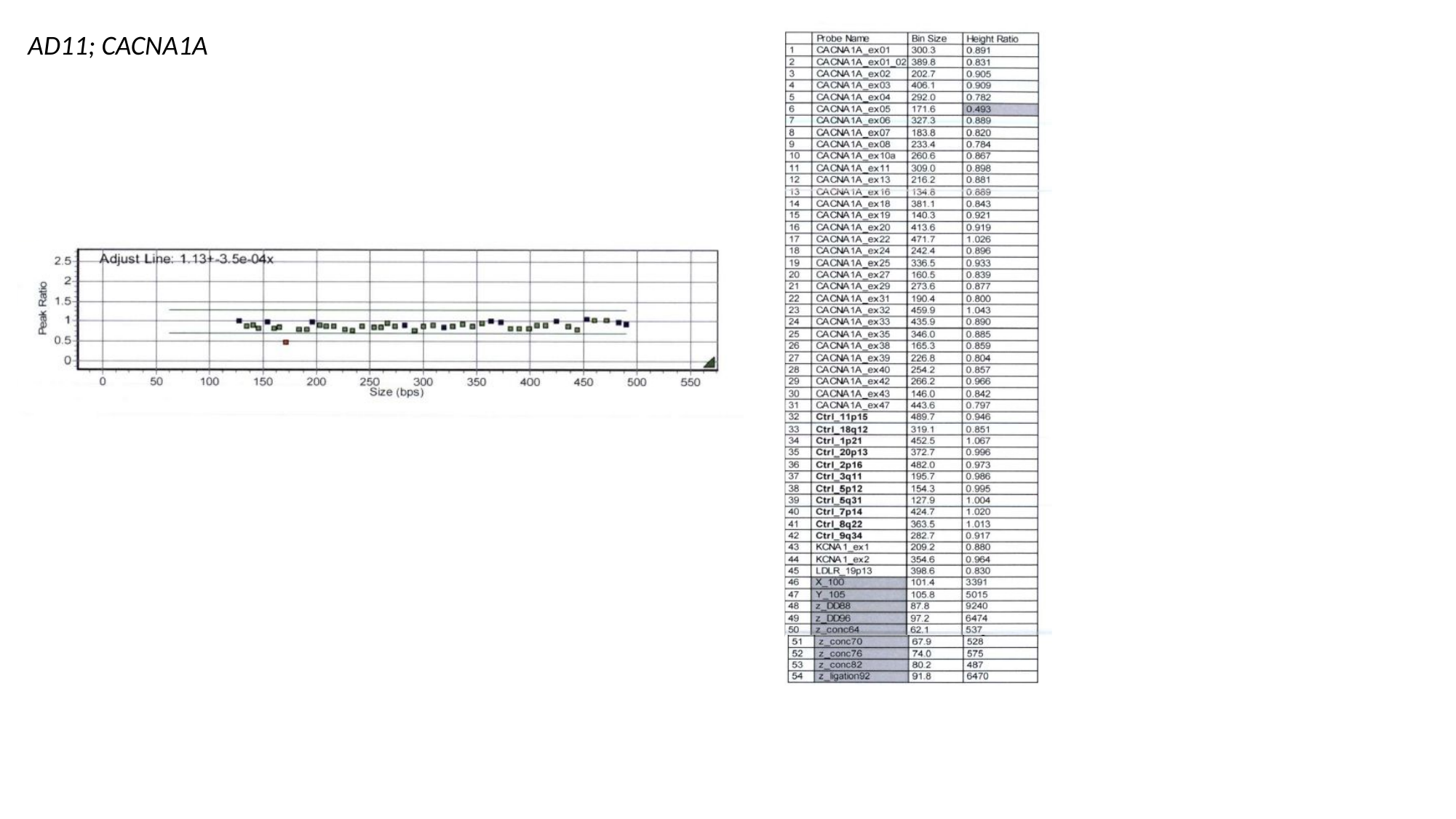

AD11; CACNA1A

## Slide 17
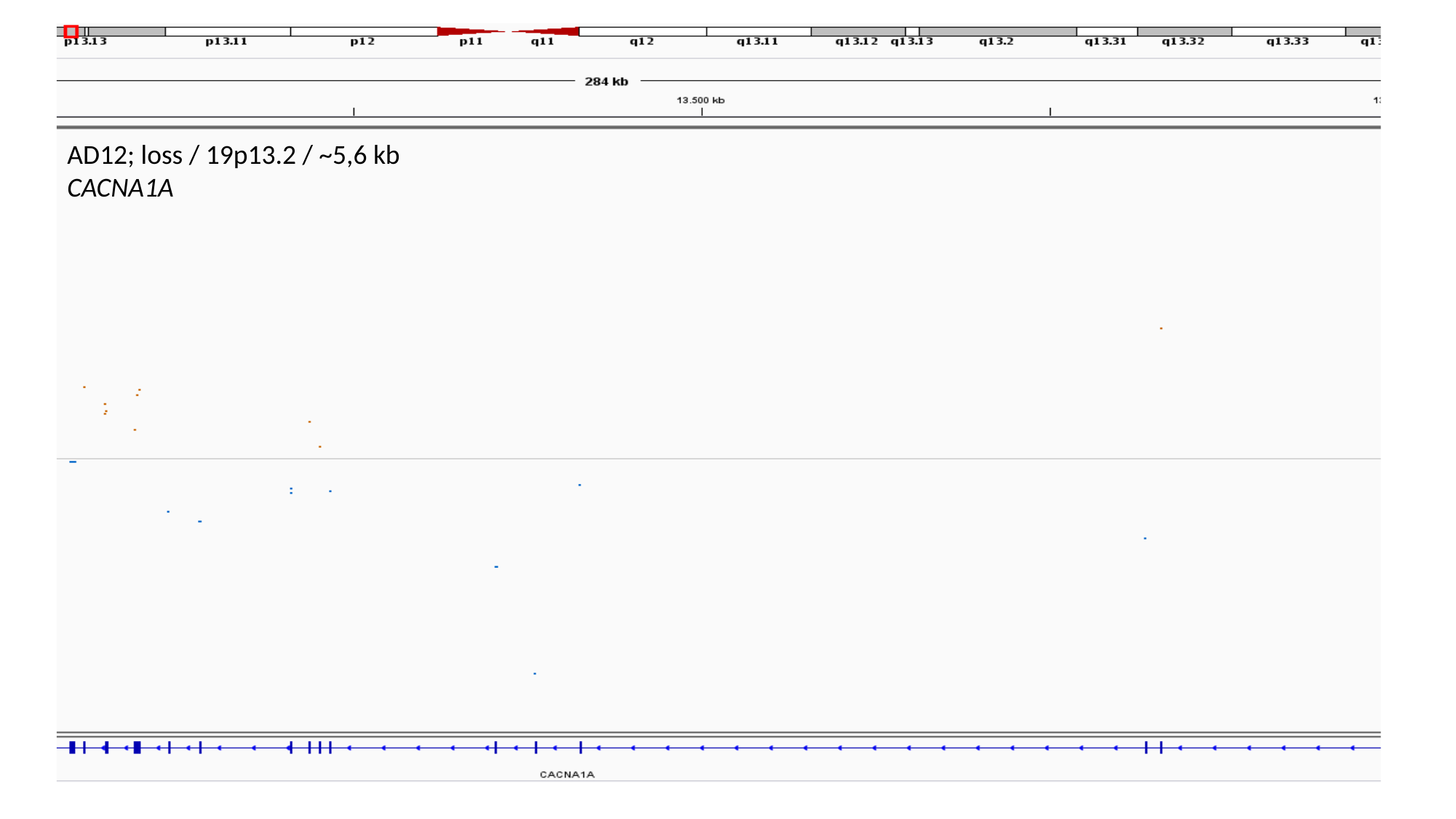

AD12; loss / 19p13.2 / ~5,6 kb
CACNA1A

## Slide 18
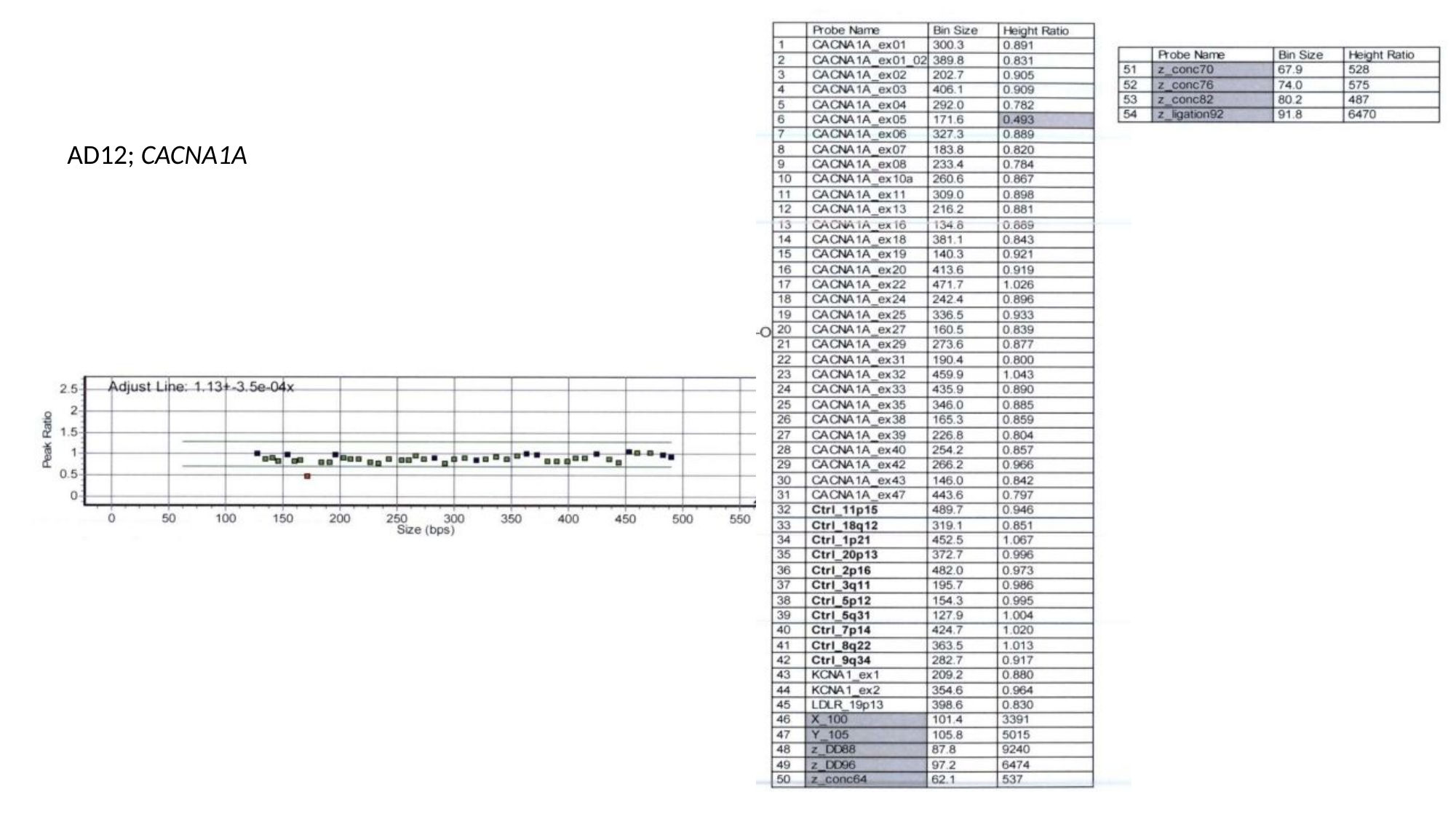

AD12; CACNA1A

## Slide 19
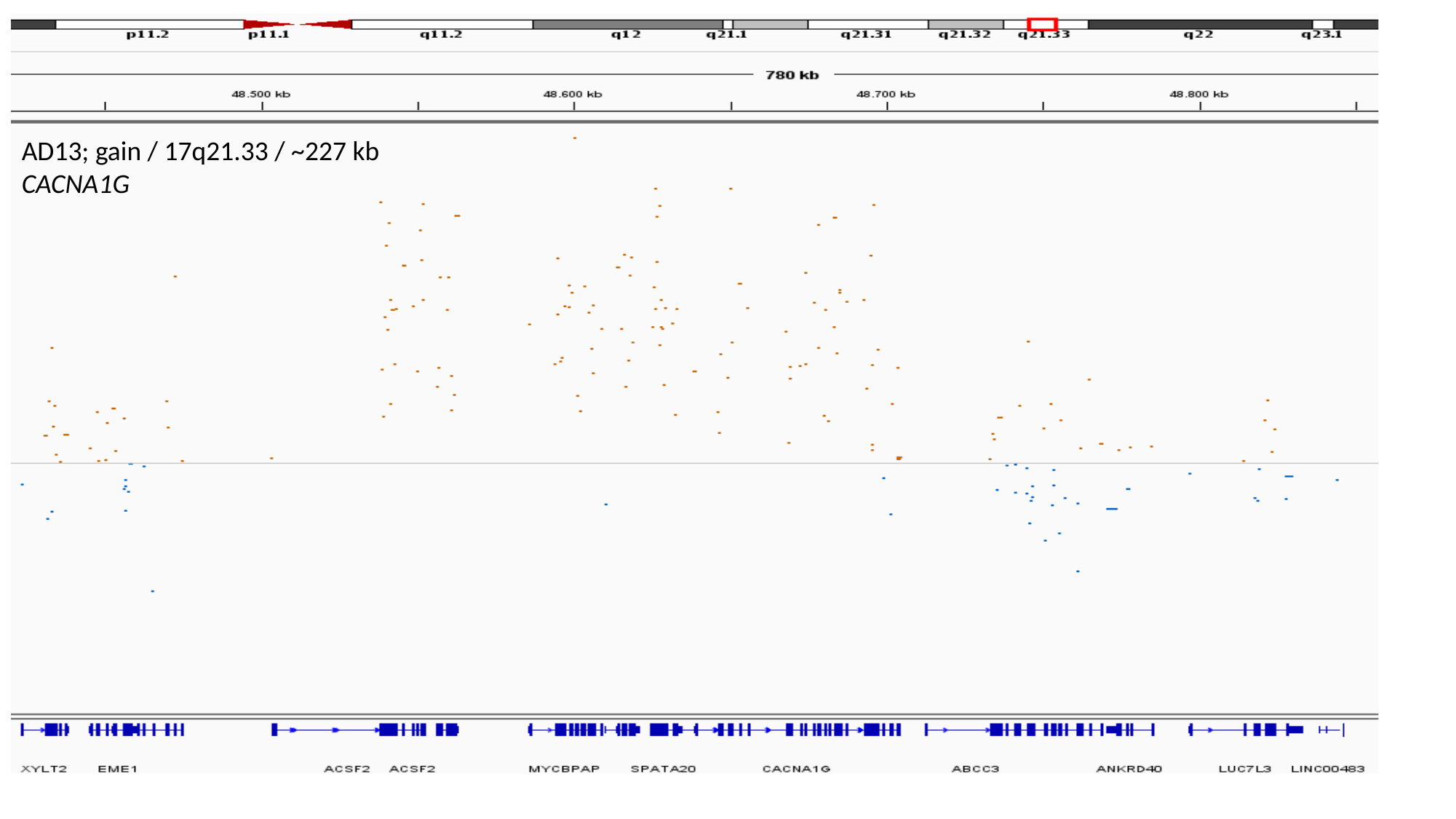

AD13; gain / 17q21.33 / ~227 kb
CACNA1G

## Slide 20
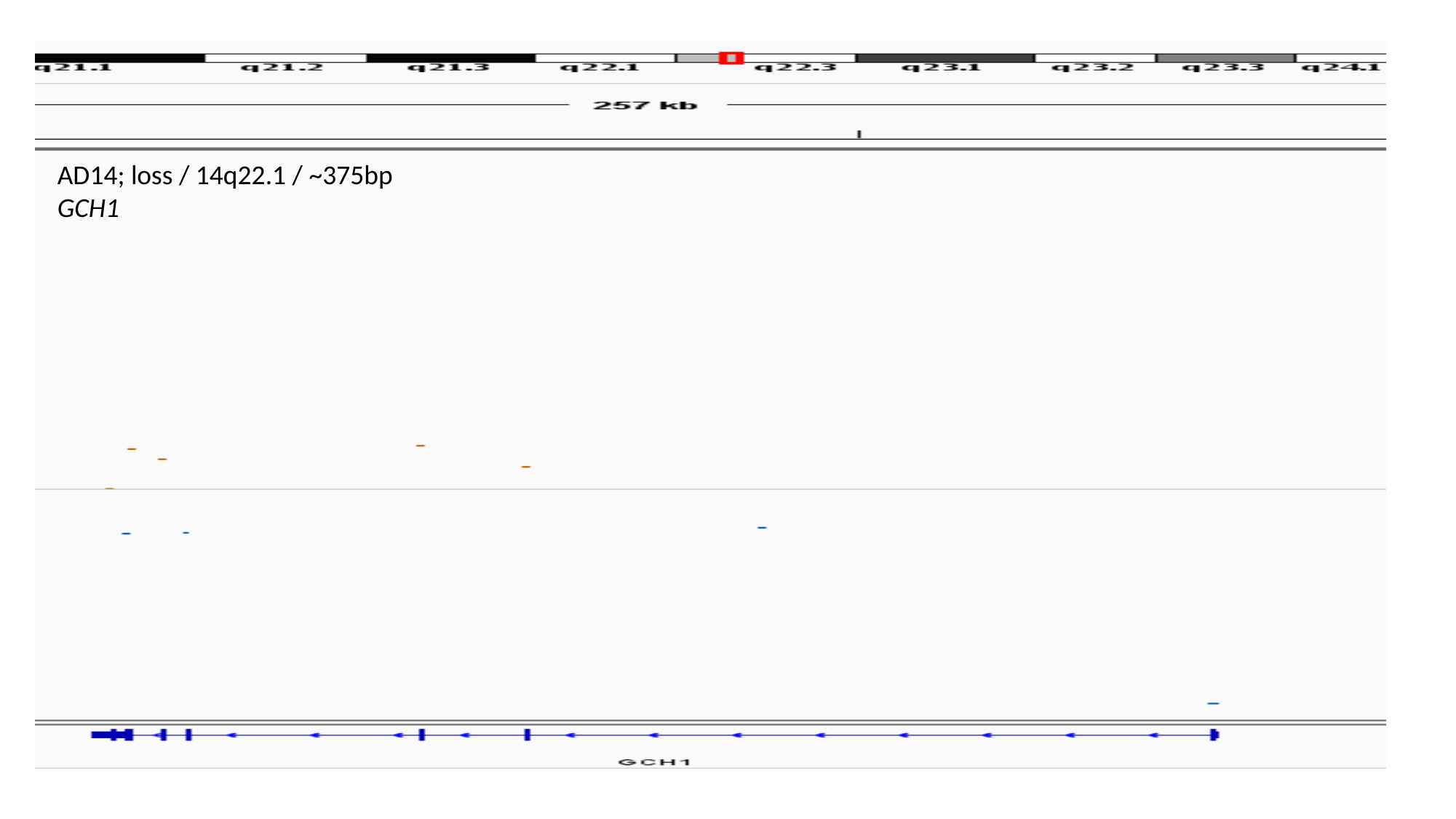

AD14; loss / 14q22.1 / ~375bp GCH1

## Slide 21
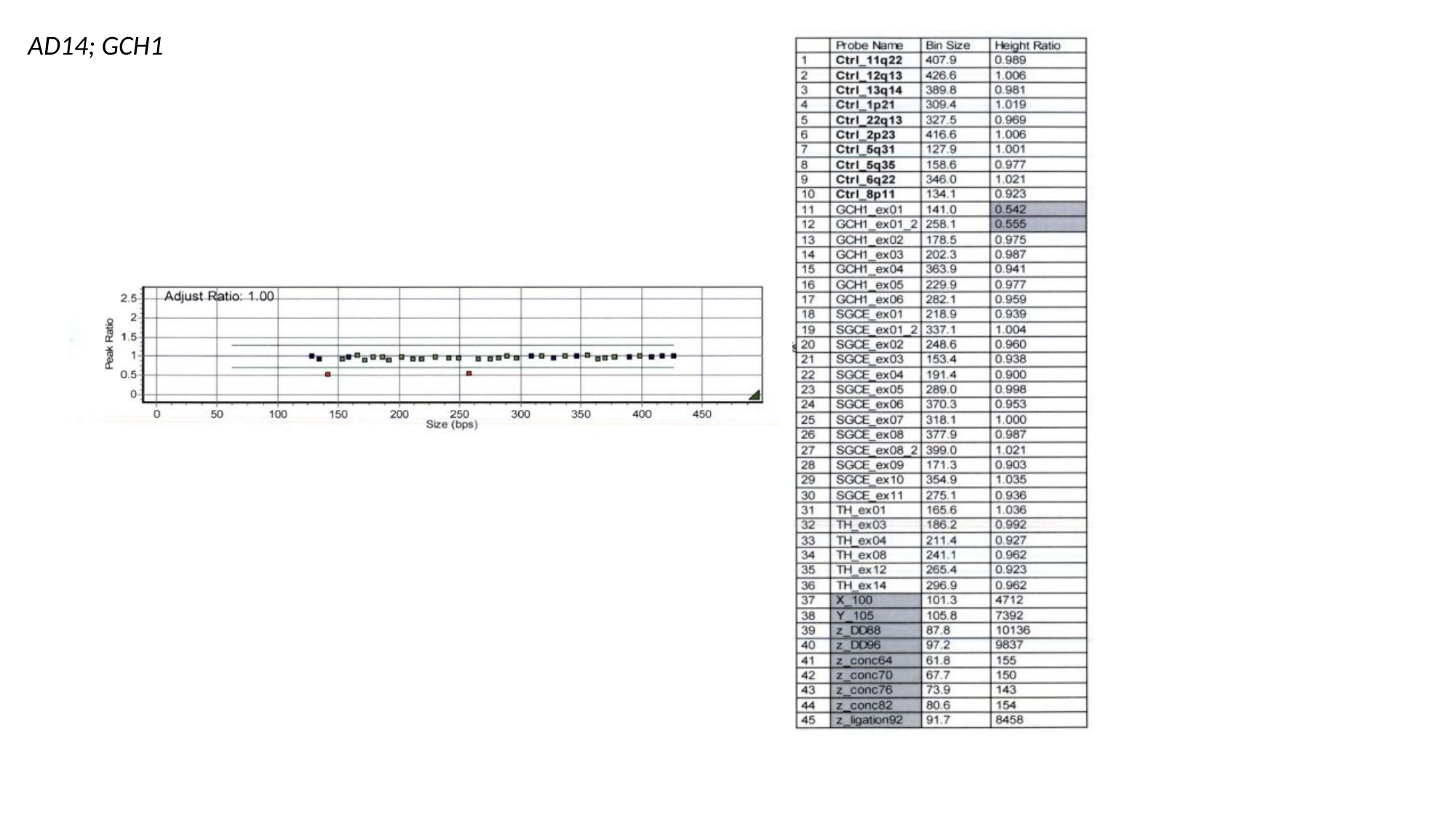

AD14; GCH1

## Slide 22
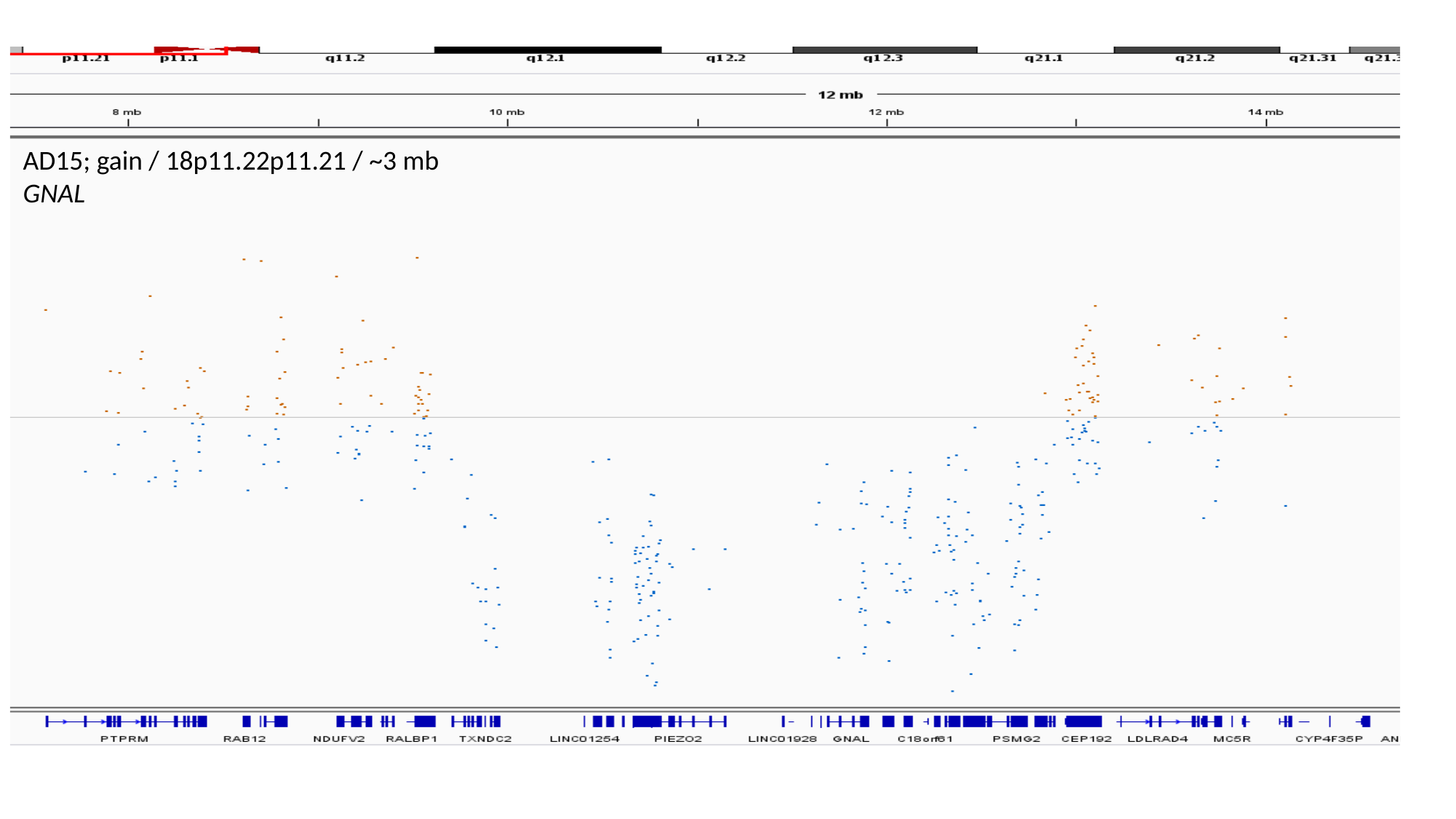

AD15; gain / 18p11.22p11.21 / ~3 mb
GNAL

## Slide 23
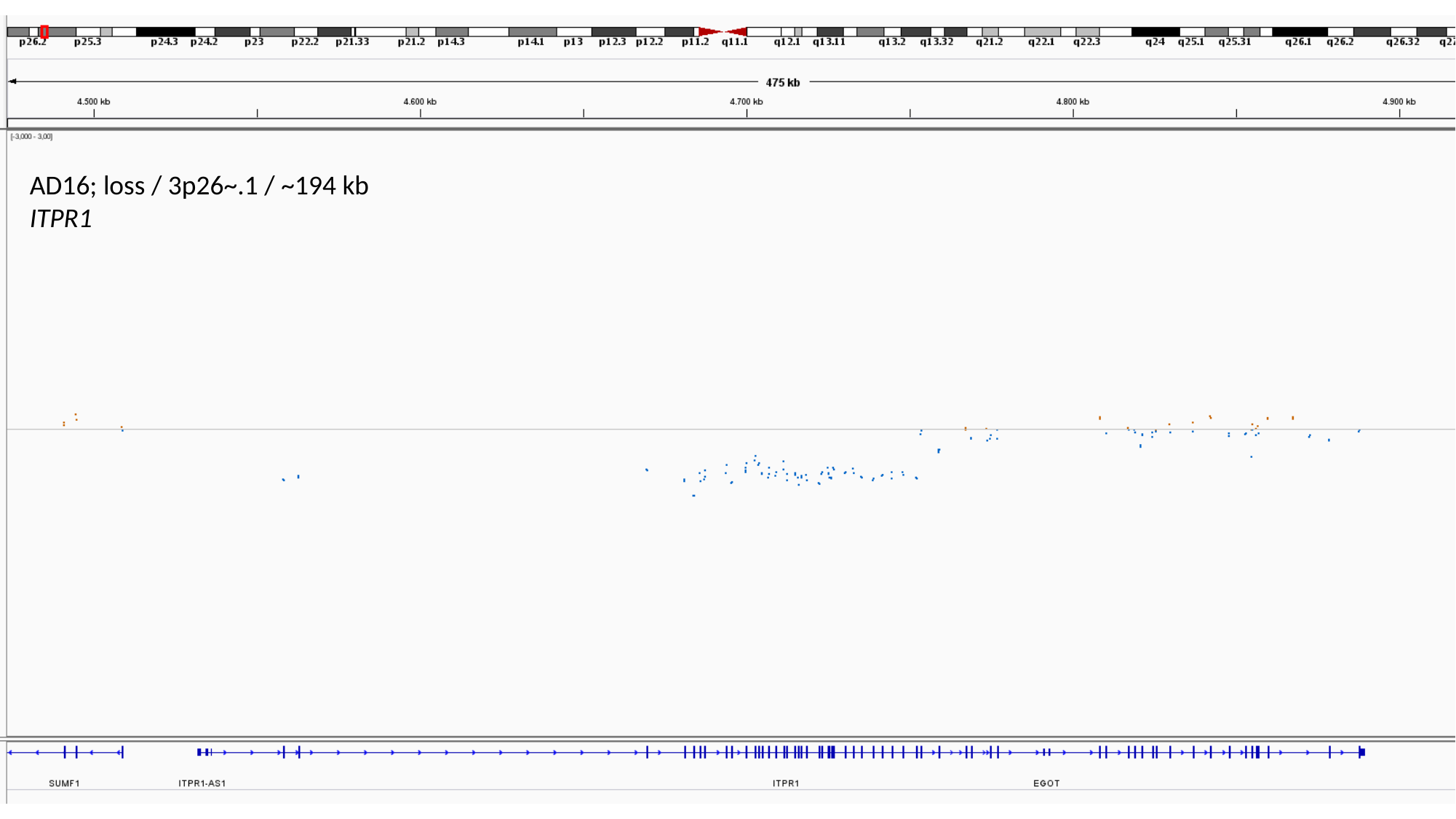

AD16; loss / 3p26~.1 / ~194 kb
ITPR1

## Slide 24
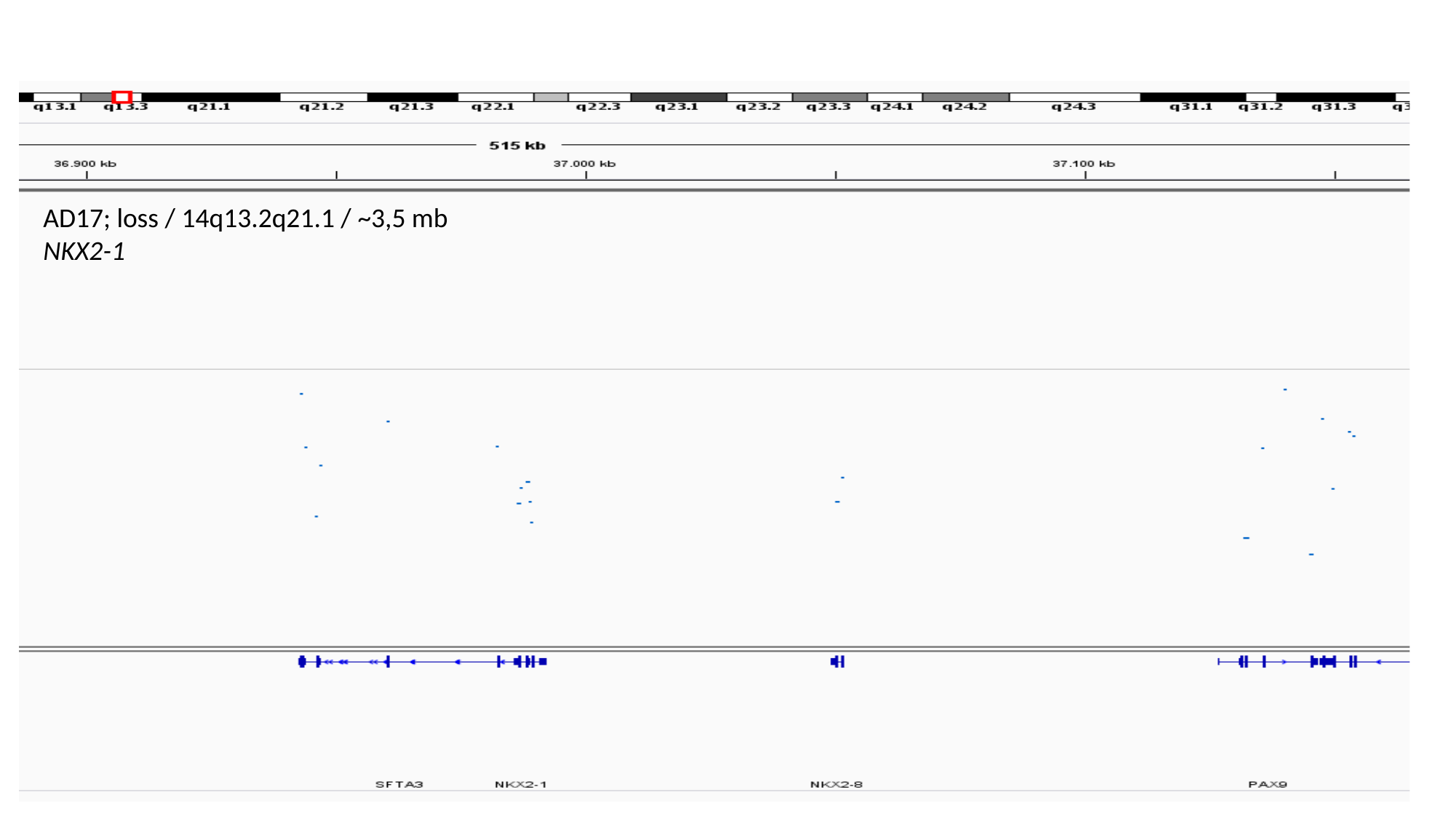

AD17; loss / 14q13.2q21.1 / ~3,5 mb
NKX2-1

## Slide 25
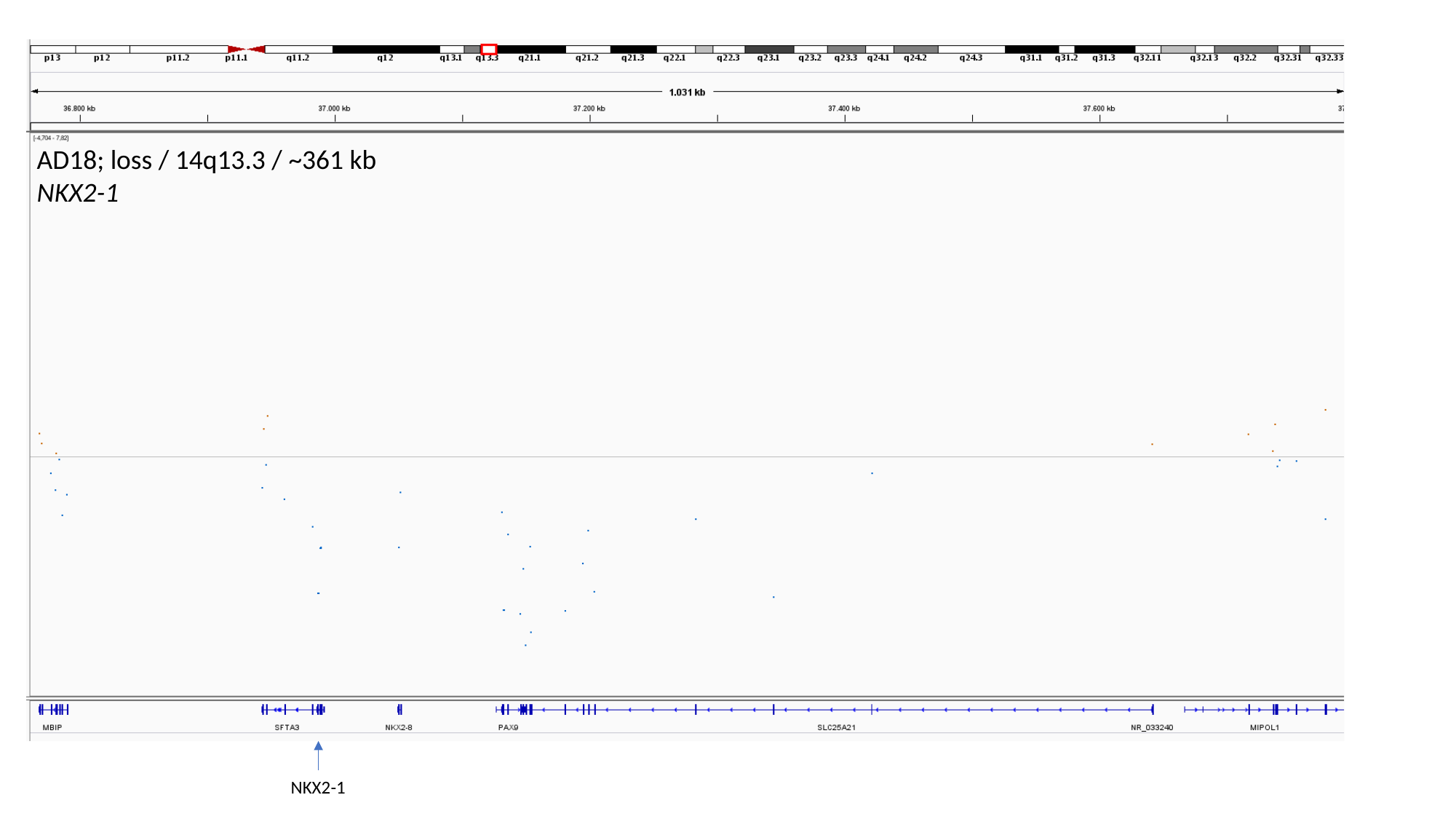

AD18; loss / 14q13.3 / ~361 kb
NKX2-1
NKX2-1

## Slide 26
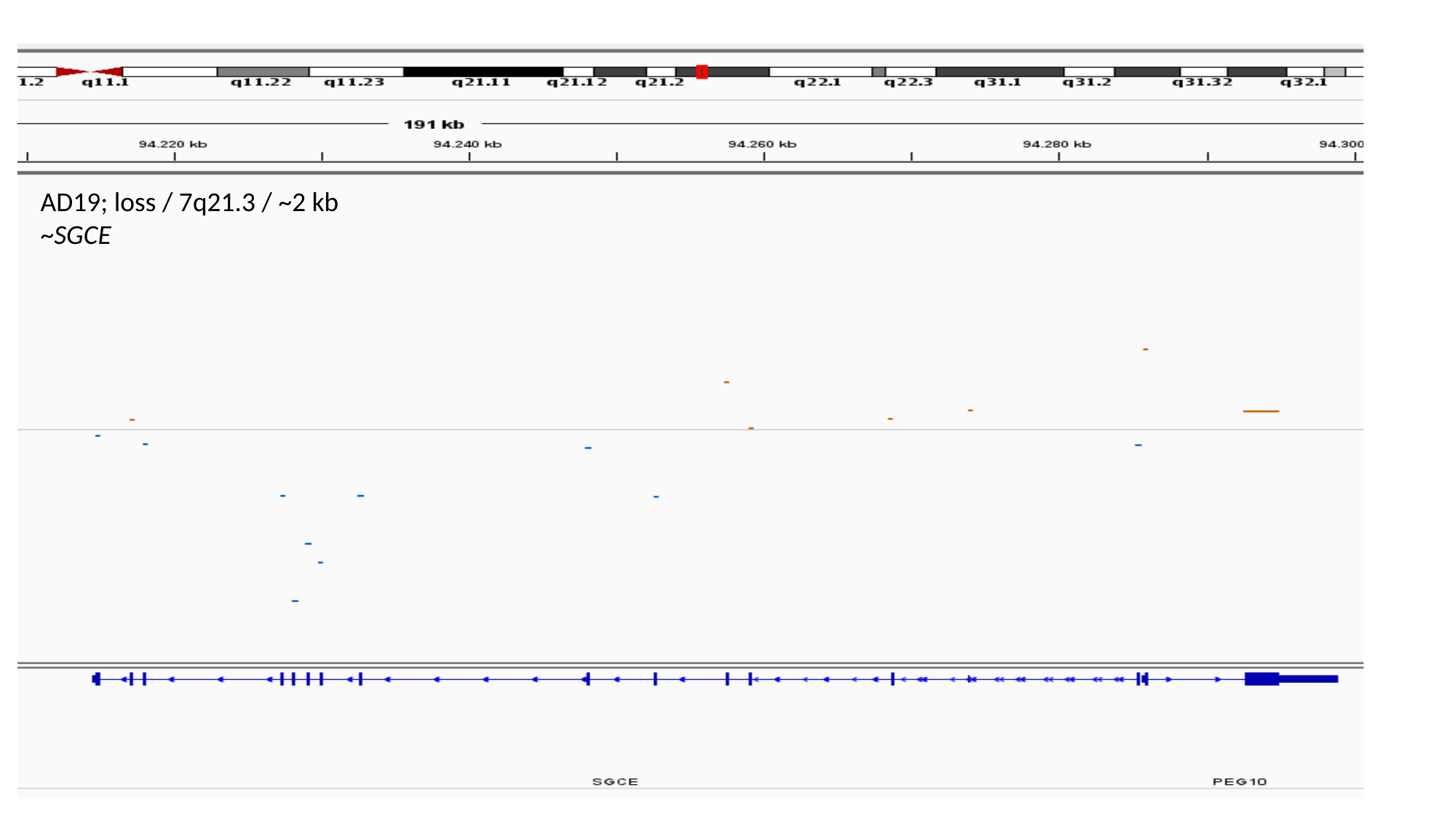

AD19; loss / 7q21.3 / ~2 kb
~SGCE

## Slide 27
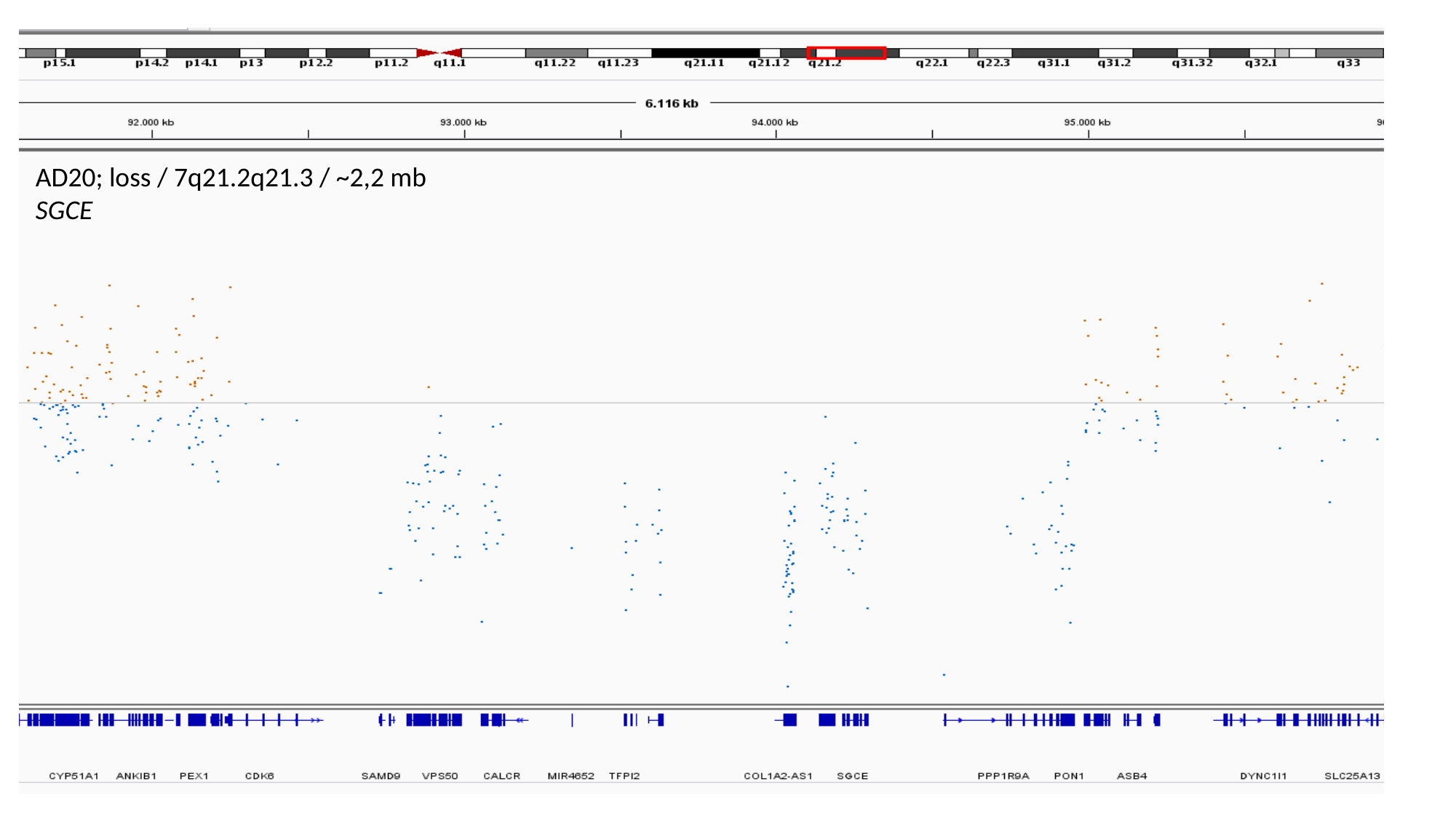

AD20; loss / 7q21.2q21.3 / ~2,2 mb
SGCE

## Slide 28
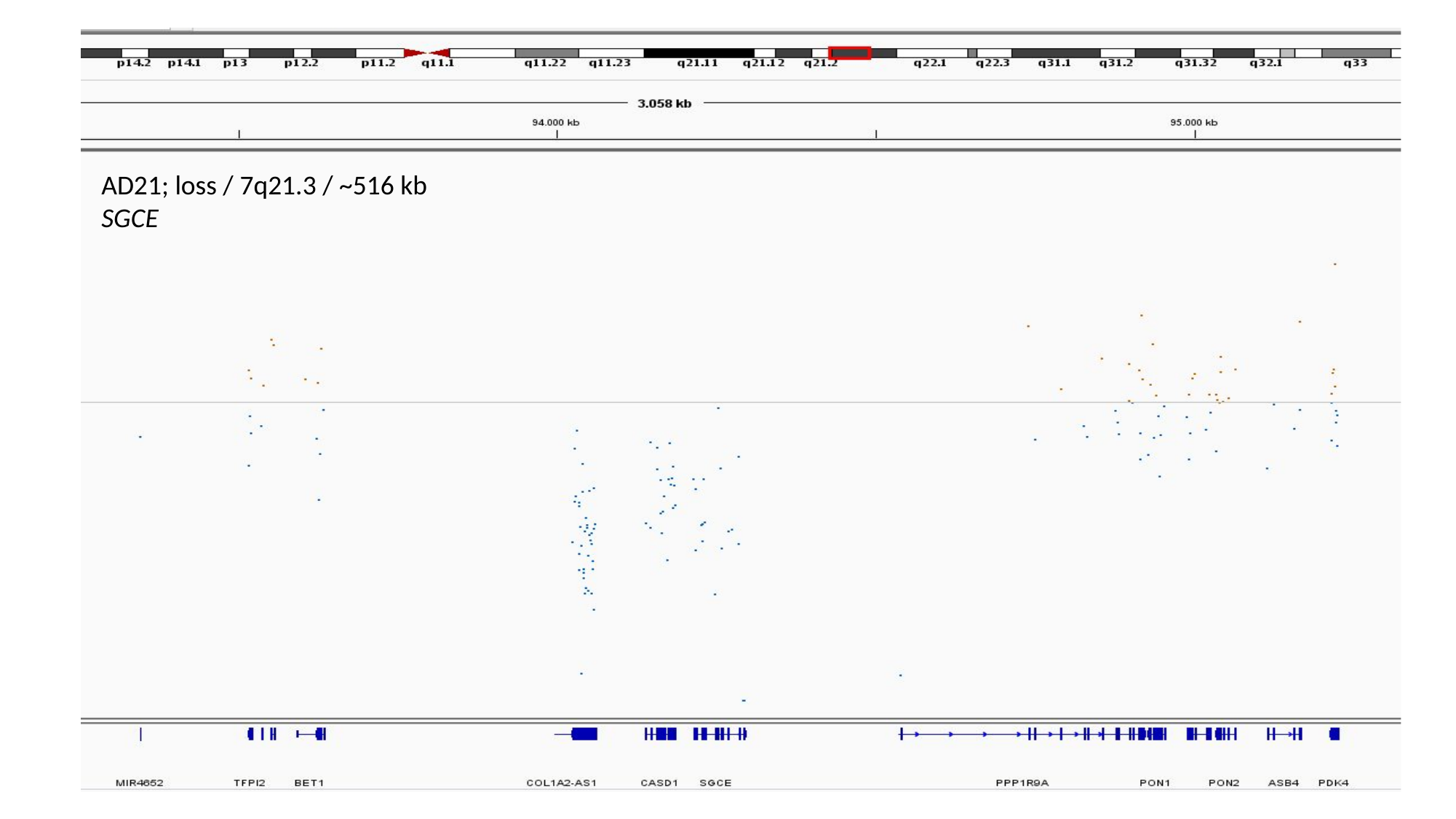

AD21; loss / 7q21.3 / ~516 kb
SGCE

## Slide 29
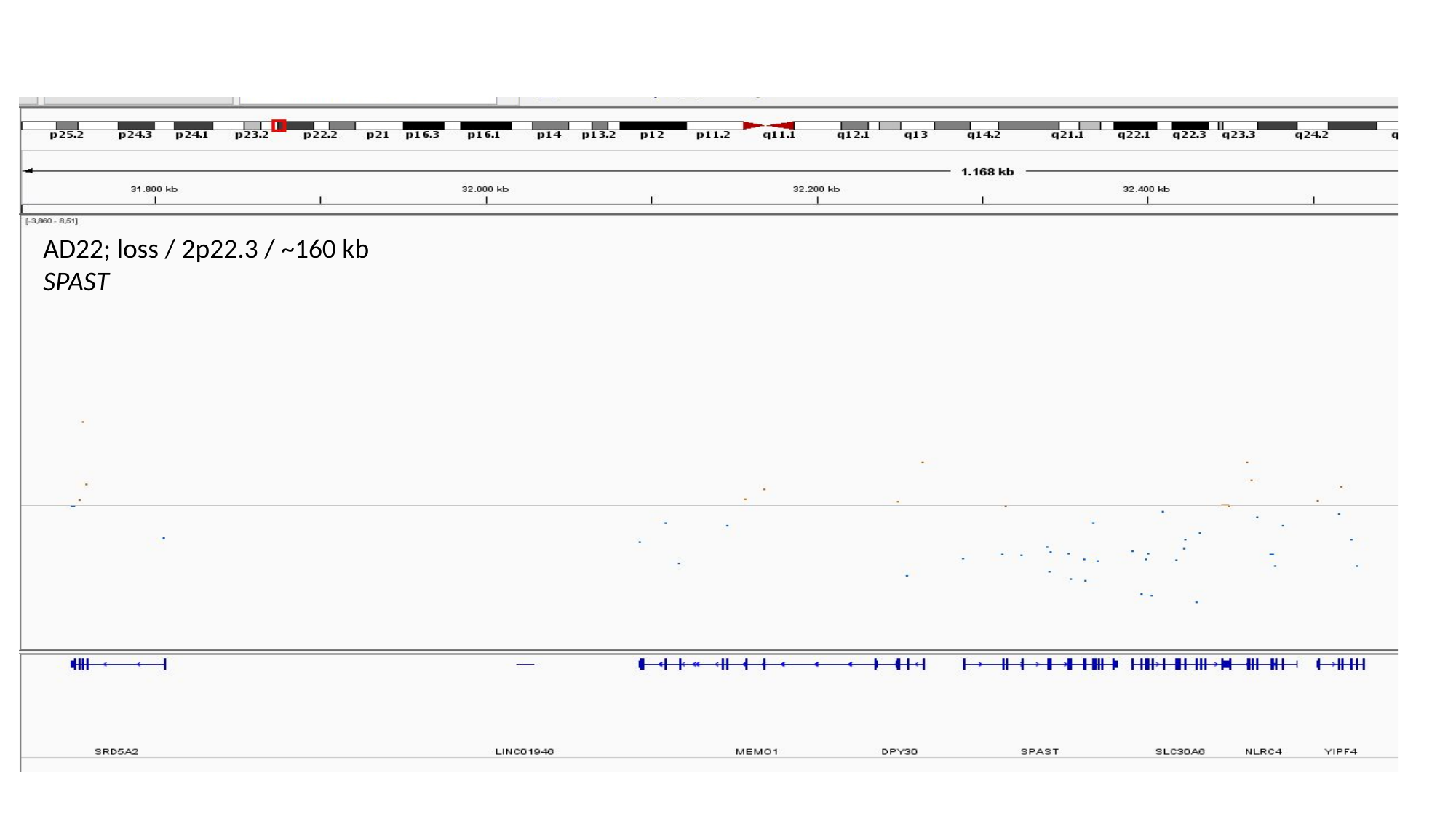

AD22; loss / 2p22.3 / ~160 kb
SPAST

## Slide 30
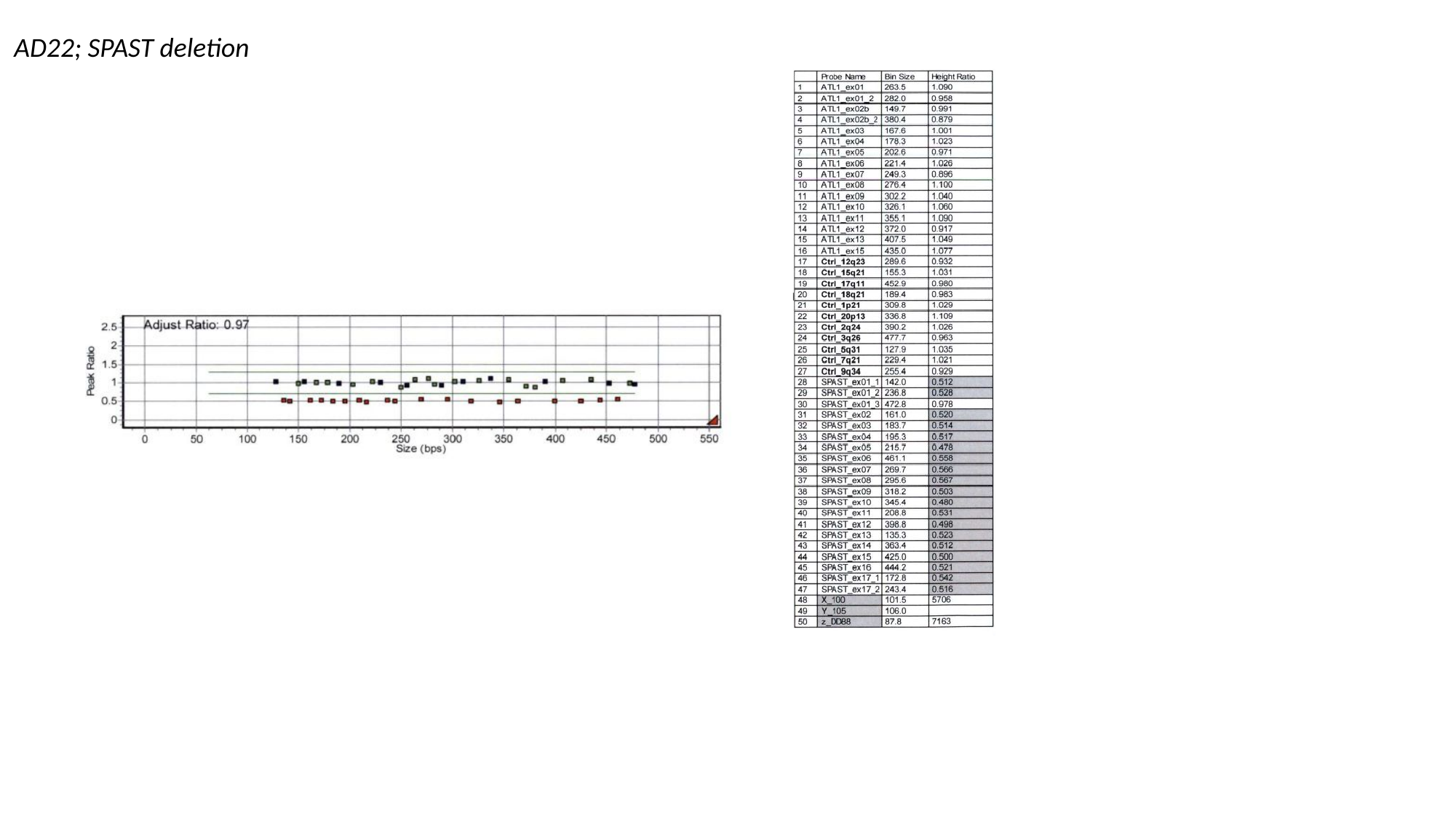

AD22; SPAST deletion

## Slide 31
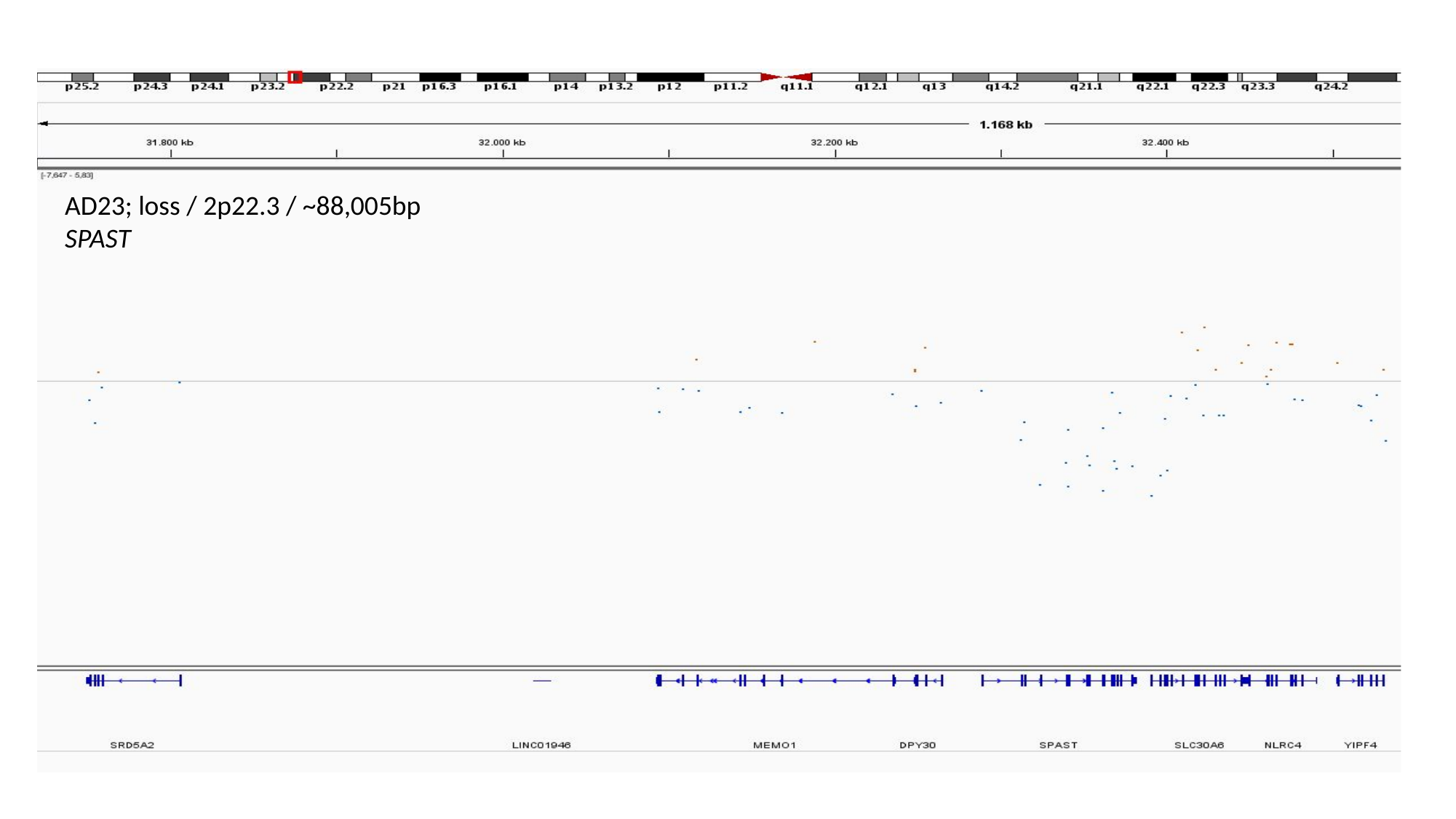

AD23; loss / 2p22.3 / ~88,005bp
SPAST

## Slide 32
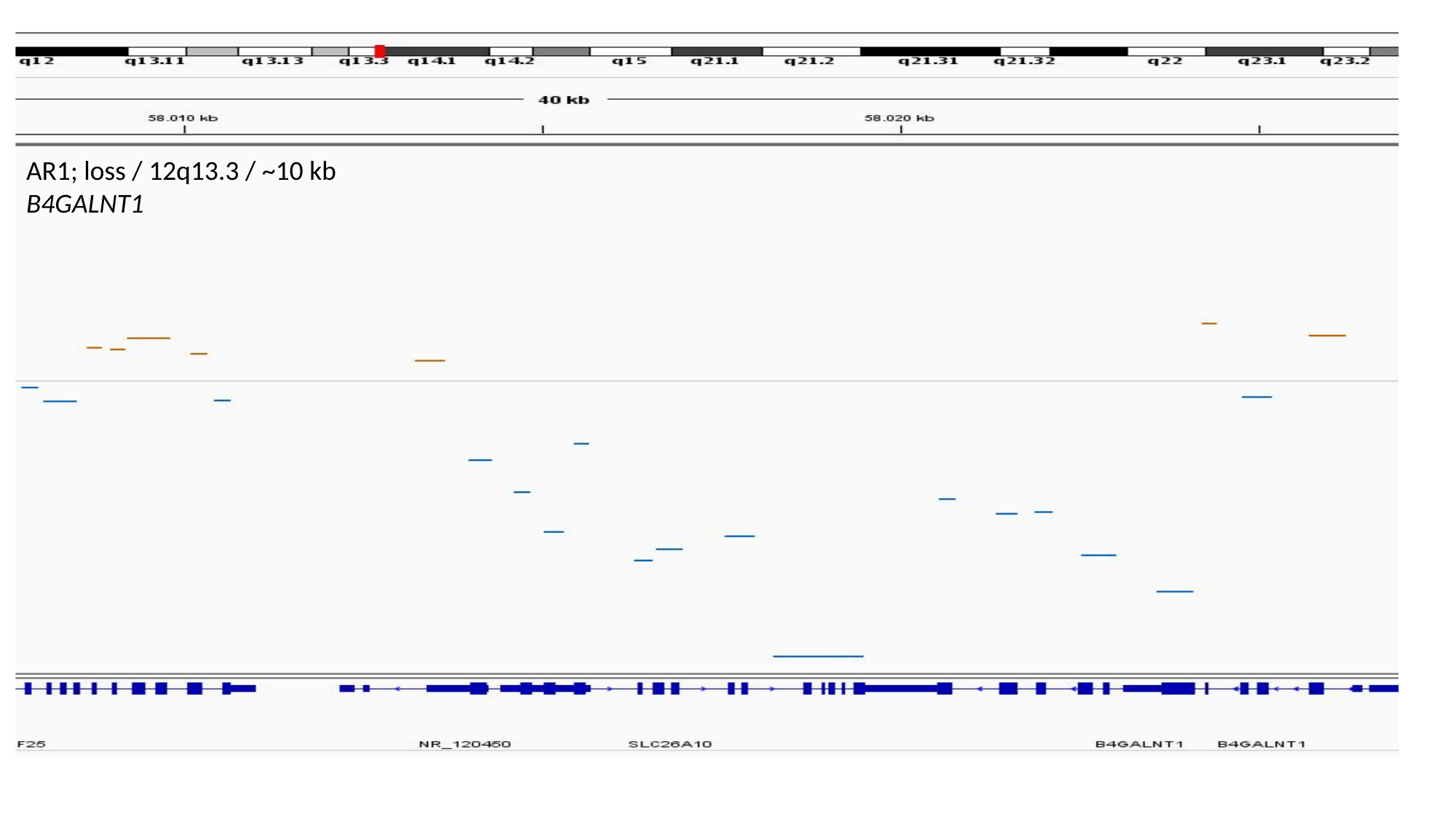

AR1; loss / 12q13.3 / ~10 kb
B4GALNT1

## Slide 33
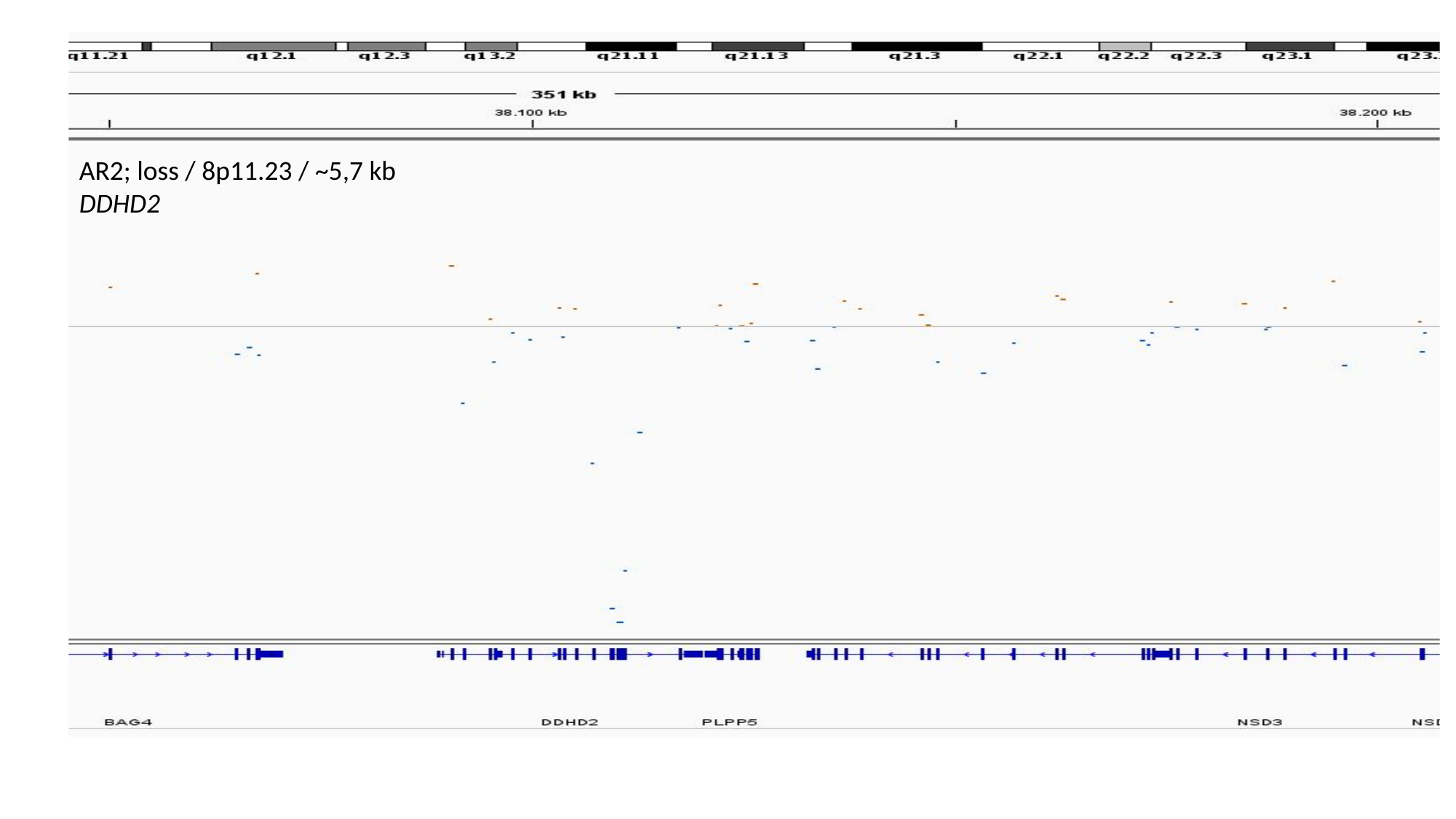

AR2; loss / 8p11.23 / ~5,7 kb
DDHD2

## Slide 34
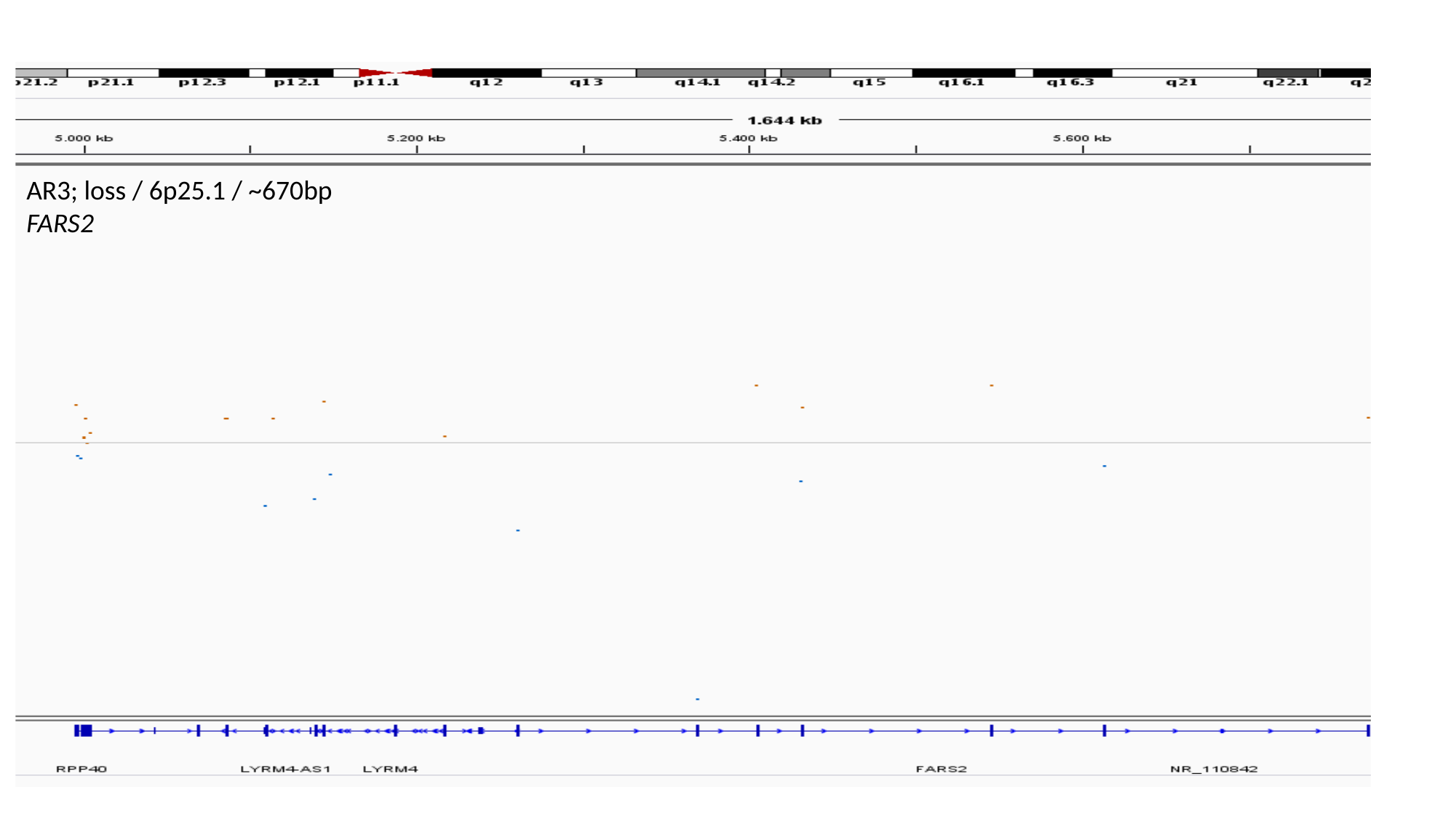

AR3; loss / 6p25.1 / ~670bp
FARS2

## Slide 35
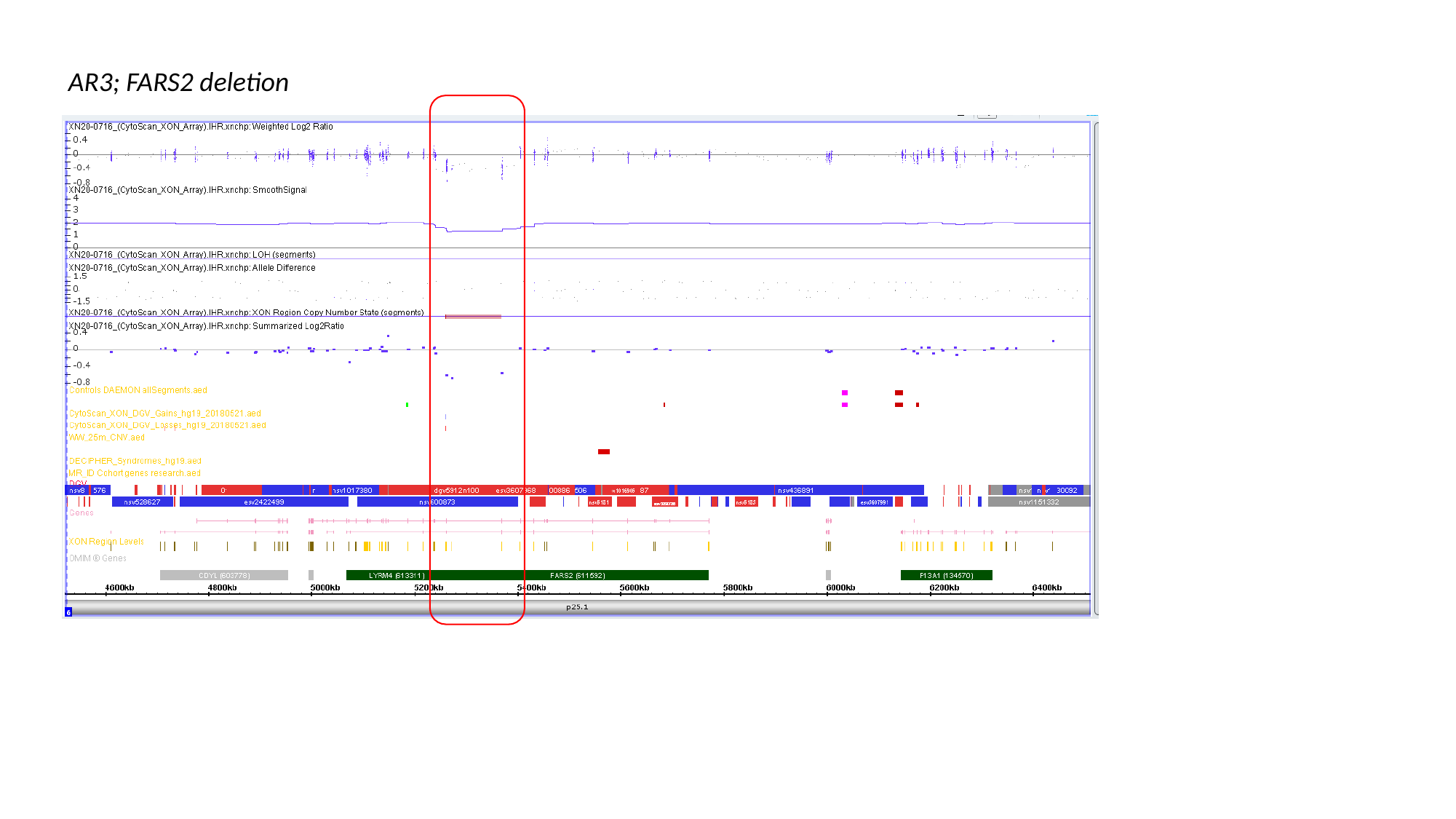

AR3; FARS2 deletion

## Slide 36
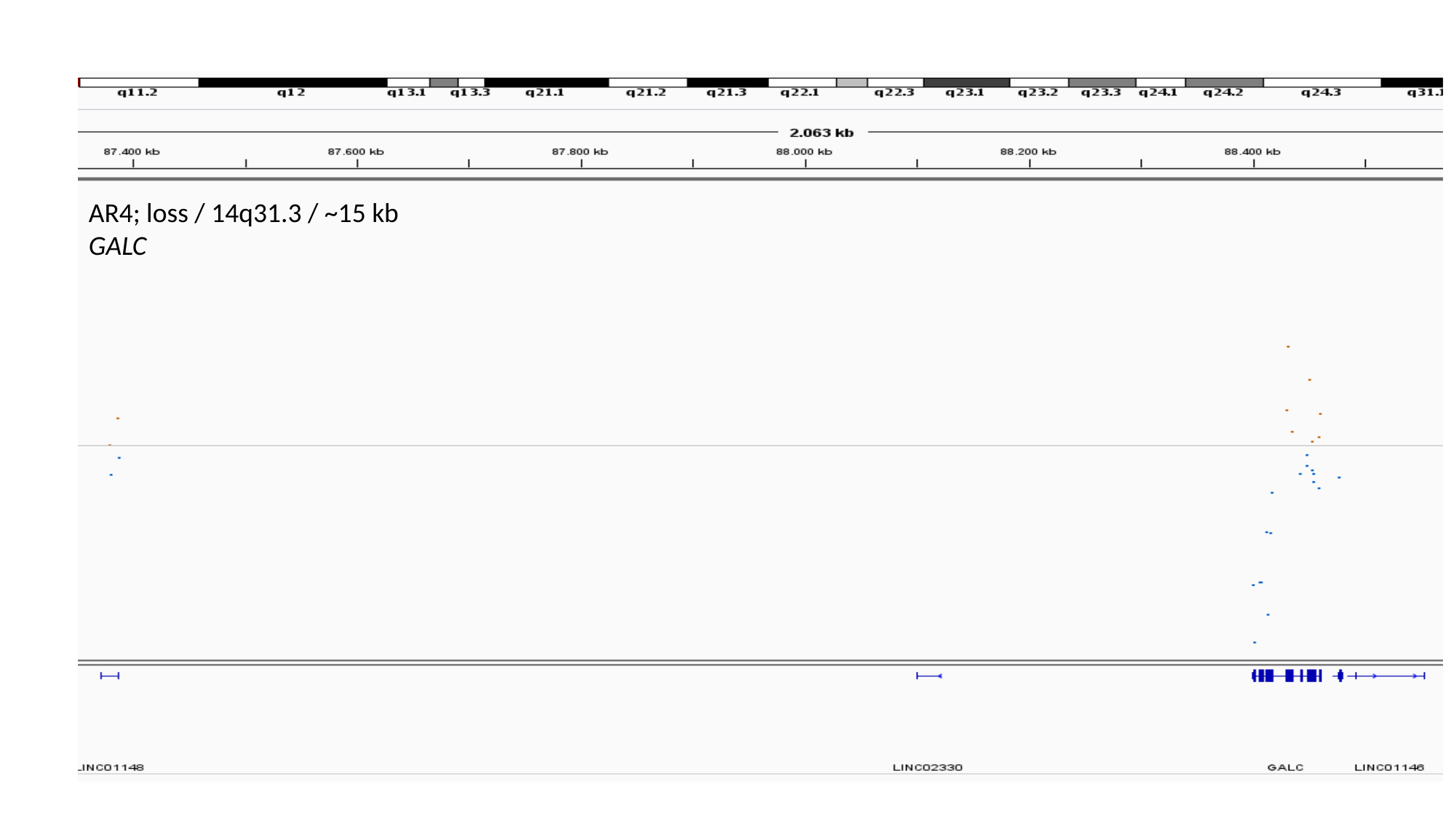

AR4; loss / 14q31.3 / ~15 kb
GALC

## Slide 37
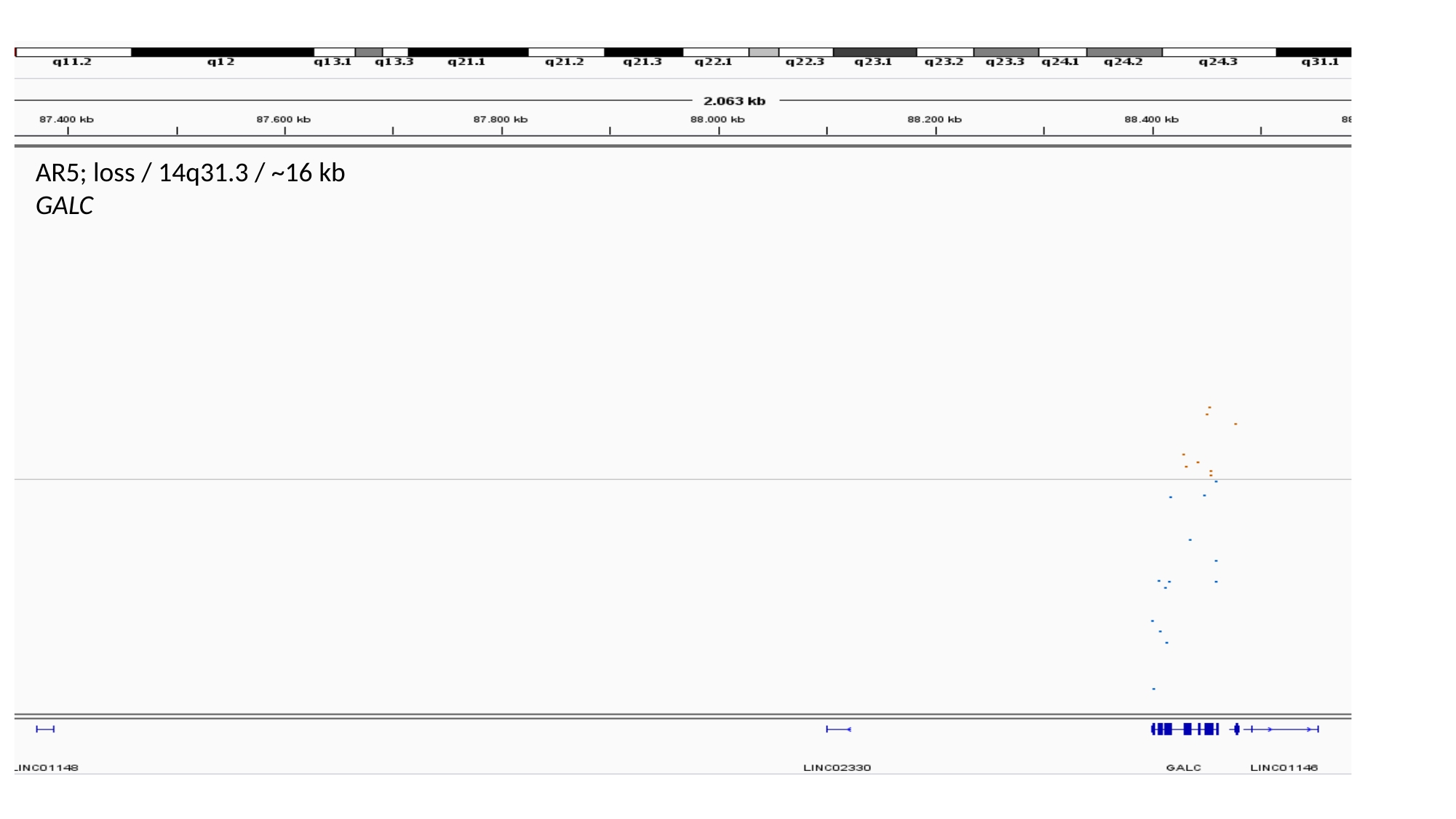

AR5; loss / 14q31.3 / ~16 kb
GALC

## Slide 38
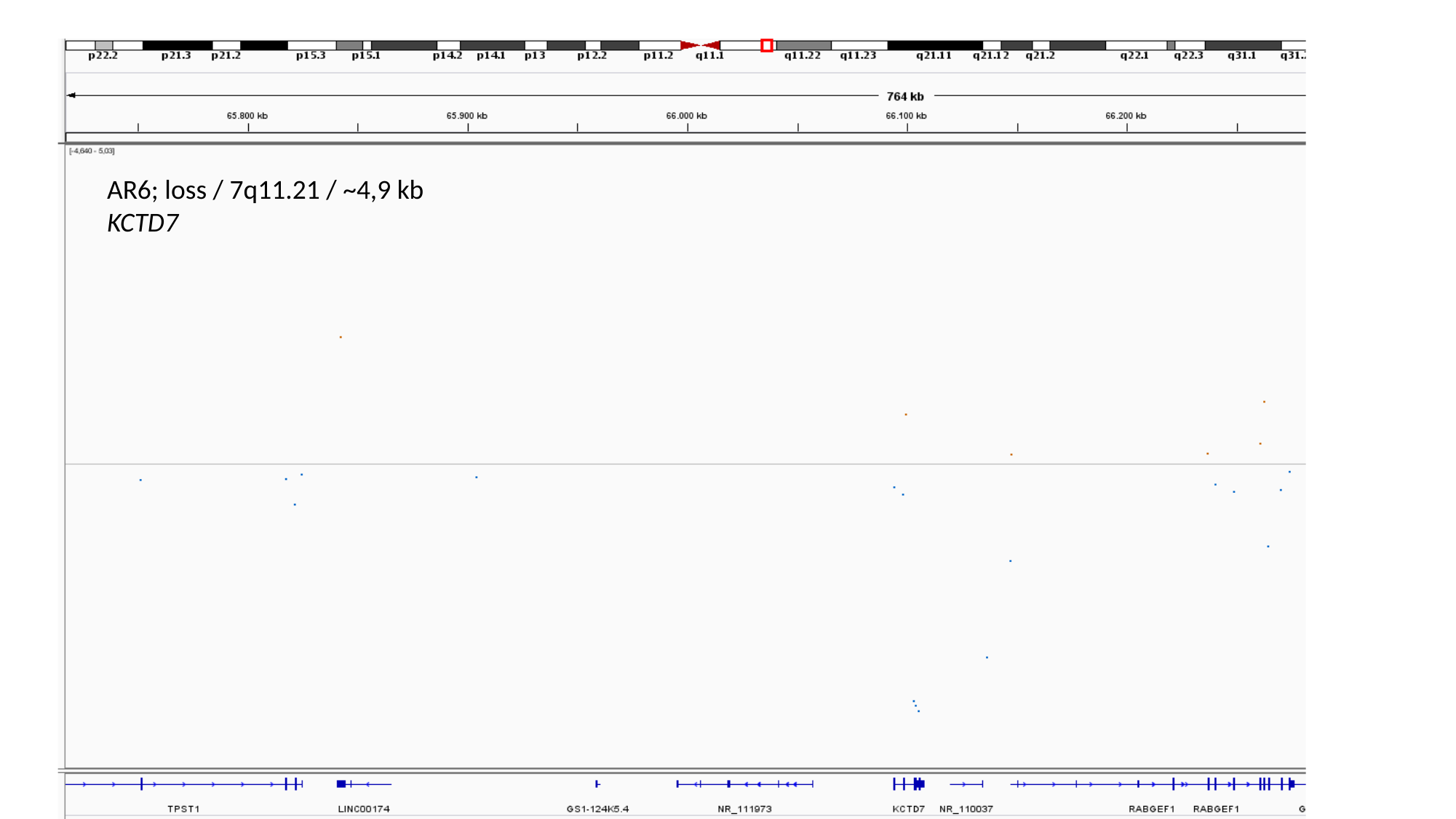

AR6; loss / 7q11.21 / ~4,9 kb
KCTD7

## Slide 39
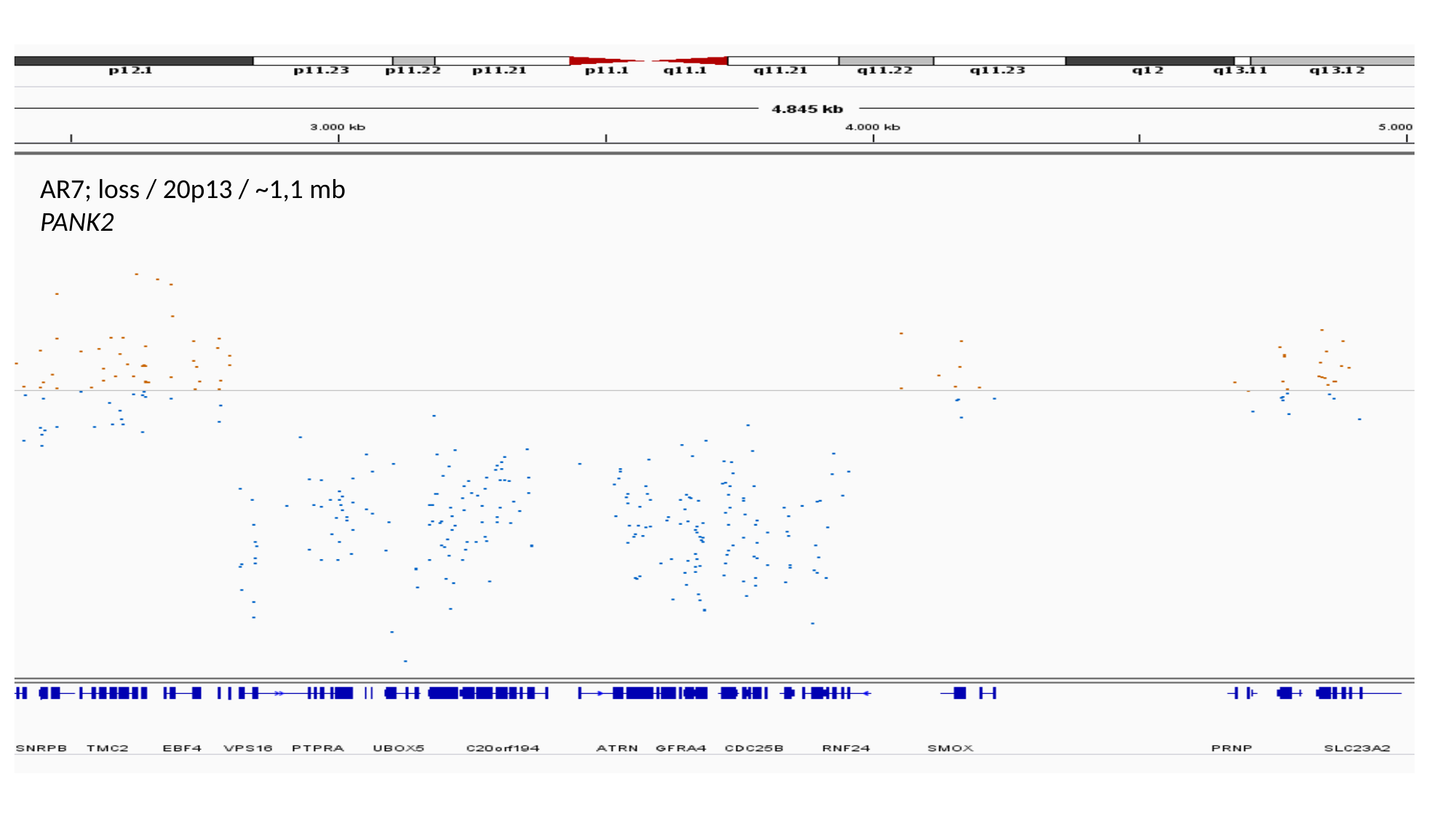

AR7; loss / 20p13 / ~1,1 mb
PANK2

## Slide 40
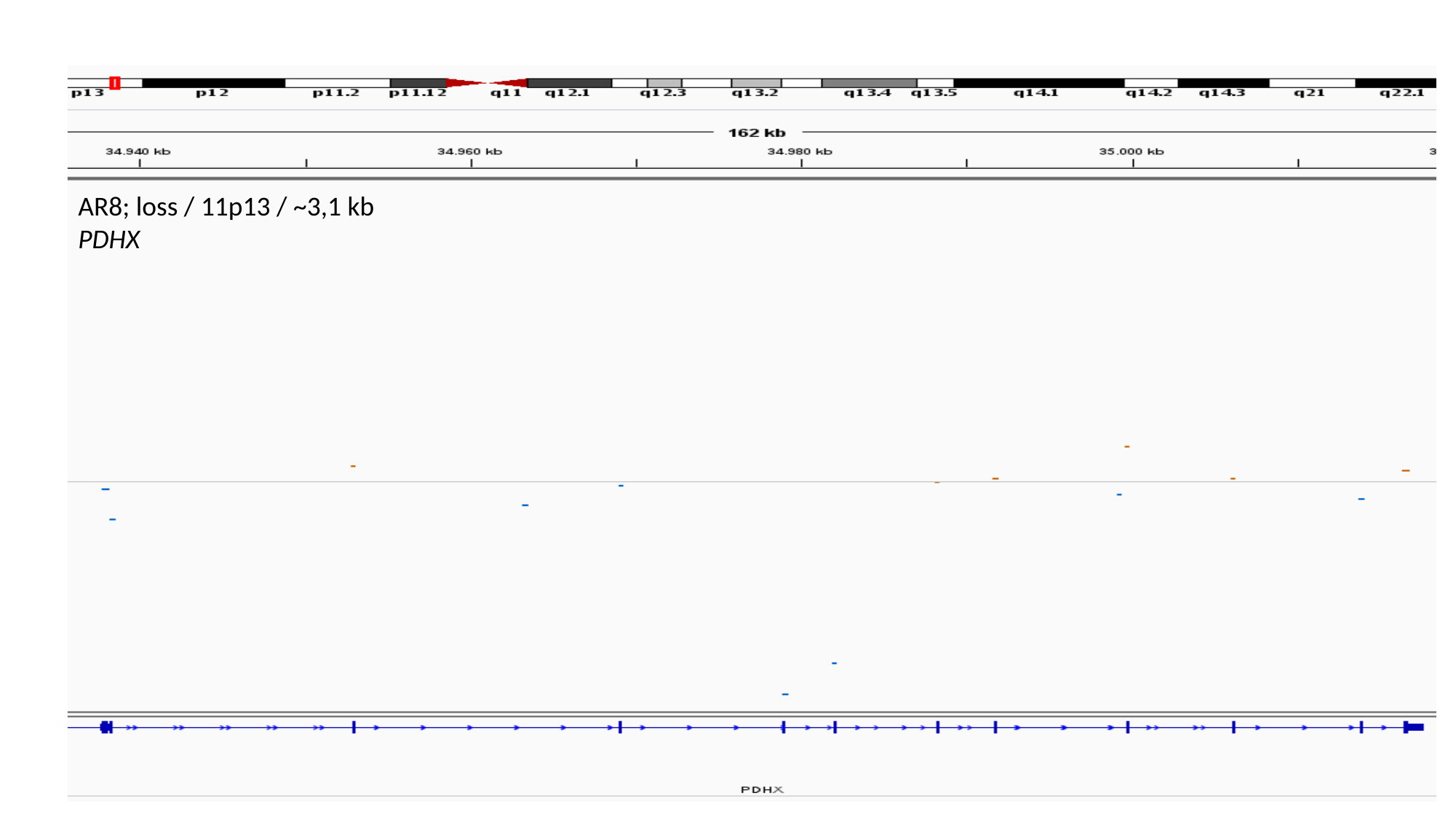

AR8; loss / 11p13 / ~3,1 kb
PDHX

## Slide 41
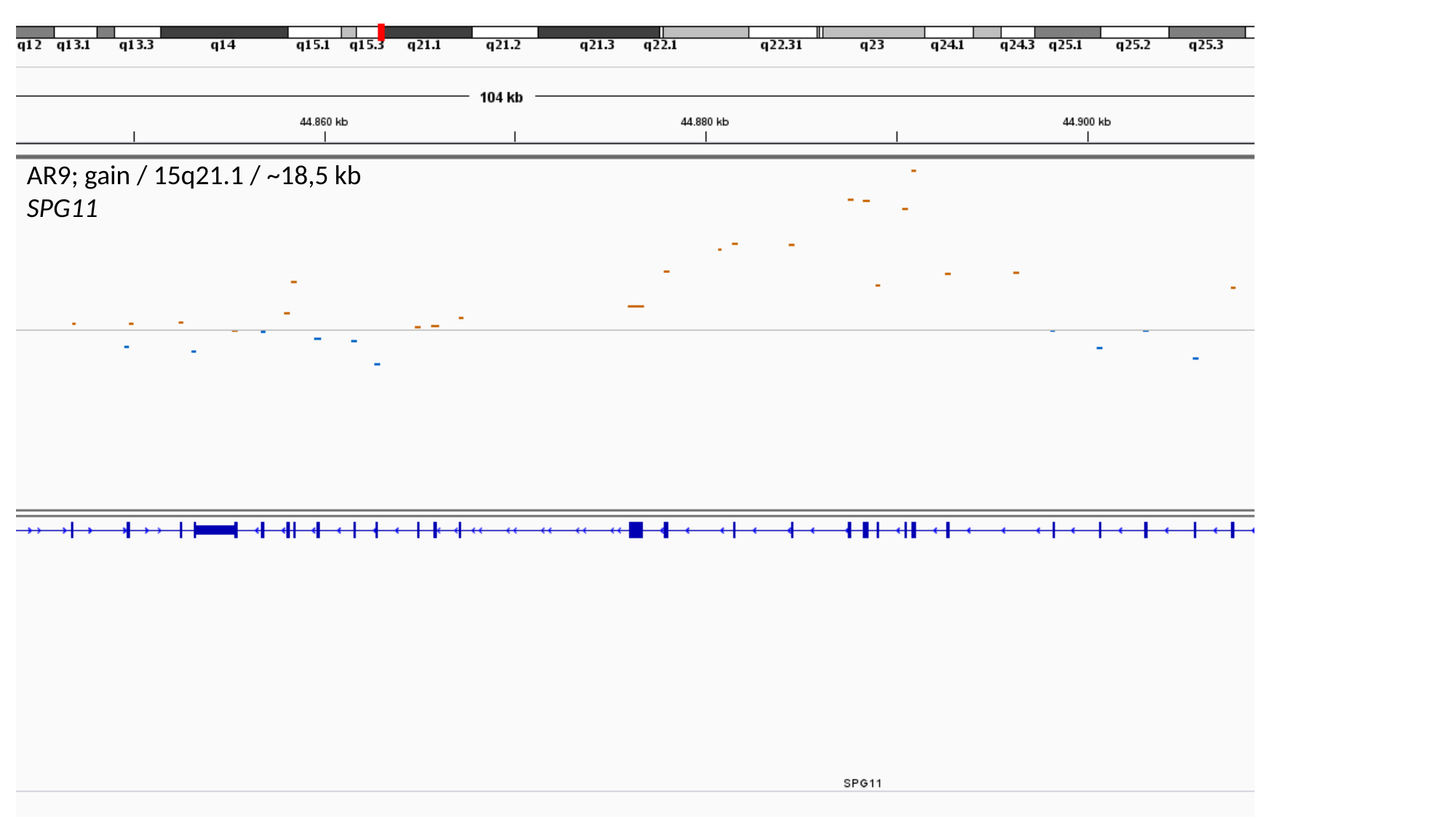

AR9; gain / 15q21.1 / ~18,5 kb
SPG11

## Slide 42
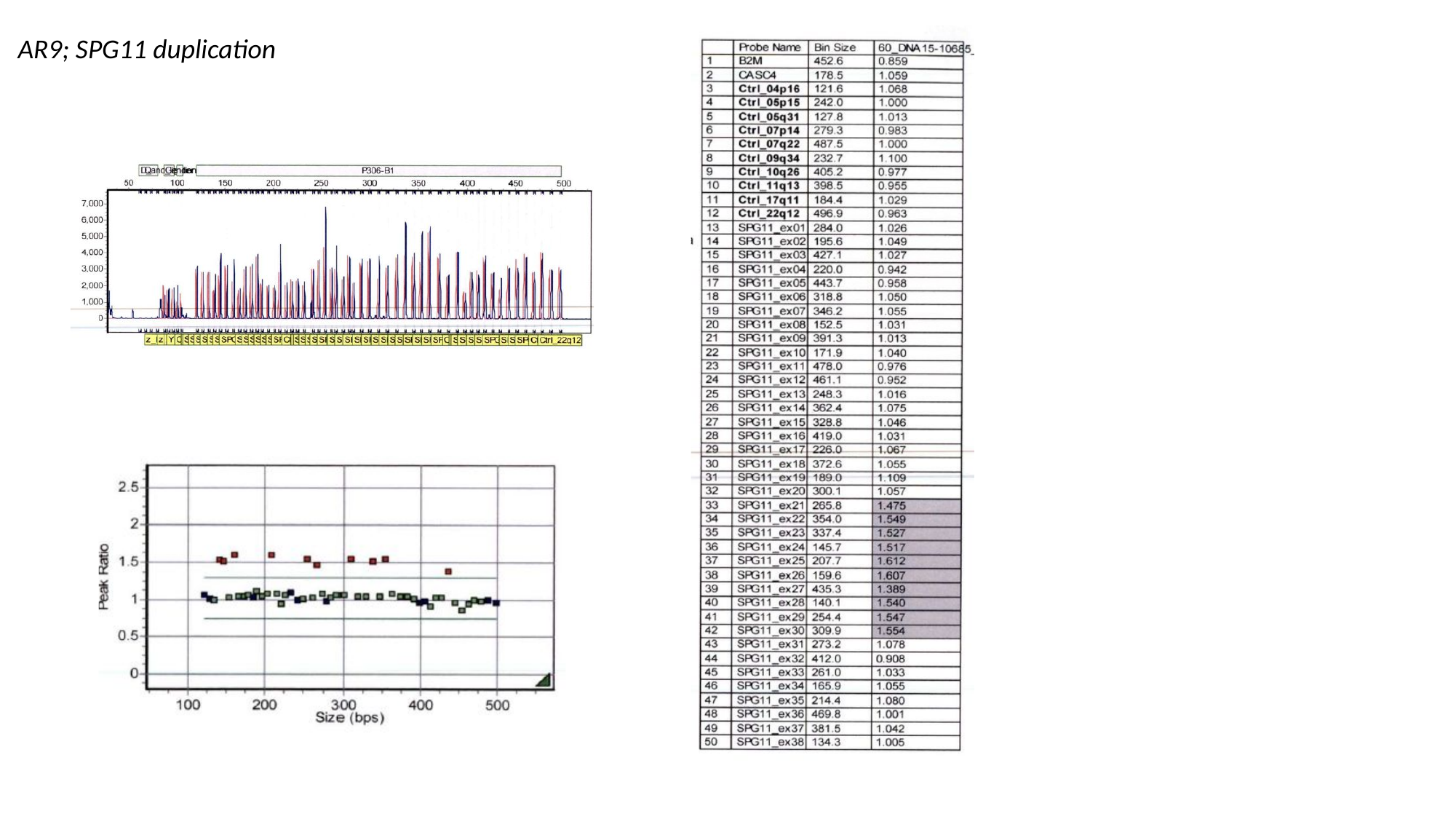

AR9; SPG11 duplication

## Slide 43
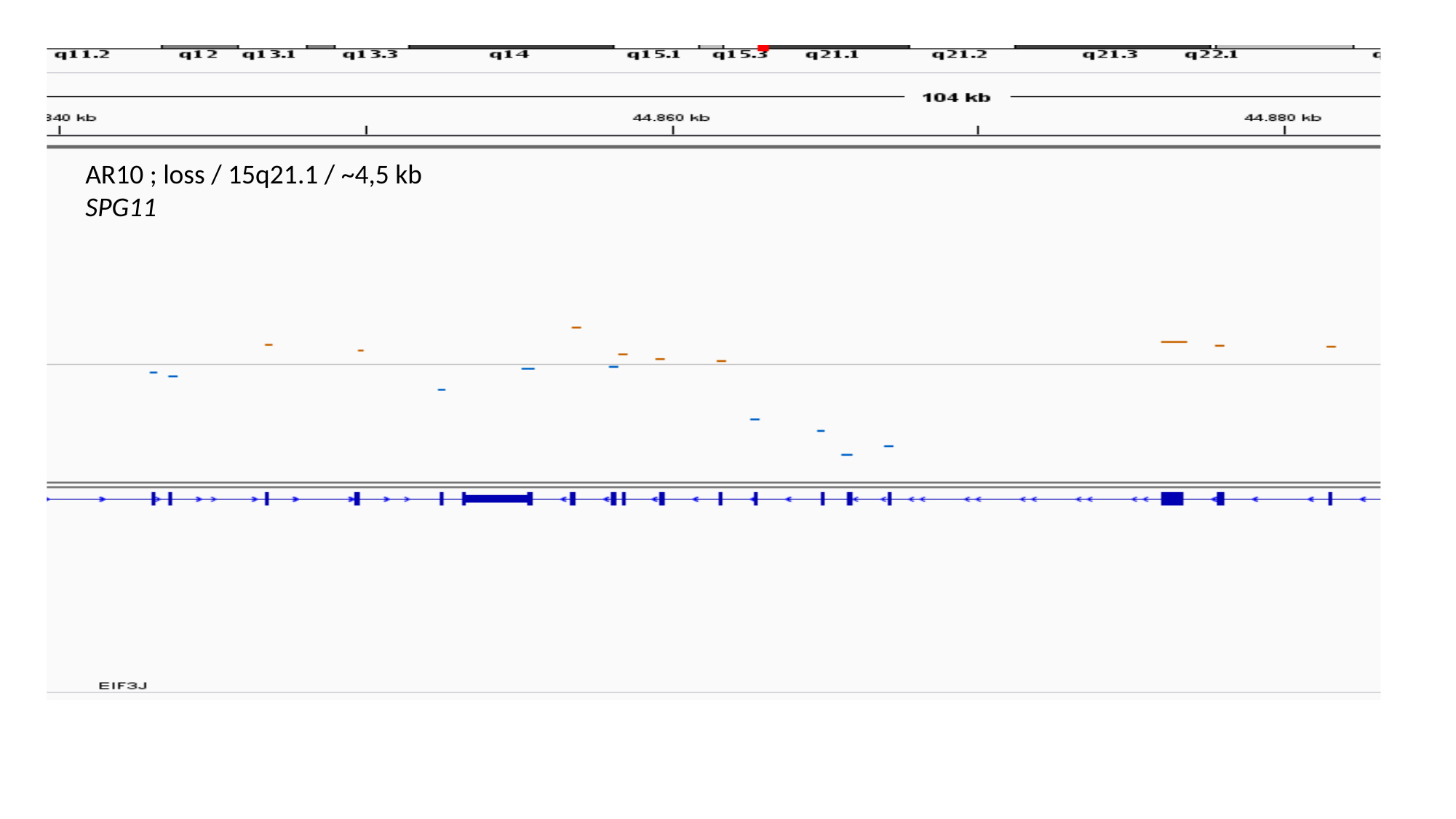

AR10 ; loss / 15q21.1 / ~4,5 kb
SPG11

## Slide 44
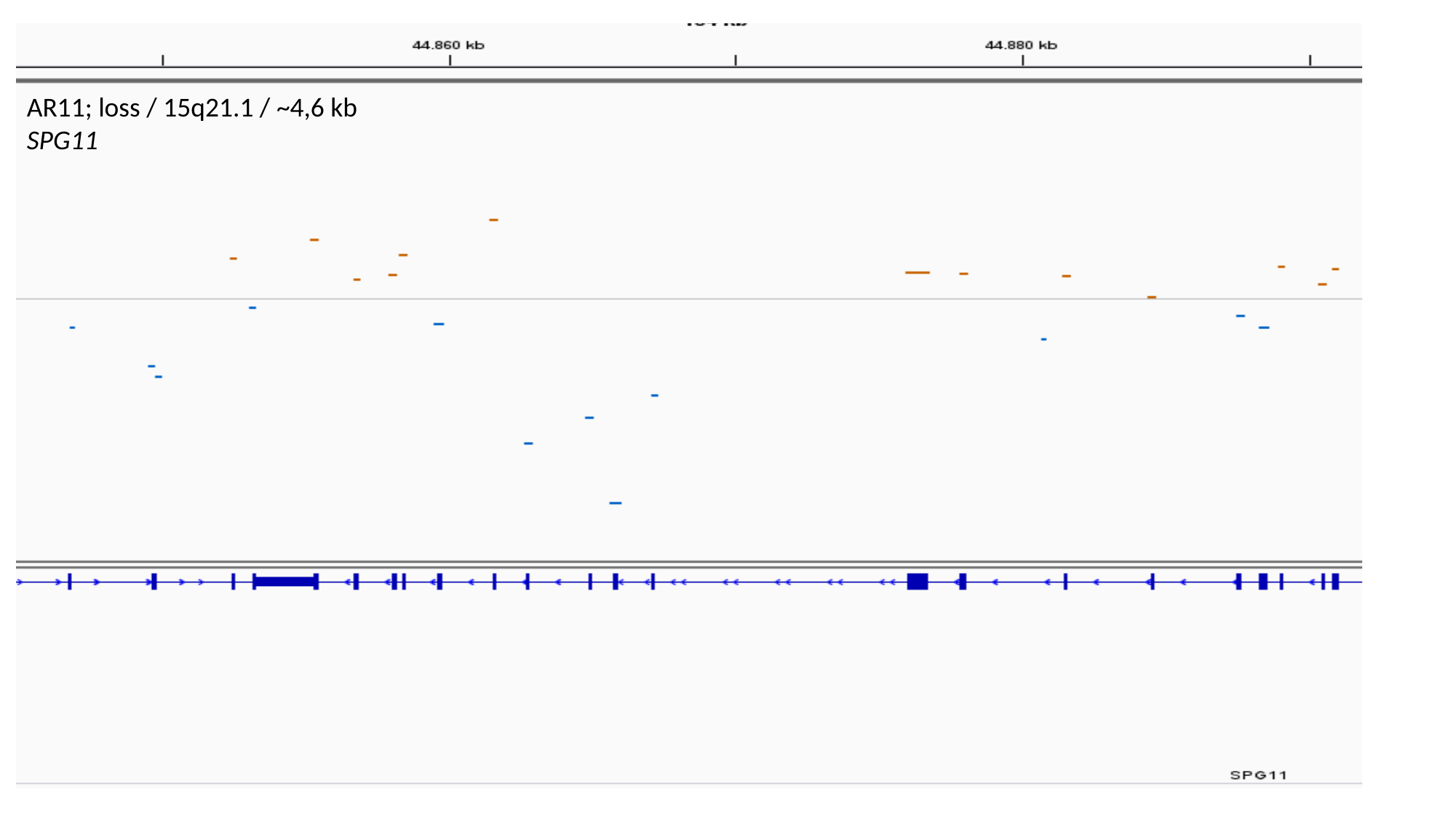

AR11; loss / 15q21.1 / ~4,6 kb
SPG11

## Slide 45
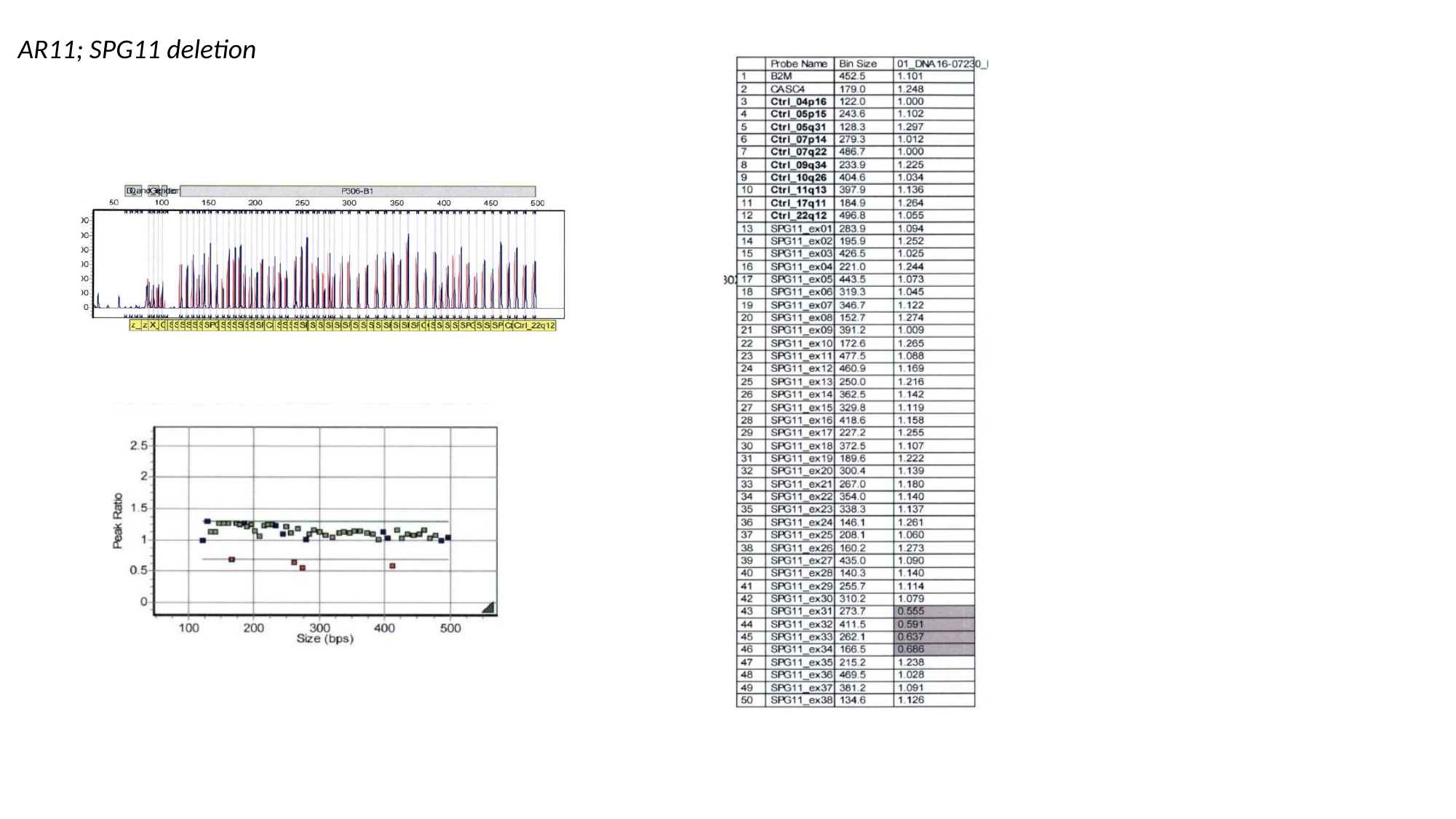

AR11; SPG11 deletion

## Slide 46
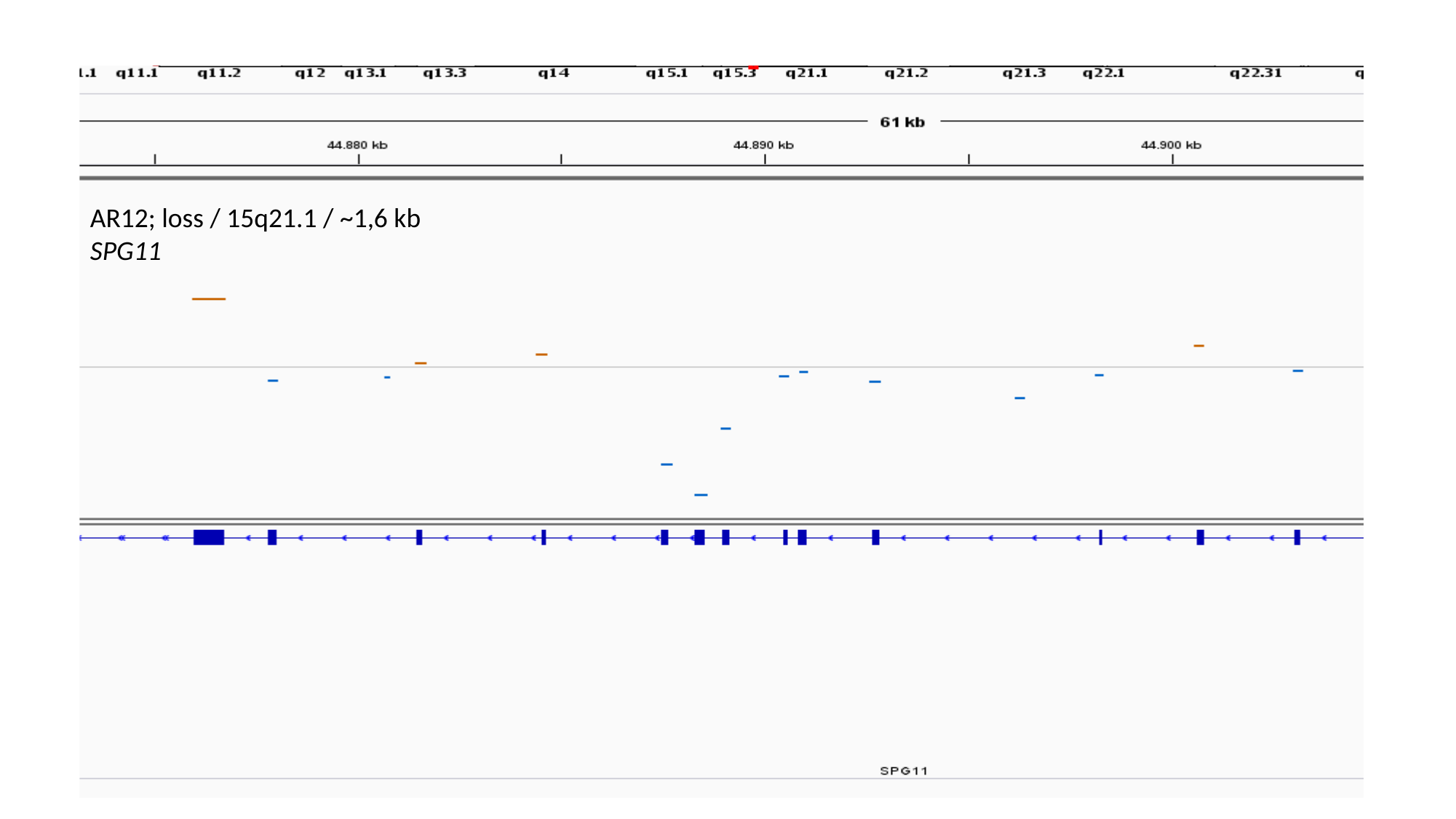

AR12; loss / 15q21.1 / ~1,6 kb
SPG11

## Slide 47
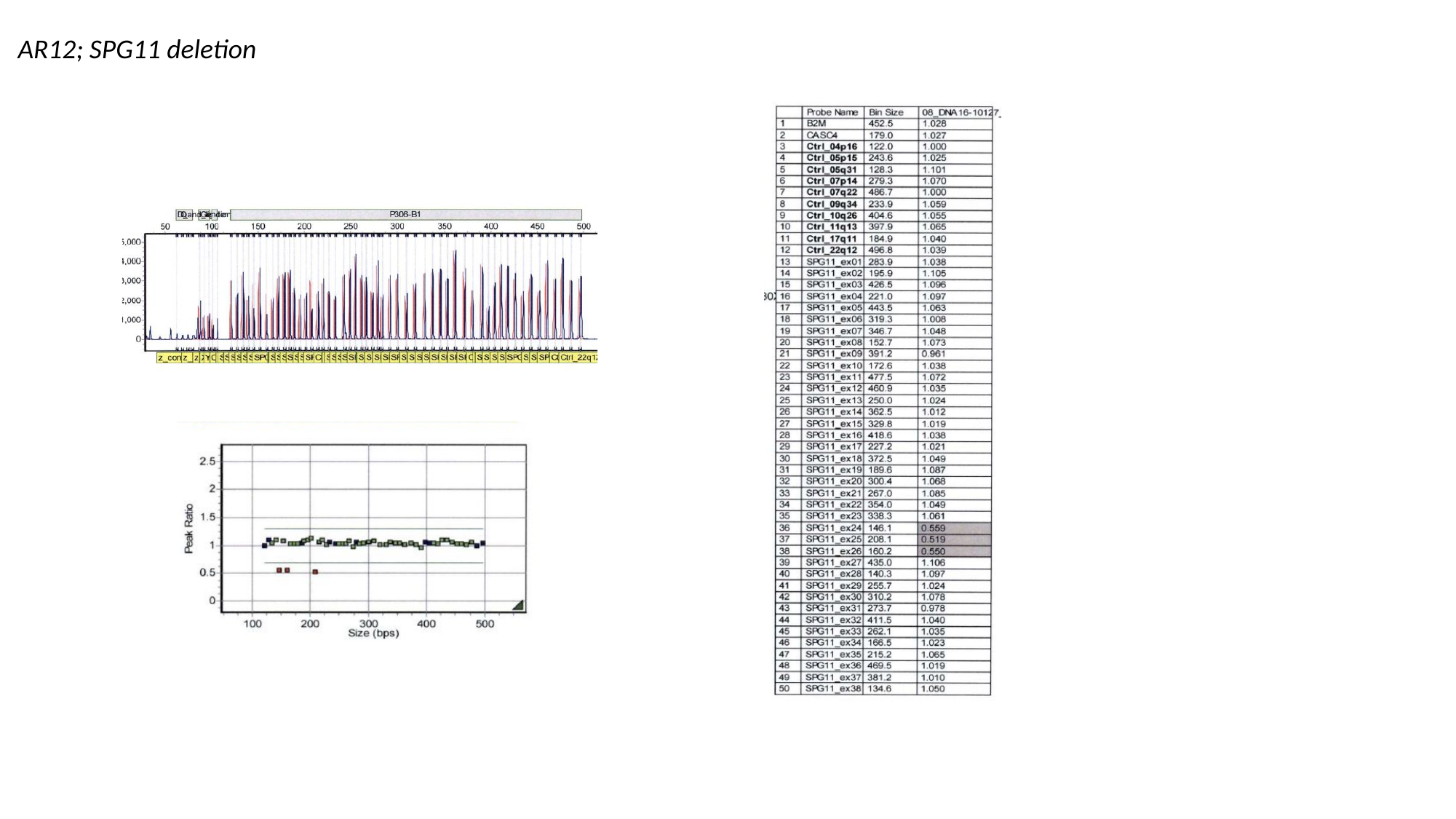

AR12; SPG11 deletion

## Slide 48
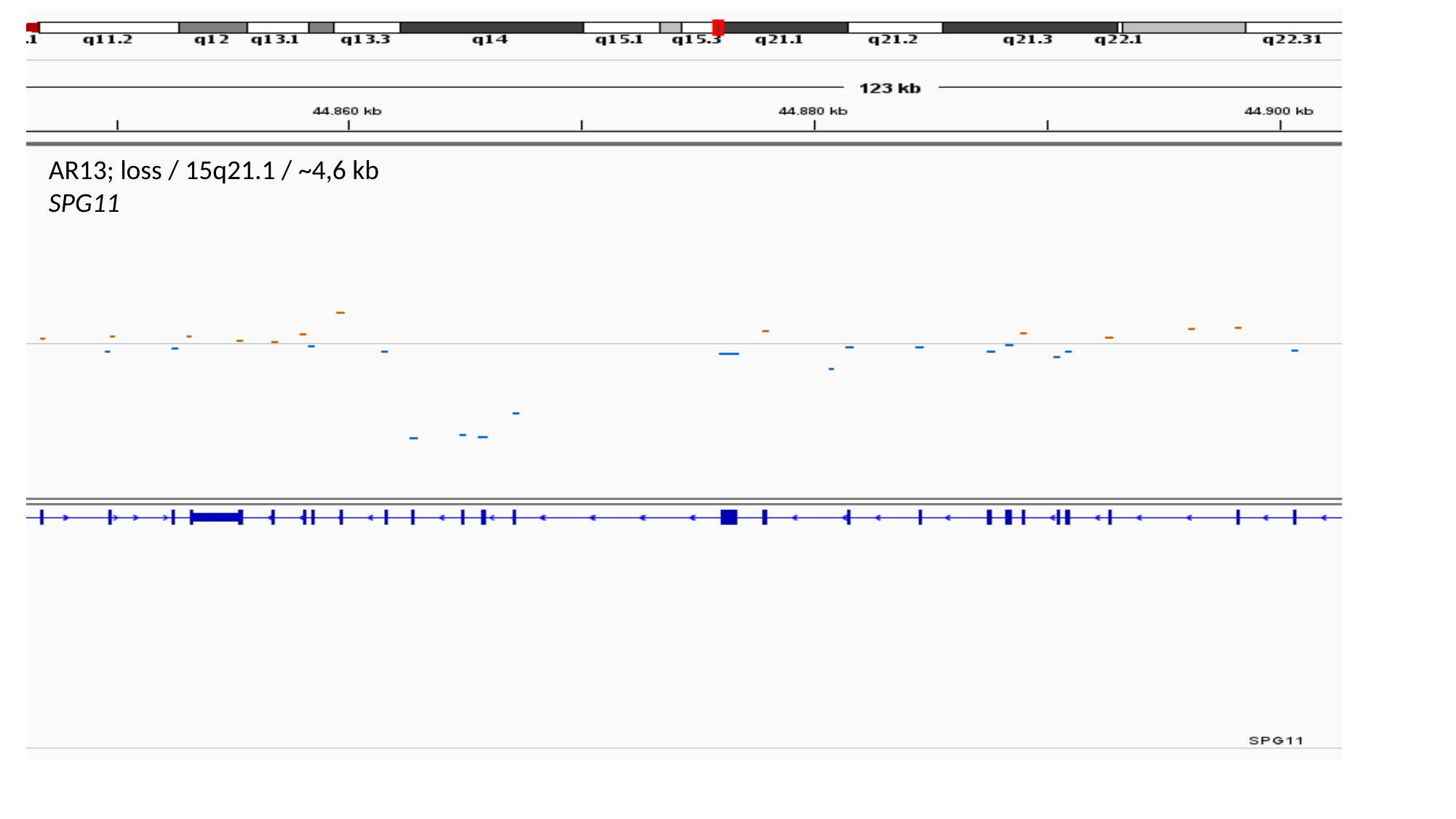

AR13; loss / 15q21.1 / ~4,6 kb
SPG11

## Slide 49
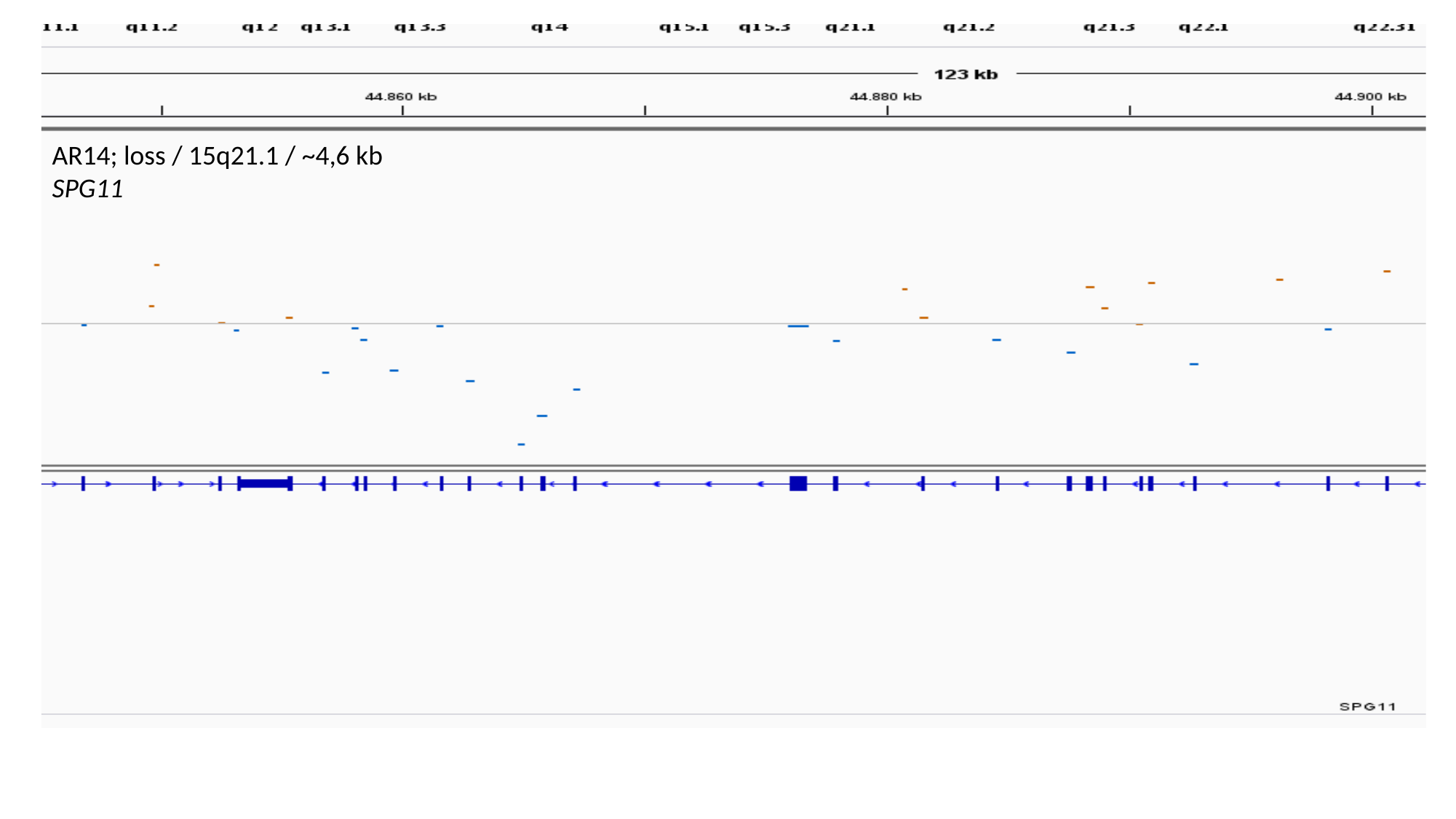

AR14; loss / 15q21.1 / ~4,6 kb
SPG11

## Slide 50
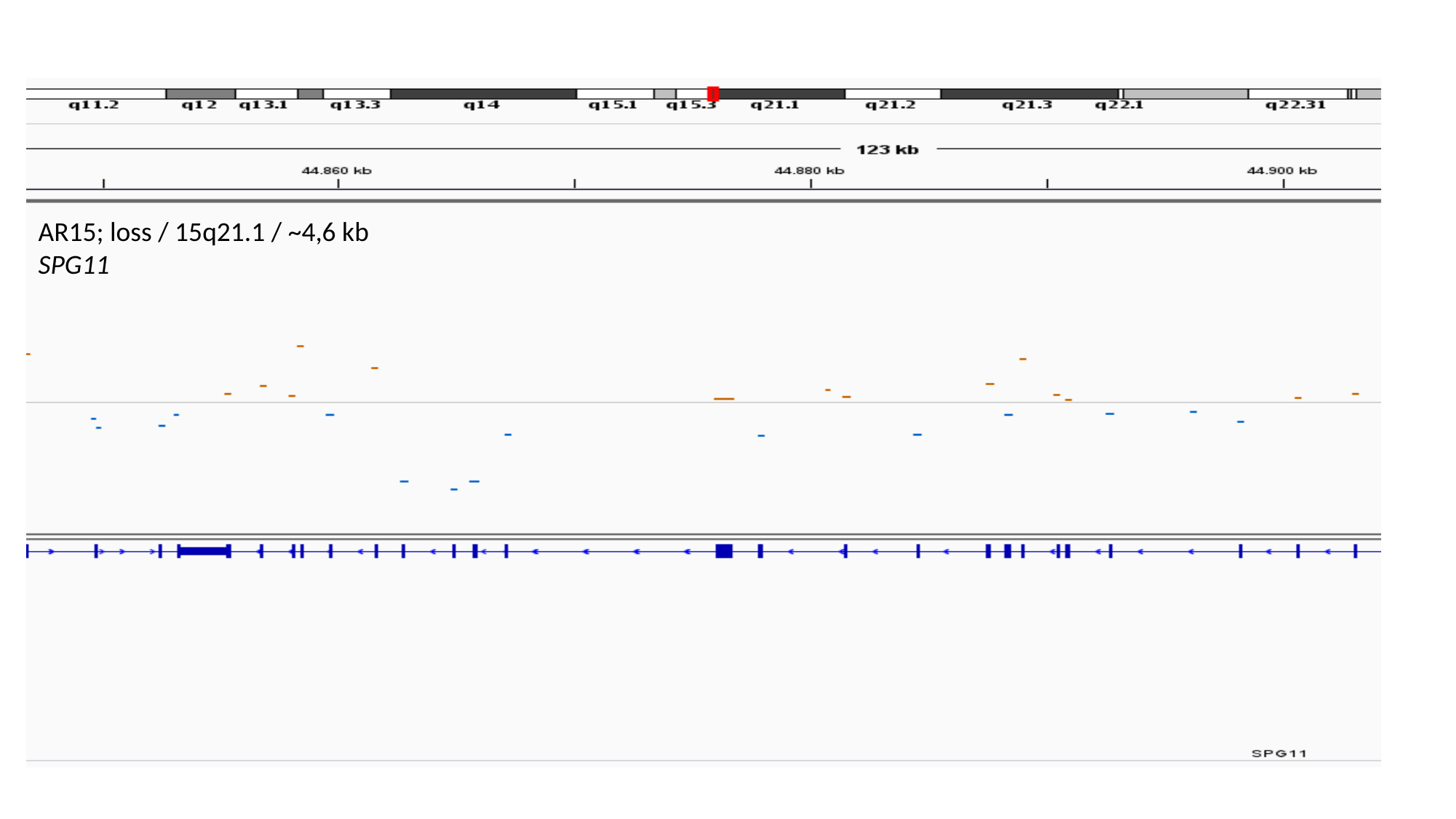

AR15; loss / 15q21.1 / ~4,6 kb SPG11

## Slide 51
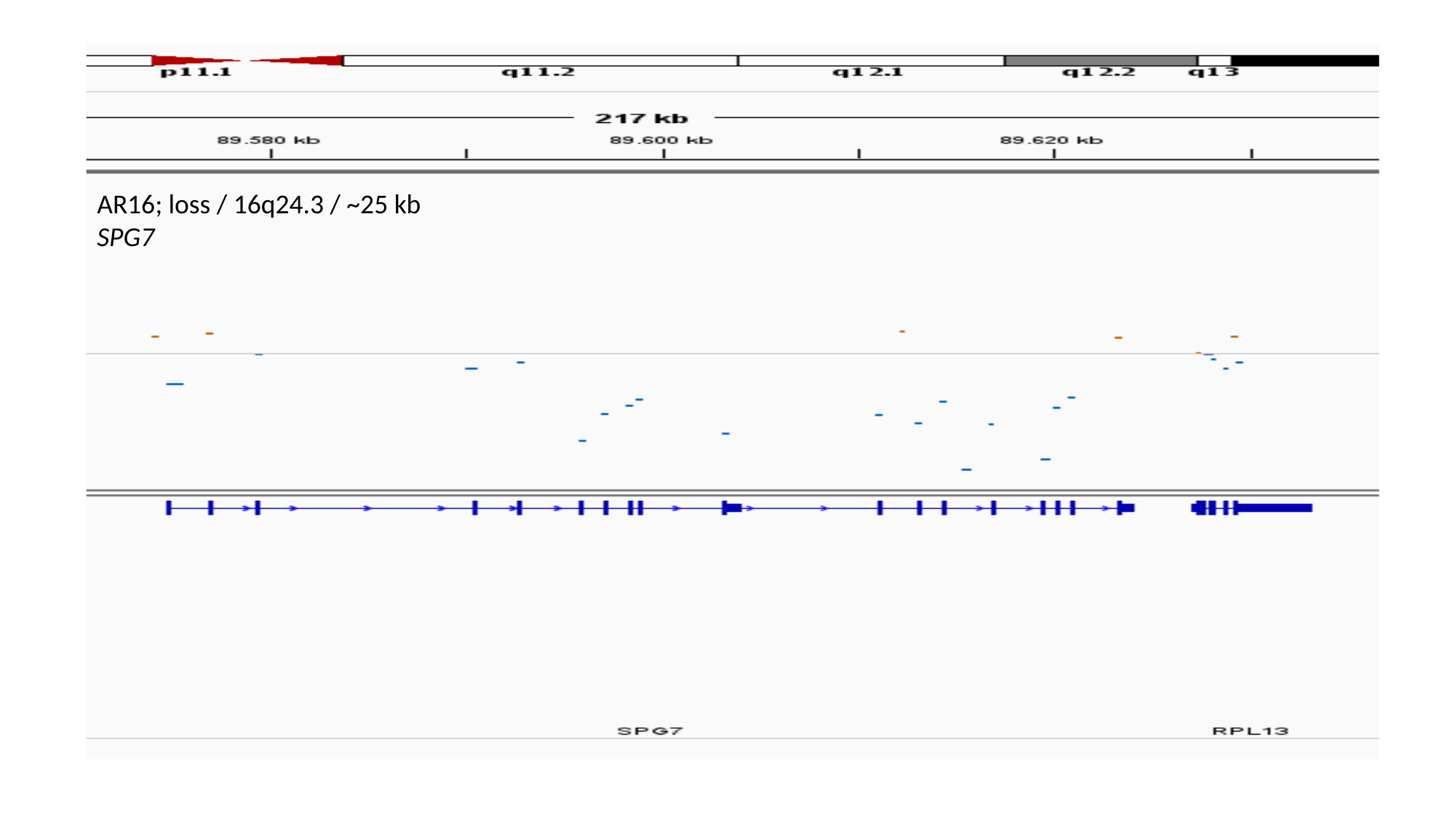

AR16; loss / 16q24.3 / ~25 kb
SPG7

## Slide 52
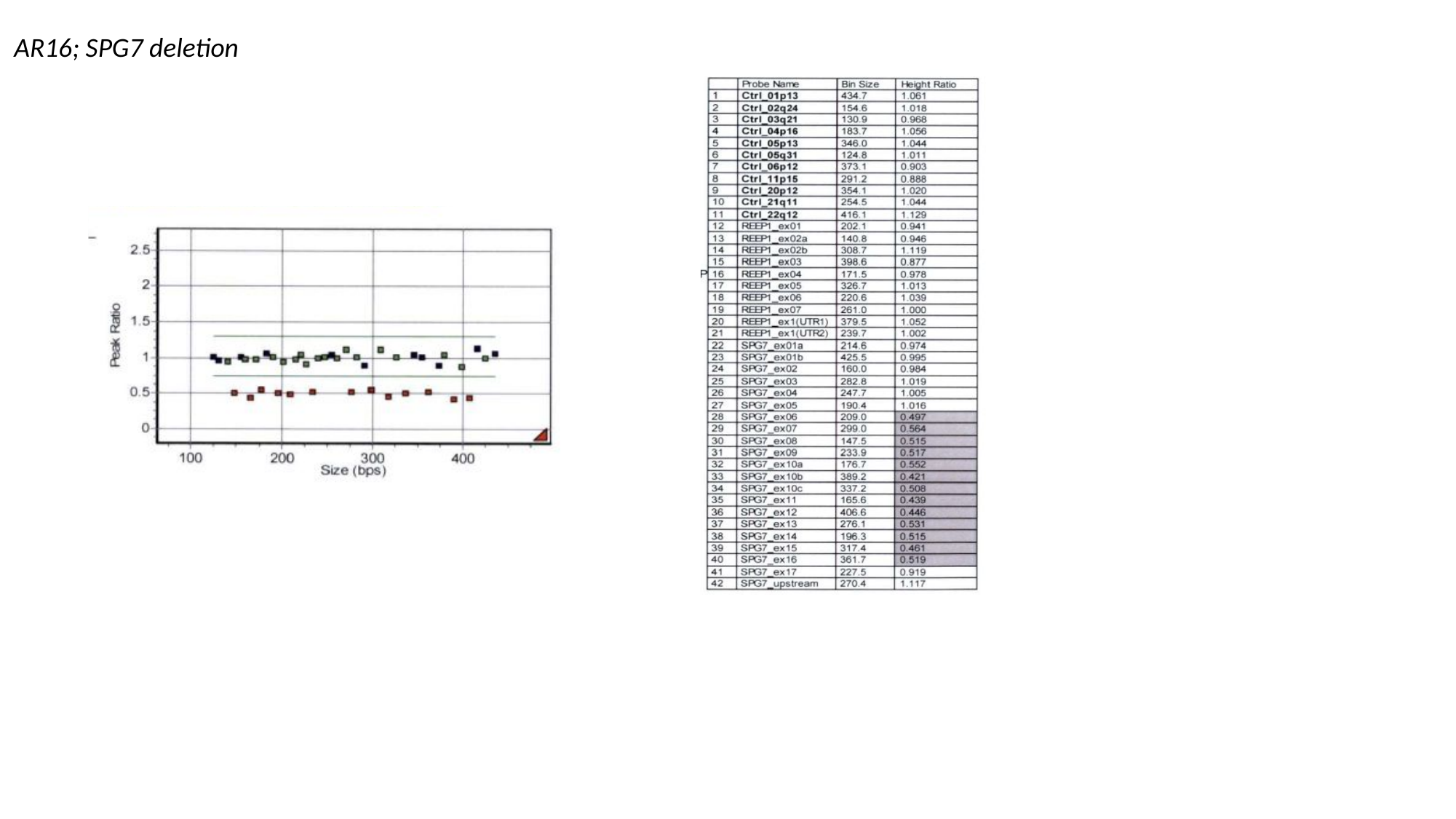

AR16; SPG7 deletion

## Slide 53
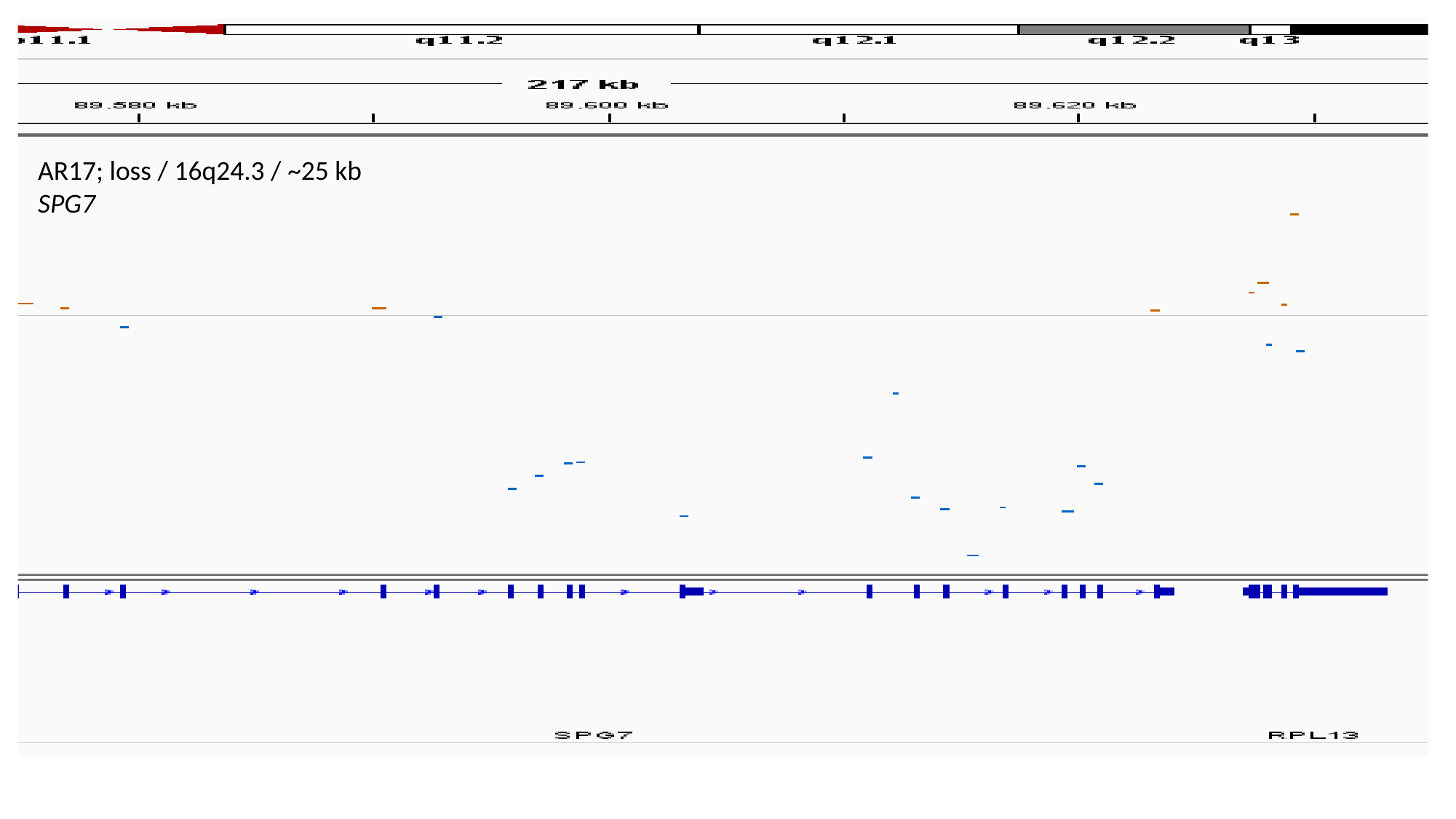

AR17; loss / 16q24.3 / ~25 kb
SPG7

## Slide 54
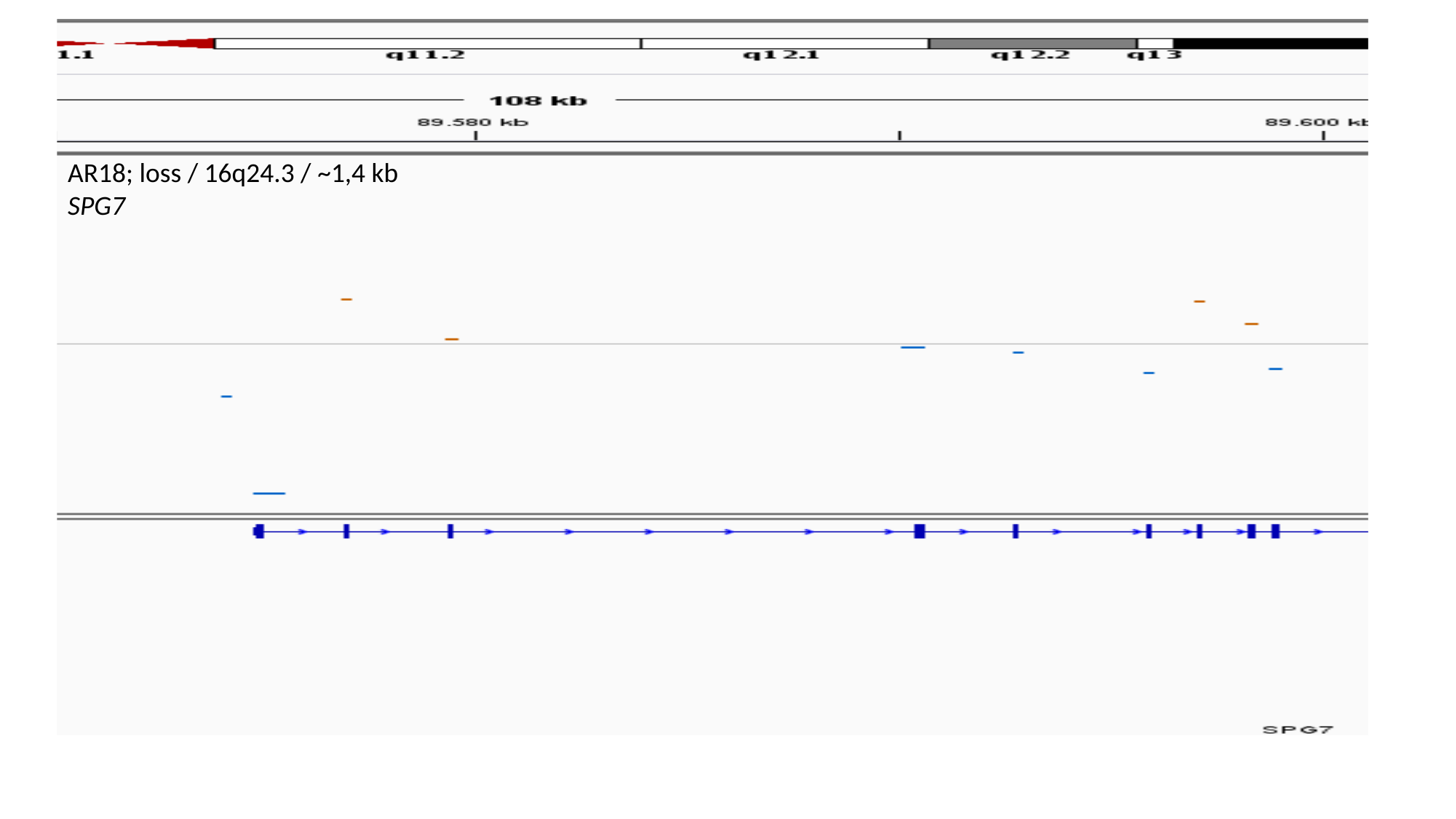

AR18; loss / 16q24.3 / ~1,4 kb
SPG7

## Slide 55
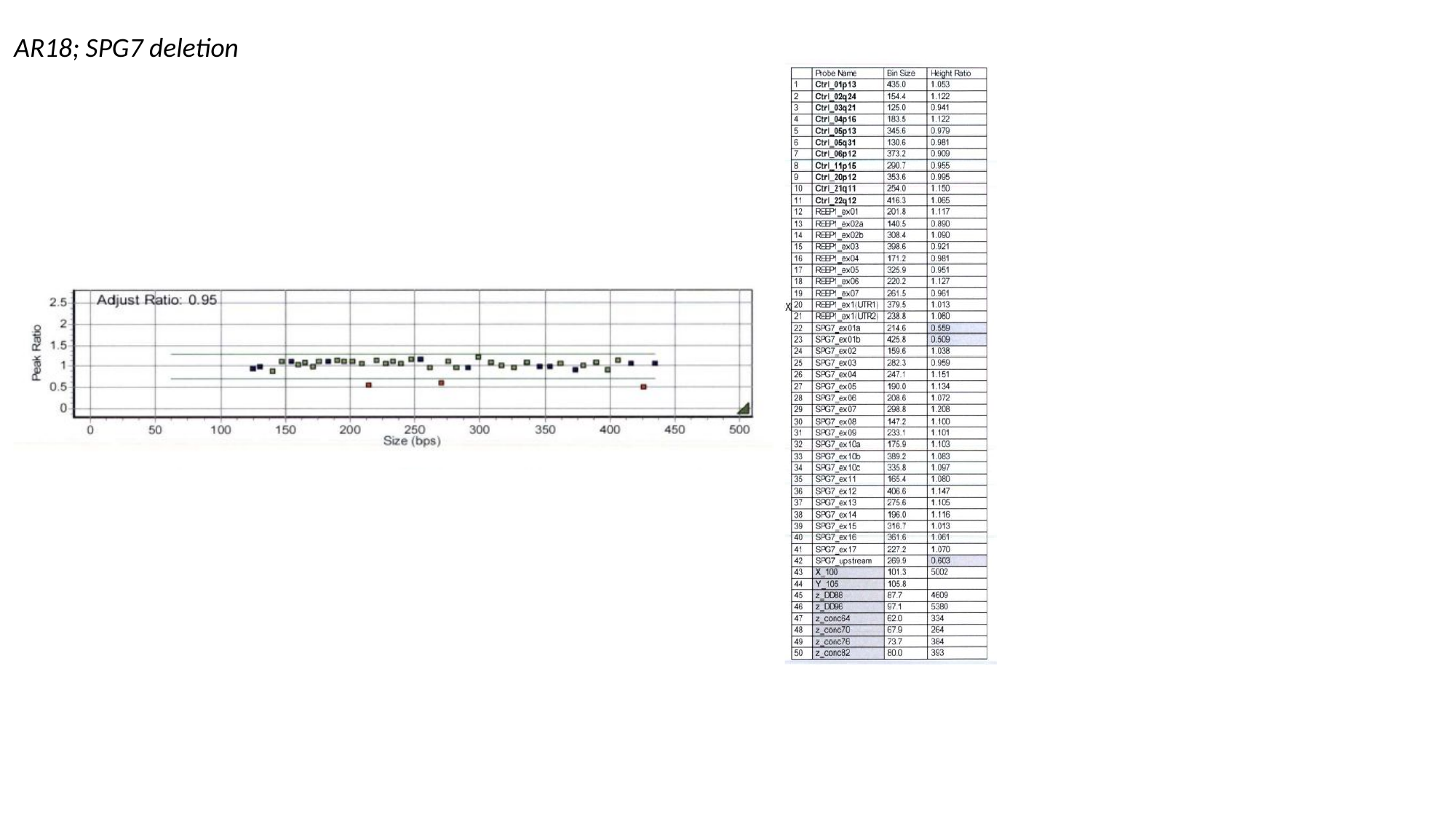

AR18; SPG7 deletion

## Slide 56
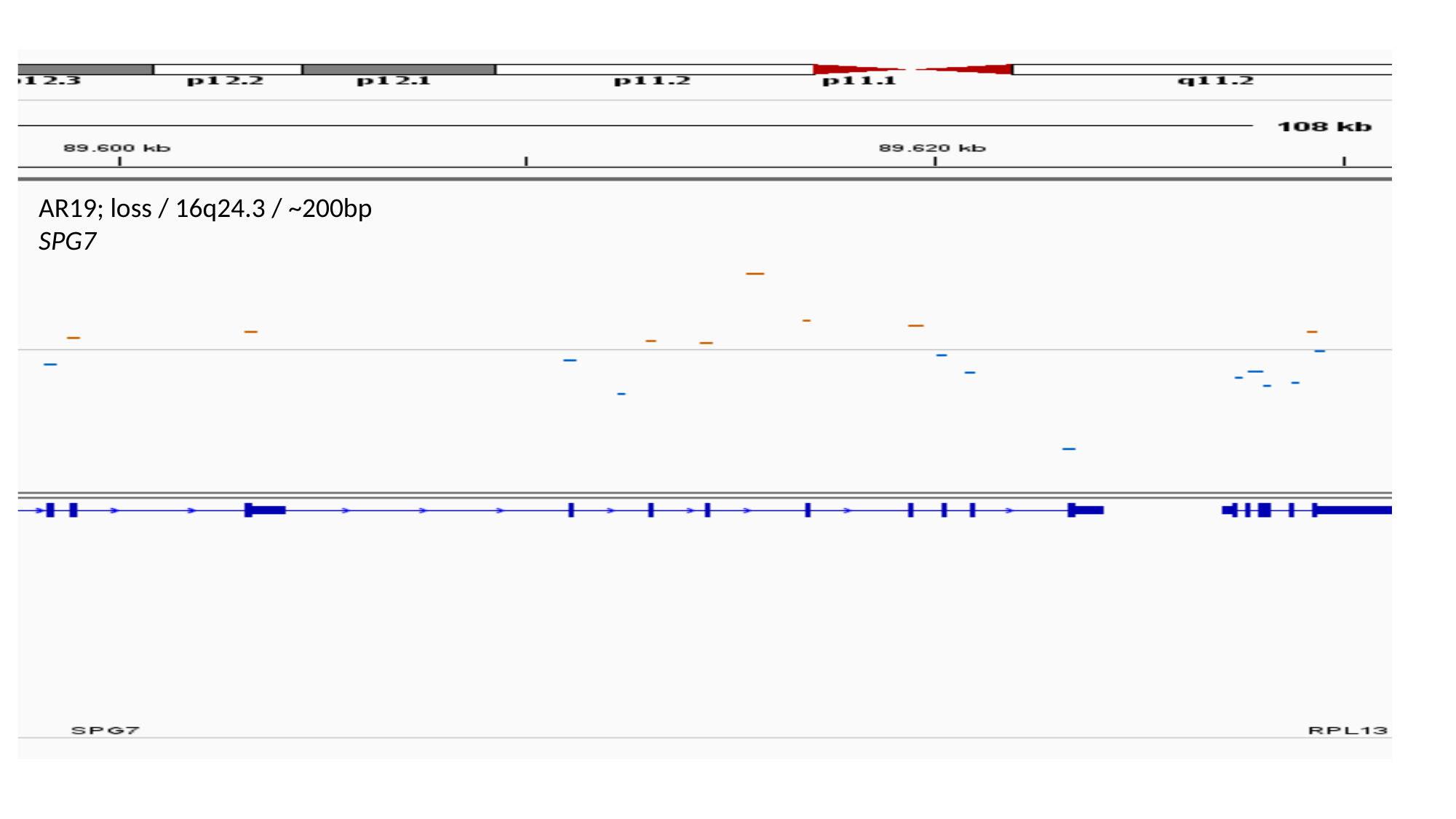

AR19; loss / 16q24.3 / ~200bp
SPG7

## Slide 57
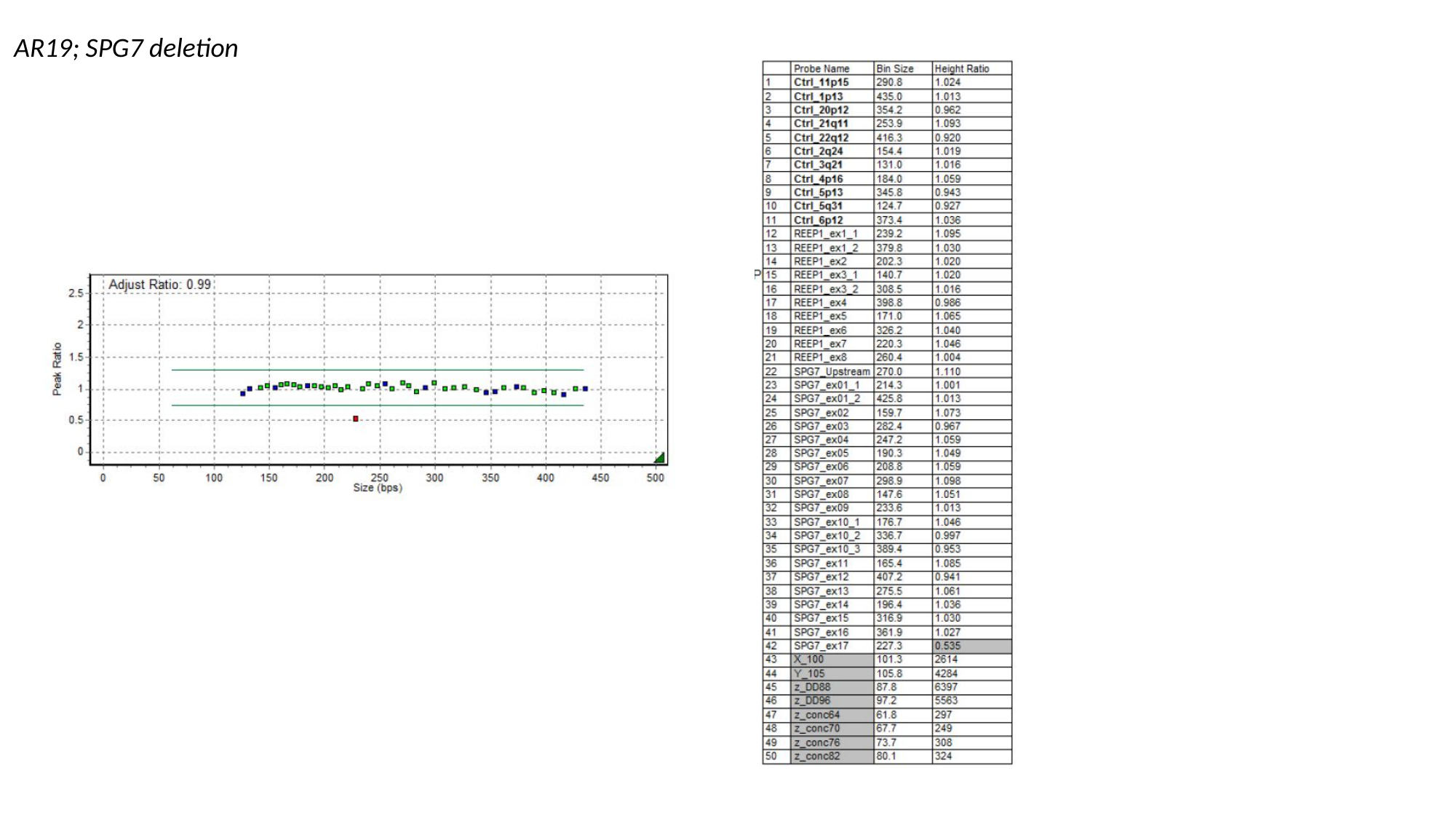

AR19; SPG7 deletion

## Slide 58
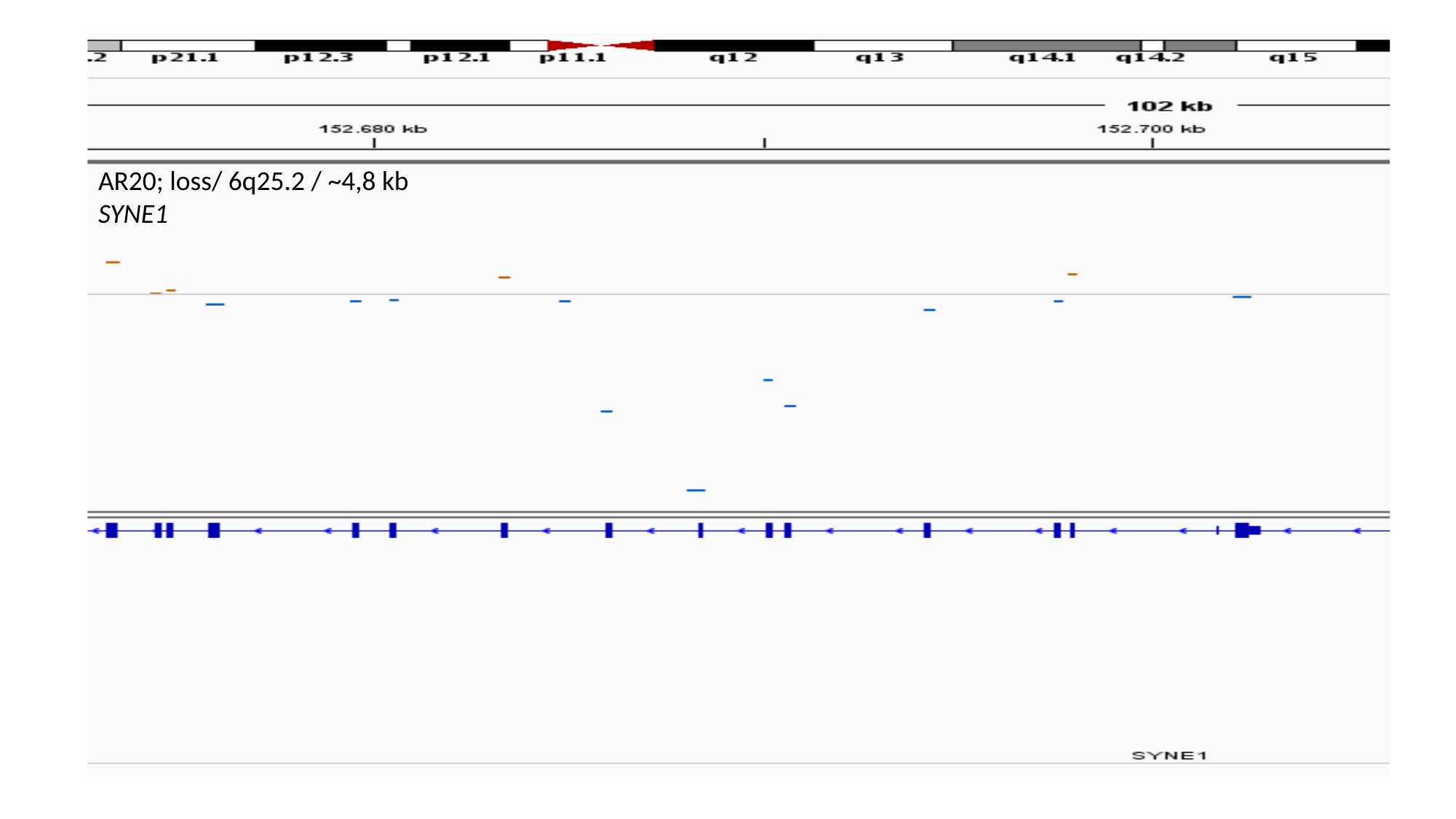

AR20; loss/ 6q25.2 / ~4,8 kb
SYNE1

## Slide 59
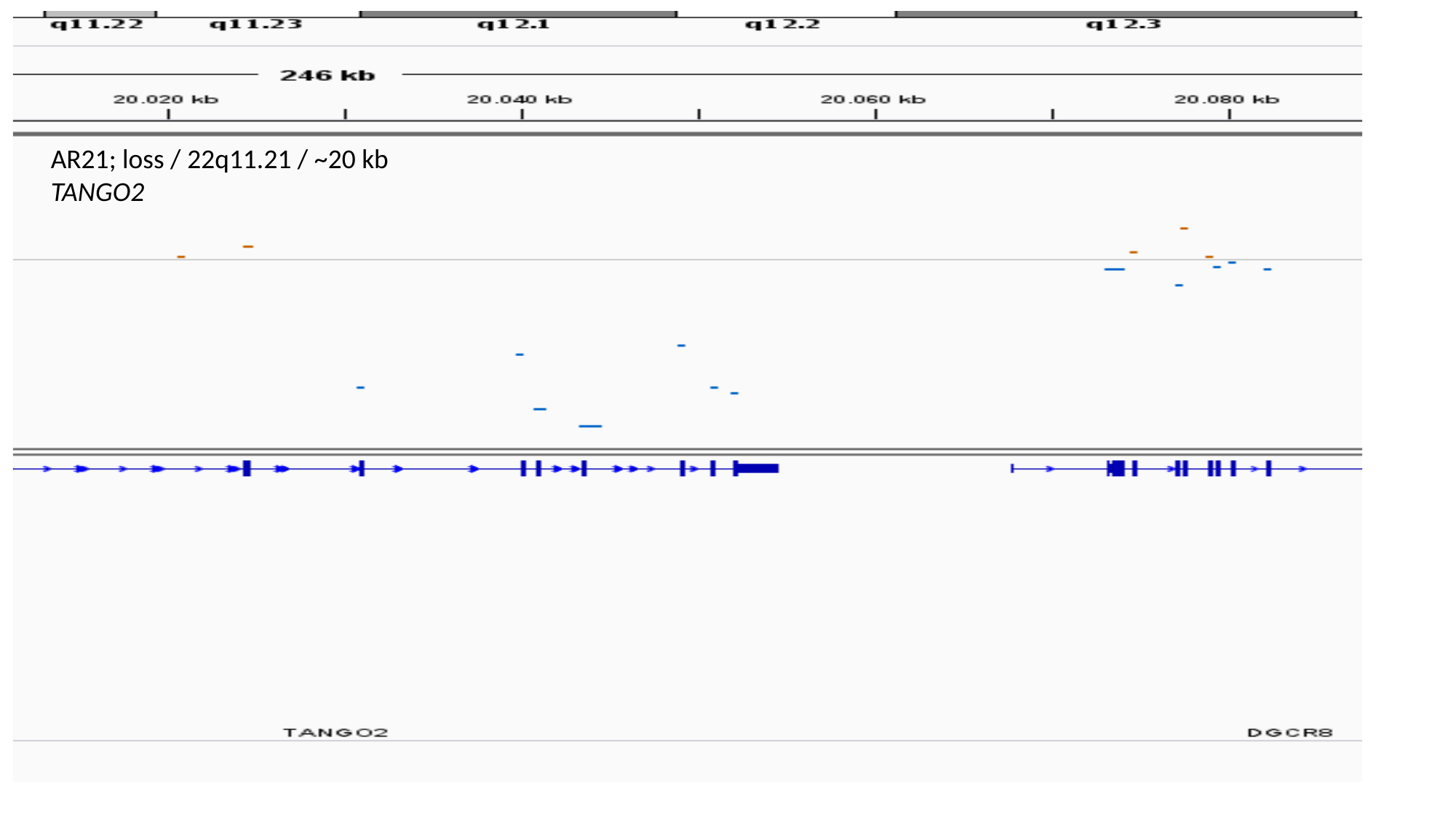

AR21; loss / 22q11.21 / ~20 kb
TANGO2

## Slide 60
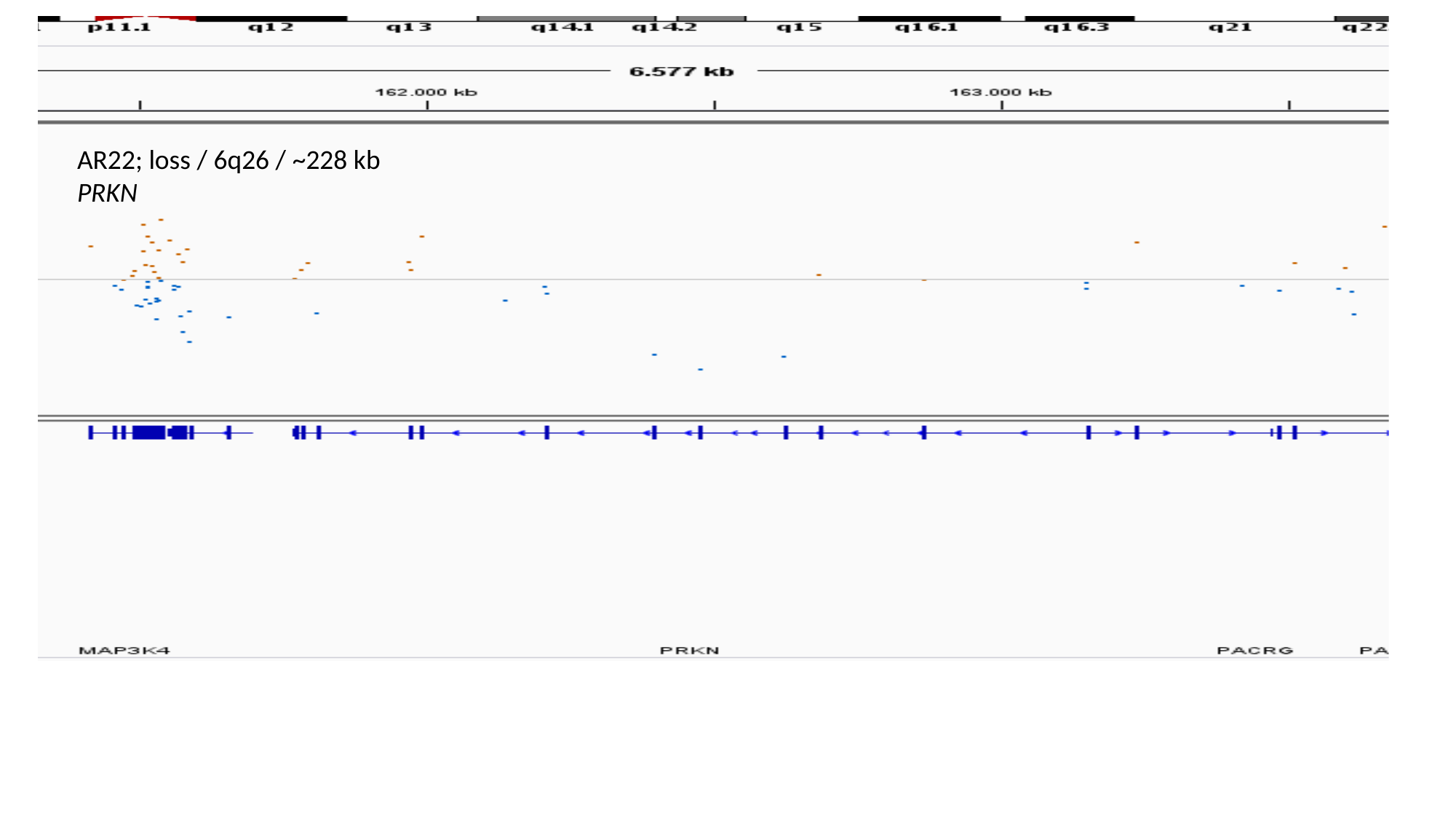

AR22; loss / 6q26 / ~228 kb
PRKN

## Slide 61
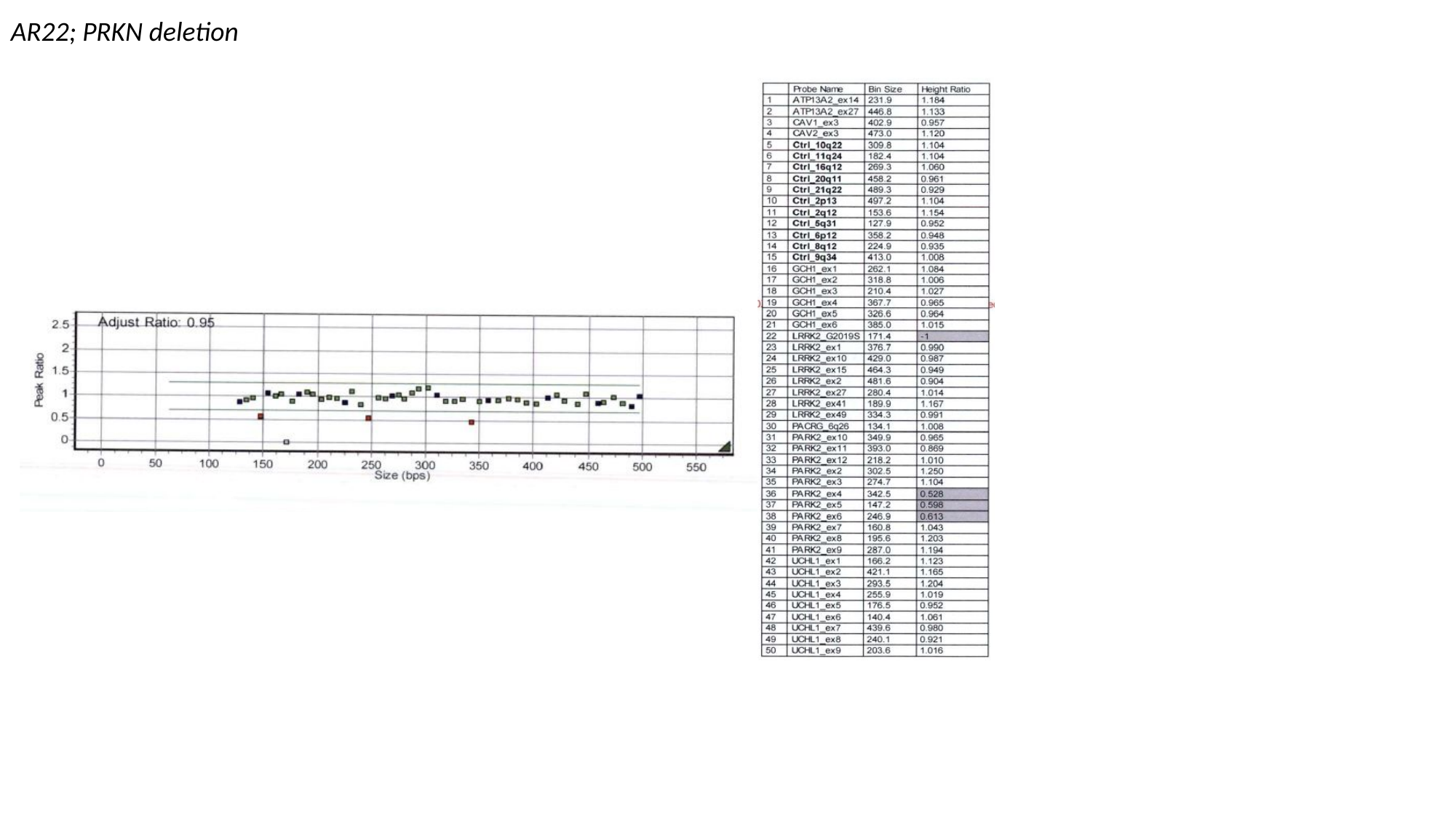

AR22; PRKN deletion

## Slide 62
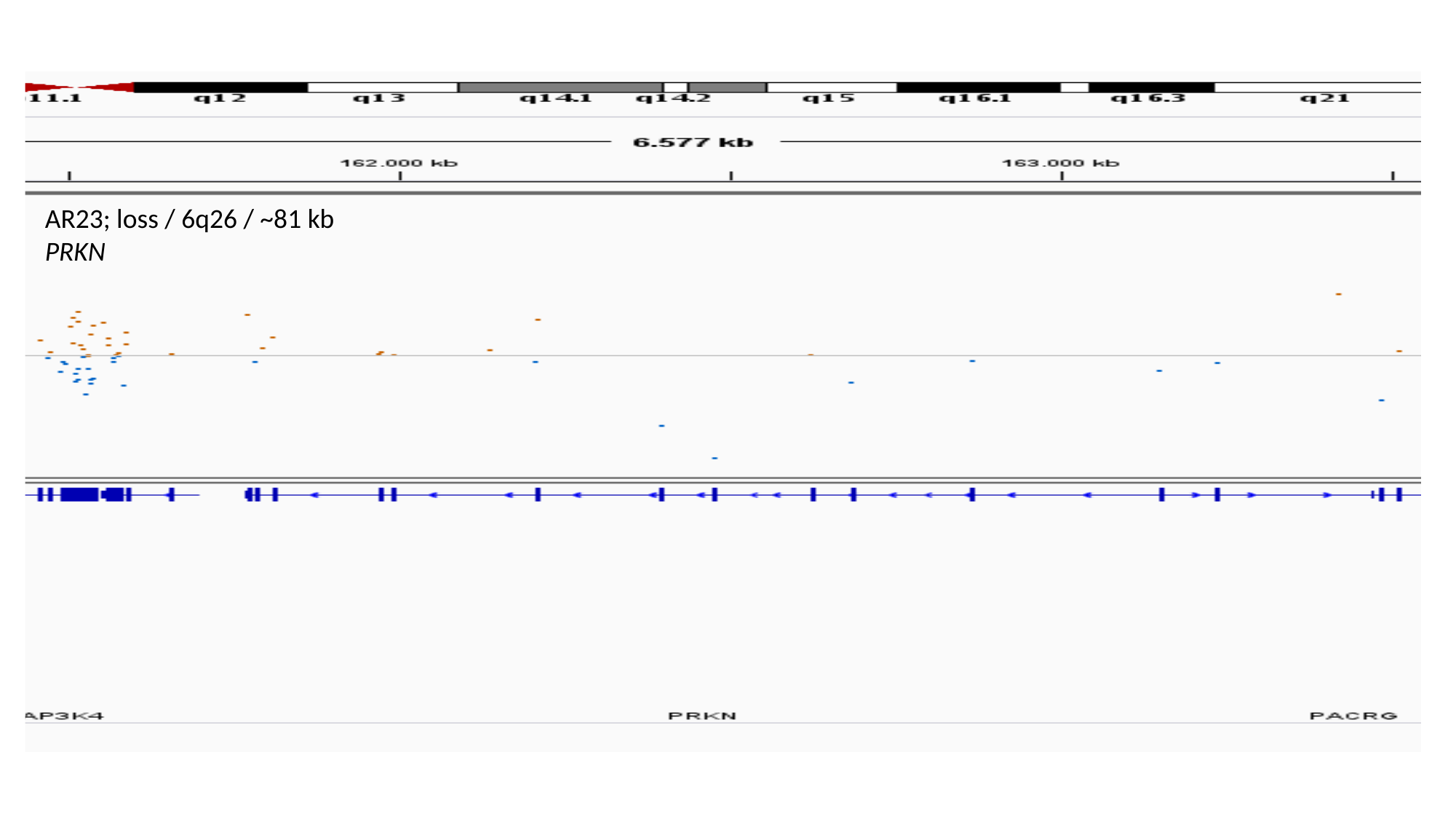

AR23; loss / 6q26 / ~81 kb
PRKN

## Slide 63
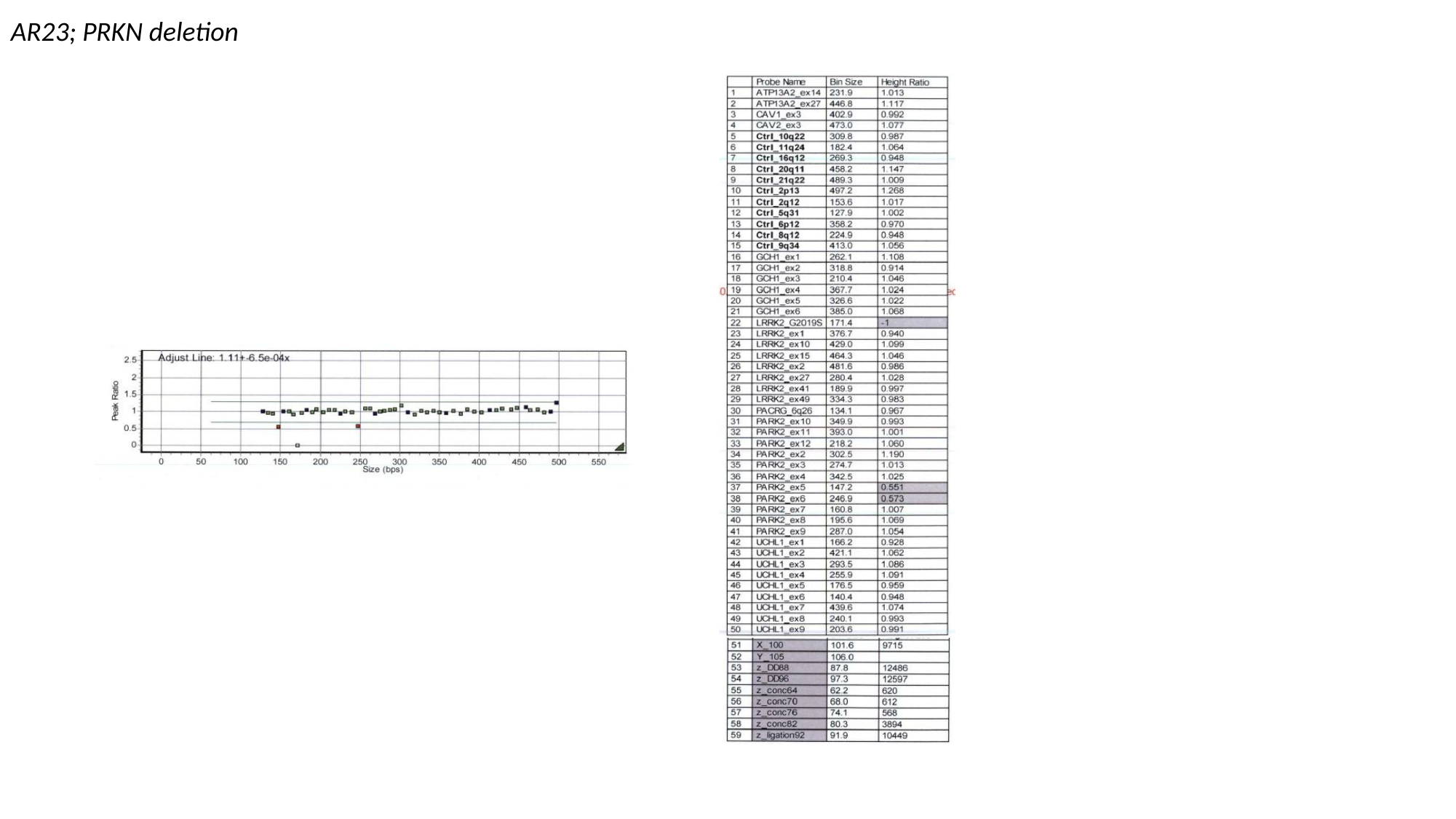

AR23; PRKN deletion

## Slide 64
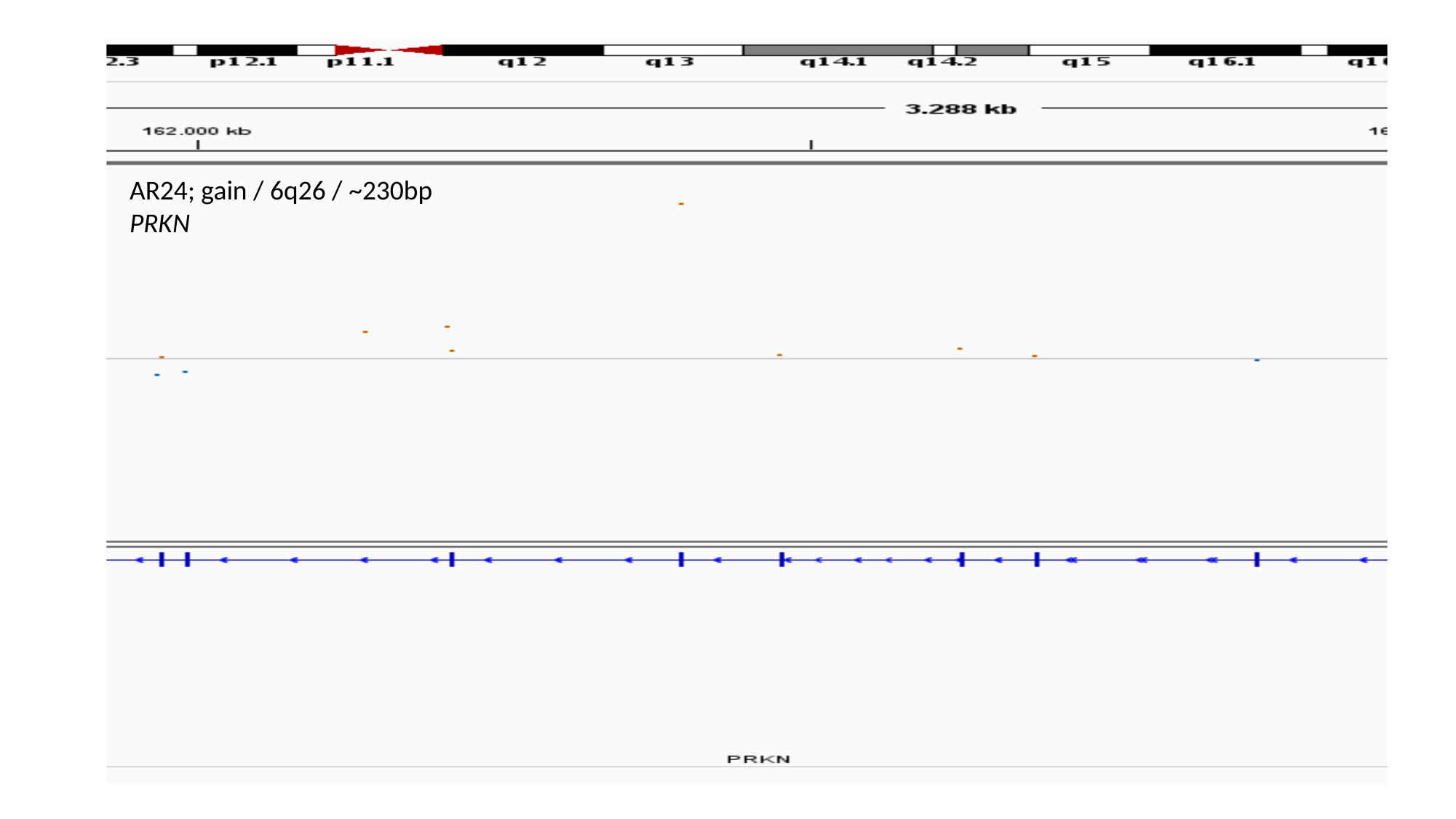

AR24; gain / 6q26 / ~230bp
PRKN

## Slide 65
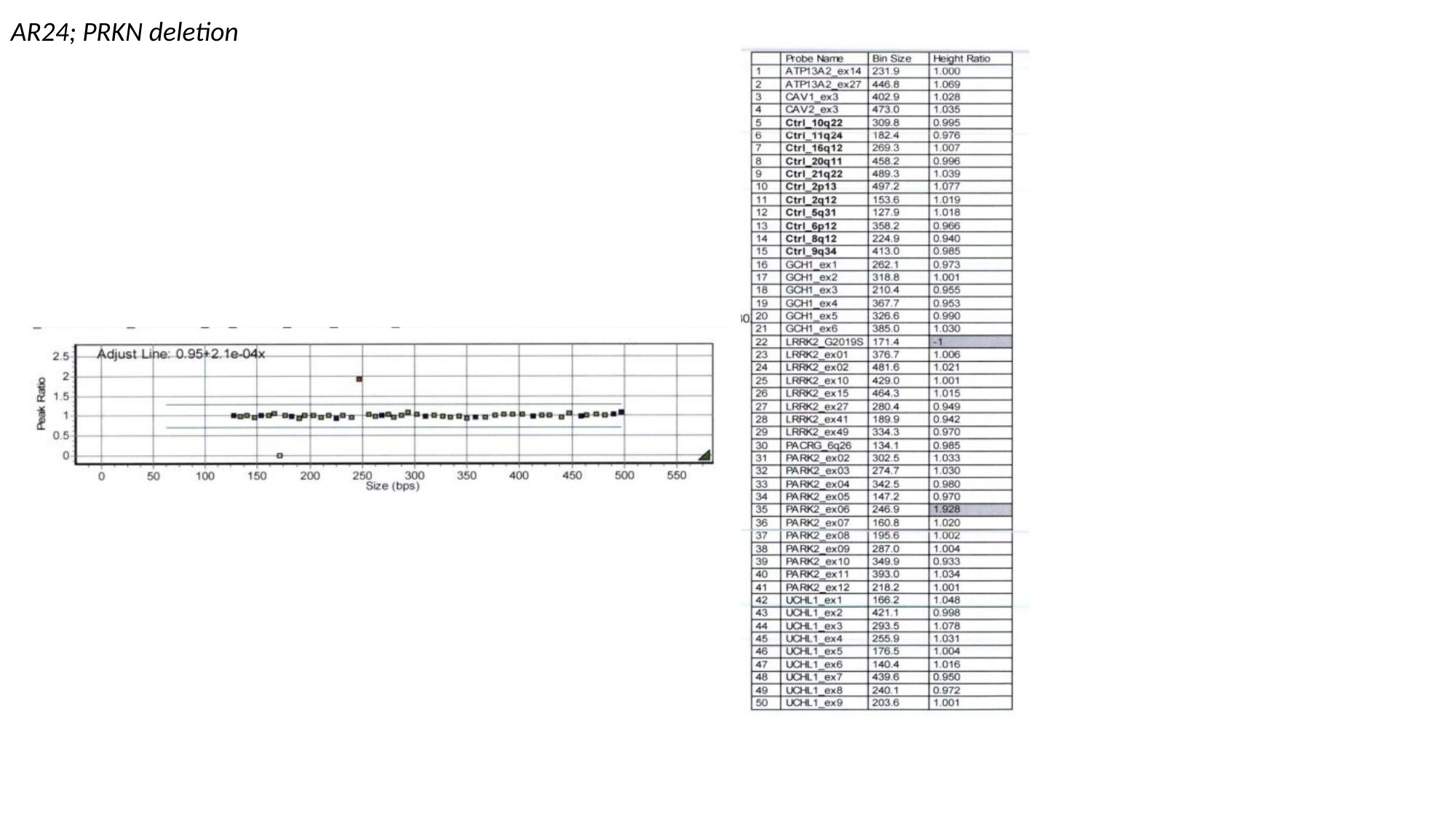

AR24; PRKN deletion

## Slide 66
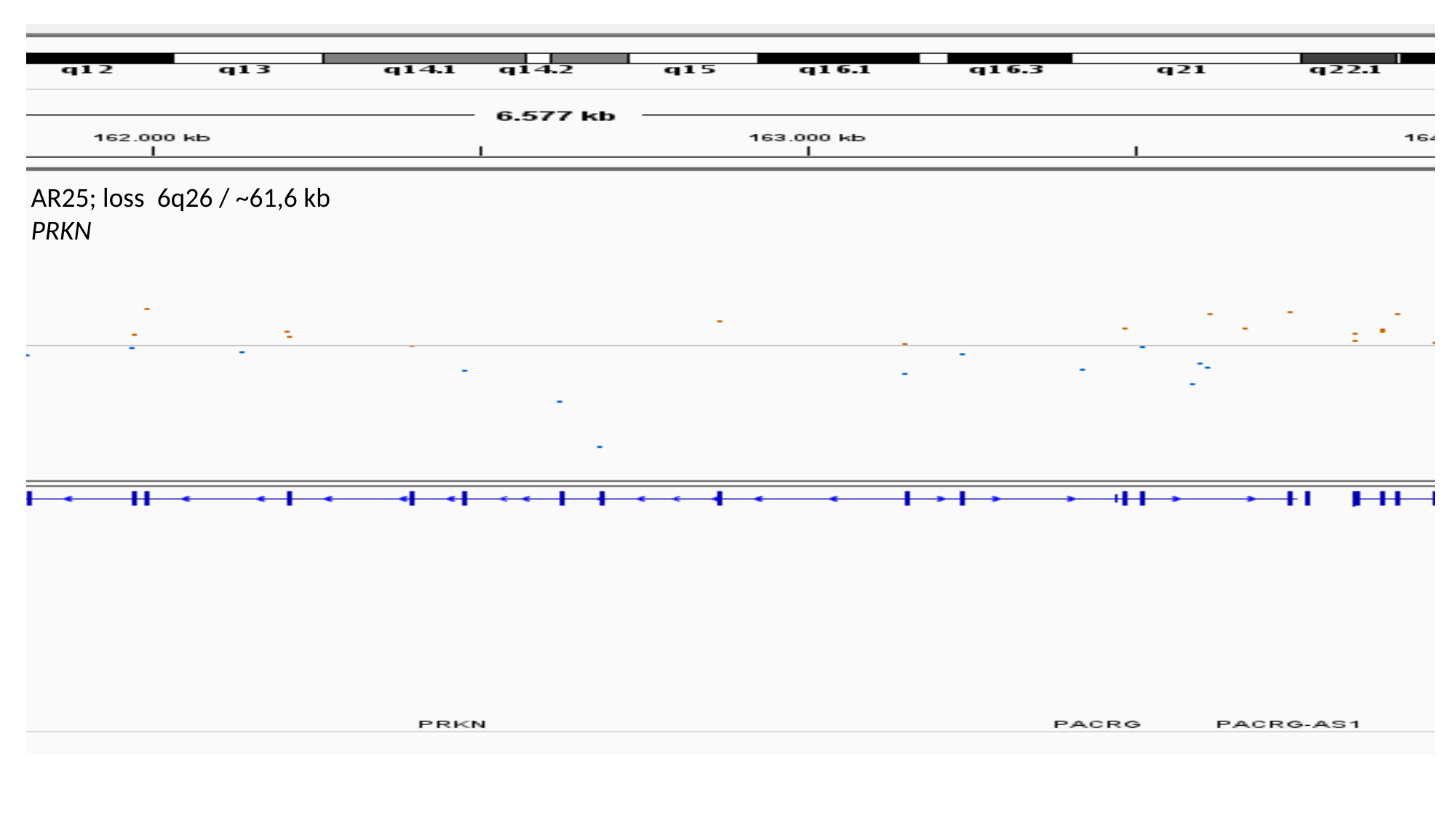

AR25; loss 6q26 / ~61,6 kb
PRKN

## Slide 67
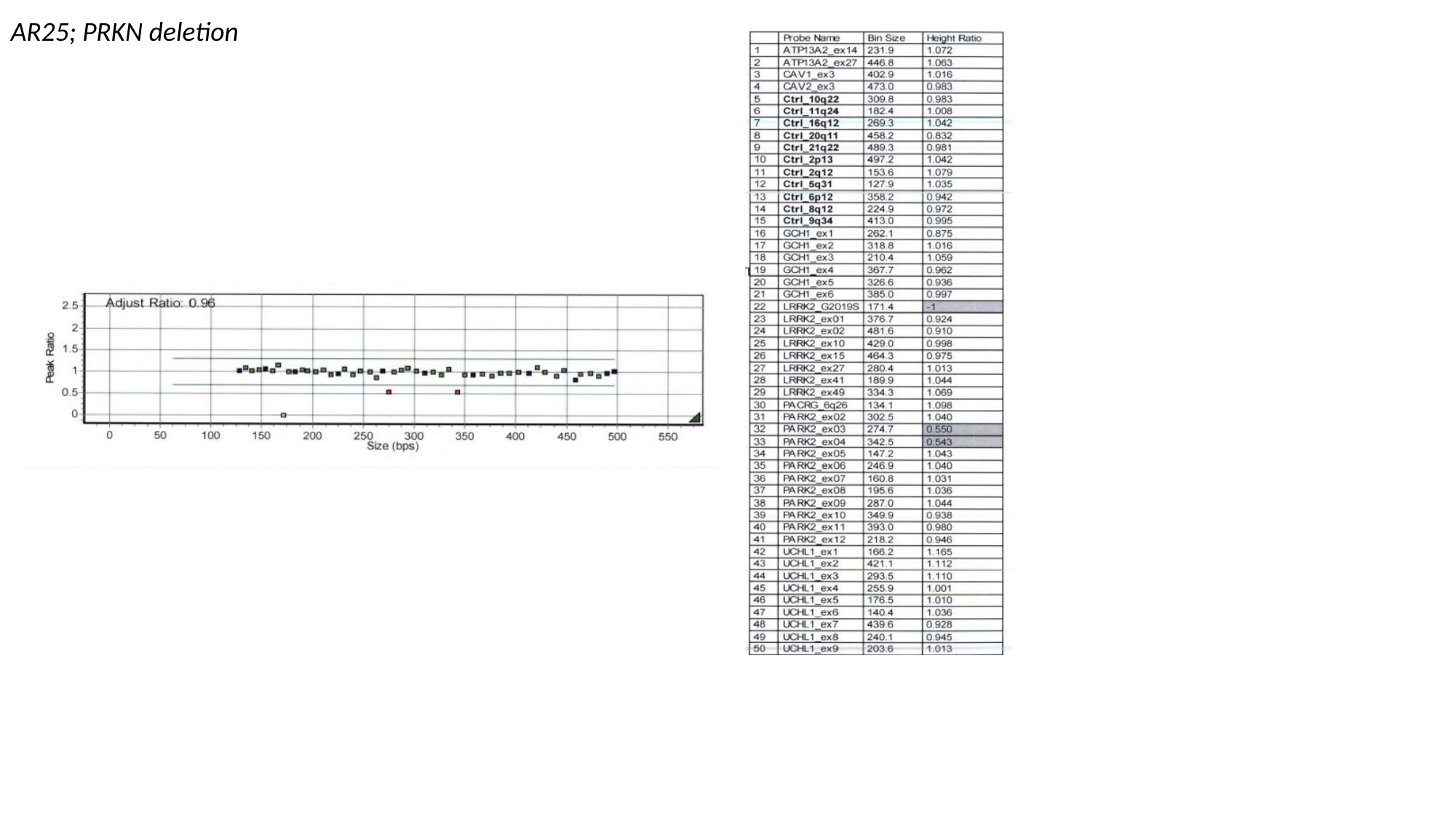

AR25; PRKN deletion

## Slide 68
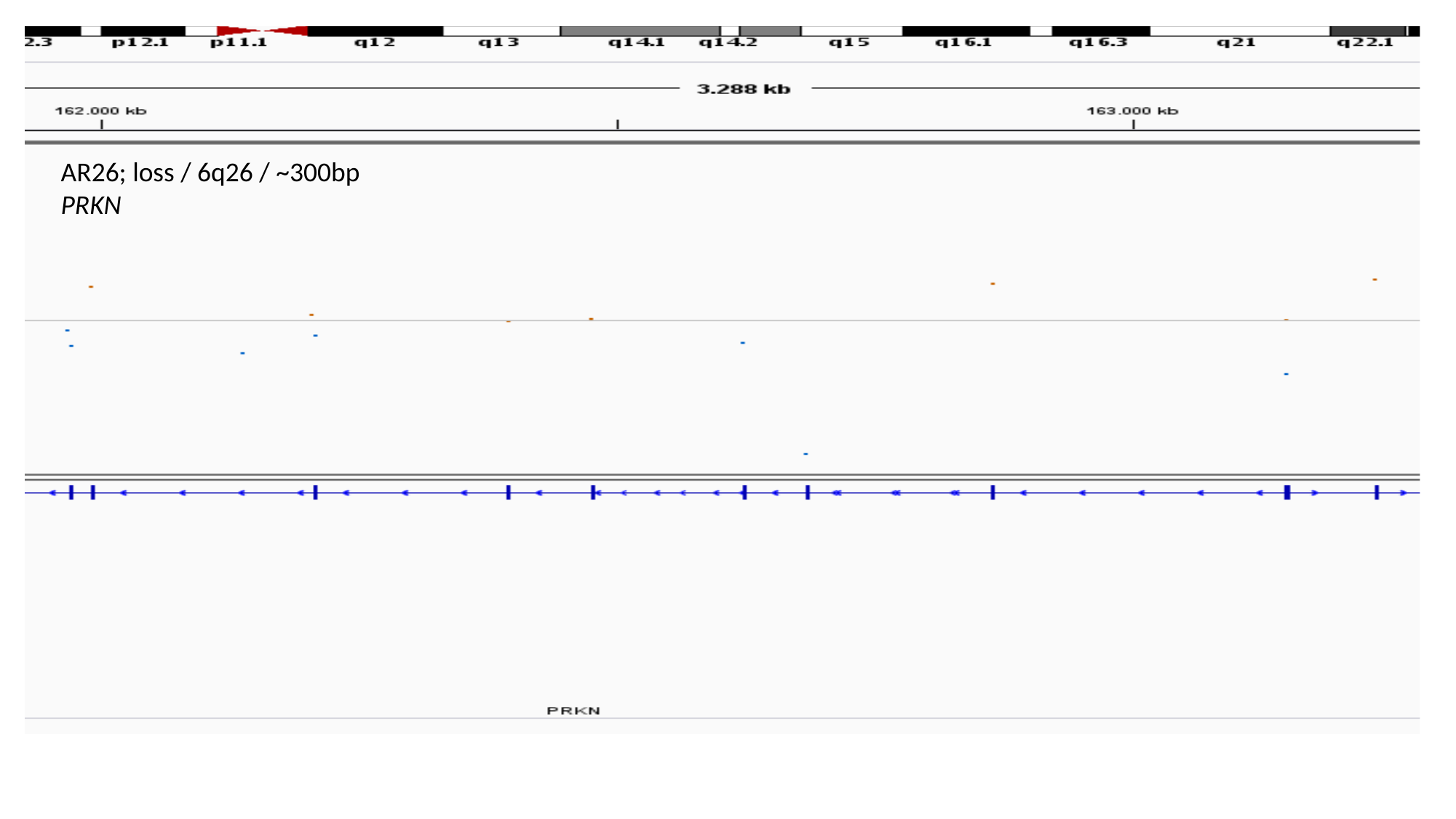

AR26; loss / 6q26 / ~300bp
PRKN

## Slide 69
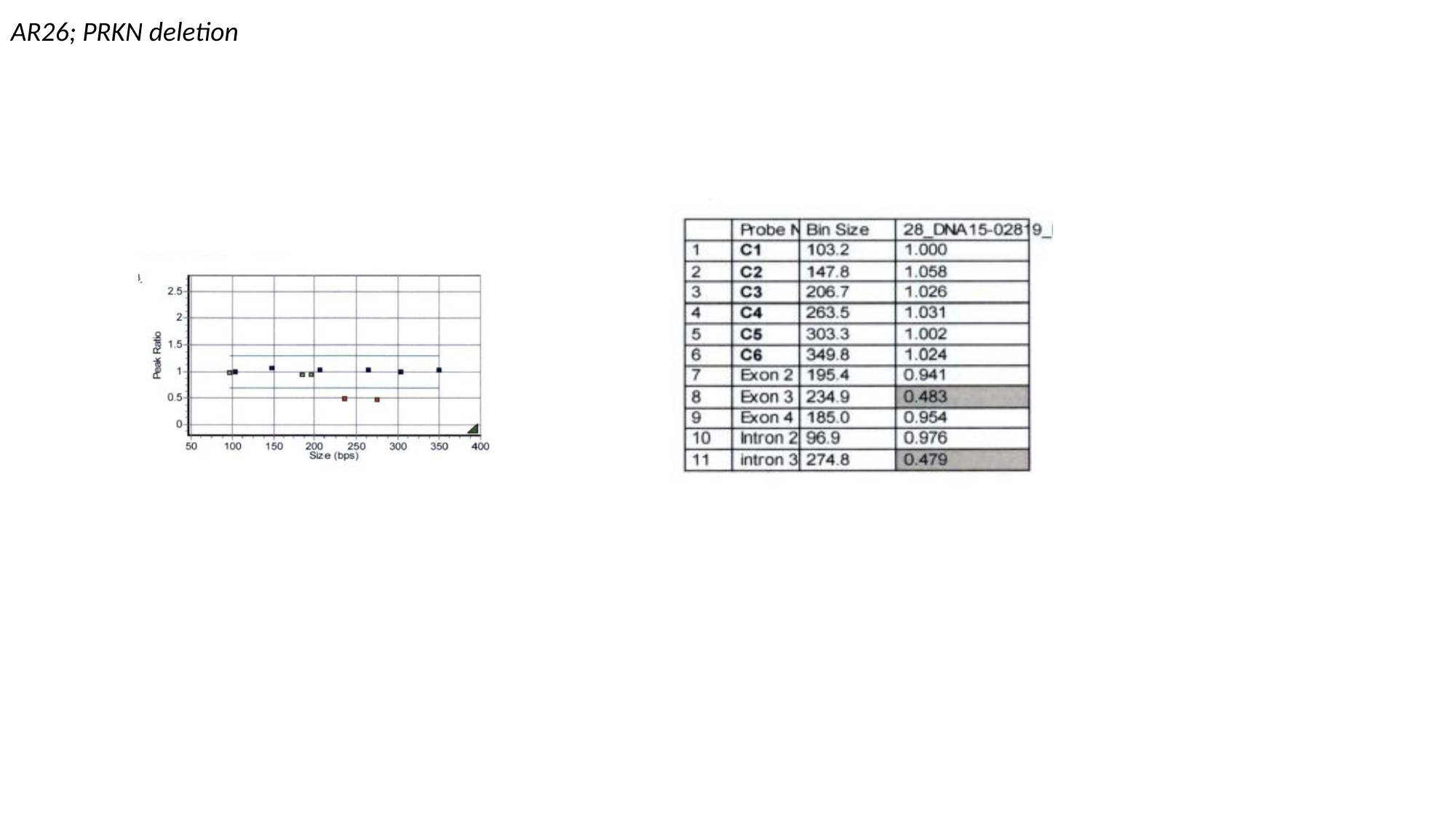

AR26; PRKN deletion

## Slide 70
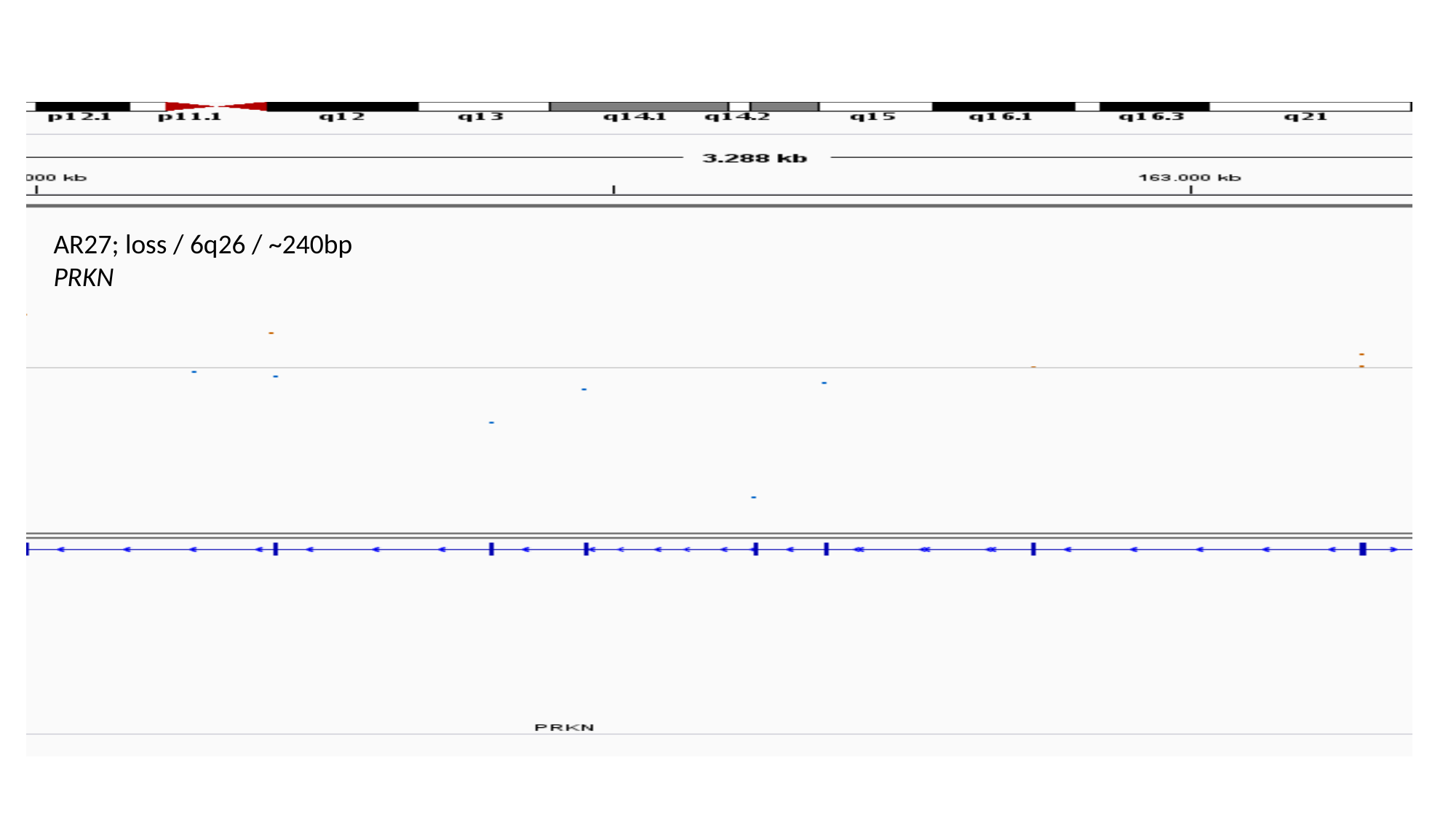

AR27; loss / 6q26 / ~240bp PRKN

## Slide 71
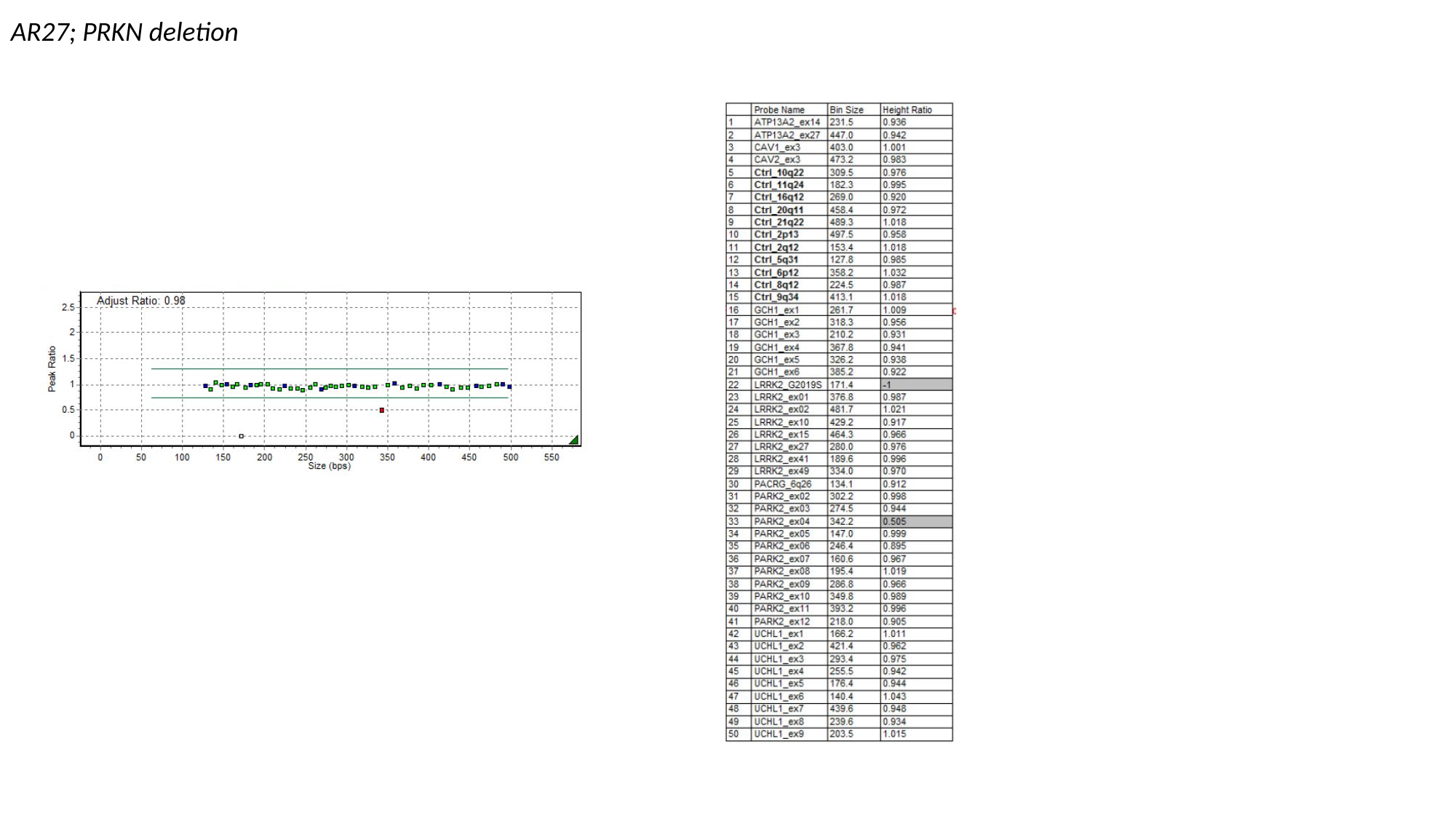

AR27; PRKN deletion

## Slide 72
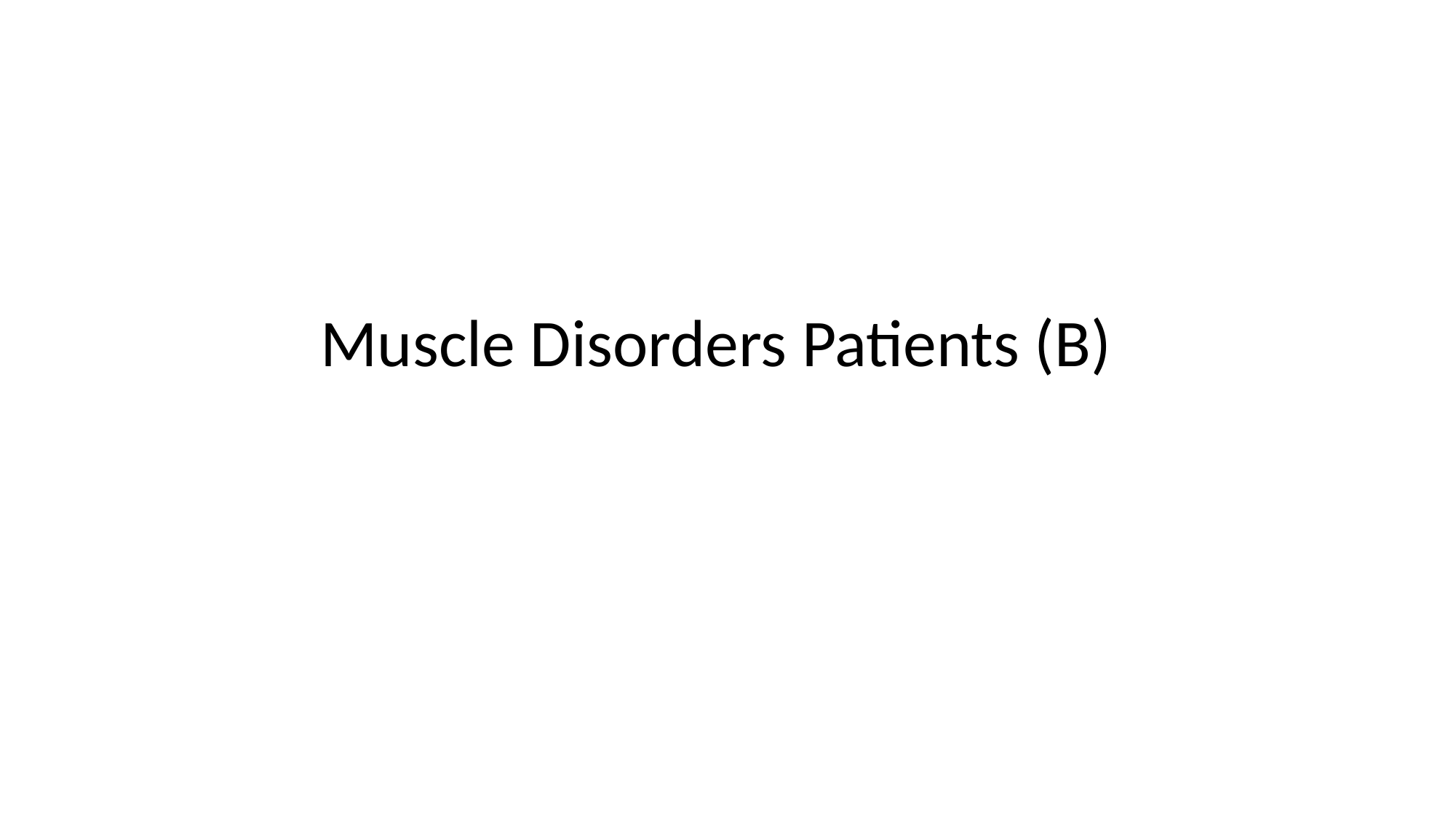

Muscle Disorders Patients (B)

## Slide 73
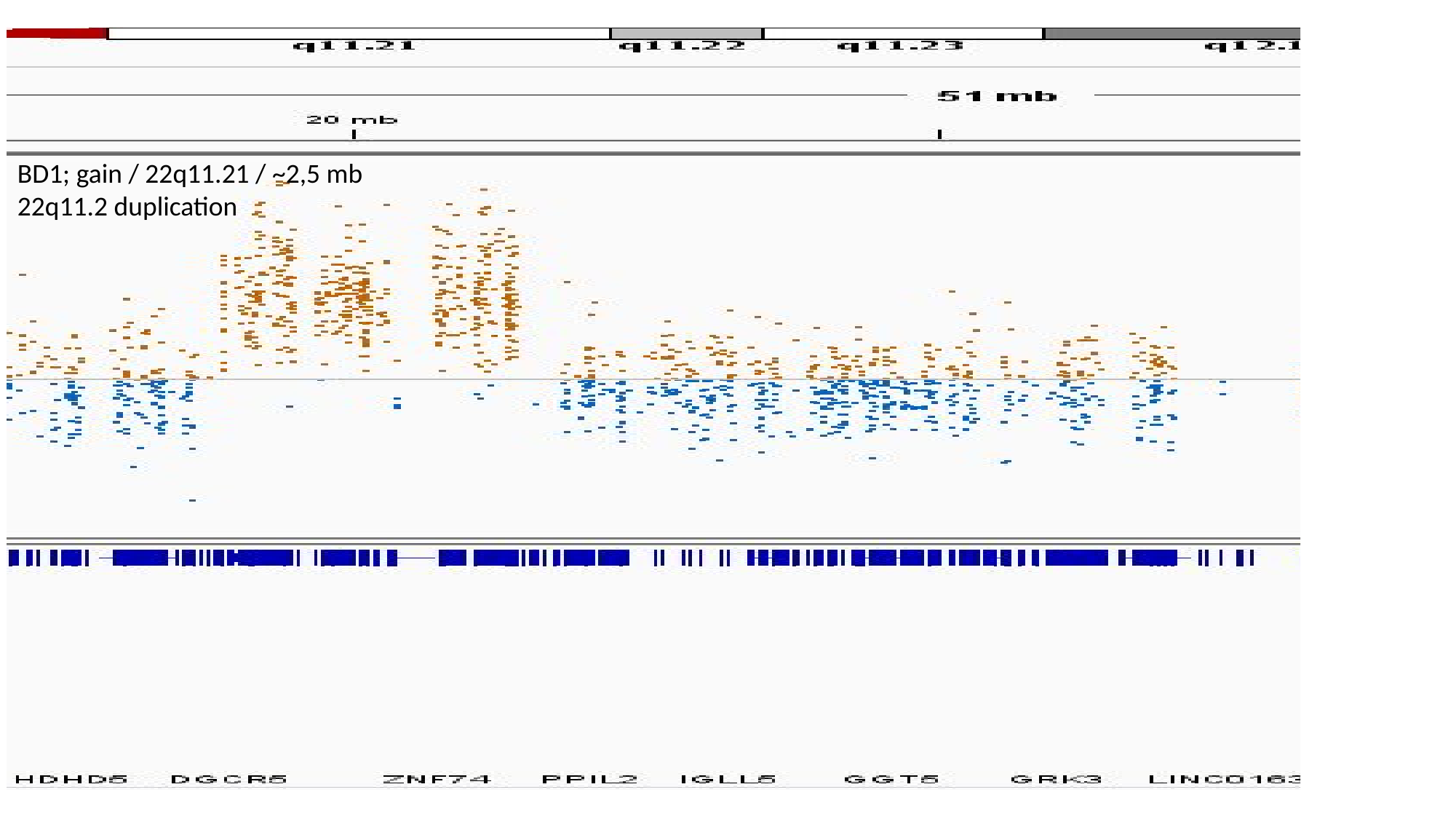

BD1; gain / 22q11.21 / ~2,5 mb
22q11.2 duplication

## Slide 74
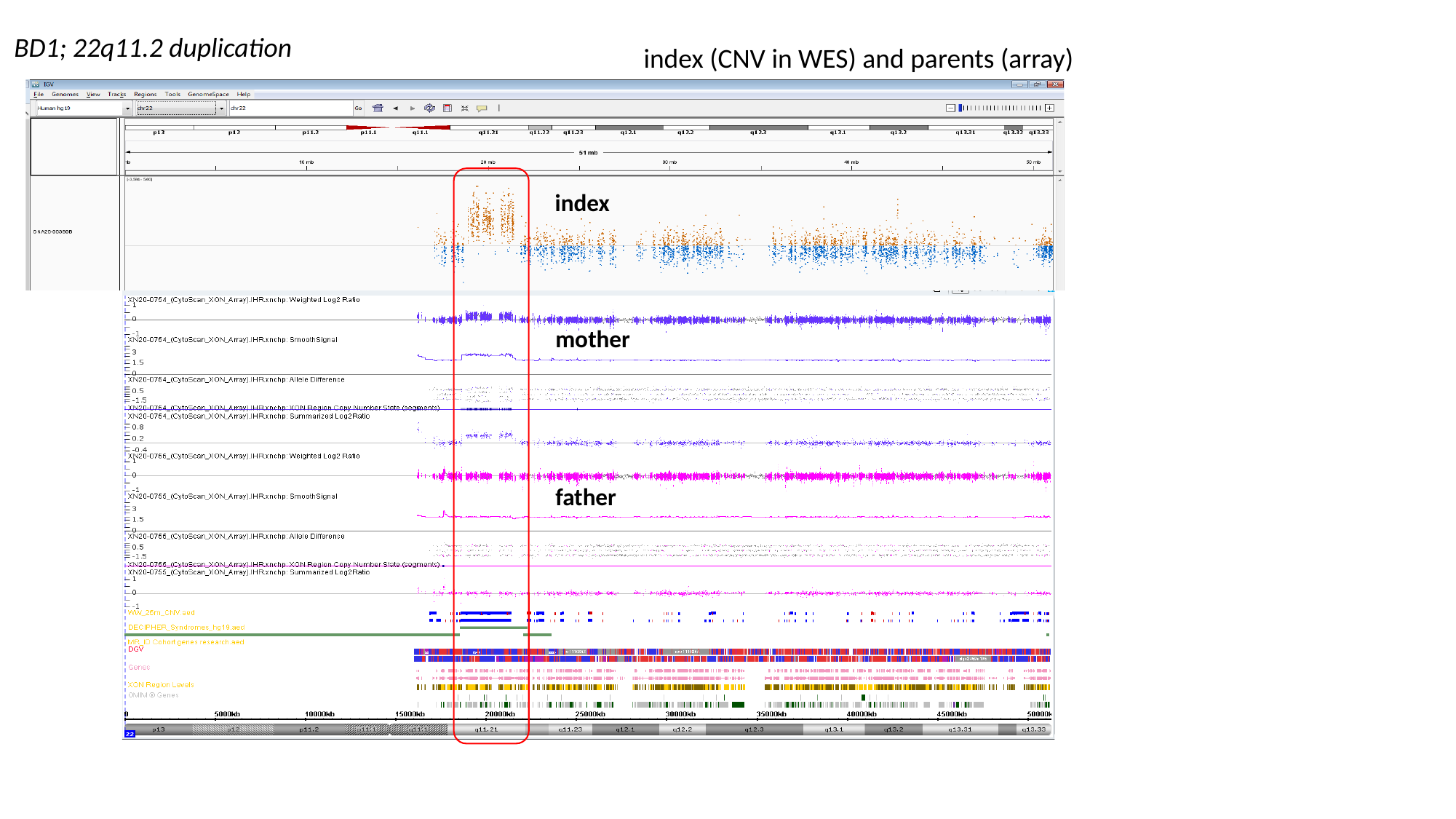

BD1; 22q11.2 duplication
 index (CNV in WES) and parents (array)
index
mother
father

## Slide 75
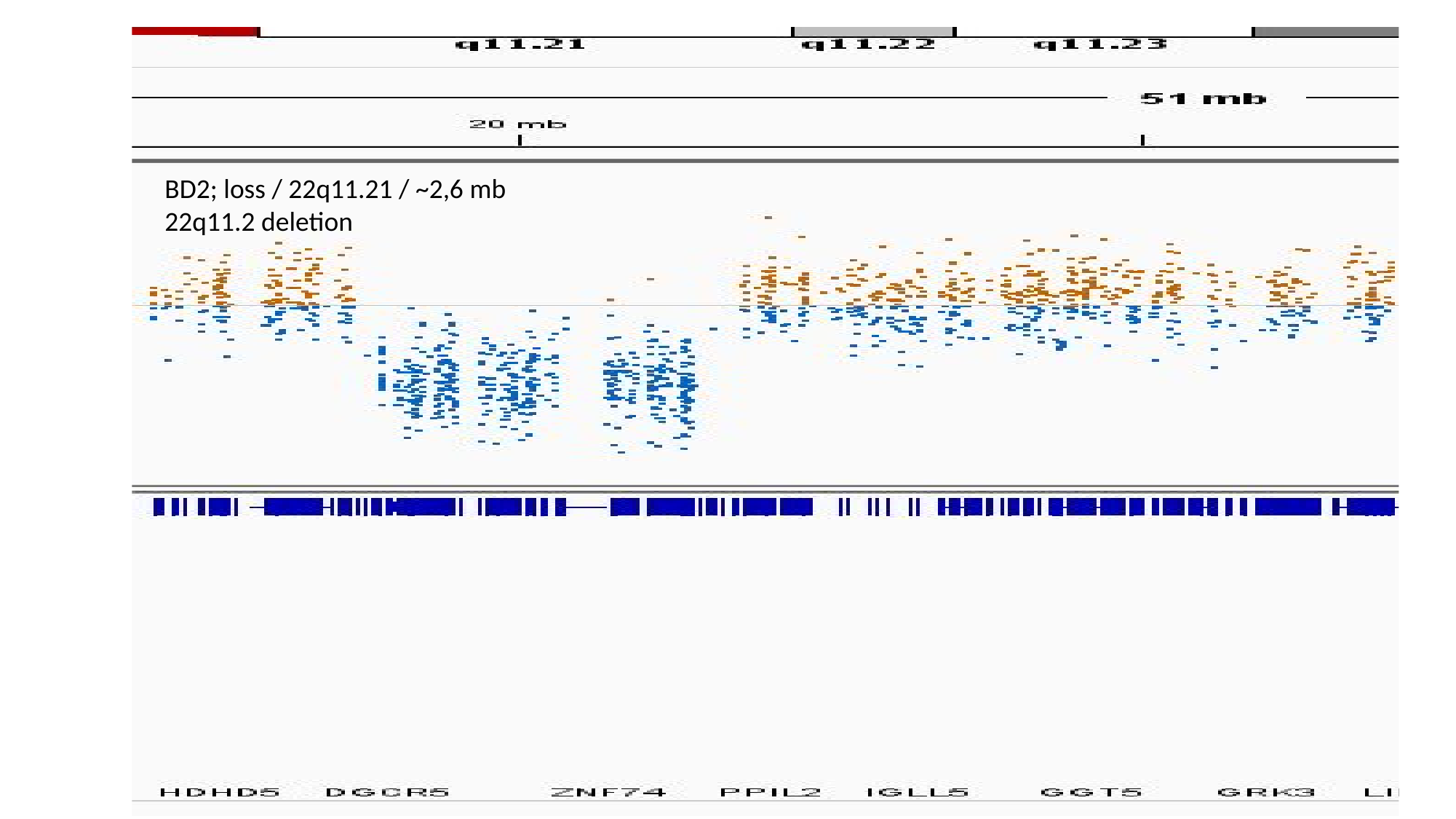

BD2; loss / 22q11.21 / ~2,6 mb
22q11.2 deletion

## Slide 76
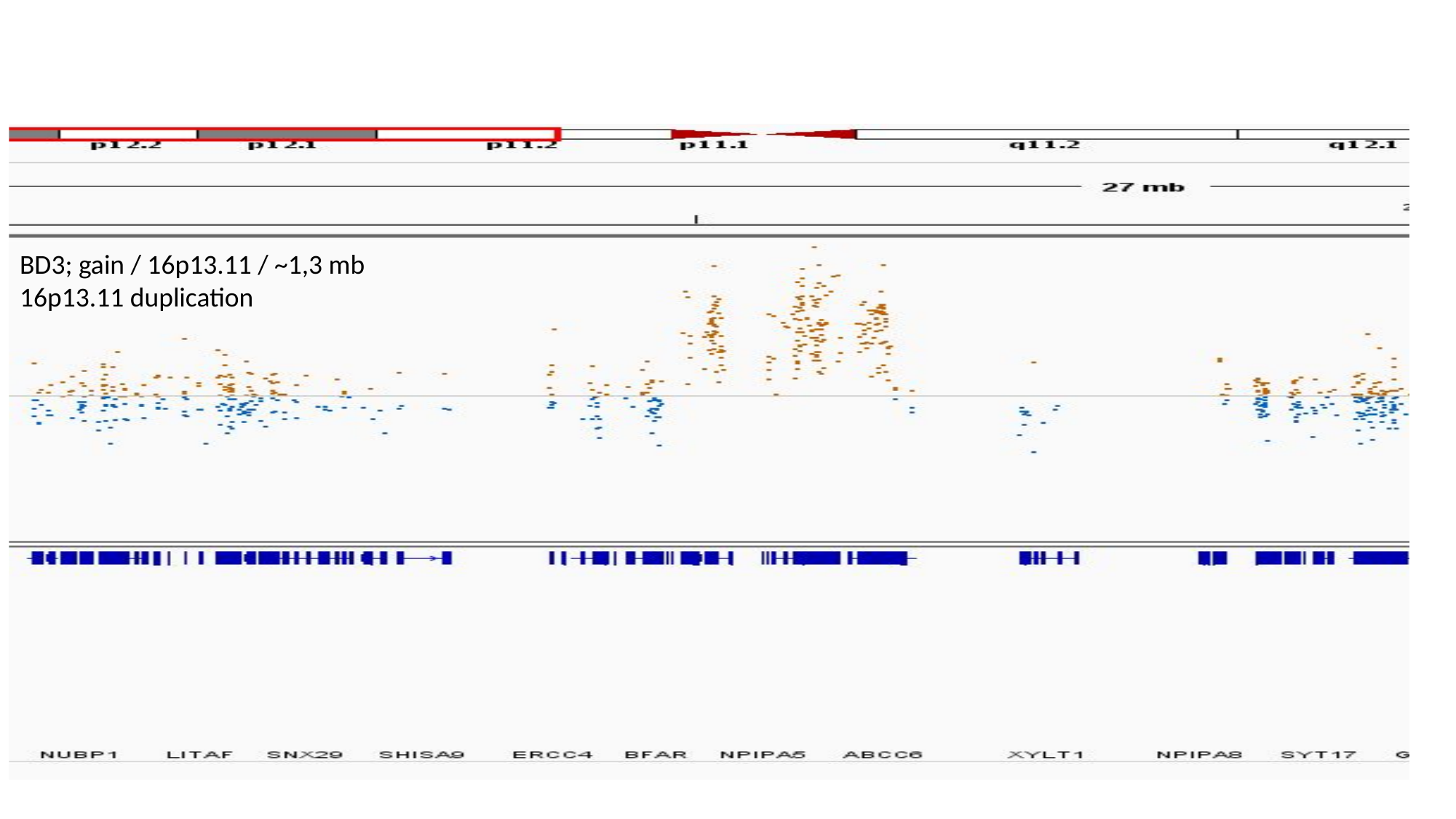

BD3; gain / 16p13.11 / ~1,3 mb
16p13.11 duplication

## Slide 77
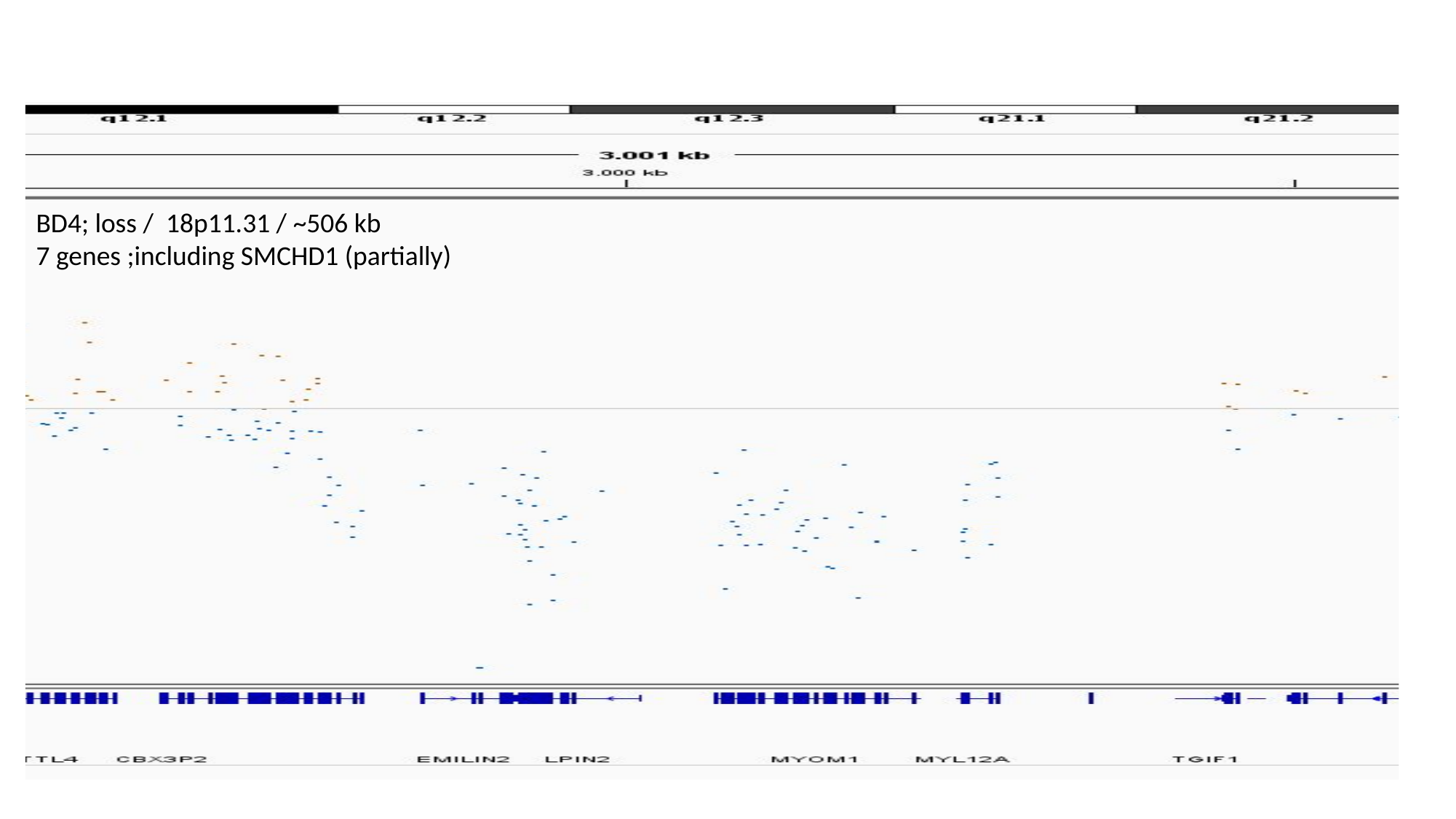

BD4; loss / 18p11.31 / ~506 kb
7 genes ;including SMCHD1 (partially)

## Slide 78
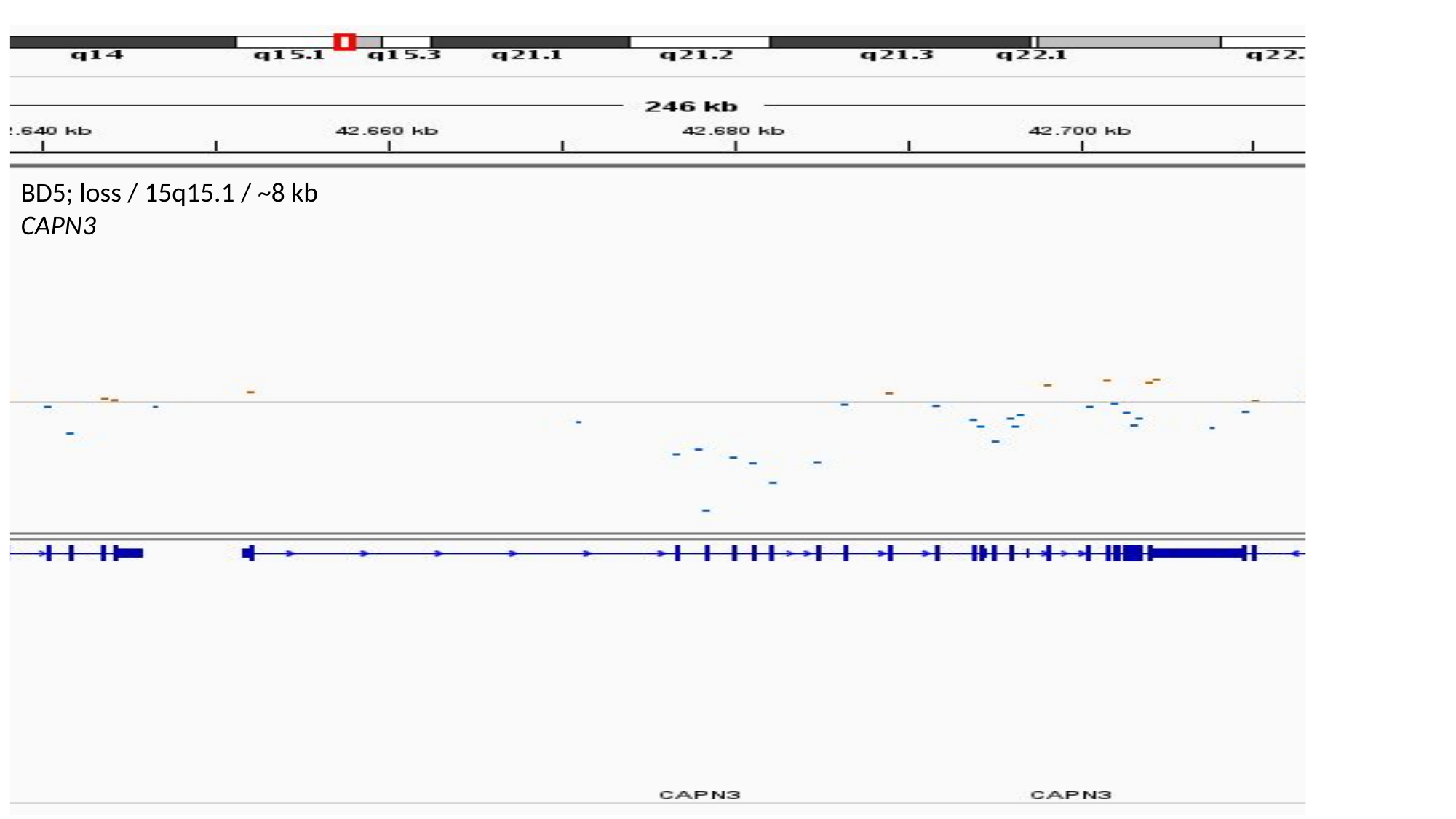

BD5; loss / 15q15.1 / ~8 kb
CAPN3

## Slide 79
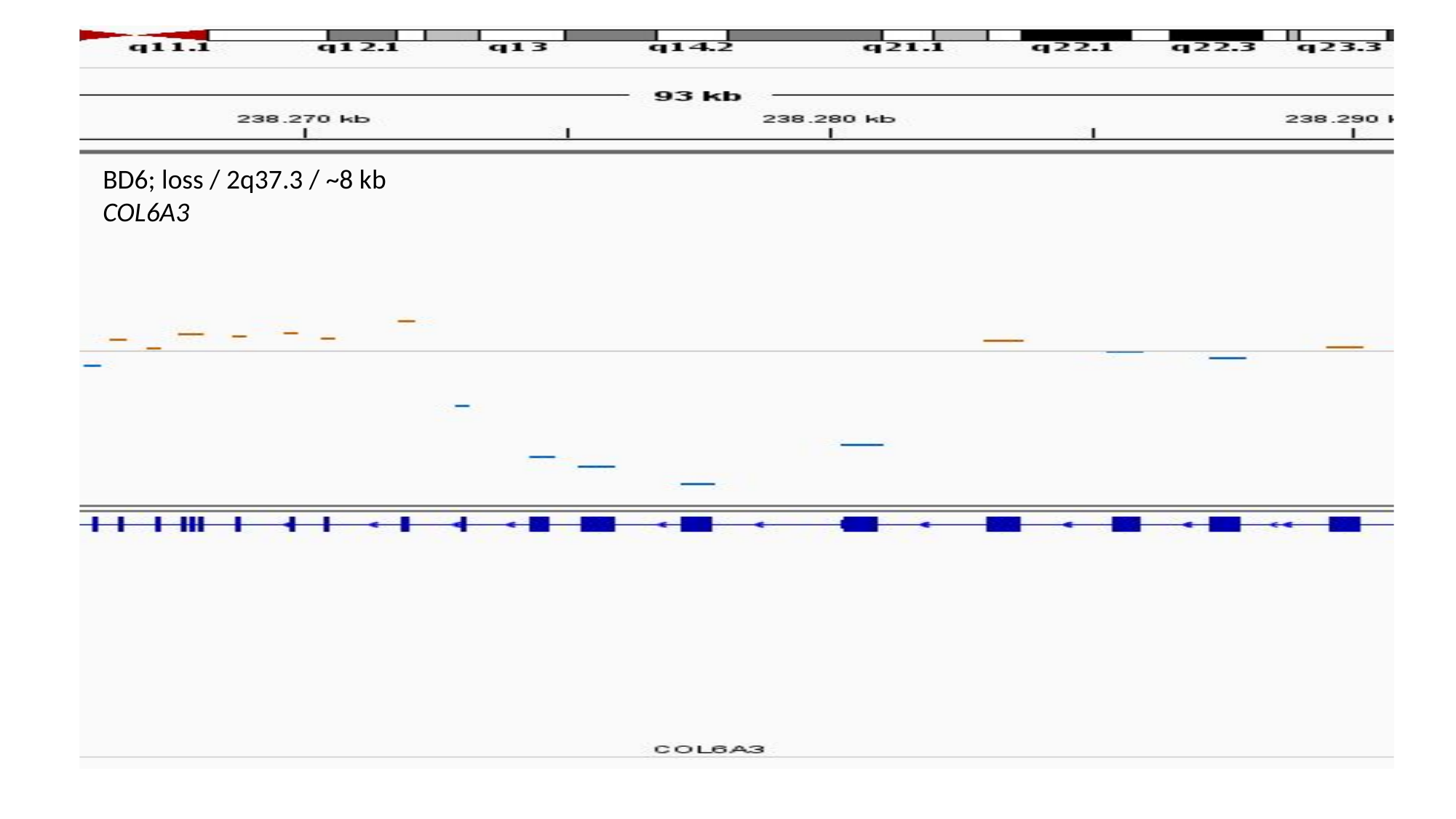

BD6; loss / 2q37.3 / ~8 kb
COL6A3

## Slide 80
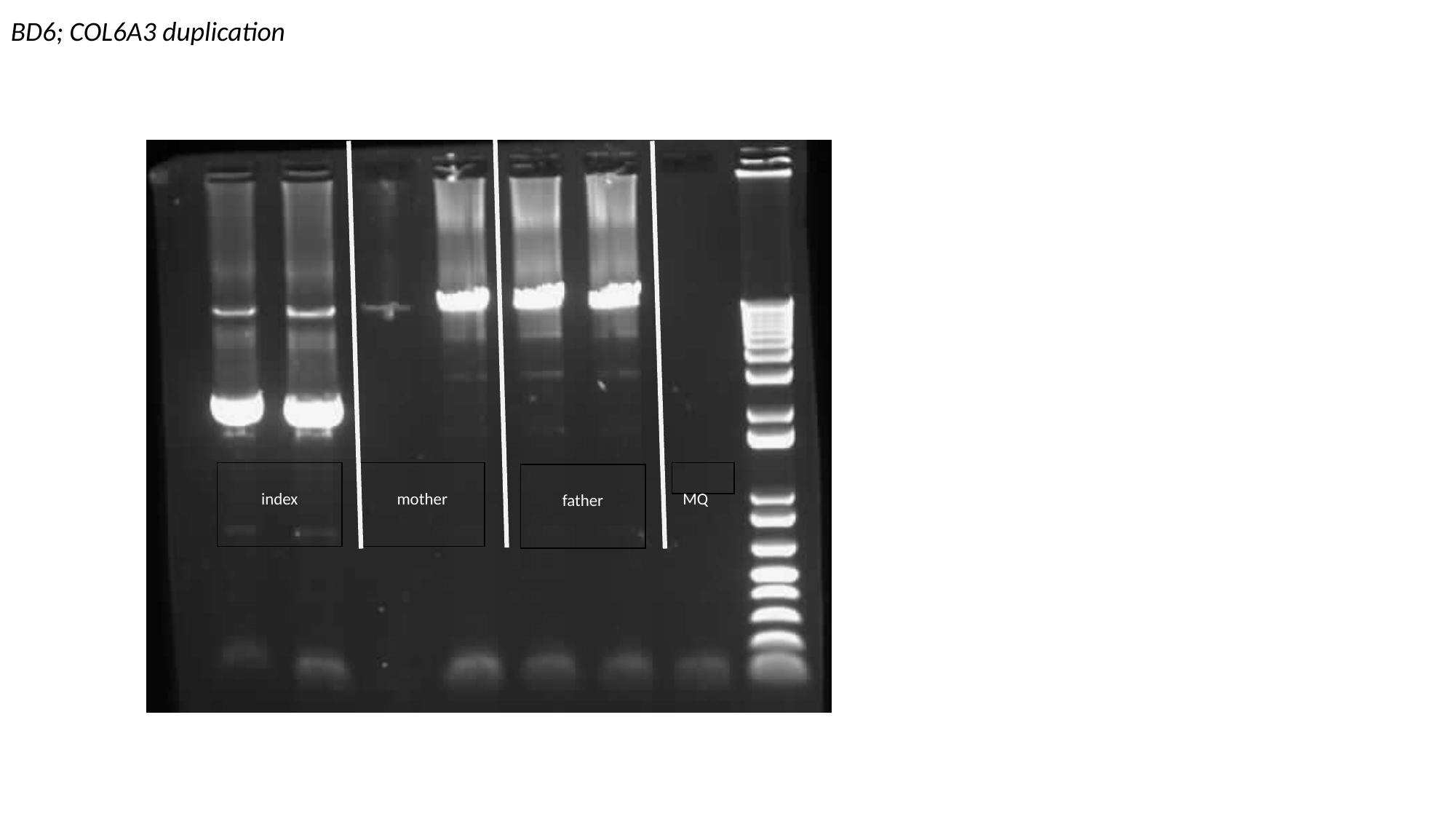

BD6; COL6A3 duplication
index
mother
MQ
father

## Slide 81
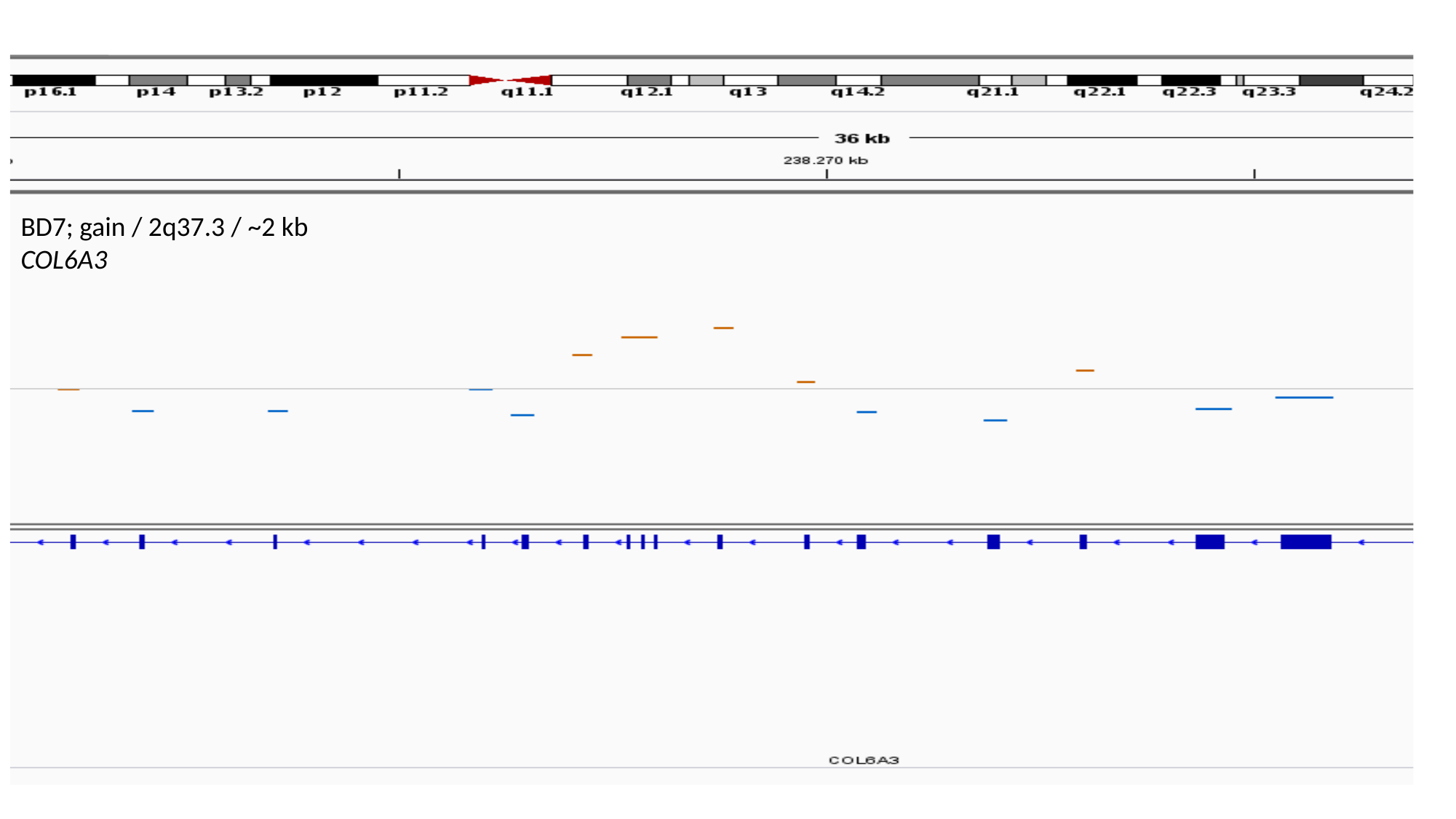

BD7; gain / 2q37.3 / ~2 kb
COL6A3

## Slide 82
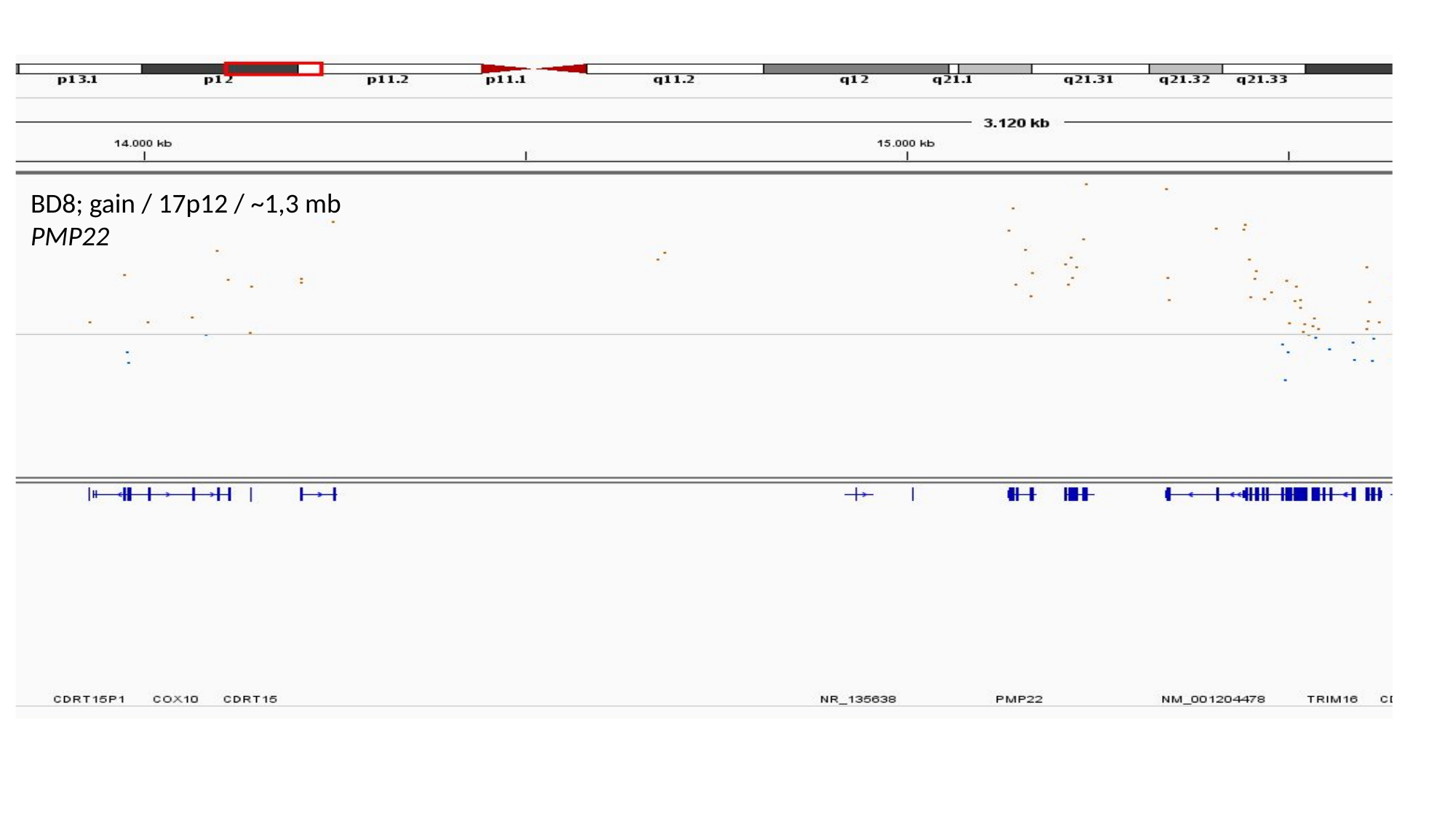

BD8; gain / 17p12 / ~1,3 mb
PMP22

## Slide 83
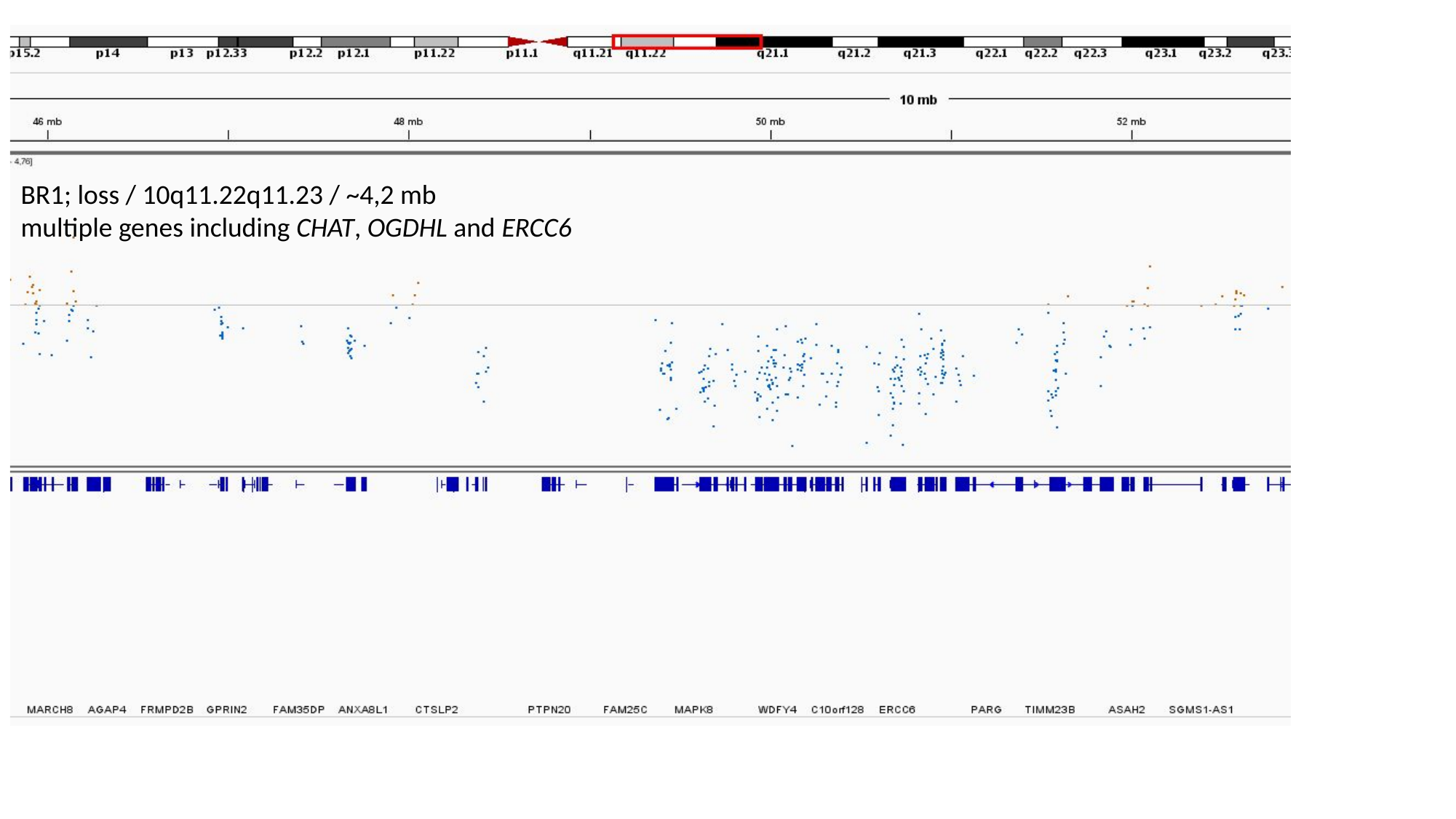

BR1; loss / 10q11.22q11.23 / ~4,2 mb
multiple genes including CHAT, OGDHL and ERCC6

## Slide 84
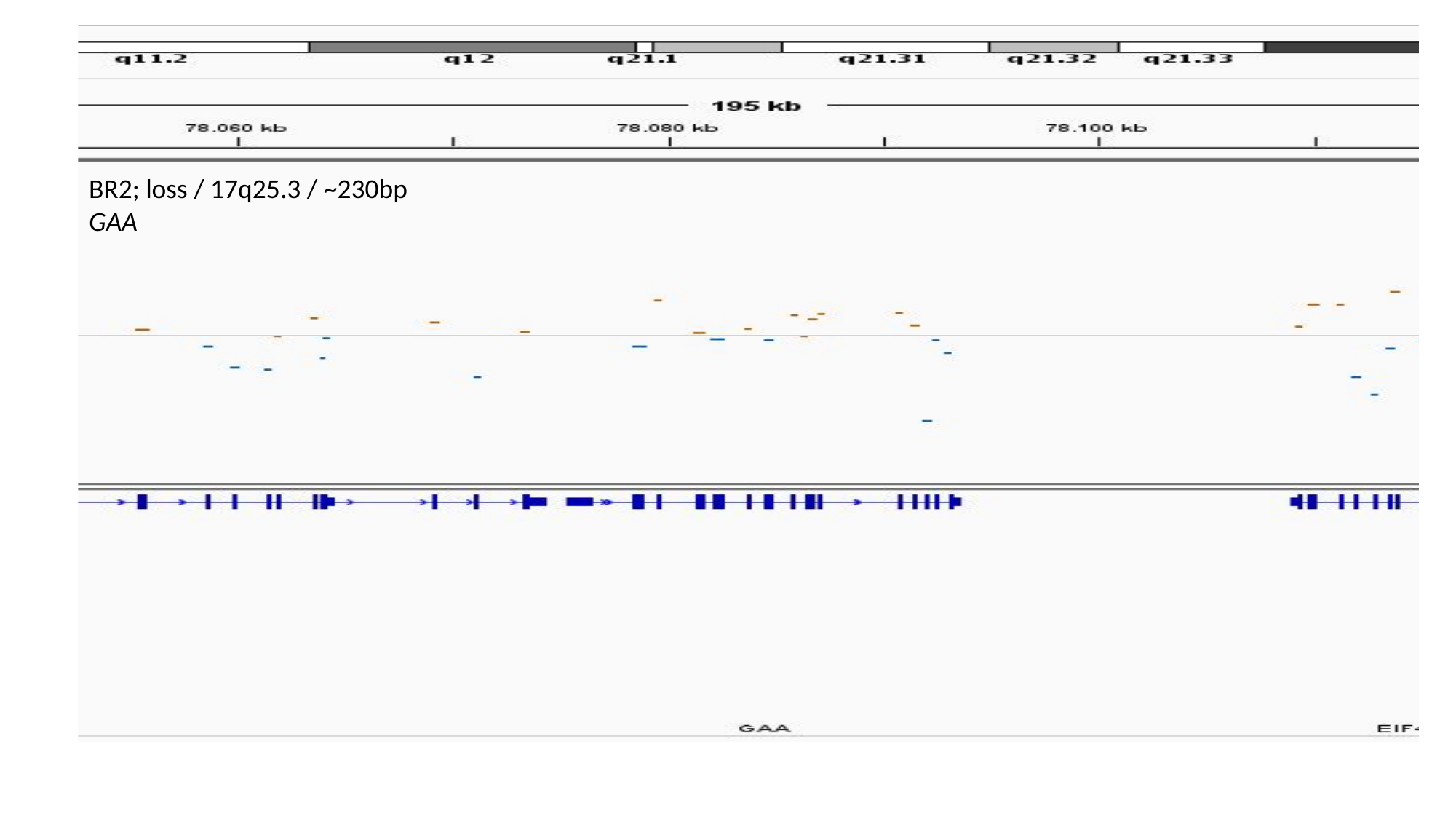

BR2; loss / 17q25.3 / ~230bp
GAA

## Slide 85
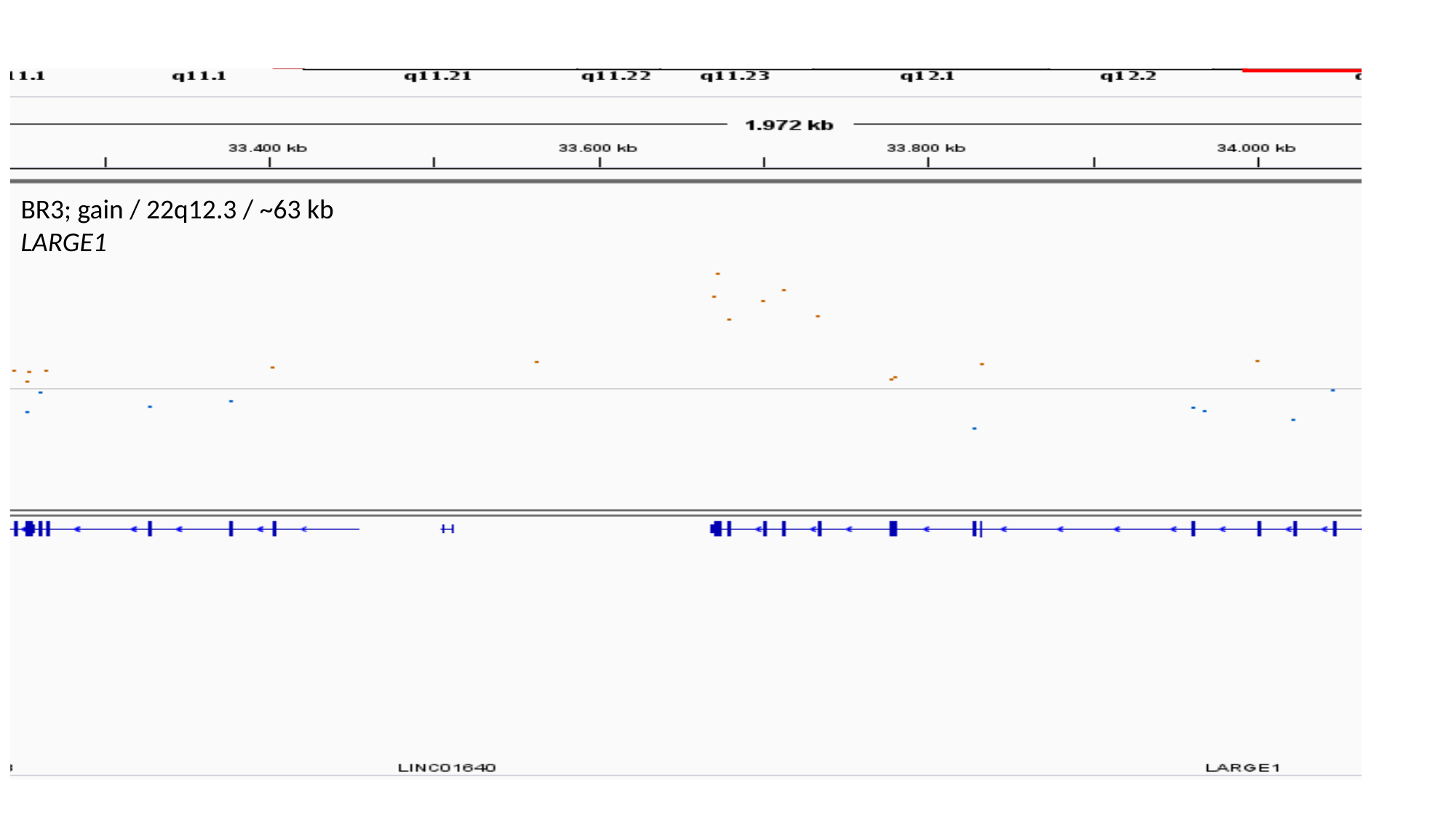

BR3; gain / 22q12.3 / ~63 kb
LARGE1

## Slide 86
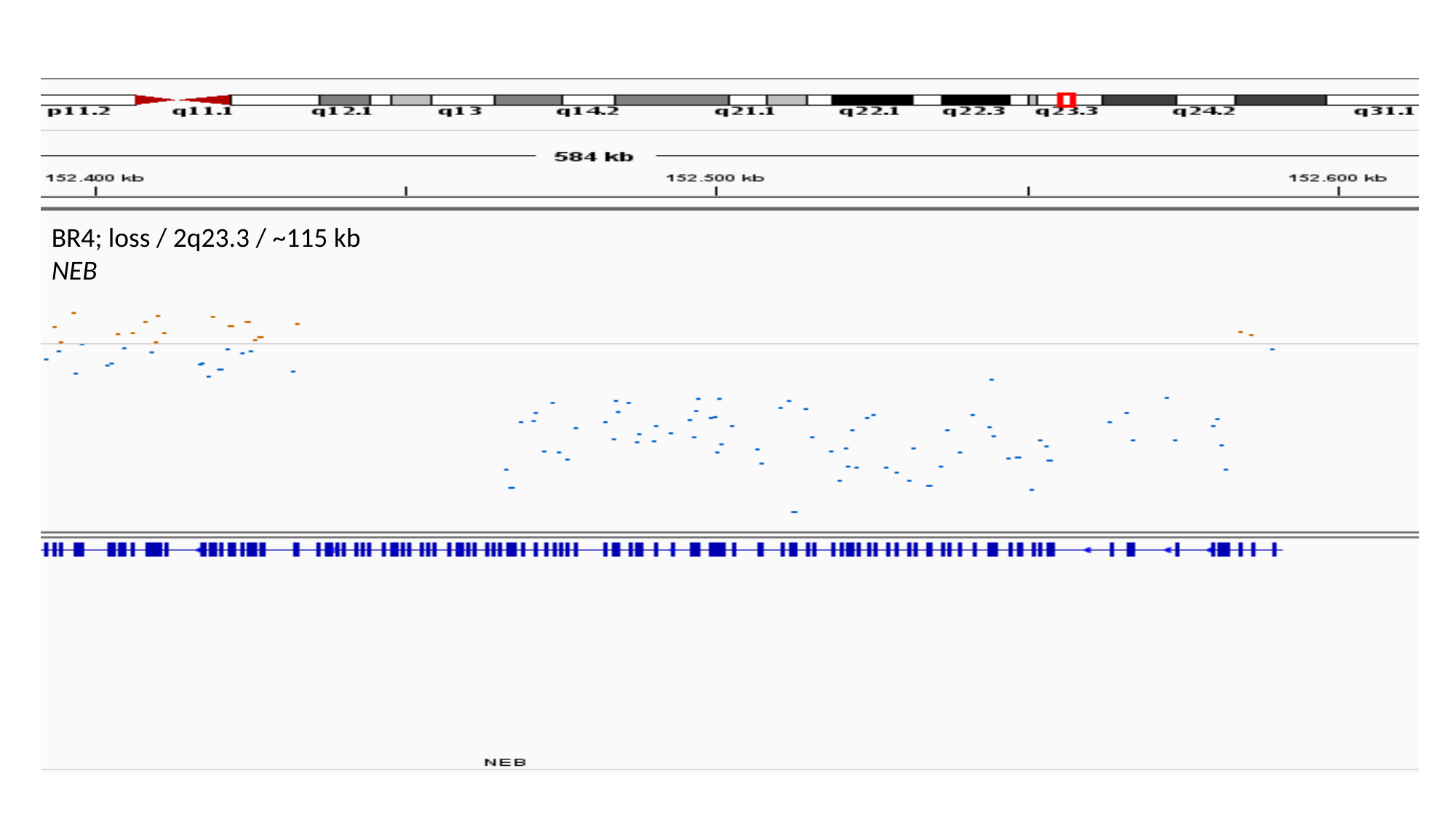

BR4; loss / 2q23.3 / ~115 kb
NEB

## Slide 87
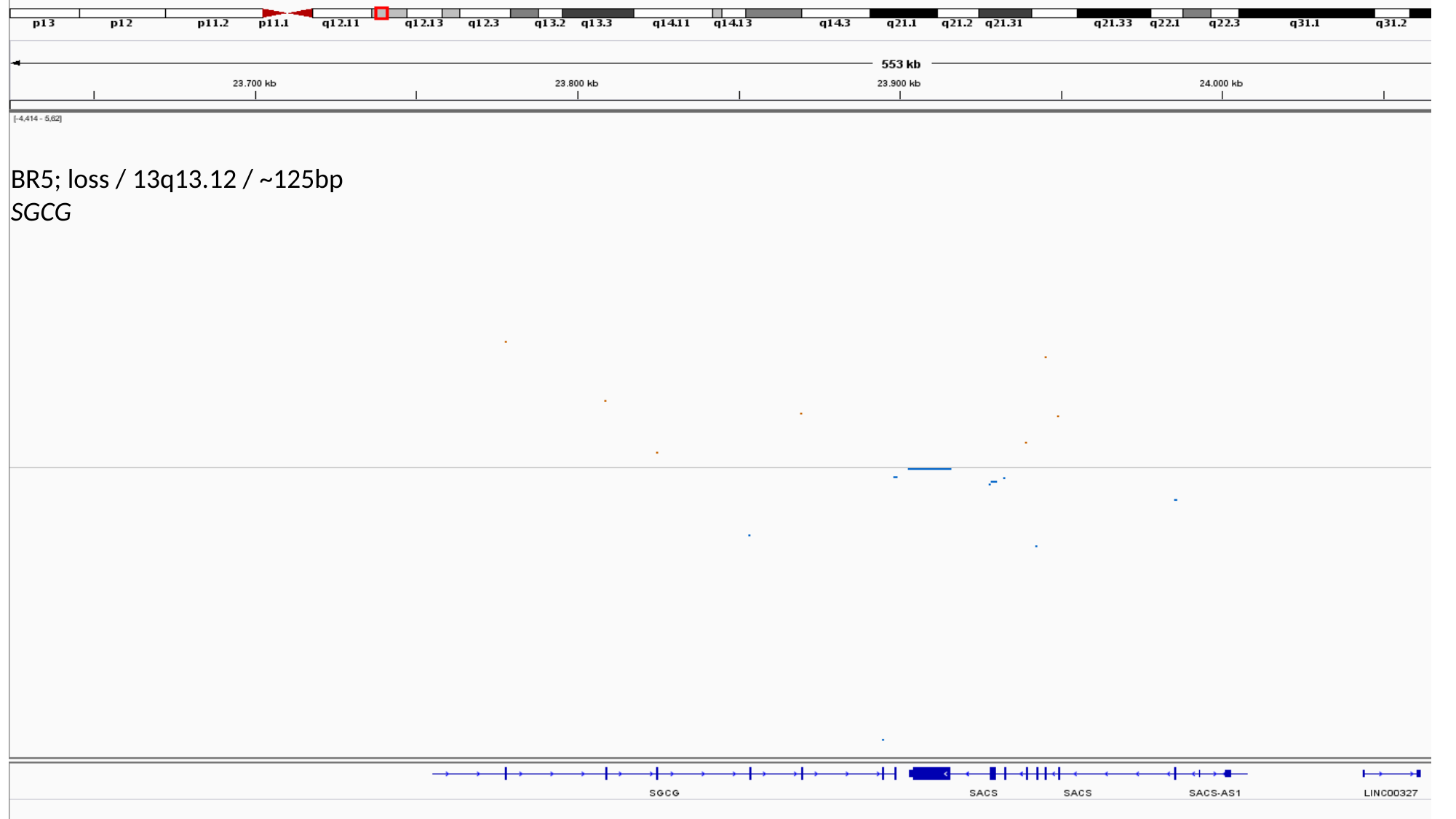

BR5; loss / 13q13.12 / ~125bp
SGCG

## Slide 88
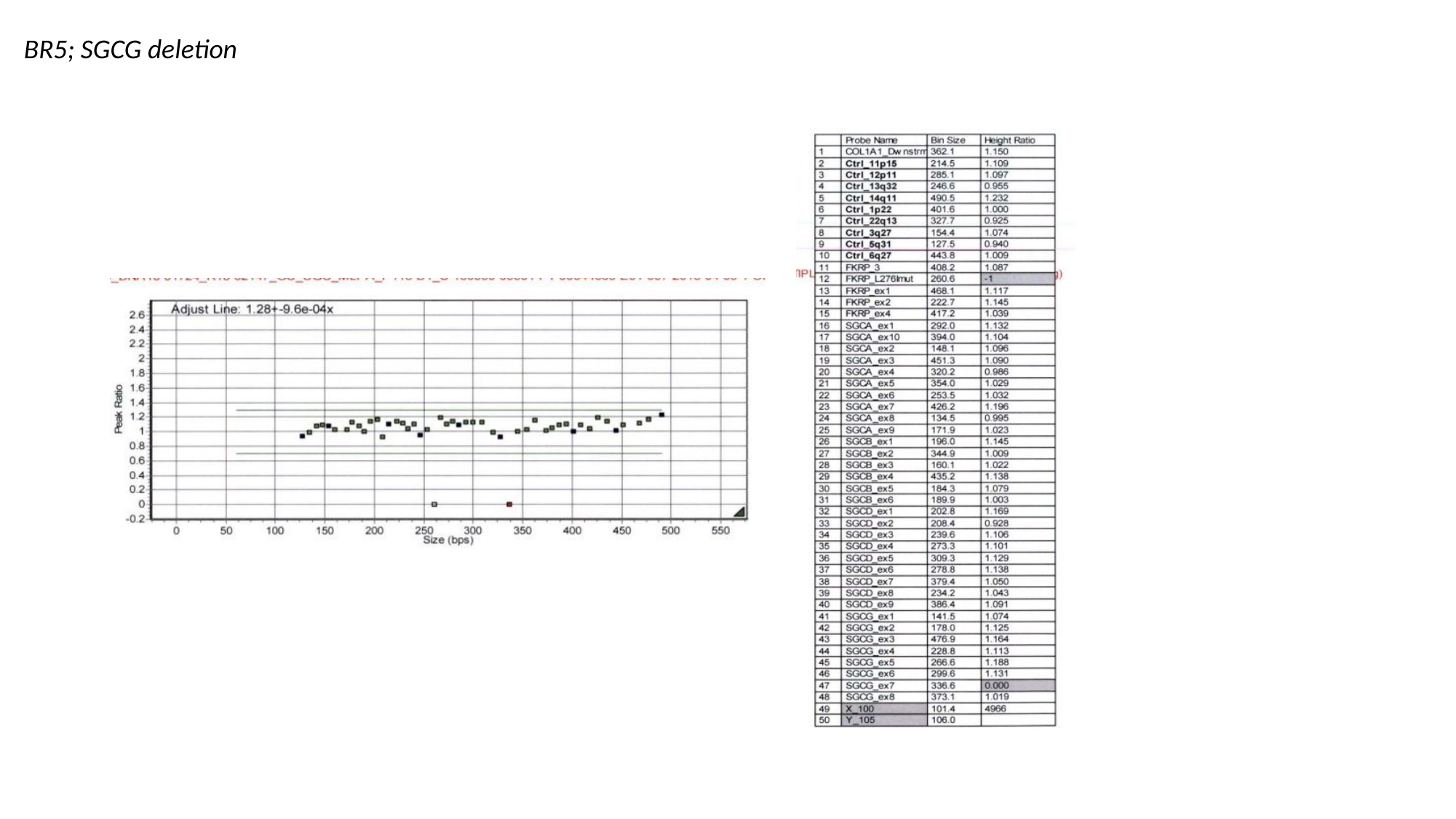

BR5; SGCG deletion

## Slide 89
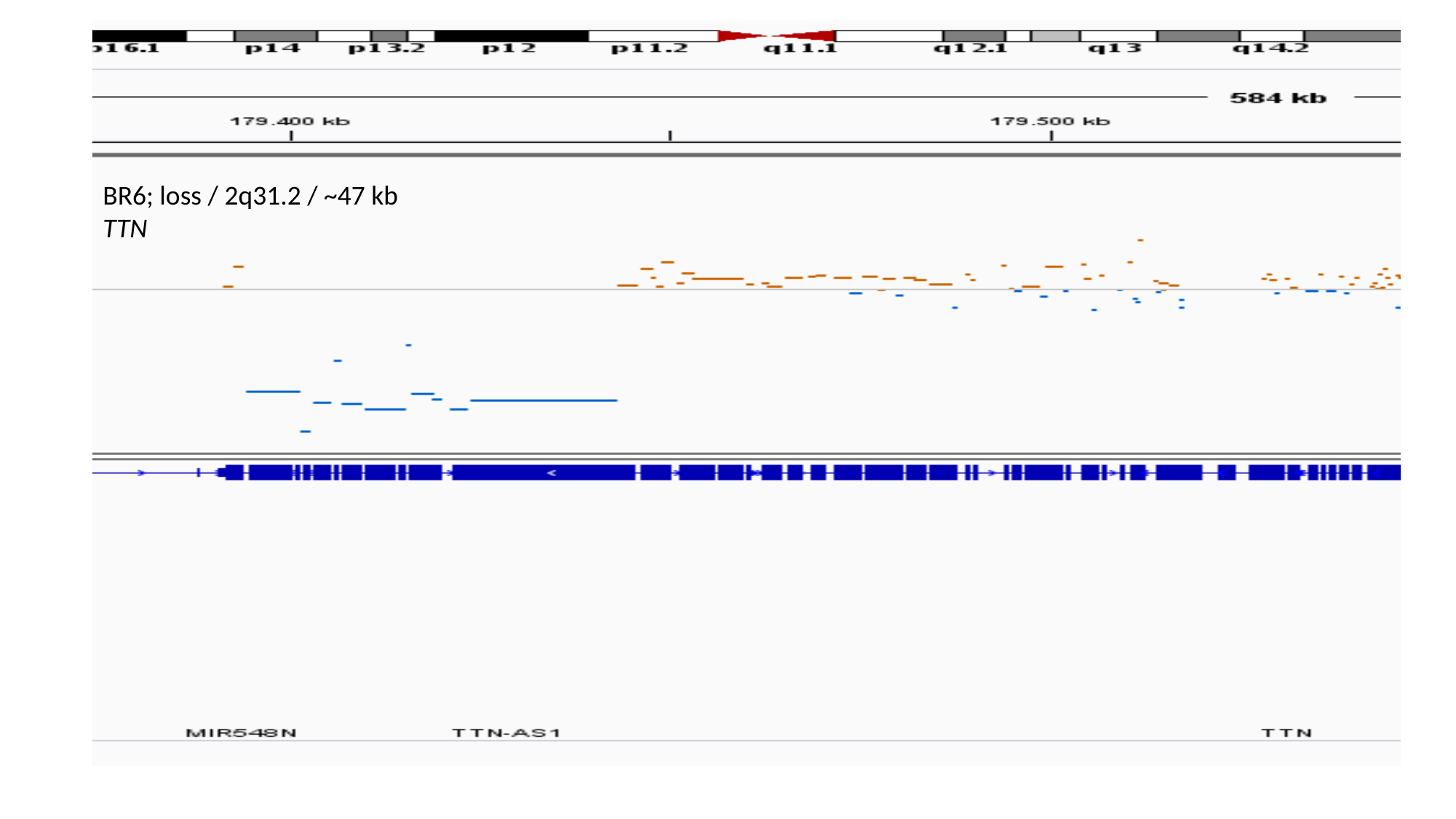

BR6; loss / 2q31.2 / ~47 kb
TTN

## Slide 90
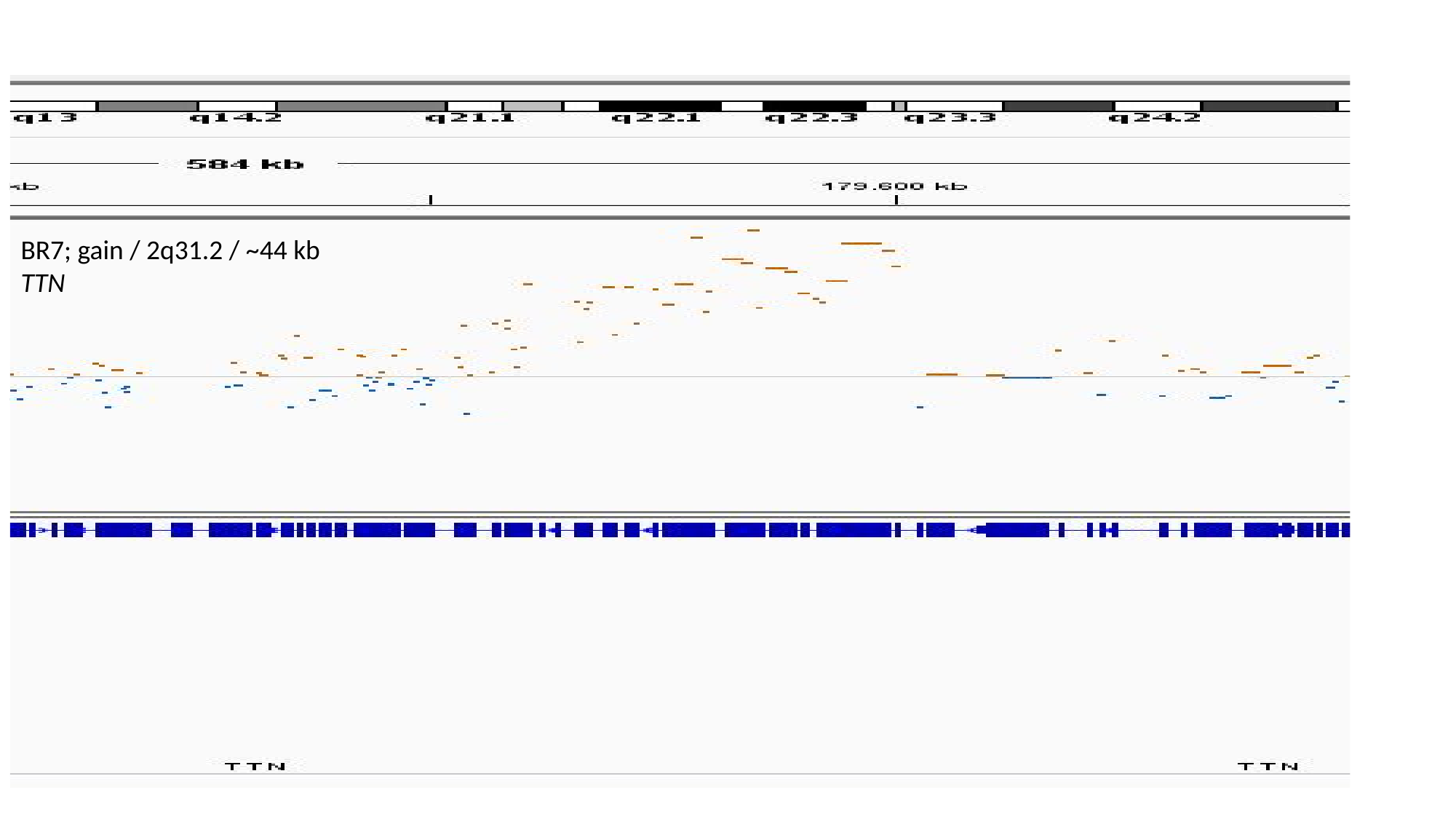

BR7; gain / 2q31.2 / ~44 kb
TTN

## Slide 91
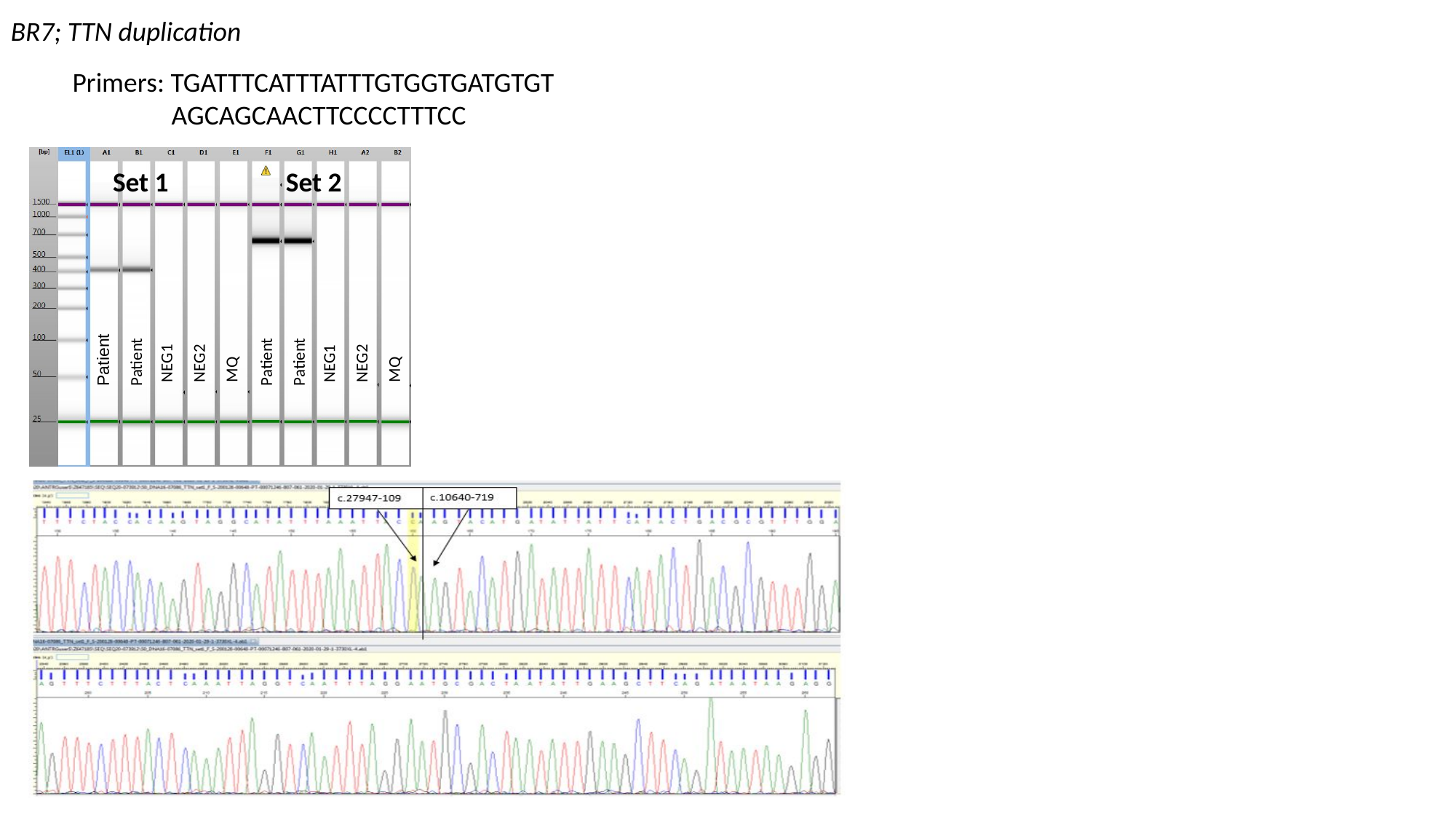

BR7; TTN duplication
Primers: TGATTTCATTTATTTGTGGTGATGTGT
 AGCAGCAACTTCCCCTTTCC
Set 1
Set 2
Patient
Patient
Patient
Patient
NEG1
NEG2
MQ
NEG2
MQ
NEG1

## Slide 92
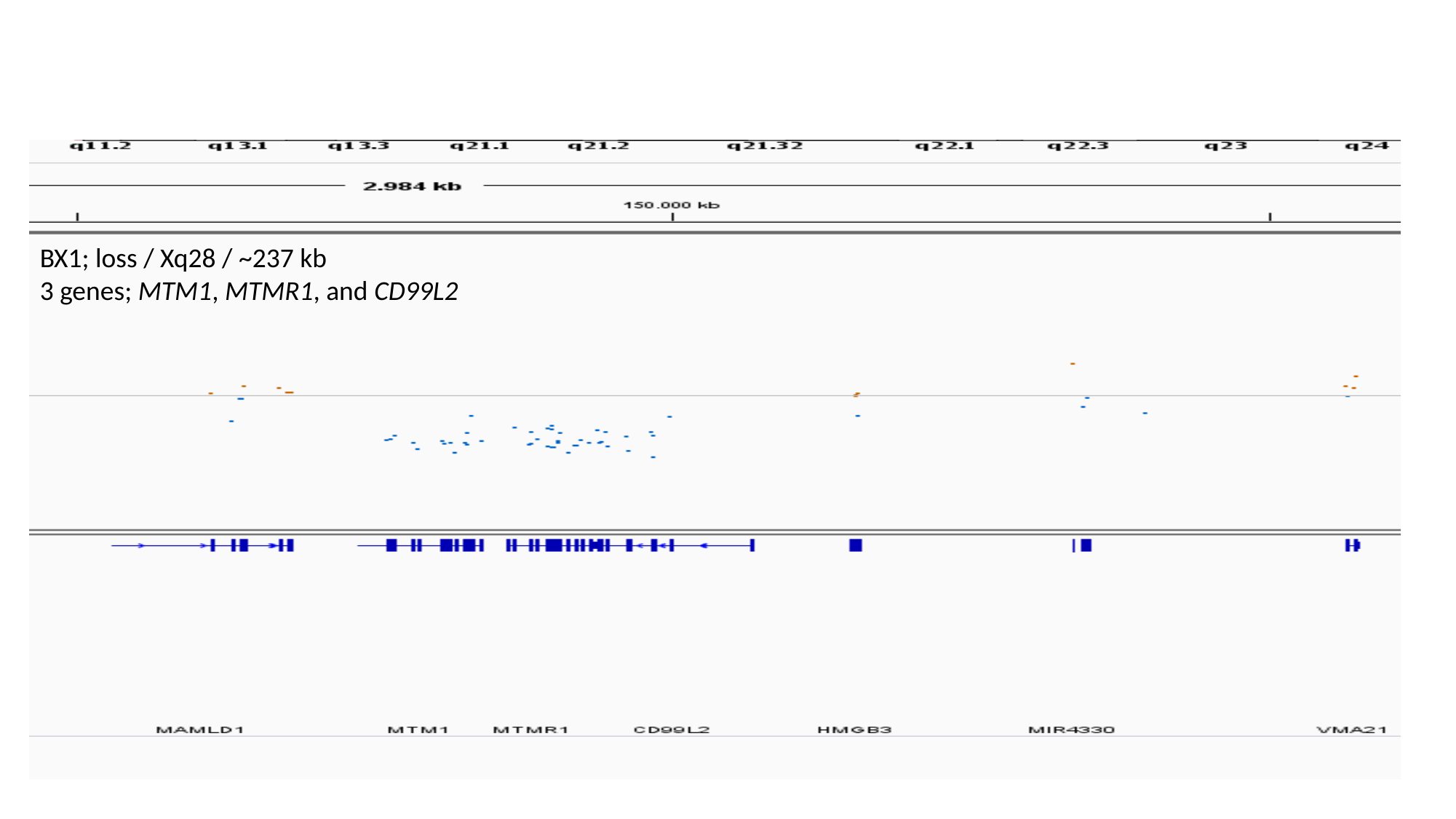

BX1; loss / Xq28 / ~237 kb
3 genes; MTM1, MTMR1, and CD99L2

## Slide 93
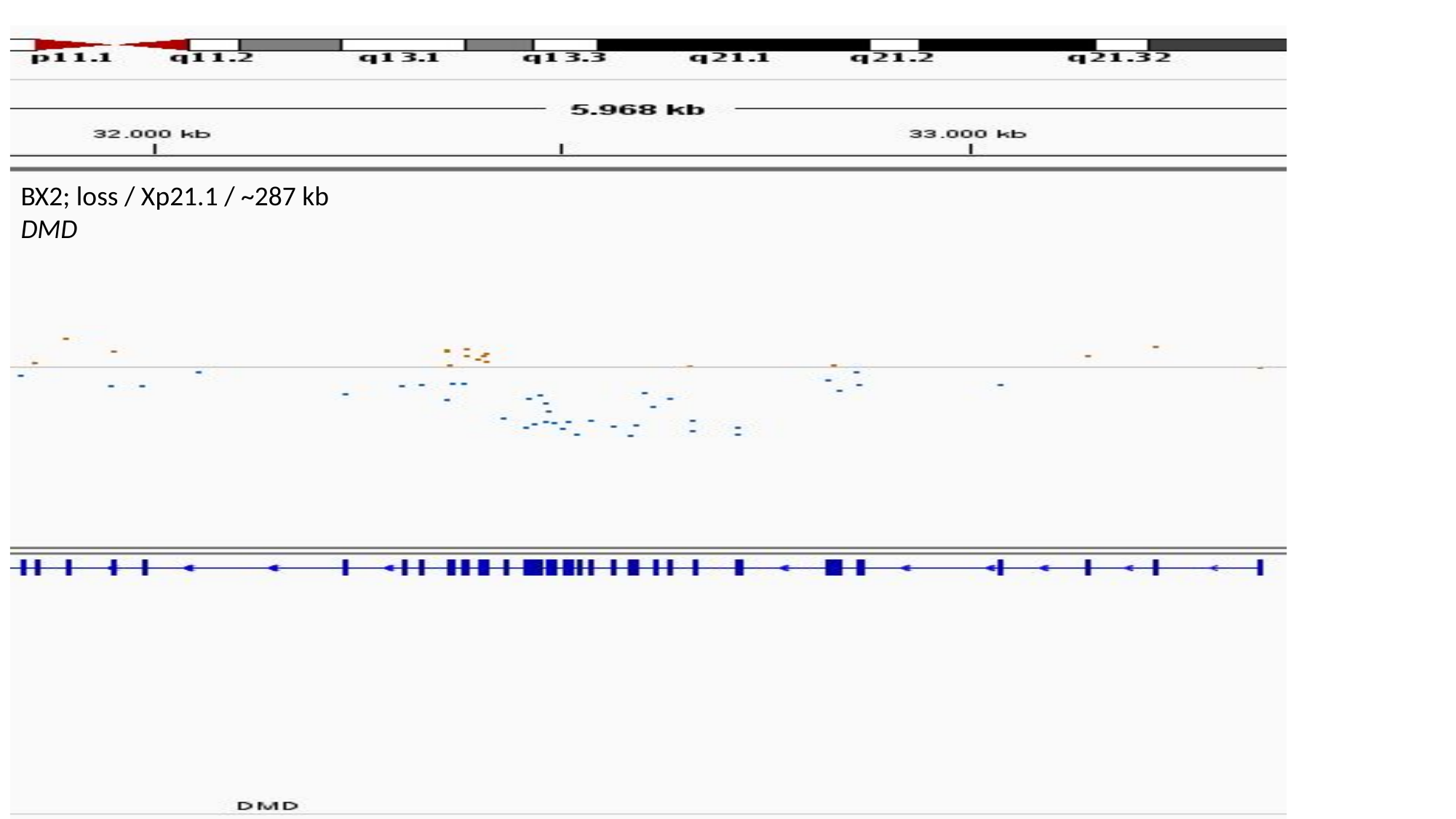

BX2; loss / Xp21.1 / ~287 kb
DMD

## Slide 94
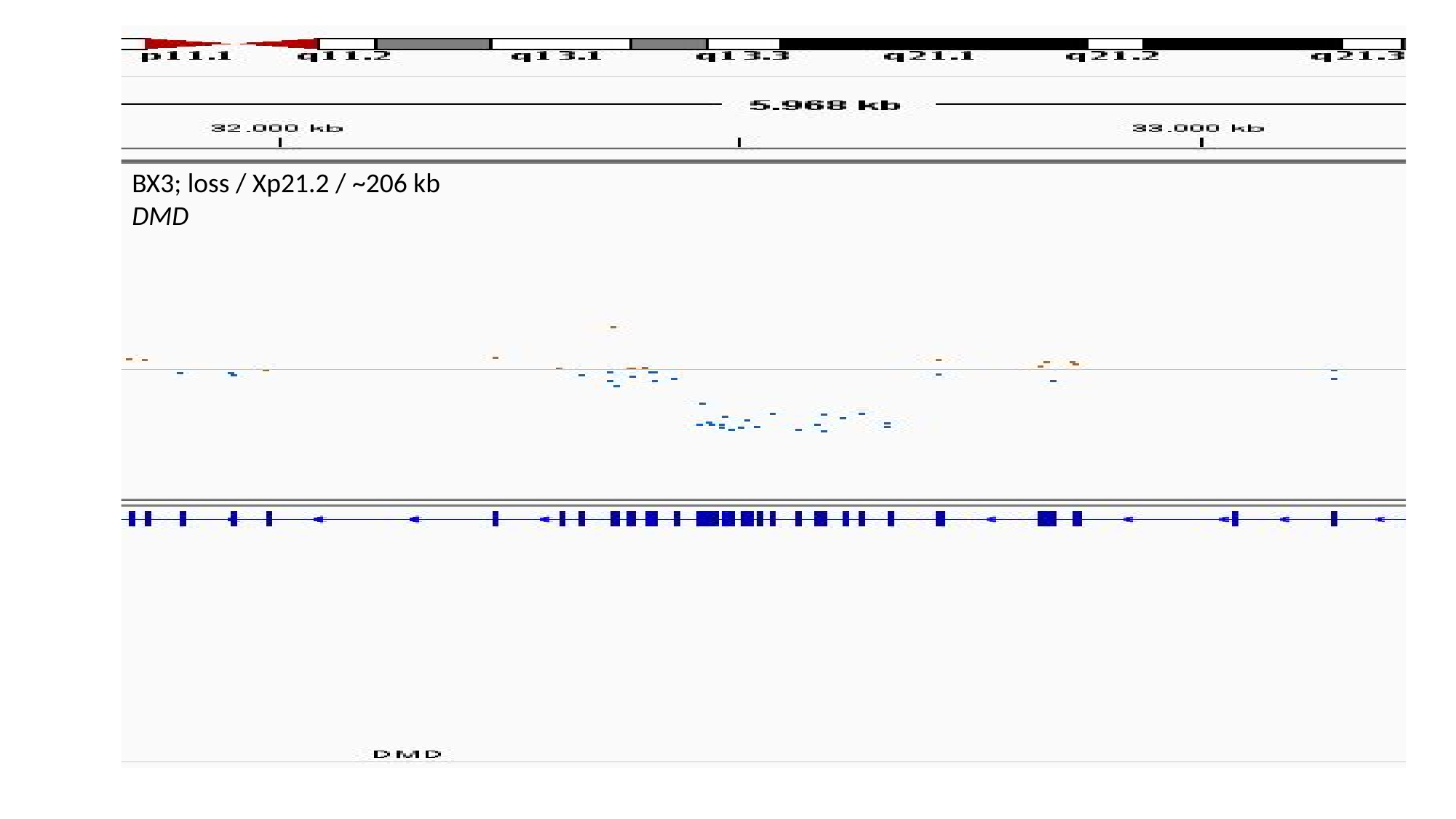

BX3; loss / Xp21.2 / ~206 kb
DMD

## Slide 95
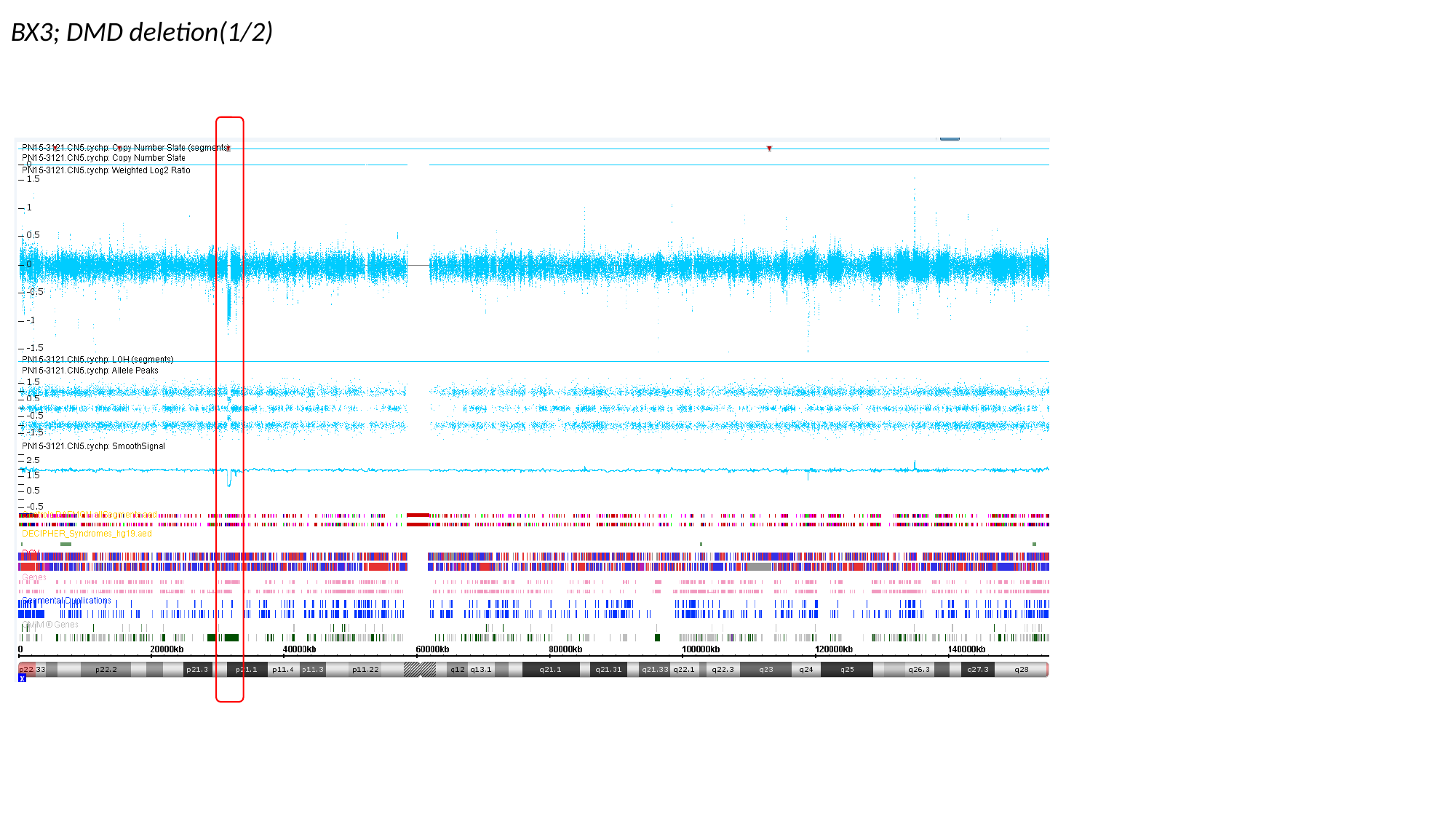

BX3; DMD deletion(1/2)

## Slide 96
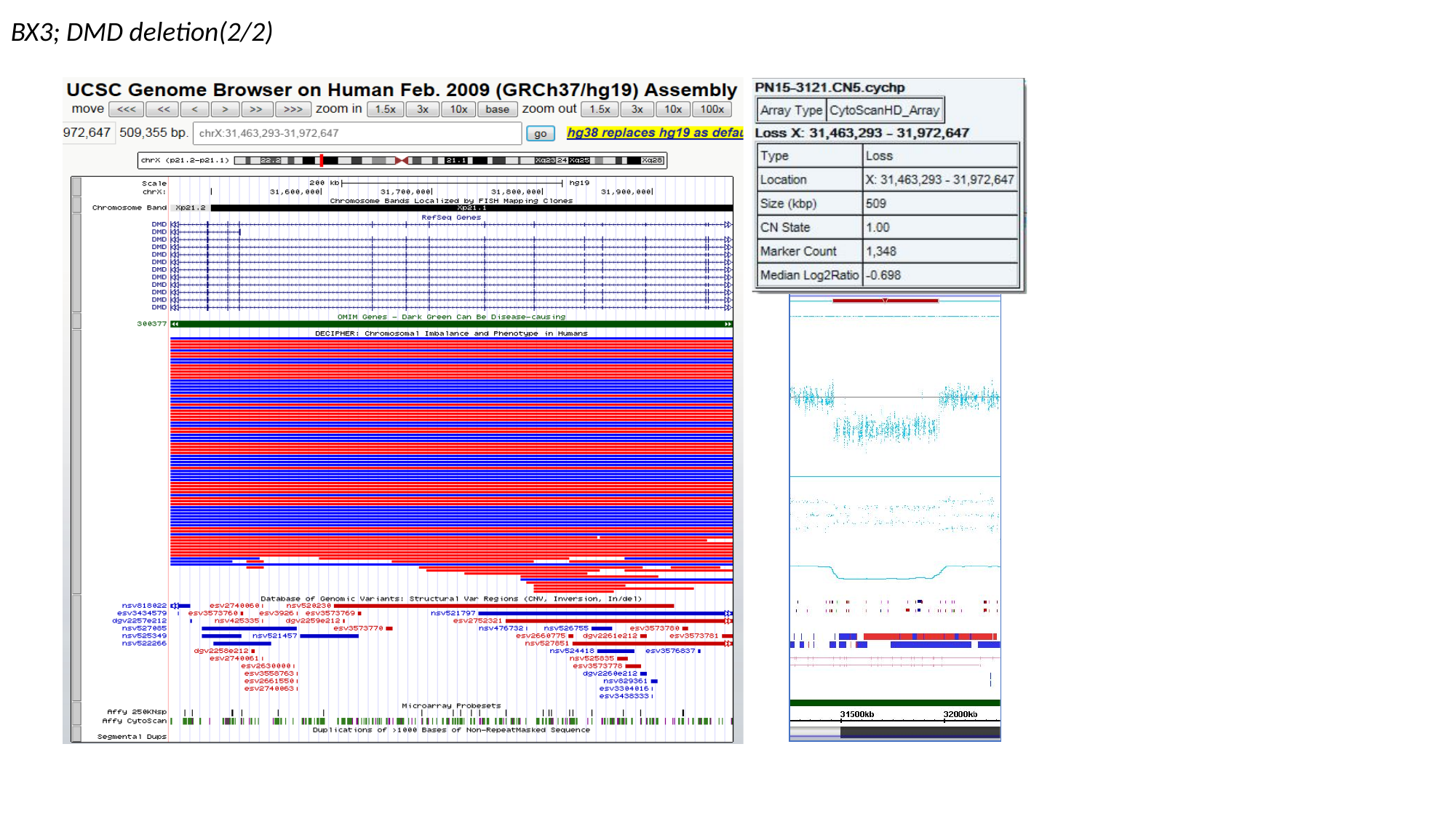

BX3; DMD deletion(2/2)

## Slide 97
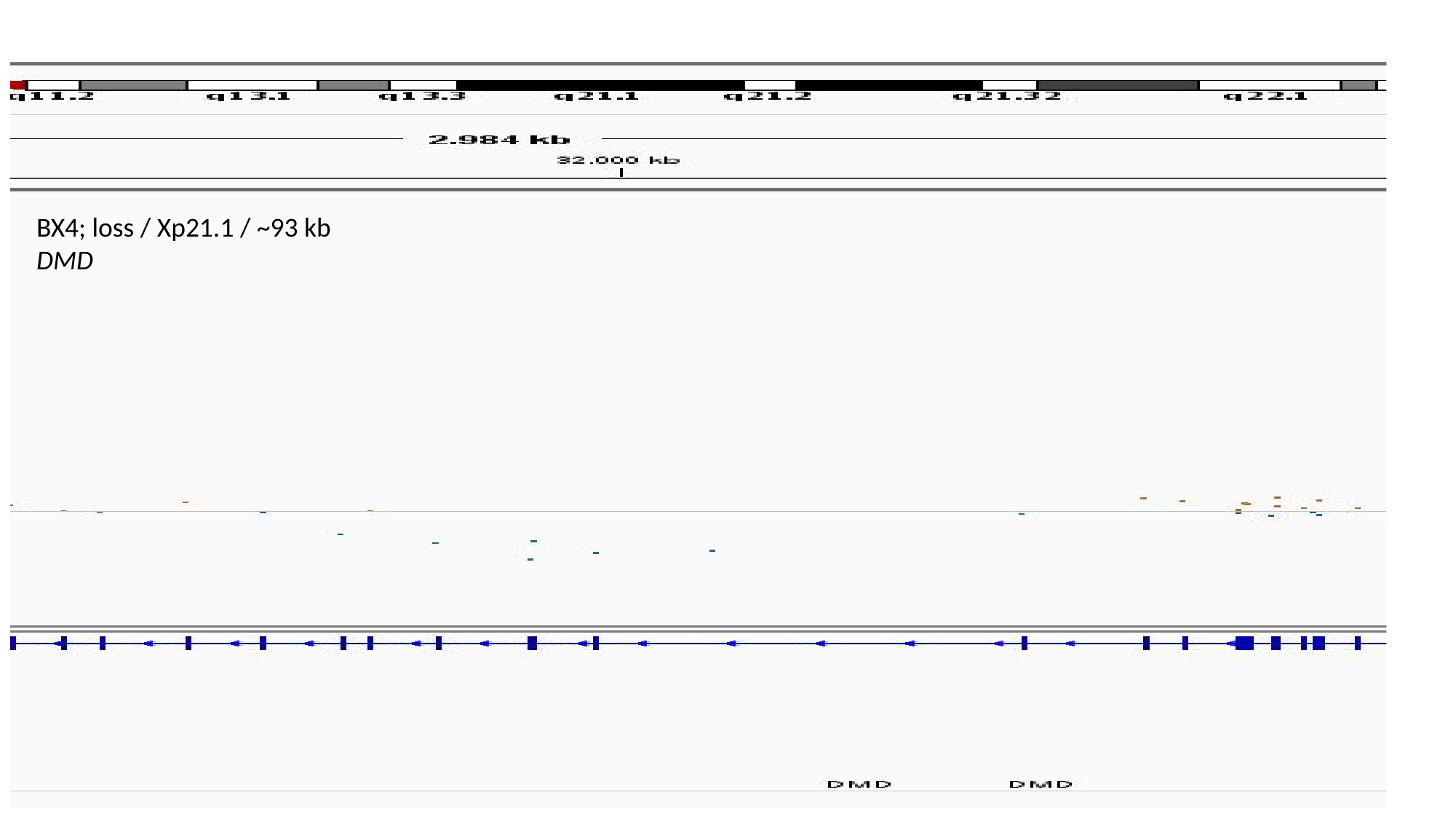

BX4; loss / Xp21.1 / ~93 kb
DMD

## Slide 98
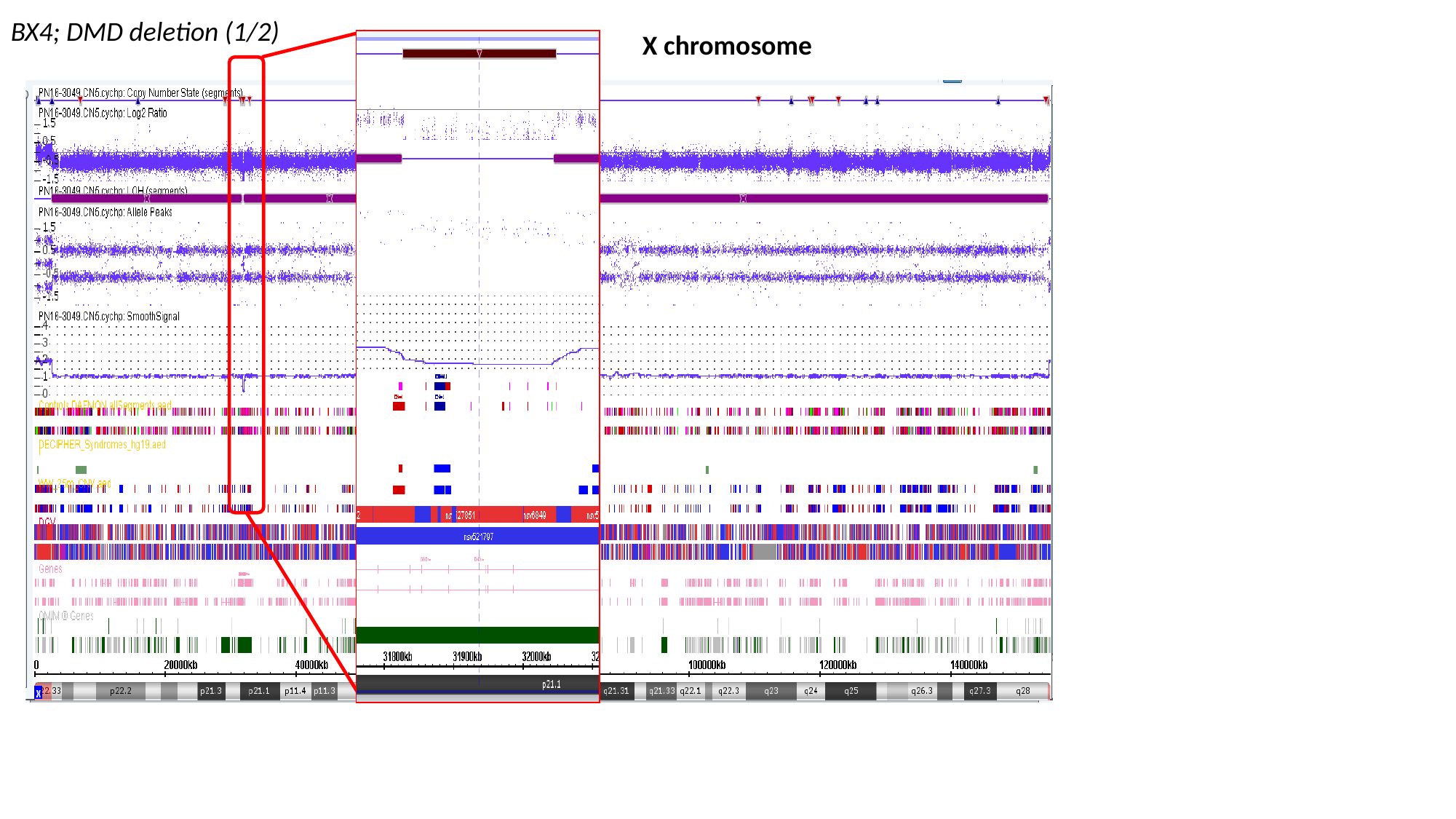

BX4; DMD deletion (1/2)
X chromosome

## Slide 99
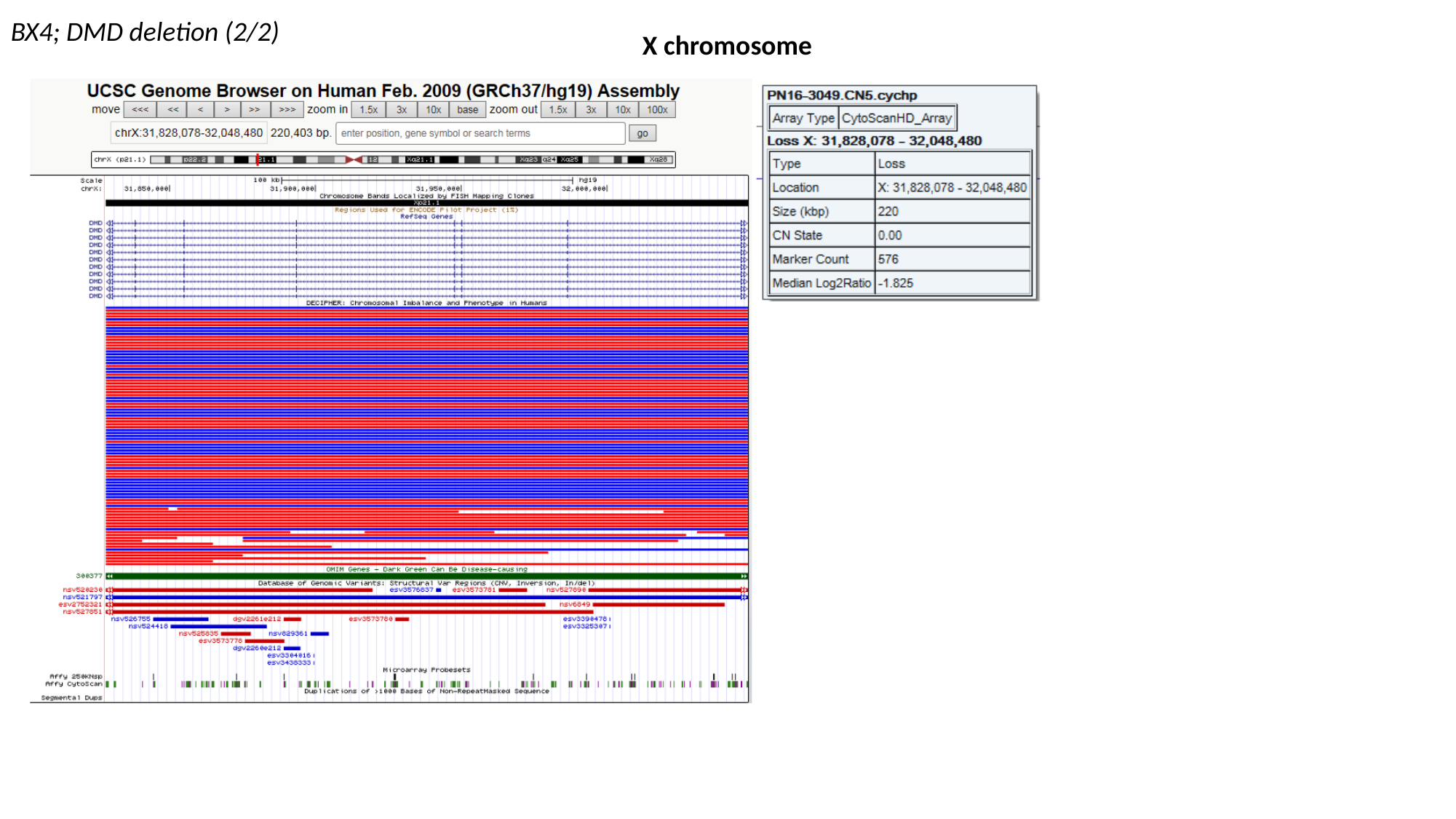

BX4; DMD deletion (2/2)
X chromosome

## Slide 100
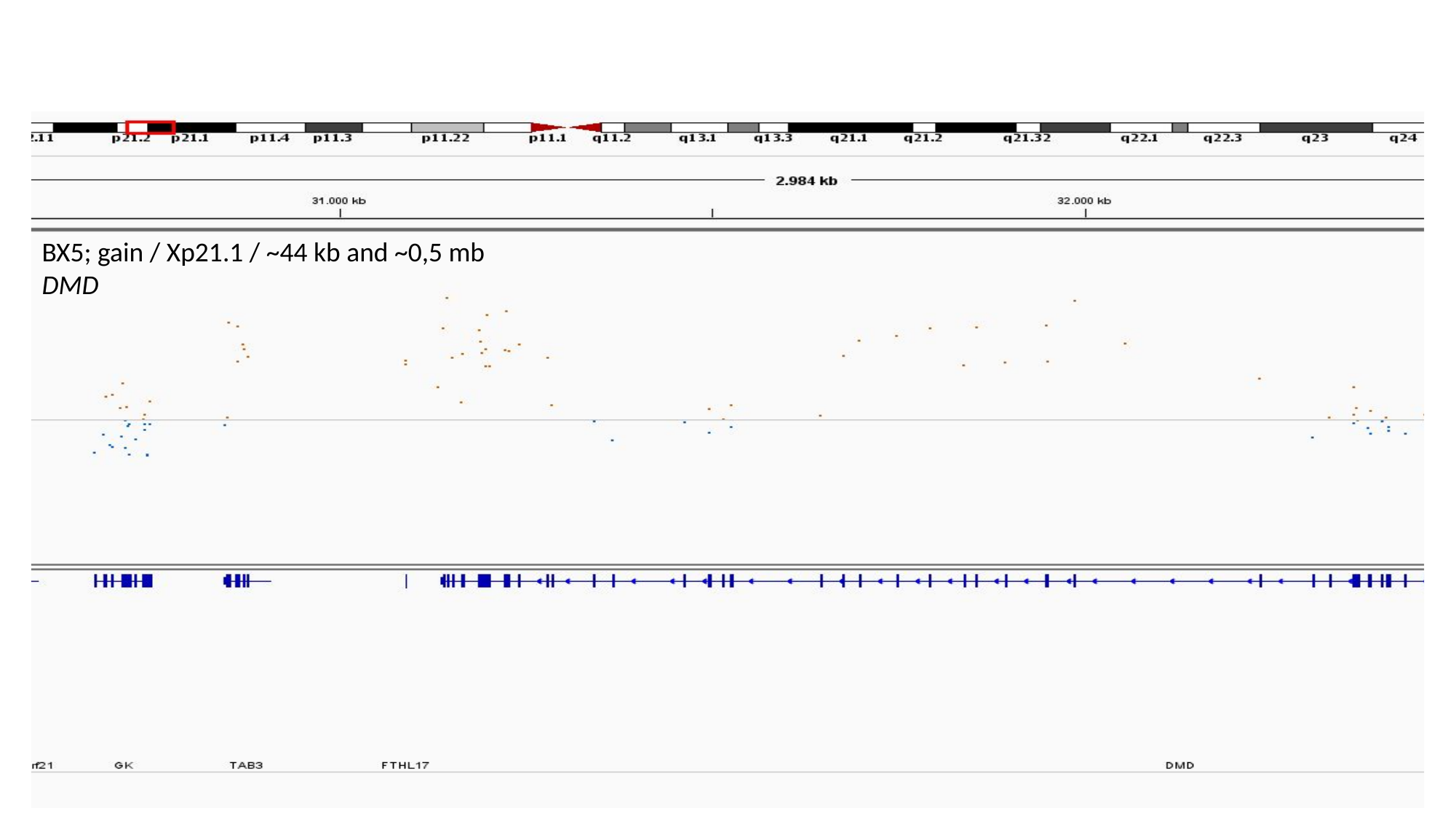

BX5; gain / Xp21.1 / ~44 kb and ~0,5 mb
DMD

## Slide 101
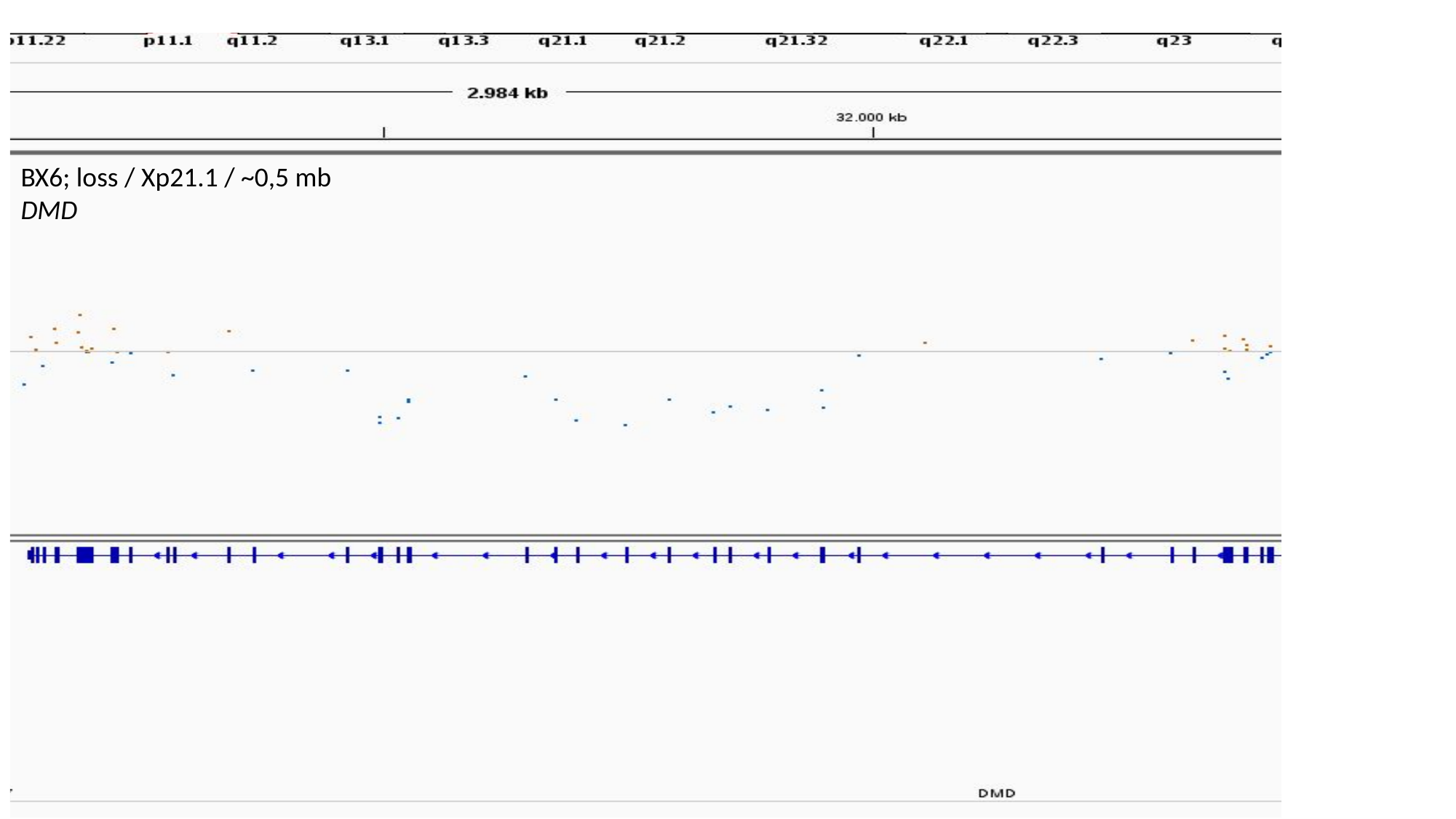

BX6; loss / Xp21.1 / ~0,5 mb
DMD

## Slide 102
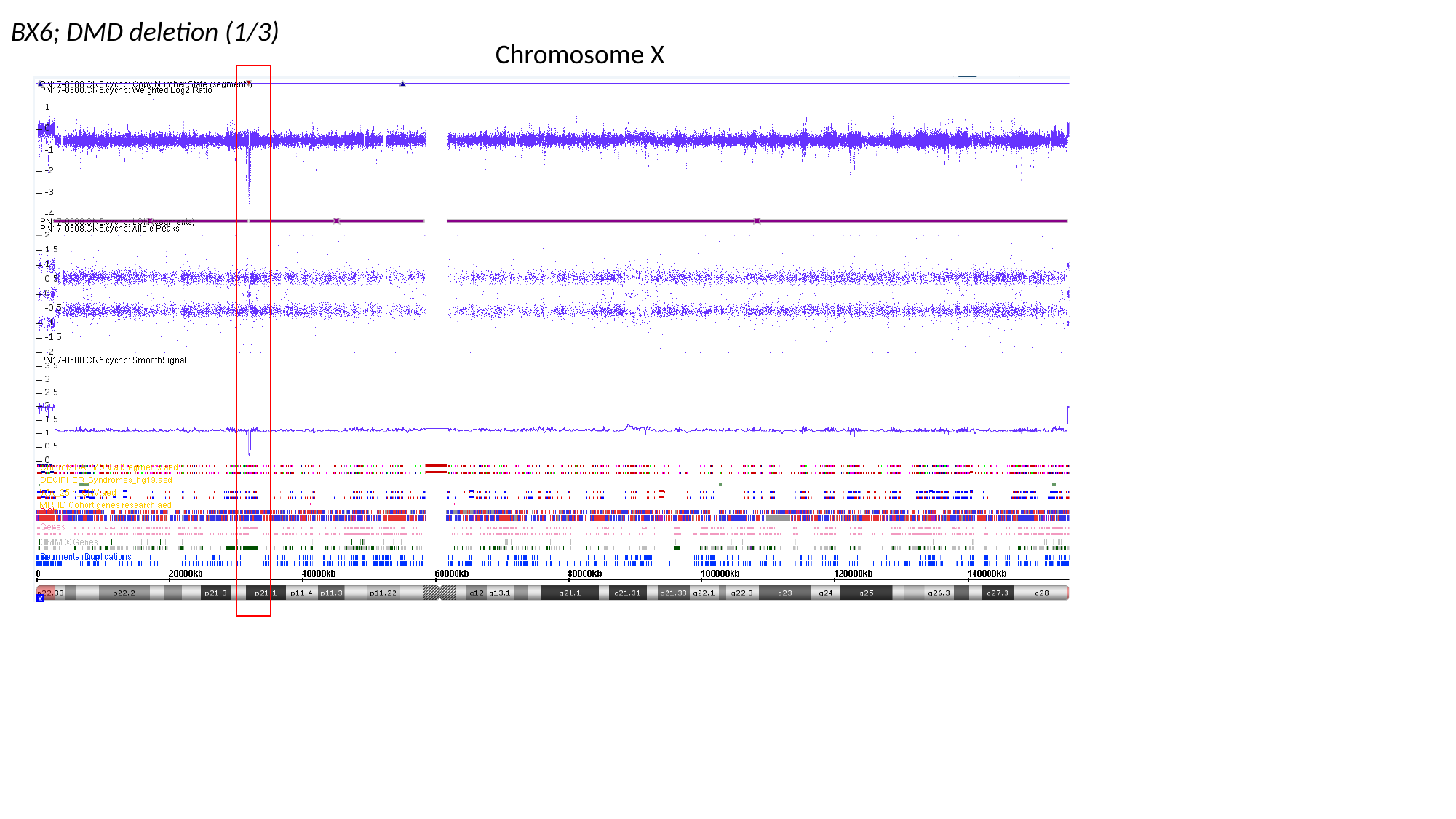

BX6; DMD deletion (1/3)
Chromosome X

## Slide 103
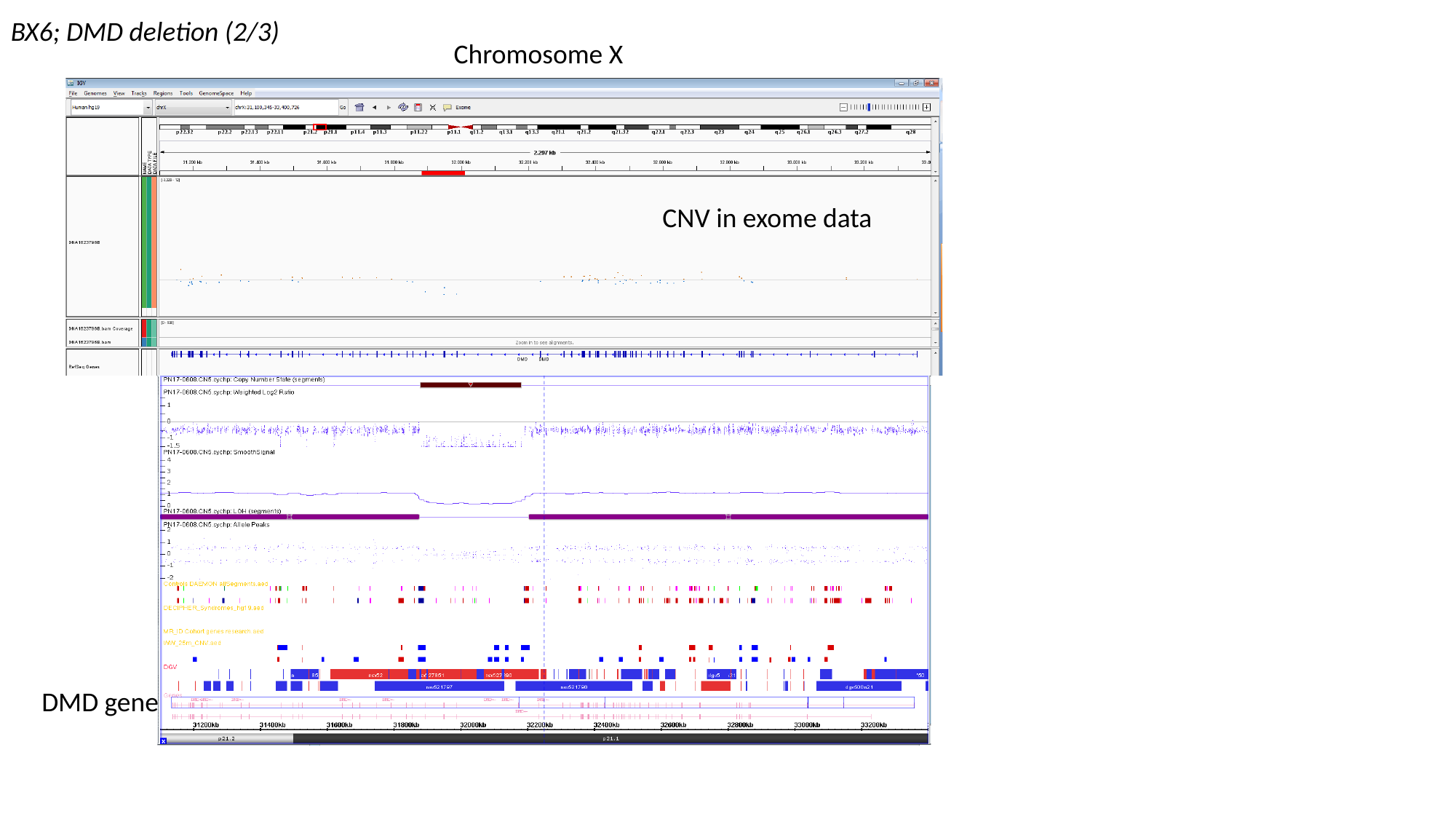

BX6; DMD deletion (2/3)
Chromosome X
CNV in exome data
DMD gene

## Slide 104
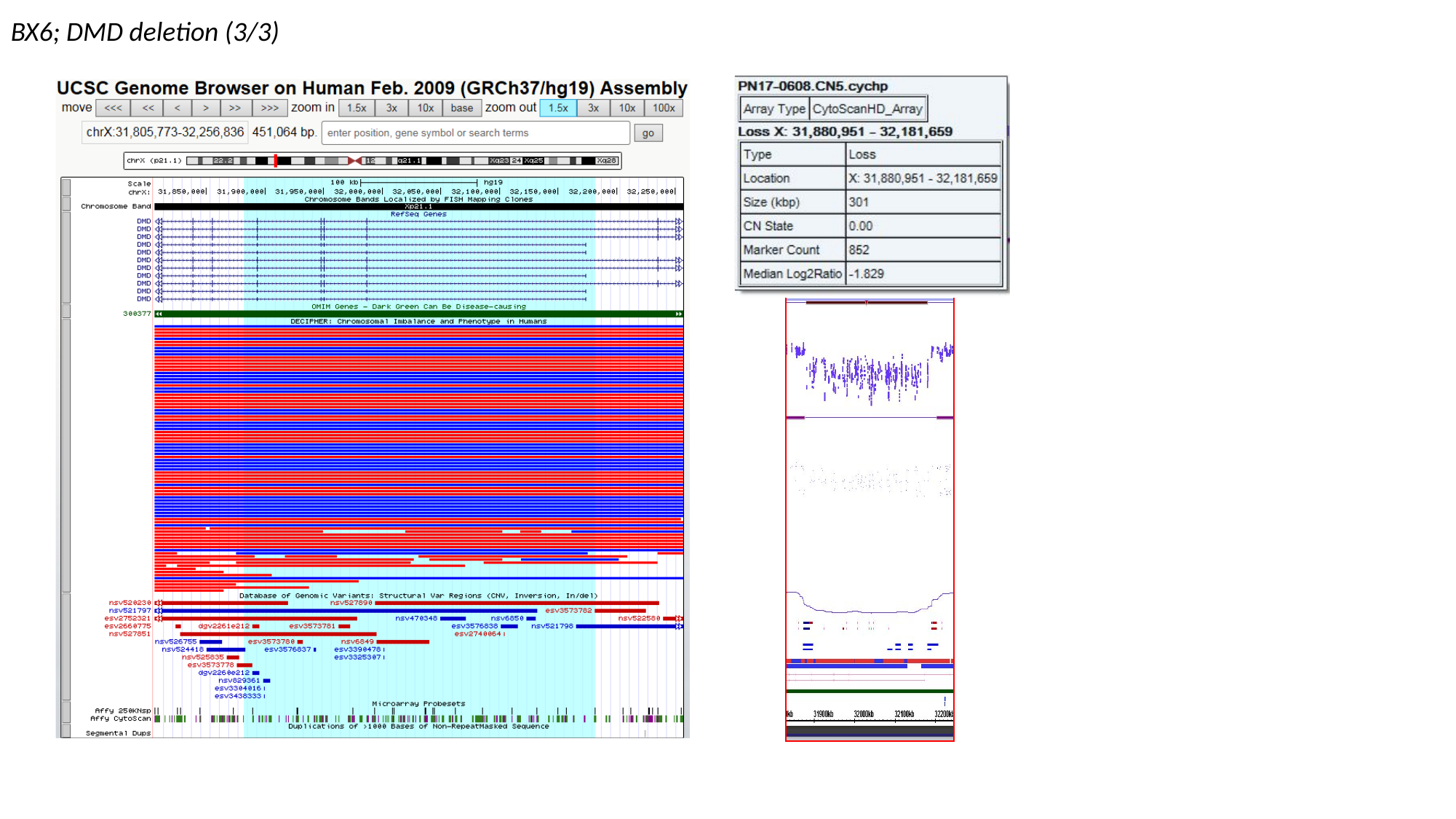

BX6; DMD deletion (3/3)

## Slide 105
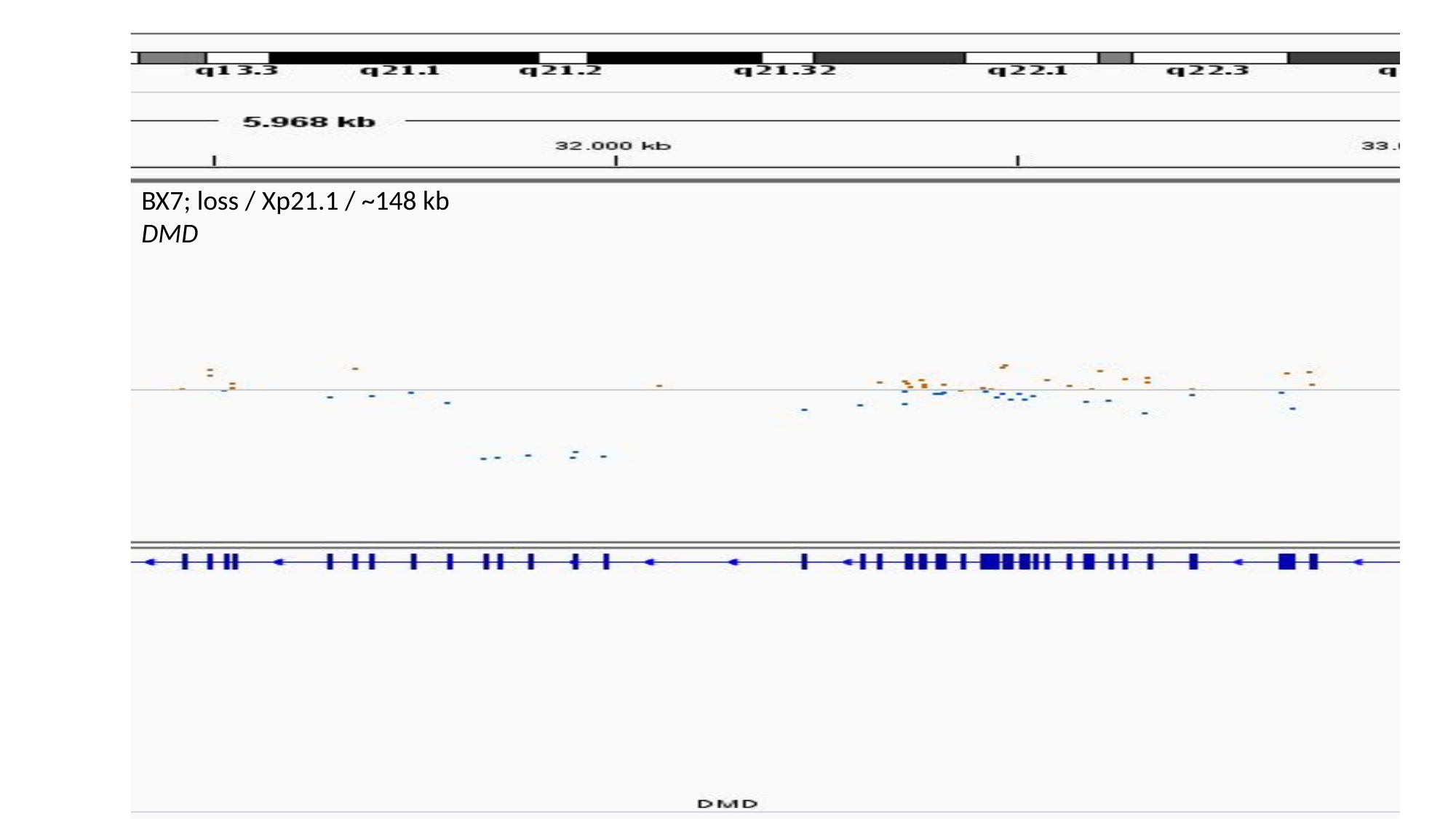

BX7; loss / Xp21.1 / ~148 kb
DMD

## Slide 106
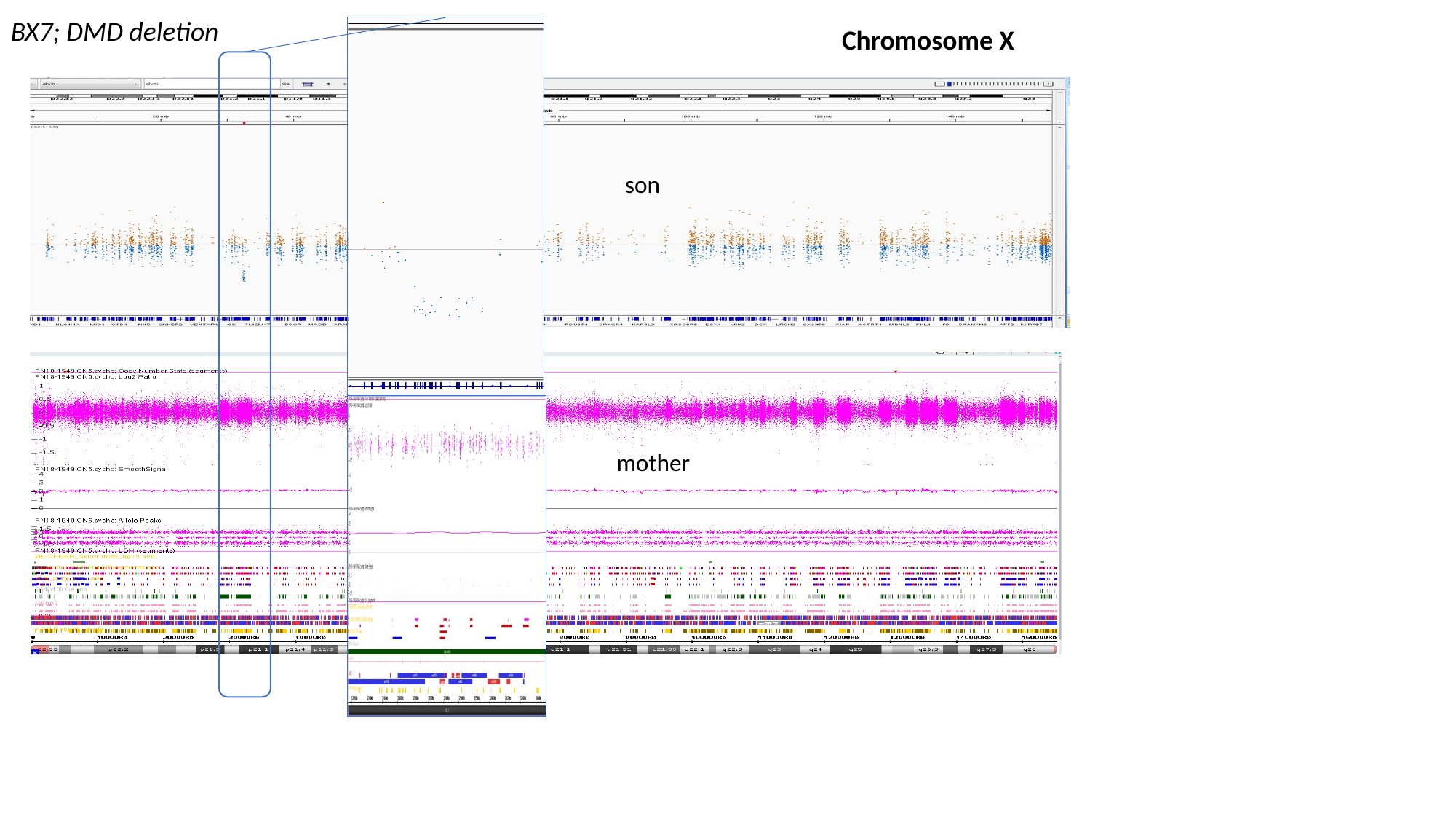

BX7; DMD deletion
Chromosome X
son
mother

## Slide 107
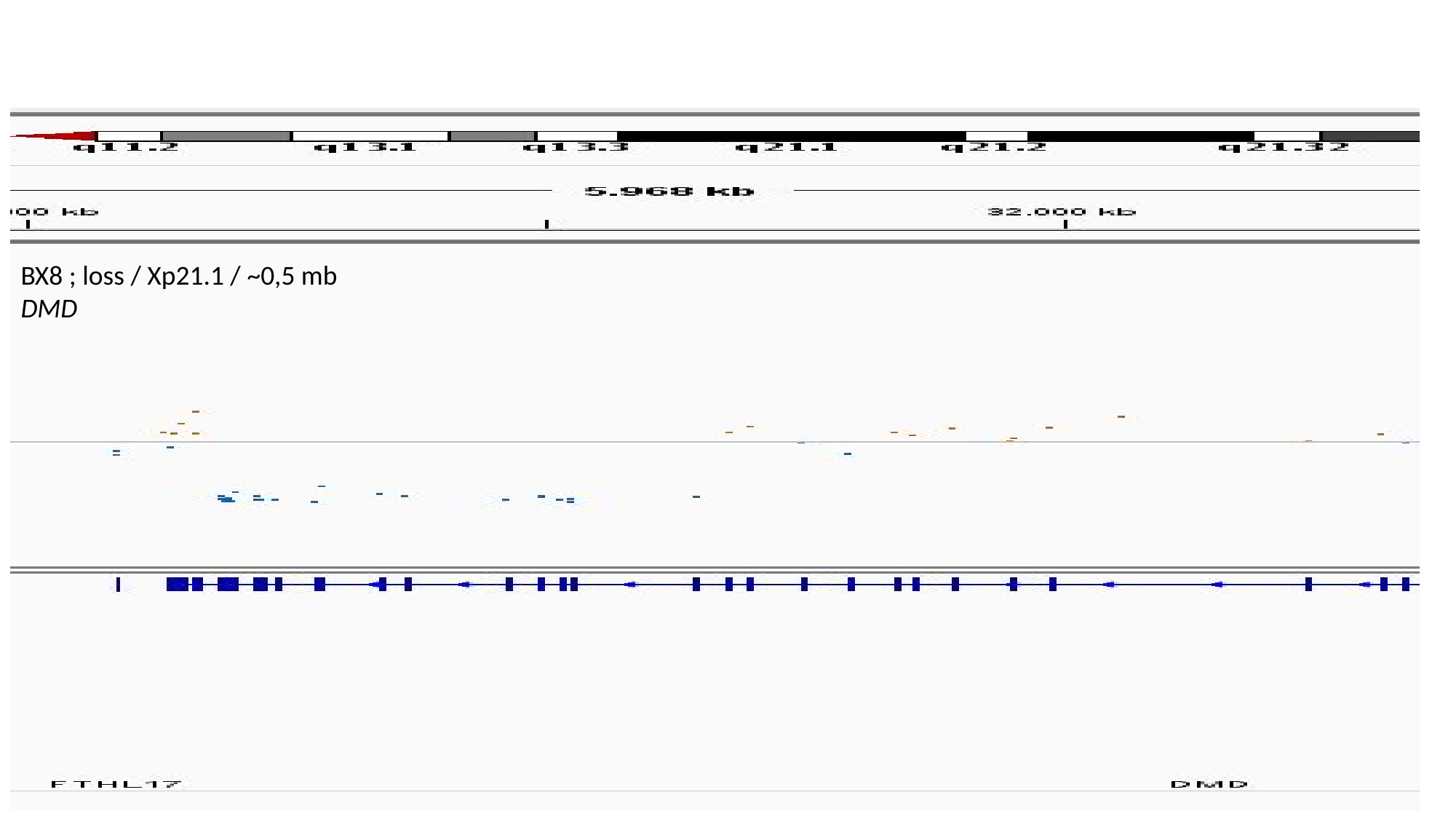

BX8 ; loss / Xp21.1 / ~0,5 mb
DMD

## Slide 108
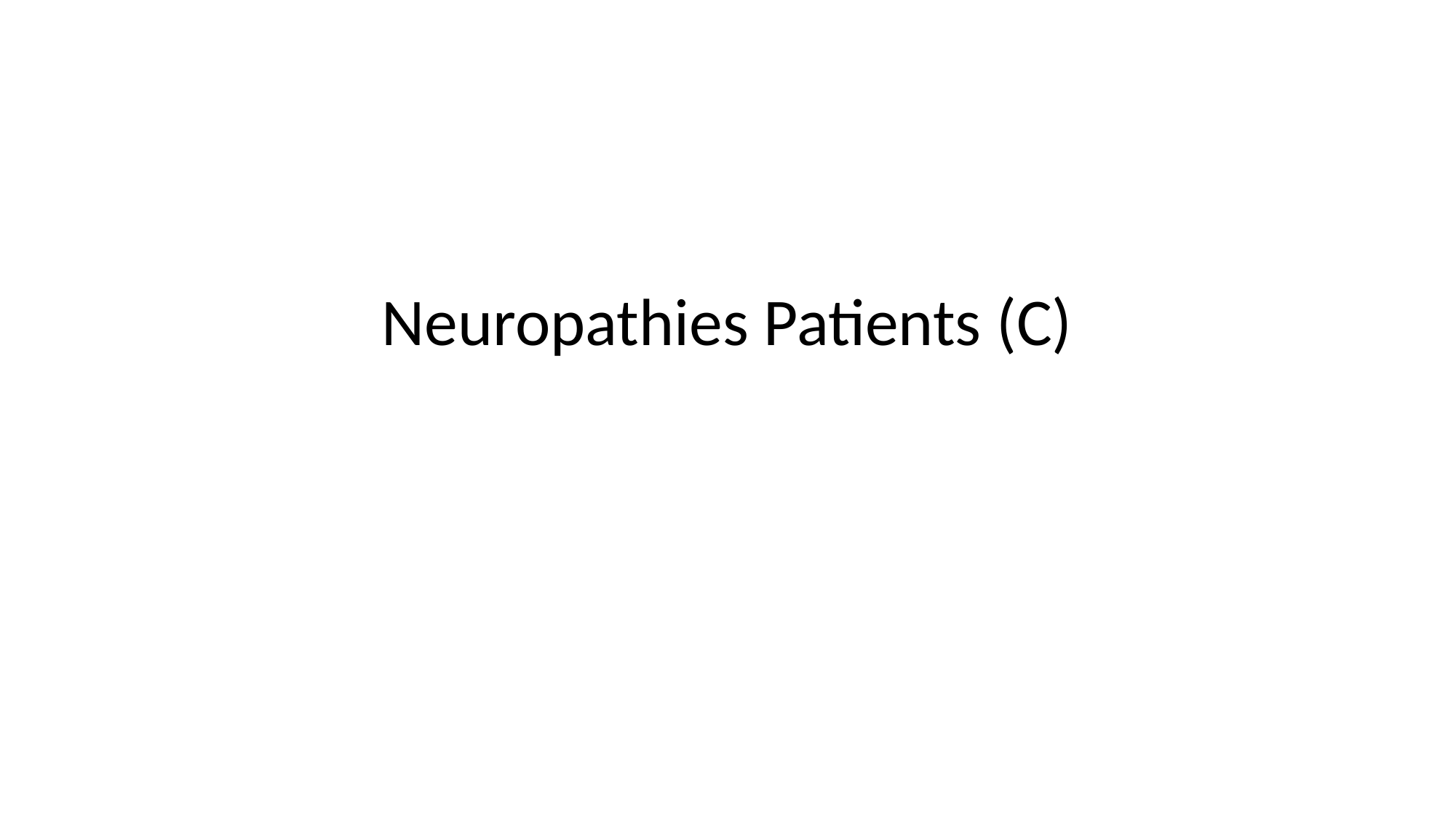

Neuropathies Patients (C)

## Slide 109
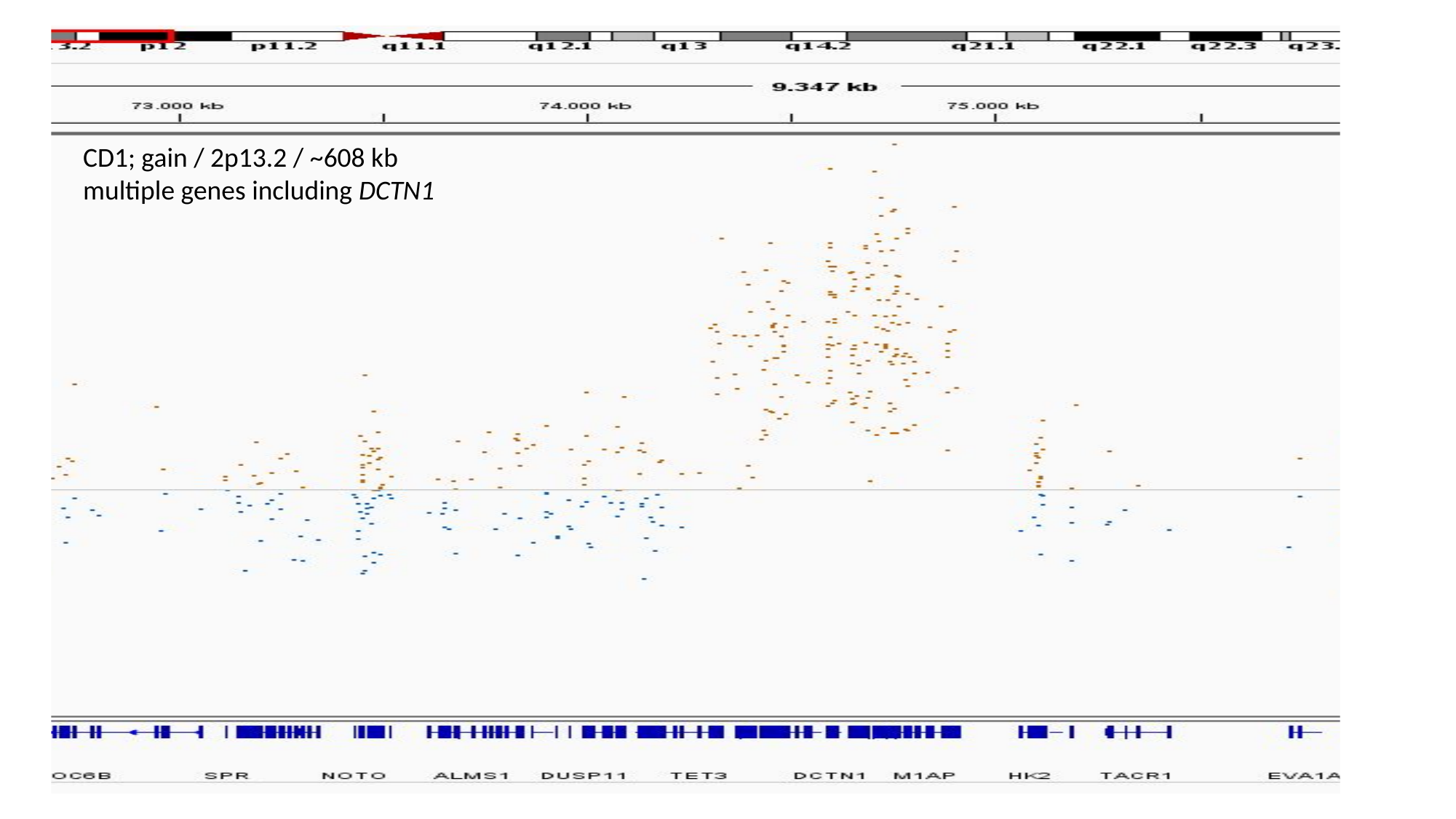

CD1; gain / 2p13.2 / ~608 kb
multiple genes including DCTN1

## Slide 110
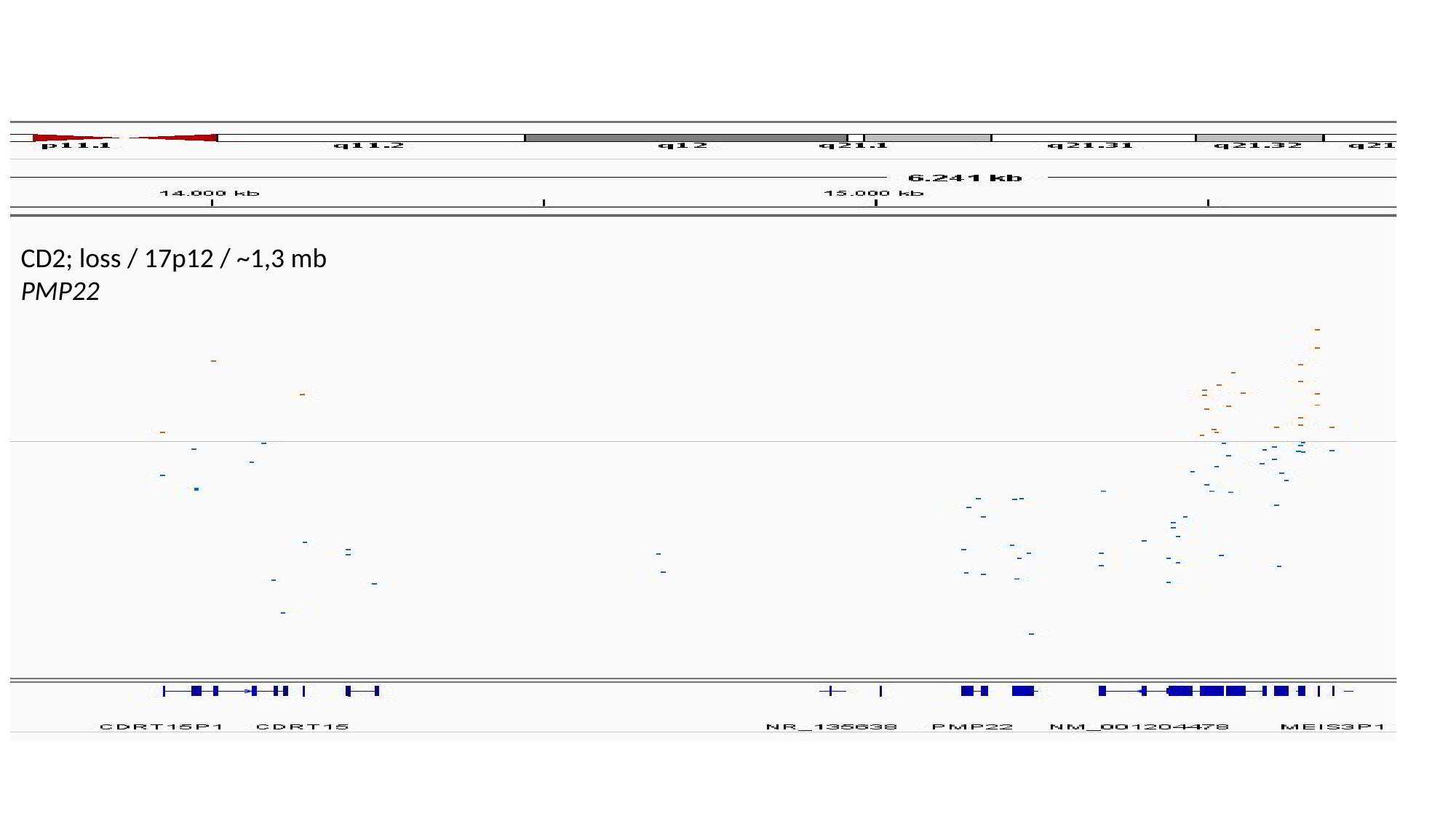

CD2; loss / 17p12 / ~1,3 mb
PMP22

## Slide 111
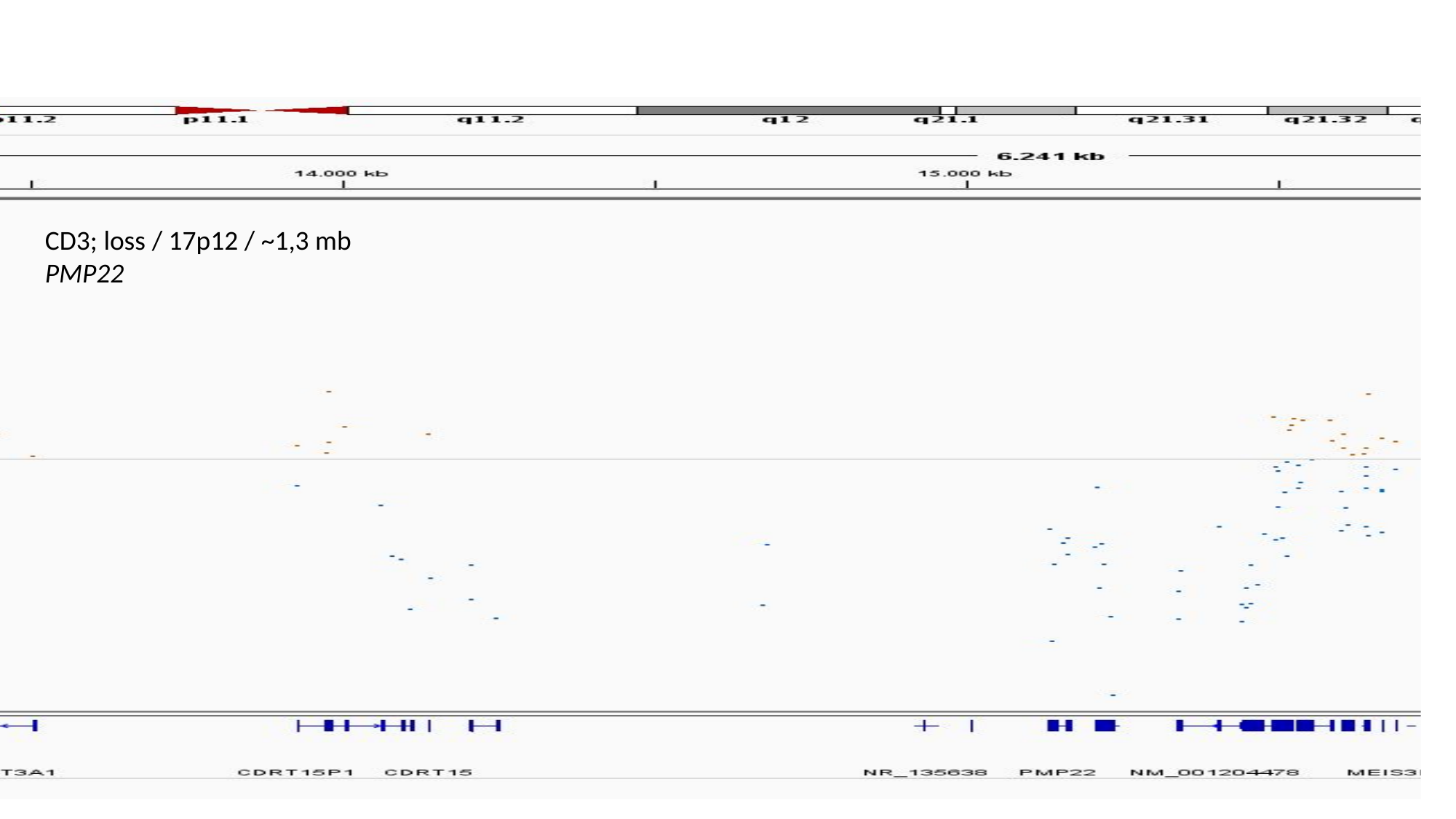

CD3; loss / 17p12 / ~1,3 mb
PMP22

## Slide 112
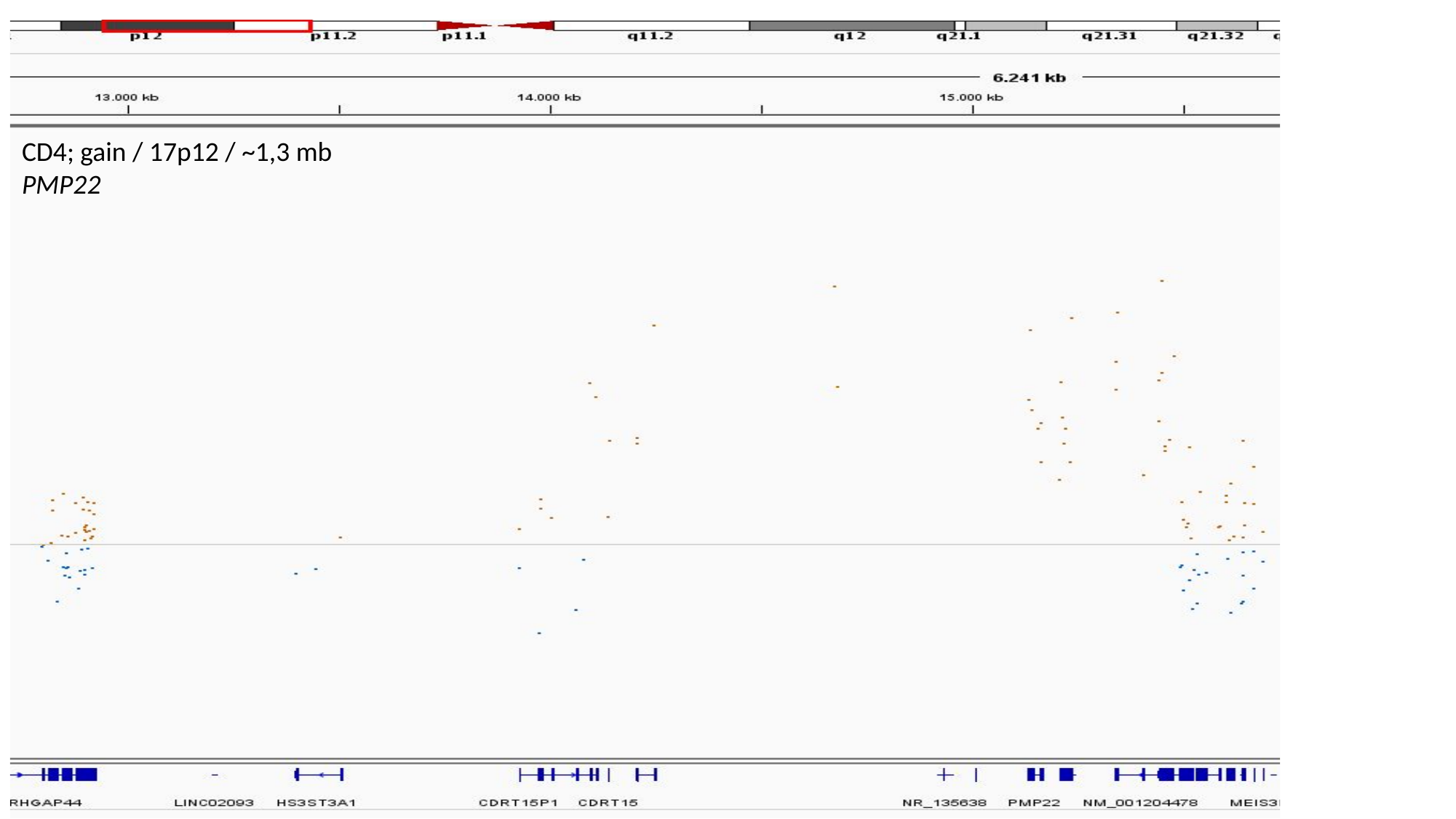

CD4; gain / 17p12 / ~1,3 mb
PMP22

## Slide 113
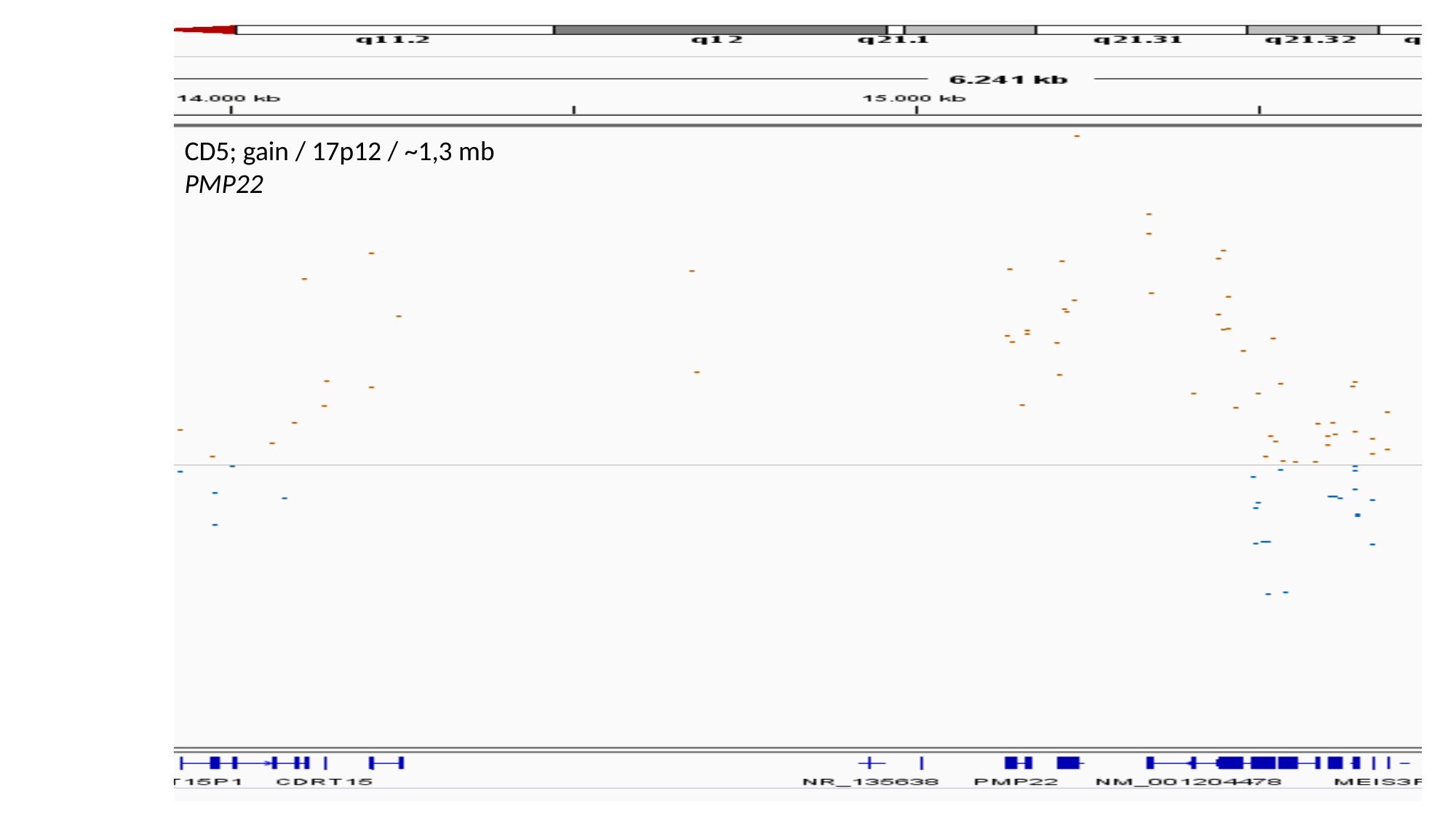

CD5; gain / 17p12 / ~1,3 mb
PMP22

## Slide 114
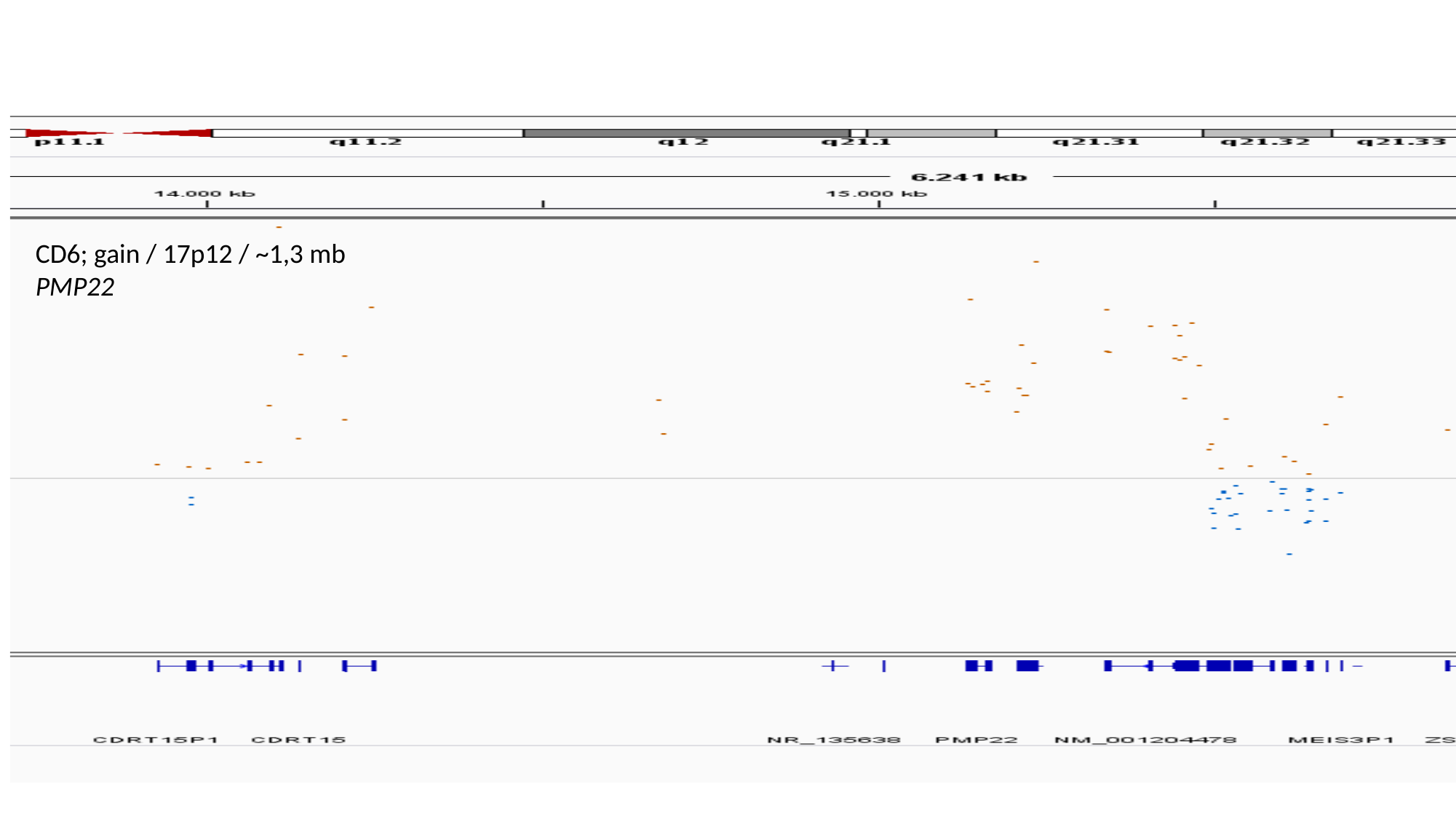

CD6; gain / 17p12 / ~1,3 mb
PMP22

## Slide 115
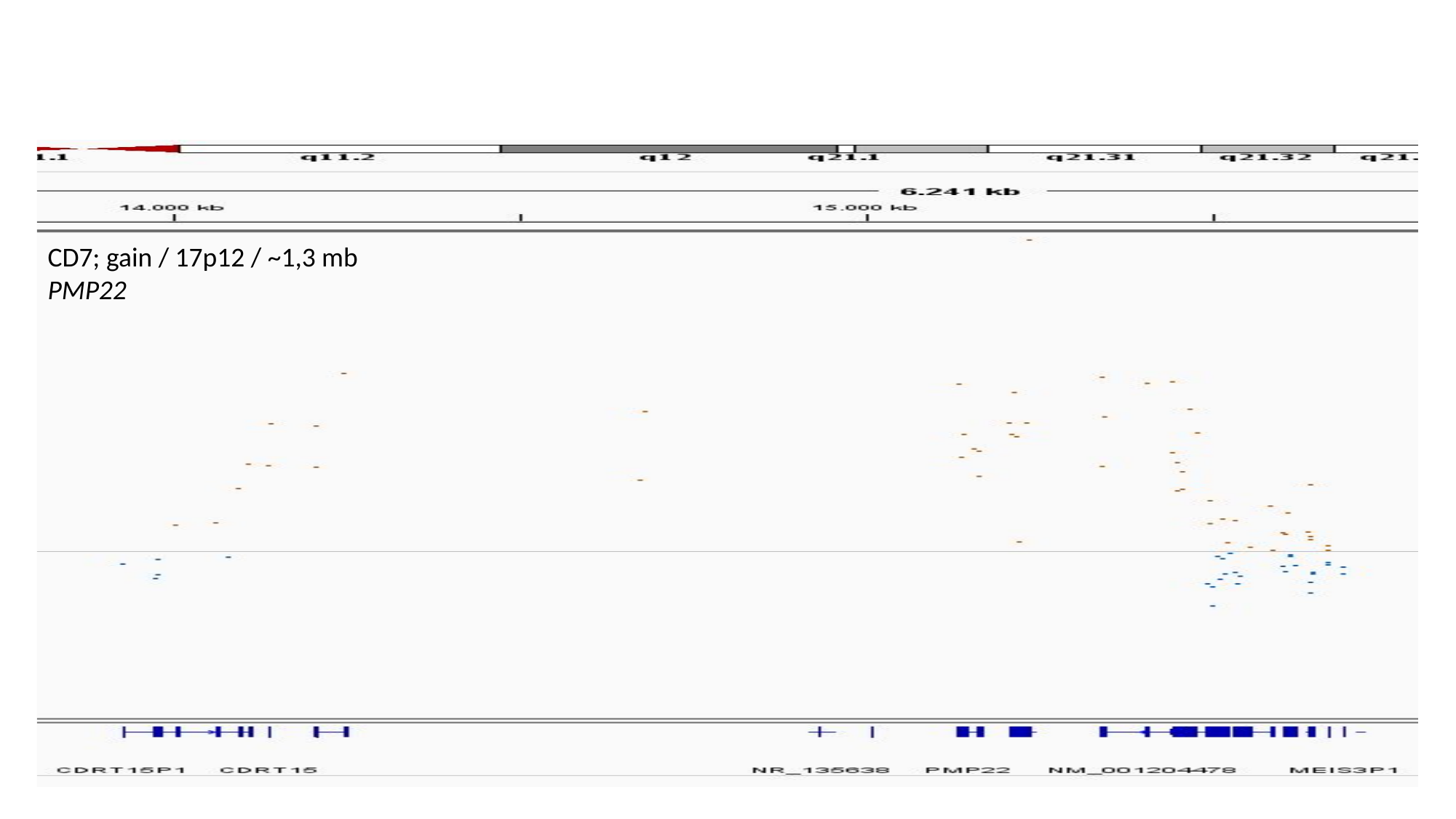

CD7; gain / 17p12 / ~1,3 mb
PMP22
CD 8

## Slide 116
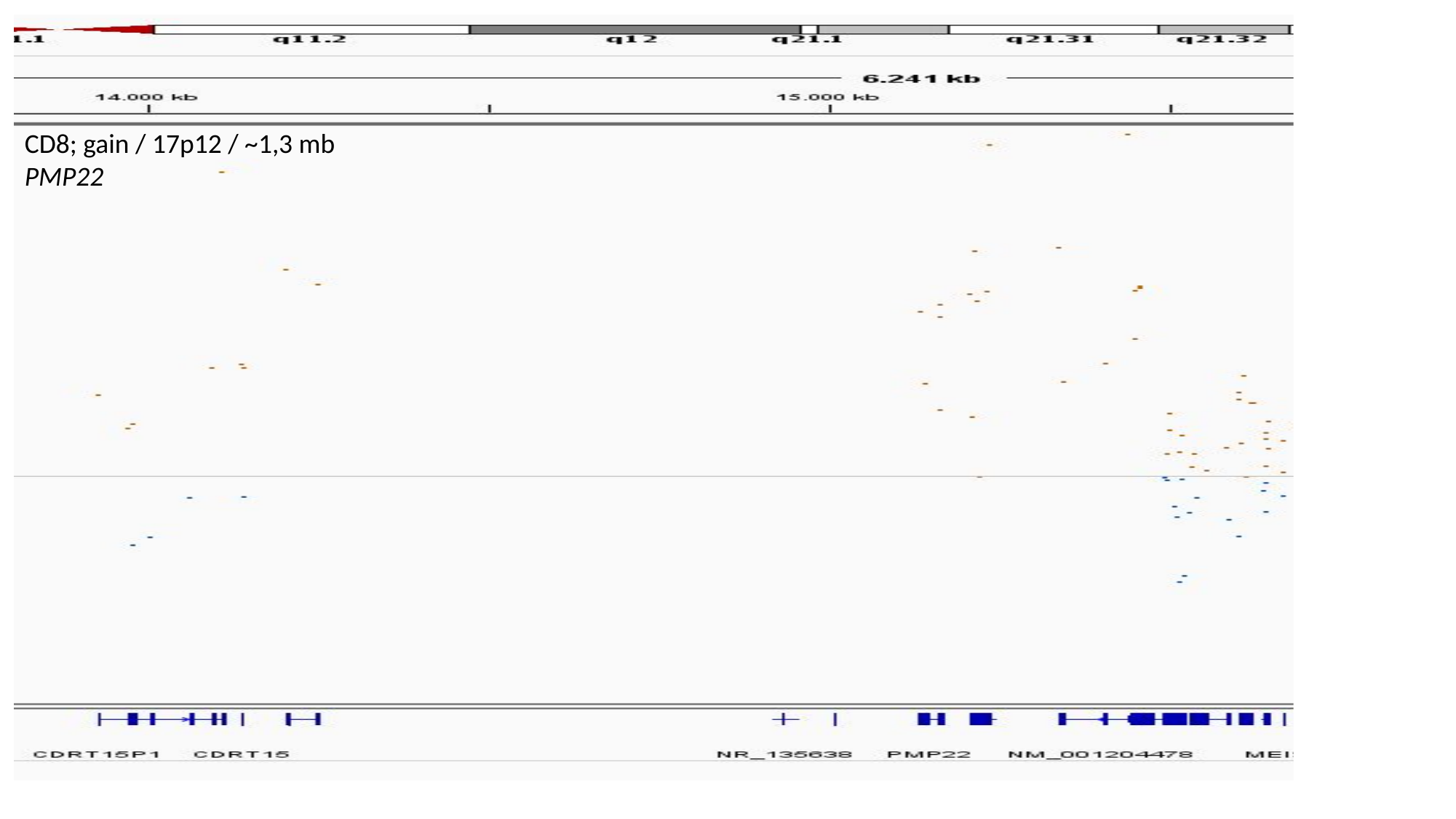

CD8; gain / 17p12 / ~1,3 mb
PMP22

## Slide 117
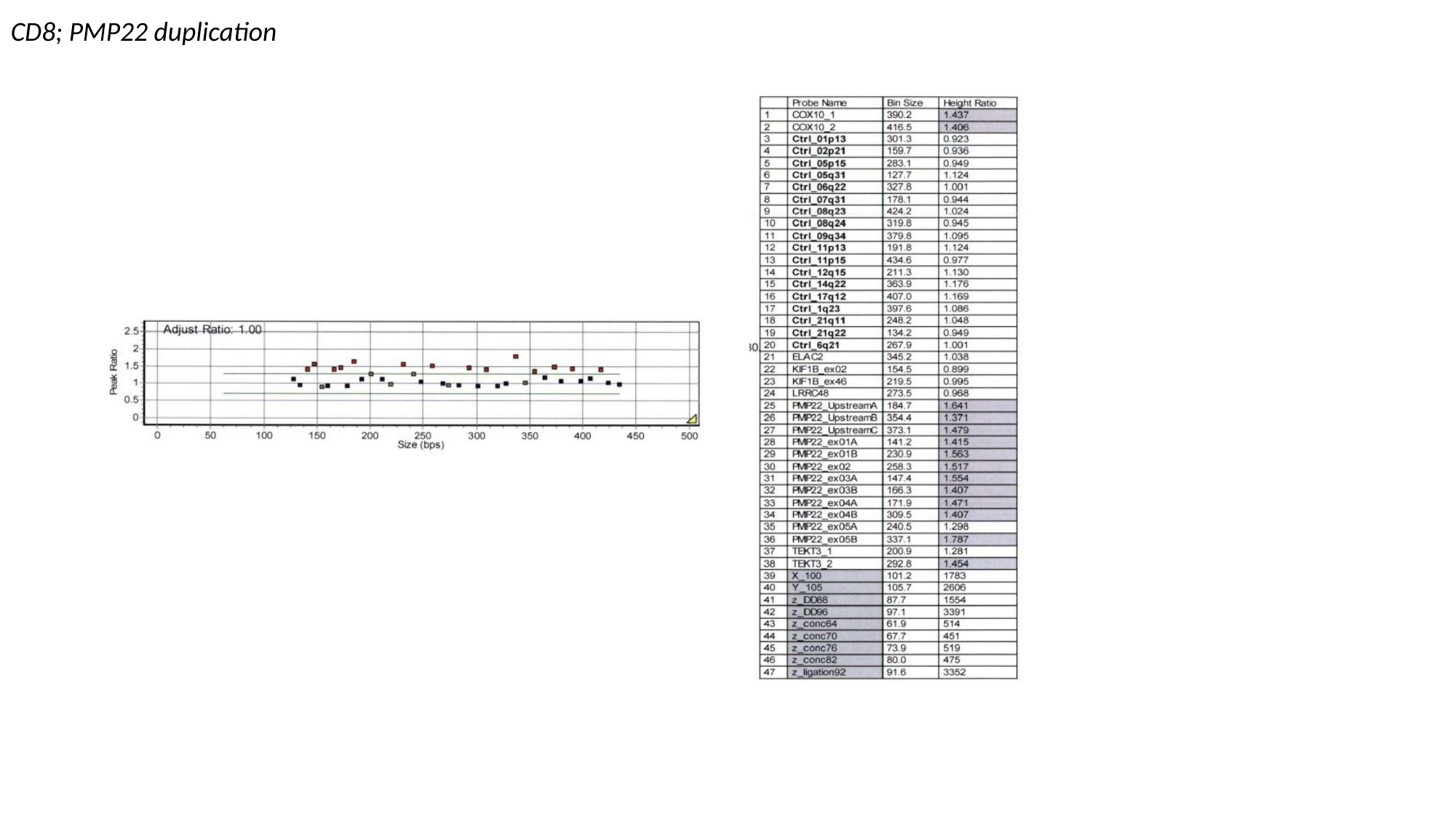

CD8; PMP22 duplication

## Slide 118
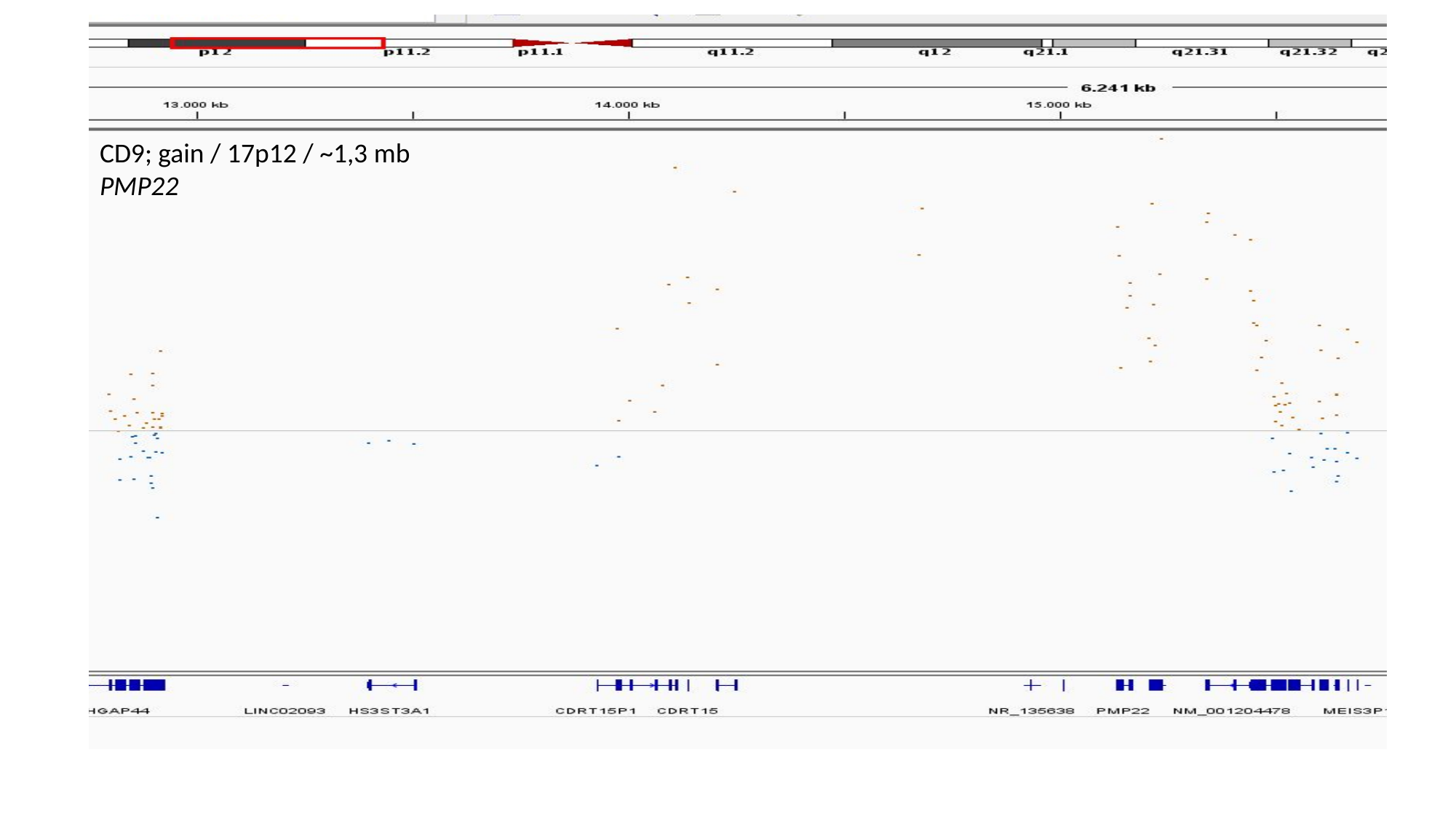

CD9; gain / 17p12 / ~1,3 mb
PMP22

## Slide 119
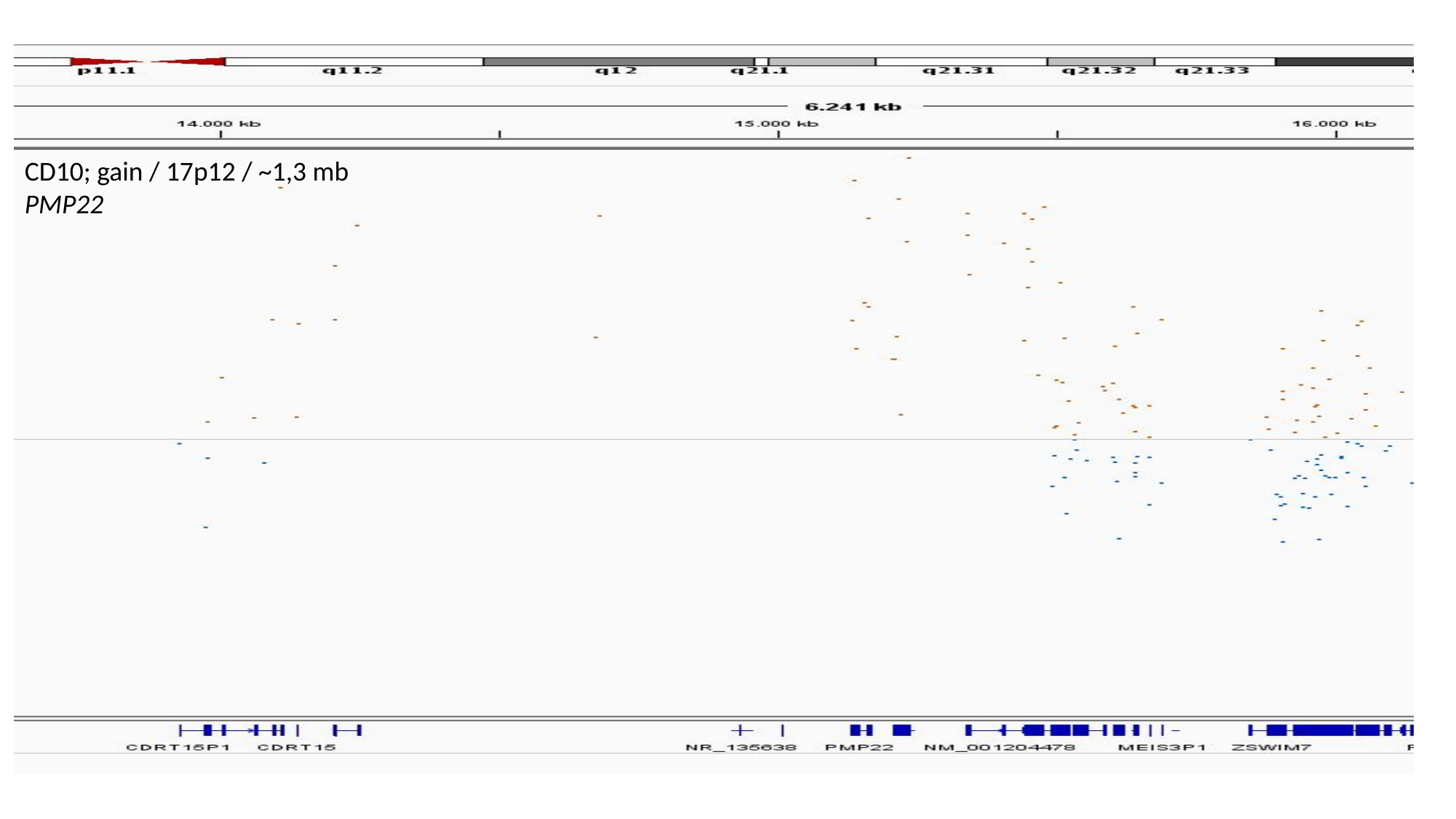

CD10; gain / 17p12 / ~1,3 mb
PMP22

## Slide 120
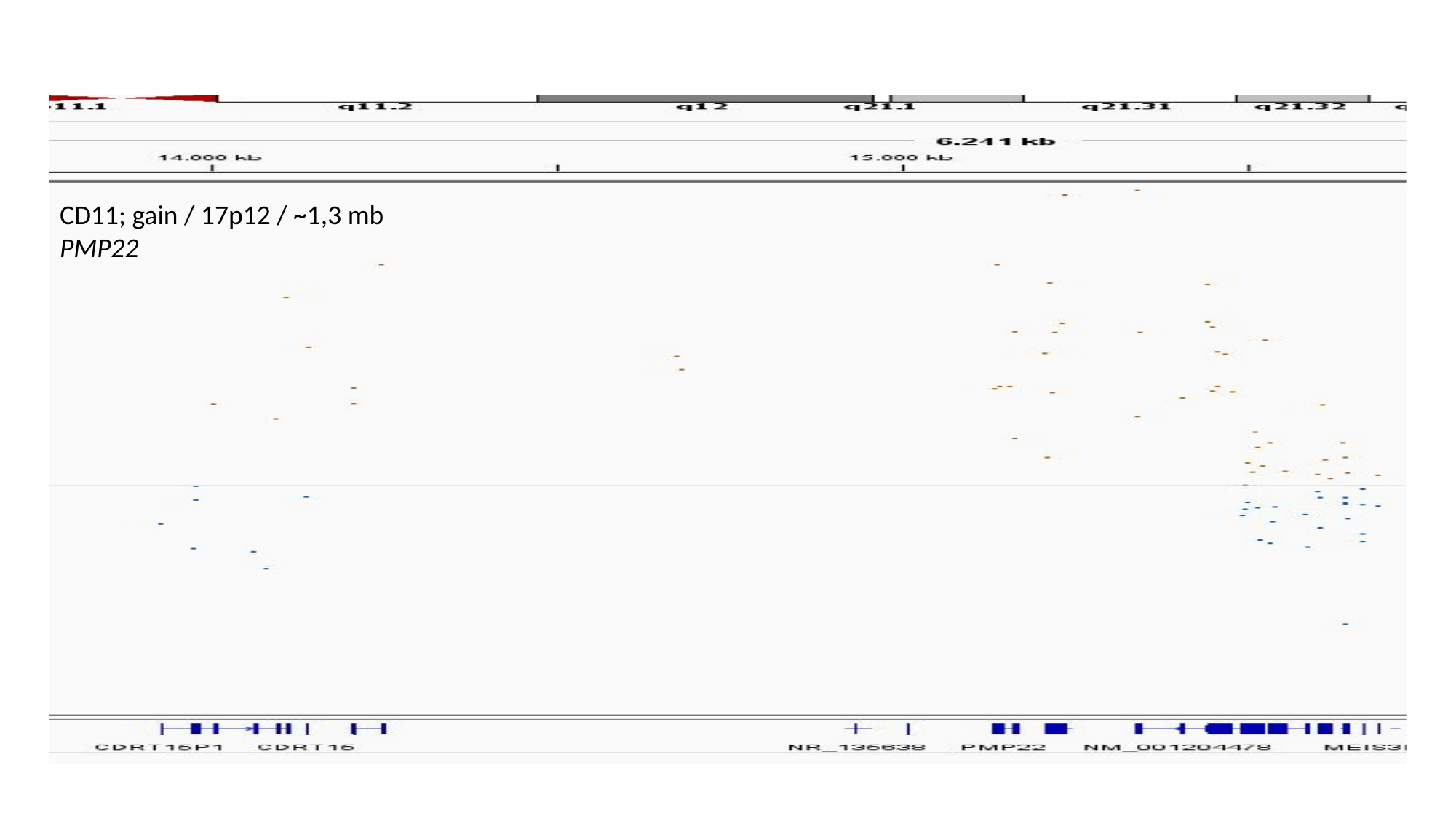

CD11; gain / 17p12 / ~1,3 mb
PMP22

## Slide 121
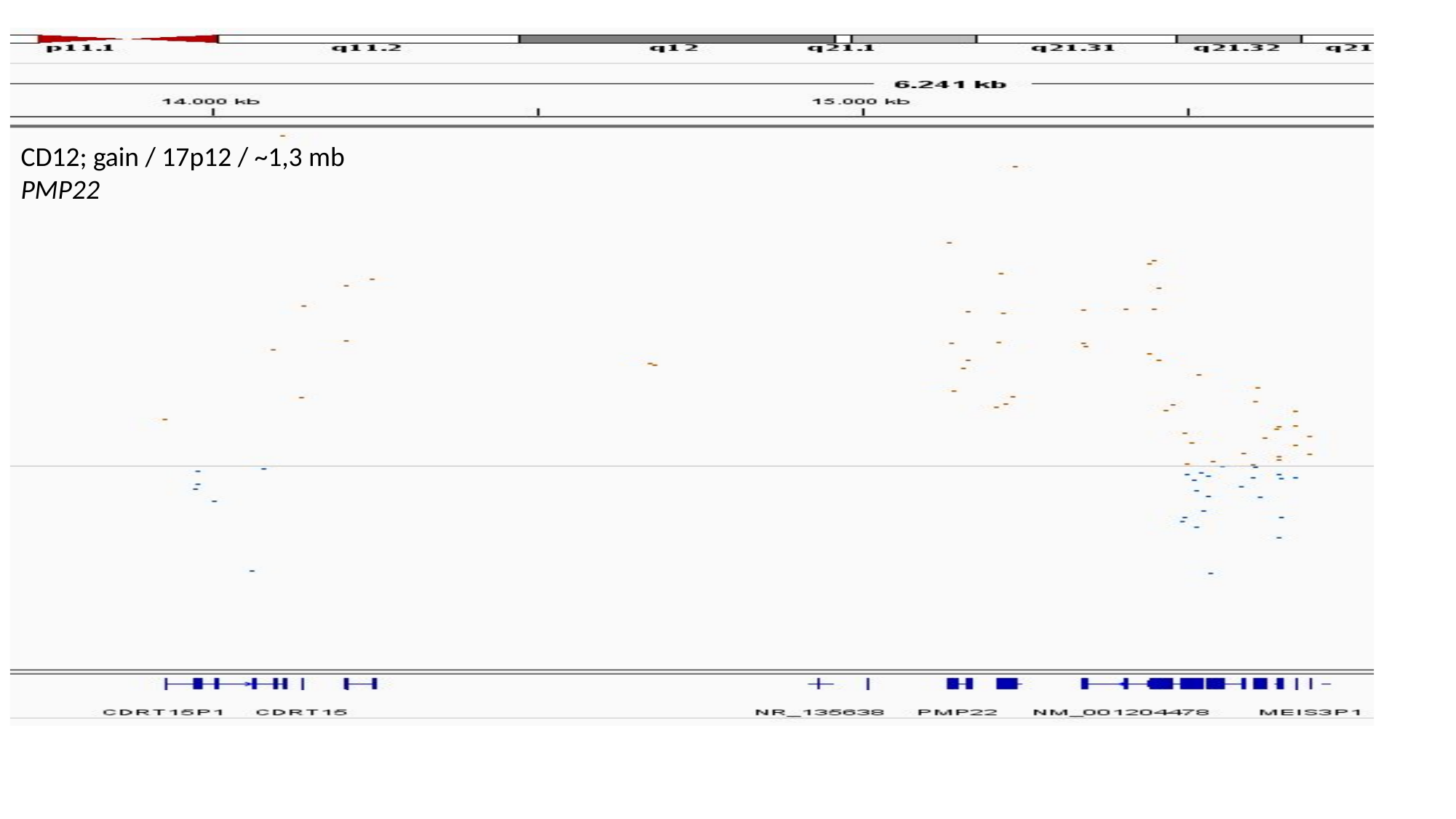

CD12; gain / 17p12 / ~1,3 mb
PMP22

## Slide 122
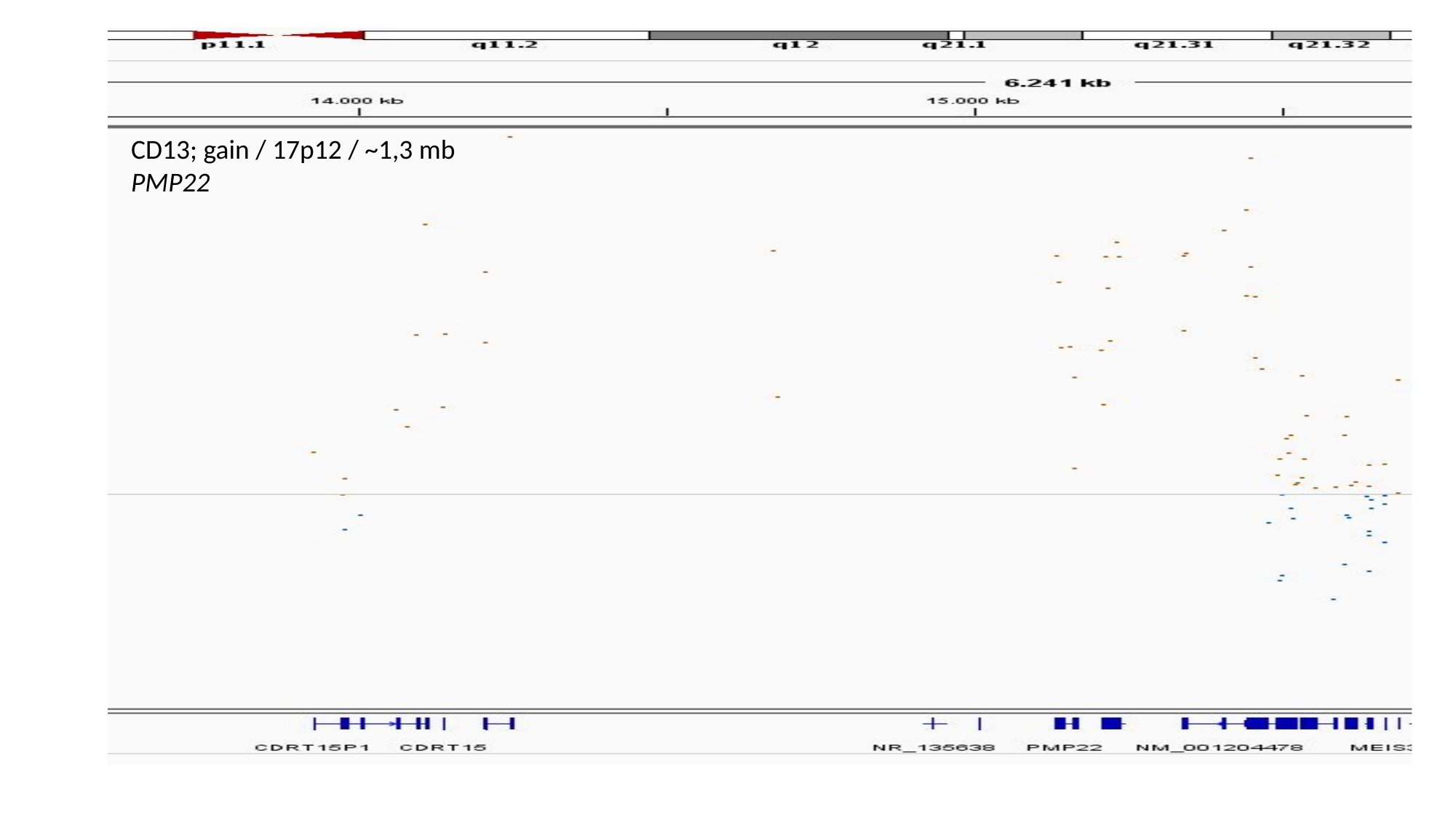

CD13; gain / 17p12 / ~1,3 mb
PMP22

## Slide 123
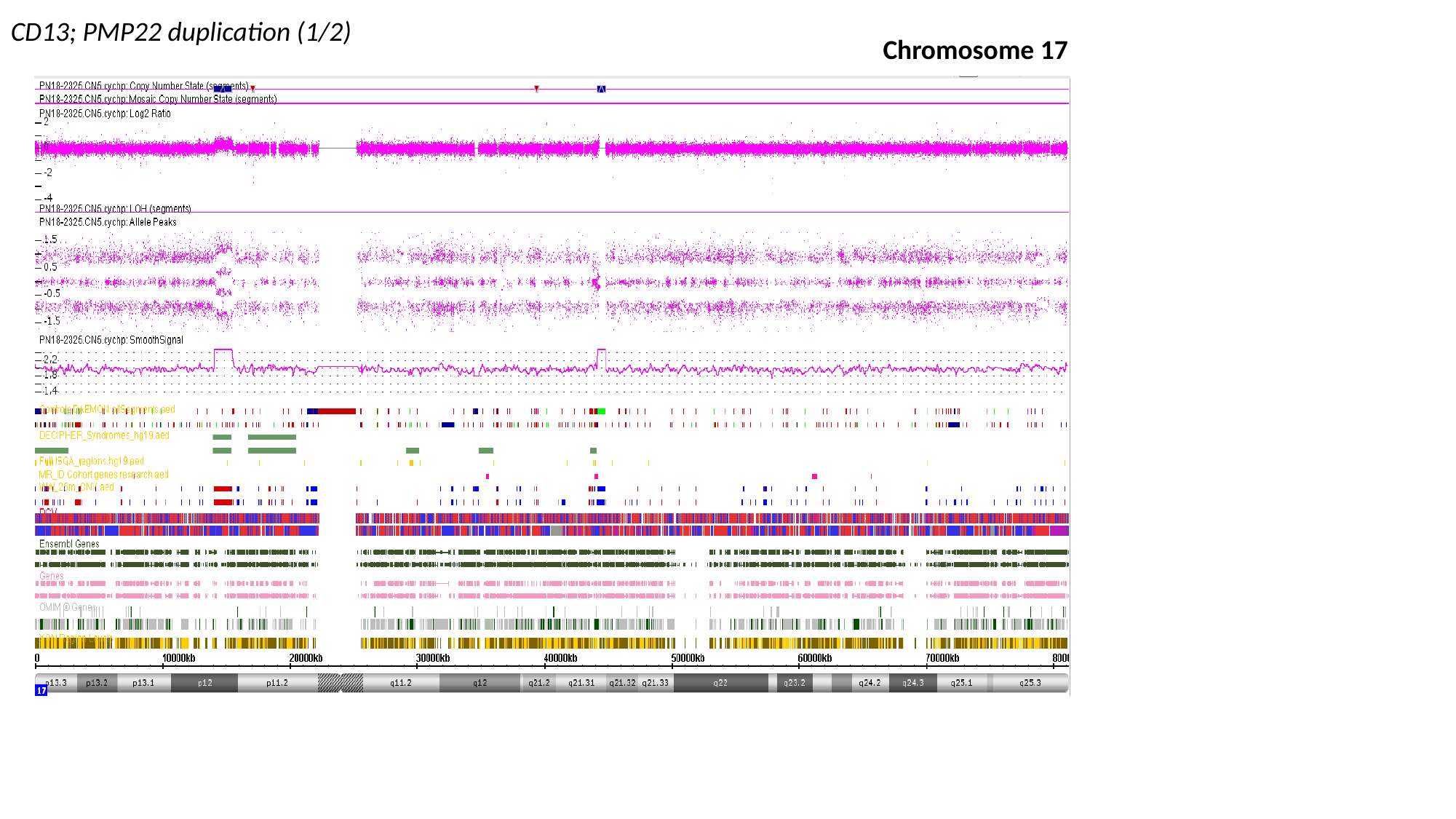

CD13; PMP22 duplication (1/2)
Chromosome 17

## Slide 124
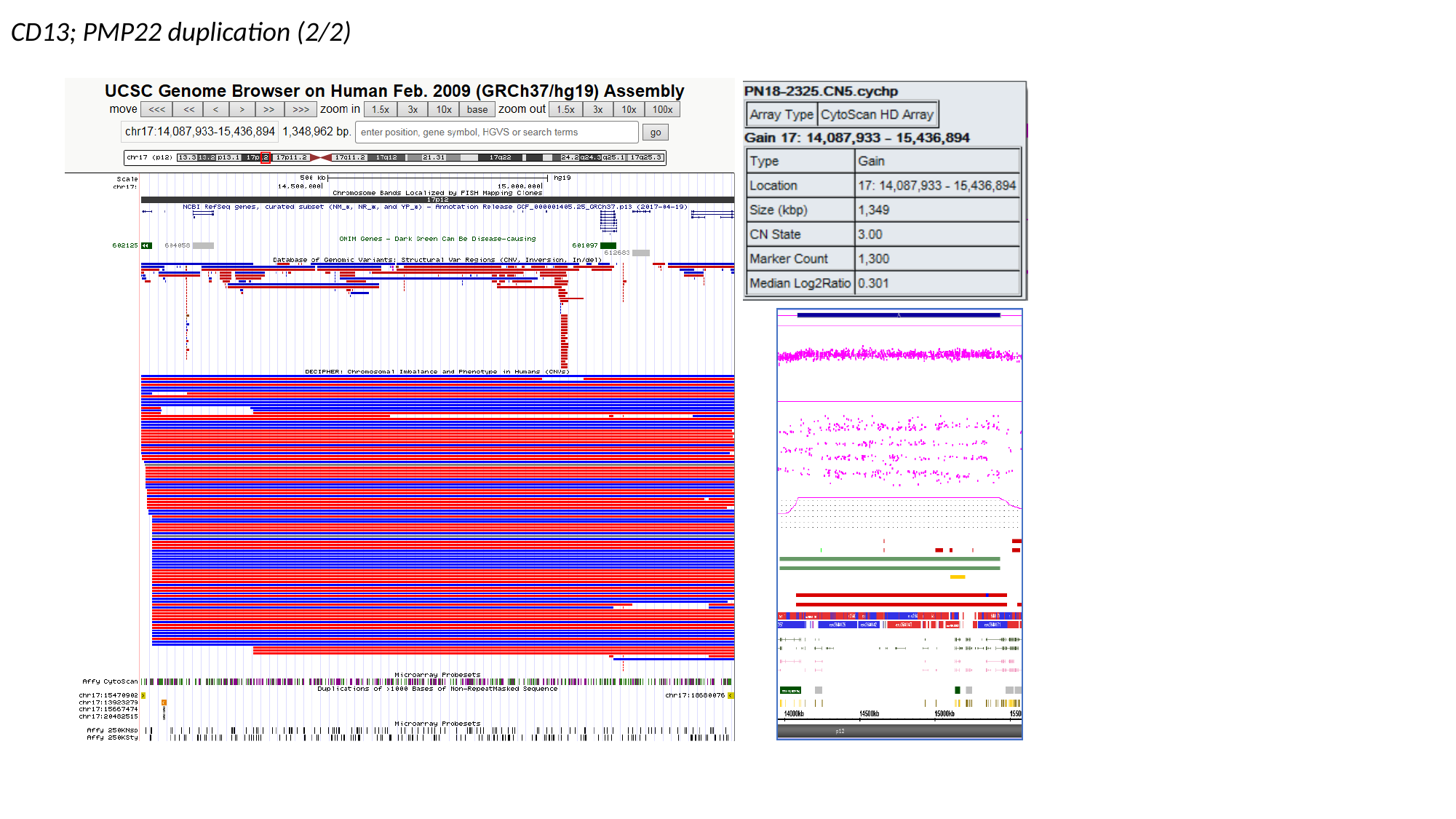

CD13; PMP22 duplication (2/2)

## Slide 125
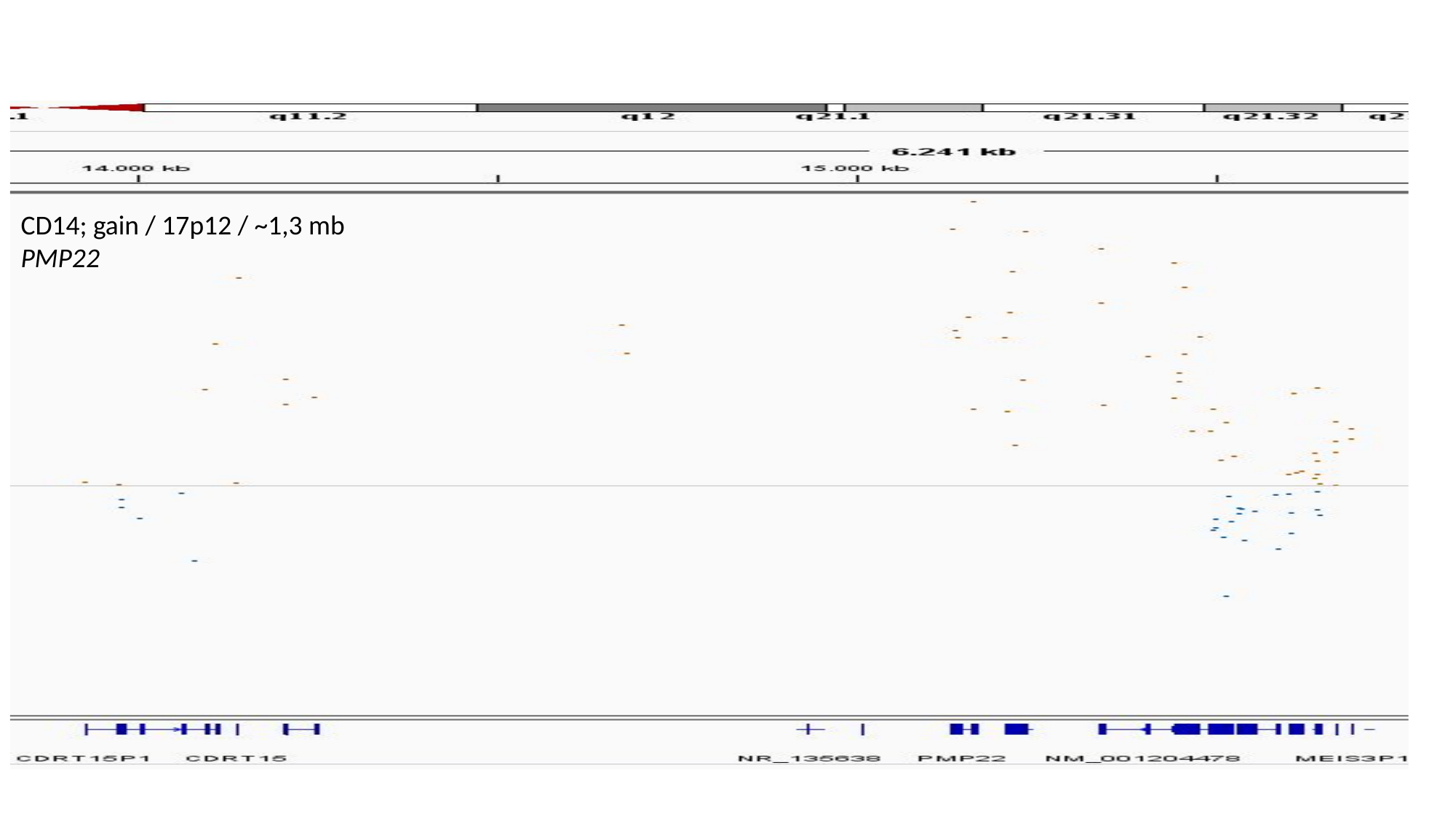

CD14; gain / 17p12 / ~1,3 mb
PMP22

## Slide 126
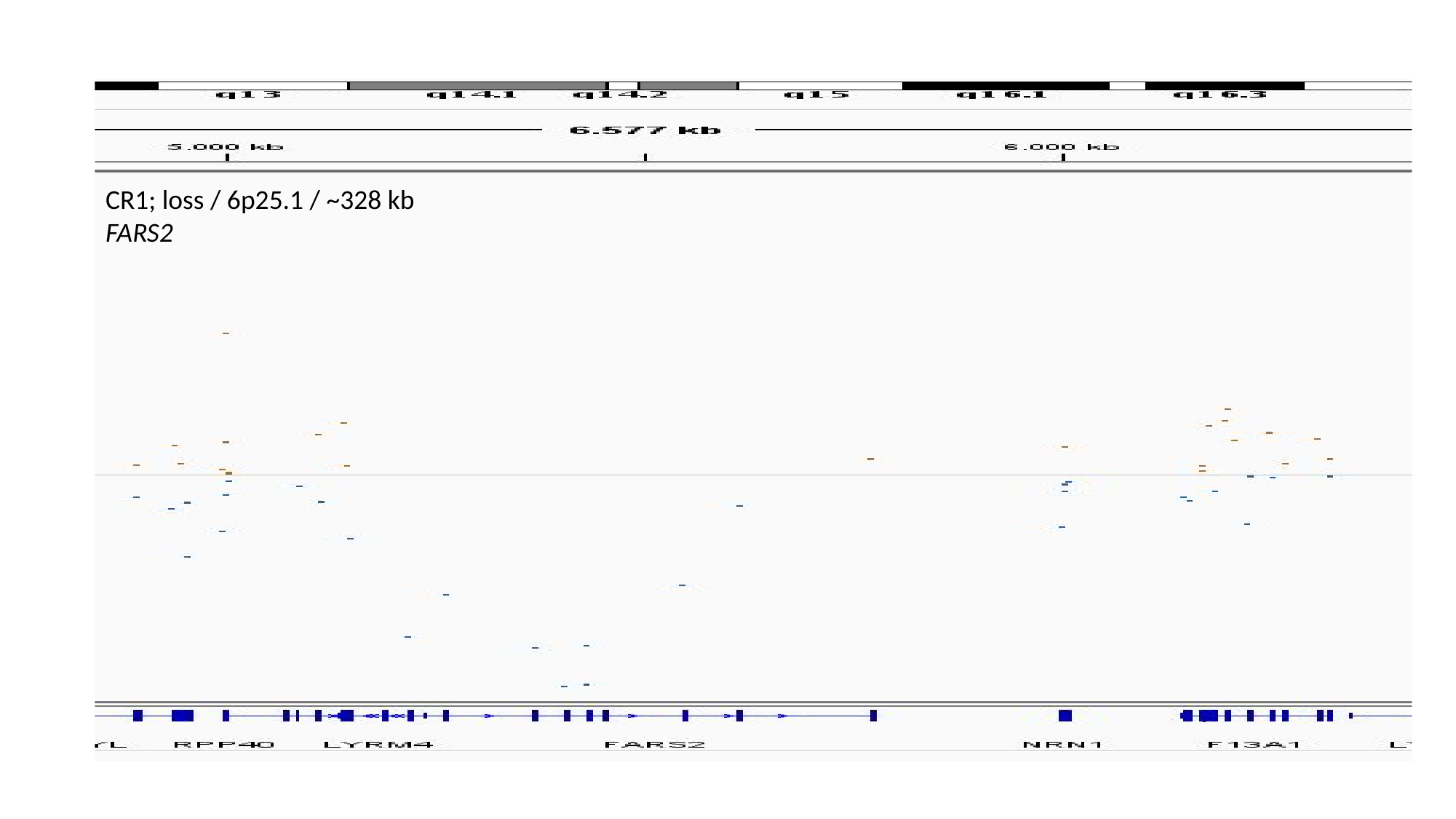

CR1; loss / 6p25.1 / ~328 kb
FARS2
