# Supplementary material for: Copy number variants from 4800 exomes contribute to ~7% of genetic diagnoses in movement disorders, muscle disorders and neuropathies
Source: Eur J Hum Genet. 2023 Feb 13;31(6):654–62. doi: 10.1038/s41431-023-01312-0 (PMC10250492; doi:10.1038/s41431-023-01312-0)
Supplement: Supplementary file 3 — Sup. Table 1 [file 41431_2023_1312_MOESM3_ESM.pdf]

Genes in Parkinson Panel

ATP1A3
C19orf12
CHCD2
CHMP2B
CSF1R
DCTN1
DNAJC6
FBXO7
FTL
GBA
GCH1
GRN
LRRK2
MAPT
MYORG
PARK7
PDGFB
PDGFRB
PINK1
PLA2G6
POLG
PRKN
PRKRA
PSEN1
SLC20A2
SLC30A10
SLC39A14
SLC6A3
SNCA
TAF1
TH
VPS13C
VPS35
WDR45
XPRR1
