# Supplementary material for: Copy number variants from 4800 exomes contribute to ~7% of genetic diagnoses in movement disorders, muscle disorders and neuropathies
Source: Eur J Hum Genet. 2023 Feb 13;31(6):654–62. doi: 10.1038/s41431-023-01312-0 (PMC10250492; doi:10.1038/s41431-023-01312-0)
Supplement: Supplementary file 4 — Sup. Table 2 [file 41431_2023_1312_MOESM4_ESM.pdf]

Individual number	Sex	age onset / age at WES request	Reason	Disease causing gene(s)	Intragenic /# of genes	Variant^	Copy number(s)	inheritance	Classification*	Likely diagnosis	OMIM disease	LOVD ID
**Inheritance pattern of the involved variant/gene: AD**												
AD1	M	? / 14	abnormal movement pattern	contiguous gene duplication syndrome	> 10	seq[GRCh37] 22q11.2q11.2(18893960_20307418)x3<br>NC_000022.10:g.(18775421_18893960)_(20307418_20383786)dup[c]	3	nd	P	y	Chromosome 22q11.2 duplication syndrome (#608363)	chr22_003028
AD2	M	4 / 58	psychomotor delay, short stature, hypertelorism, ptosis, dystonia, extrapyramidal features	contiguous gene deletion syndrome	> 50	seq[GRCh37] 18p11.32p11.21(1168410_14764089)x1<br>NC_000018.9:g.(pter_1168410)_(14764089_14772154)del	1	nd	P	y	Chromosome 18p deletion syndrome (#146390)	chr18_02478
AD3	M	? / 7	spastic paraparesis, IQ57	contiguous gene deletion syndrome	> 50	seq[GRCh37] 2q37q37(238482964_243037178)x1<br>NC_000002.11:g.(238475818_238482964)_(243037178_qter)del	1	nd	P	y	2q37 deletion syndrome (#600430)	chr2_020446
AD4	M	? / 2	hemiplegia	contiguous gene deletion syndrome	> 40	seq[GRCh37] 16p11.2p11.2(29468940_30199846)x1<br>NC_000016.9:g.(29376391_29468940)_(30199846_30208282)del[c]	1	maternal	P	uncertain (incomplete penetrance)	Chromosome 16p11.2 deletion syndrome (*TBX6*)[33]	chr16_006613
AD5	M	0 / 11	hypotonia, facial muscle weakness, choreoathetotic movements	contiguous gene duplication syndrome	> 50	seq[GRCh37] 15q11q13(20170101_32449994)x3<br>NC_000015.9:g.(pter_20170101)_(32449994_32450569)dup	3	nd	P	y	Chromosome 15q11-q13 duplication syndrome (#608636)	chr15_005981
AD6	M	0/ 1	hypotonia, epilepsy	contiguous gene deletion syndrome	~ 20	seq[GRCh37] 15q11.2q13.1(23684730_28544722)x1<br>NC_000015.9:g.(23330473_23684730)_(28544722_28632805)del	1	nd	P	y	Prader-Willi / Angelman syndrome (#176270 and #105830)	chr15_005982
AD7	M	? / 37	ataxia, obesity, cognitive impairment	contiguous gene deletion syndrome	~ 30	seq[GRCh37] 16p11.2p11.2(28403054_(29376391)x1<br>NC_000016.9:g.(28332251_28403054)_(29376391_29468940)del	1	nd	P	uncertain (incomplete penetrance)	Chromosome 16p11.2 deletion syndrome (*SH2B1*)[&]	chr16_006614
AD8	F	45 / 59	multiple cerebral infarctions, walking problems, speech problems, memory problems, MRI shows leukoencephalopathy and small vessel disease	*COL4A1, COL4A2*	2	seq[GRCh37] 13q34q34(110801458_111009963)x3<br>NC_000013.10:g.(110438412_110801458)_(111009963_111077120)dup	3	nd	LP	y	Brain small vessel disease with or without ocular anomalies (#175780) and Brain small vessel disease 2 (#614483)	chr13_002359
AD9	M	? / 4	seizures, dystonia	*CACNB4, SCN1A, SCN2A*	4 / >50	seq[GRCh37] 2q23.3q23.3(152520033_153192242)x3<br>NC_000002.11:g.(152518886_152520033)_(153192242_153378379)dup and seq[GRCh37] 2q24.2q31.1(164450154_173916537)x3<br>NC_000002.11:g.(163695052_164450154)_(173916537_174047565)dup[c]	3	de novo (paternity and maternity checked)	LP	y	Episodic ataxia type 5 (#613855), Epileptic encephalopathy type 6 (#607208), Familial febrile seizures 3A (#604403), Epileptic encephalopathy 11 (#613721) and Familial infantile<br>seizures 3 (#607745)	chr2_020447 and chr2_020456
AD10	M	? / 39	episodic ataxia	*CACNA1A*	Intragenic	seq[GRCh37] 19p13.2p13.2(13335371_13346648)x1<br>NC_000019.9:g.(13325430_13335371)_(13346648_13352244)delc<br>*exon 33-39 (NM_023035.3) Out-of-frame*	1	maternal	LP	y	Episodic ataxia type 2 (#108500)	CACNA1A_000464
AD11	M	? / 44	episodic ataxia	*CACNA1A*	Intragenic	seq[GRCh37] 19p13.2p13.2(13445205_13446729)x1<br>NC_000019.9:g.(13441150_13445205)_(13446729_13470438)delc<br>*exon 7-8 (NM_023035.3) Out-of-frame*	1	nd	P	y	Episodic ataxia type 2 (#108500)	chr19_007427
AD12	M	? / 47	episodic ataxia	*CACNA1A*	Intragenic	seq[GRCh37] 19p13.2p13.2(13470613_13476283)x1<br>NC_000019.9:g.(13470613_13470613)_(13476283_13482502)del[c]<br>*exon 5 (NM_023035.3) In-frame*	1	maternal	LP	y	Episodic ataxia type 2 (#108500)	chr19_007428
AD13	M	50 / 63	spastic ataxia, dysarthria	*CACNA1G*	10	seq[GRCh37] 17q21.33q21.33(48469758_48697187)x3<br>NC_000017.10:g.(48465493_48469758)_(48697187_48699020)dup	3	nd	VOUS	uncertain	Spinocerebellar ataxie type 42 (#616795)	chr17_008700
AD14	M	? / 18	partial DOPA-responsive dystonia	*GCH1*	Intragenic	seq[GRCh37] 14q22.1q22.1(55369012_55369387)x1<br>NC_000014.8:g.(55344978_55369012)_(55369387_55408261)delc<br>*exon 1 (NM_000161.3) Out-of-frame*	1	nd	P	y	DOPA-responsive dystonia (#128230)	chr14_005090
AD15	F	? / 67	dystonia and dysarthria	*GNAL*	~ 28	seq[GRCh37] 18p11.22p11.21(9708318_12884298)x1<br>NC_000018.9:g.(9595186_9708318)_(12884298_12948060)del	1	nd	P	y	Dystonia 25 (#615073)	chr18_002479
AD16	F	<10 / 47	ataxia	*ITPR1*	Intragenic	seq[GRCh37] 3p26.1p26.1(4558177_4752241)x1<br>NC_000003.11:g.(4508999_4558177)_(4752241_4753421)del<br>*exon 1-37 (NM_002222.6) In-frame*	1	nd	P	y	spinocerebellaire ataxie type 15 (606658) and type 29 (#117360)	chr3_007292
AD17	M	<10 / 22	fever-induced ataxia, cerebellar atrophy, myoclonus, chorea and dystonia	*NKX2-1*	~ 20	seq[GRCh37] 14q13.2q21.1(36064815_39594432)x1<br>NC_000014.8:g.(36044496_36064815)_(39594432_39769044)del	1	nd	P	y	NKX2-1: Choreoathetosis, hypothyroidism and neonatal respiratory distress (#610978) and Hereditary benign chorea (#118700)	chr14_005091
AD18	F	? / 6	ataxia	*NKX2-1*	4	seq[GRCh37] 14q13.3q13.3(36982316_37344209)x1<br>NC_000014.8:g.(36960496_36982316)_(37344209_37641230)del	1	nd	P	y	NKX2-1: Choreoathetosis, hypothyroidism and neonatal respiratory distress (#610978) and Hereditary benign chorea (#118700)	chr14_005092
AD19	M	? / 25	dystonia, myoclonus	*SGCE*	Intragenic	seq[GRCh37] 7q21.3q21.3(94228057_94230141)x1<br>NC_000007.13:g.(94218180_94228057)_(94230141_94248020)del<br>*exon 7-9 (NM_003919.3) Out-of-frame*	1	nd	P	y	myoclonic dystonia type 11 (#159900)	chr7_006039
AD20	F	? / 6	myoclonic movements of the head and right arm	*SGCE*	~ 15	seq[GRCh37] 7q21.2q21.3(92730616_94953813)x1<br>NC_000007.13:g.(92462638_92730616)_(94953813_94989267)del	1	nd	P	y	myoclonic dystonia type 11 (#159900)	chr7_006040
AD21	F	? / 29	dystonia, myoclonus	*SGCE*	5	seq[GRCh37] 7q21.3q21.3(94024386_94540787)x1<br>NC_000007.13:g.(93633589_94024386)_(94540787_94740572)del	1	nd	P	y	myoclonic dystonia type 11 (#159900)	chr7_006042
AD22	F	35 / 41	spastic paraparesis	*SPAST*	2	seq[GRCh37] 2p22.3p22.3(32288679_32449181)x1<br>NC_000002.11:g.(32264395_32288679)_(32449181_32449517)del	1	nd	P	y	Spastic paraplegia type 4 (#182601)	chr2_020448
AD23	F	20 / 51	spastic paraparesis	*SPAST*	2	seq[GRCh37] 2p22.3p22.3(32312485_32400490)x1<br>NC_000002.11:g.((32289336_32312485)_(32400490_32402622)del	1	nd	P	y	Spastic paraplegia type 4 (#182601)	chr2_020449

Inheritance: AR

AR1	M	40 / 51	polyneuropathy, pyramidal features	*B4GALNT1*	2	seq[GRCh37] 12q13.3q13.3(58013640_58024073)x1<br>NC_000012.11:g.(58013640_58013640)_(58024073_58024237)del<br>*exon 6-11 (NM_001478.5)*<br>*second variant: c.451G>A p.(Gly151Ser)*	1	nd	P<br>VOUS	y	Spastic paraplegia type 26 (#609195)	chr12_007428<br>B4GALNT1_000010
AR2	M	? / 15	paraplegia, cognitive delay	*DDHD2*	Intragenic	*seq[GRCh37] 8p11.23p11.23(38107219_38112943)x0*<br>*NC_000008.10:g.(38105438_38107219)_(38112943_38117509)del*<br>*exon 11-16 (NM_015214.3) Out-of-frame*	*0*	nd	P	y	Spastic paraplegia type 54 (#615033)	chr8_005236

AR3	M	? / 11	dystonia, progressive bipiramidal syndrome, gait disturbances	*FARS2*	Intragenic	seq[GRCh37] 6p25.1p25.1(5368795_5369464)x1<br>NC_000006.11:g.(5261001_5368795)_(5369464_5404748)del[c]<br>*exon 1-2 (NM_006567.5) In-frame*<br>*second variant: c.1082C>T p.(Pro361Leu)*	1	nd	P<br>VOUS	y	Combined oxidative phosphorylation deficiency type 14 (#614946) and Spastic paraplegia type 77 (OMIM#617046)	chr6_007447<br>FARS2_000029
AR4	F	? / 50	lethargy, pyramidal features, MRI abnormalities, distal muscle weakness, reduced balance	*GALC*	Intragenic	seq[GRCh37] 14q31.3q31.3(88401222_88417002)x1<br>NC_000014.8:g.(86087482_88401222)_(88417002_88429855)del<br>*exon 11-17 (NM_000153.4) In-frame*<br>*second variant: c.334A>G p.(Thr112Ala)*	1	nd	P<br>VOUS	y	Krabbe disease (#245200)	chr14_005093<br>GALC_00004
AR5	M	30 / 52	spasticity and pyramidal features	*GALC*	Intragenic	seq[GRCh37] 14q31.3q31.3(88400110_88416379)x1<br>NC_000014.8:g.(85996261_88400110)_(88416379_88416977)del<br>*exon 12-17 (NM_000153.4) In-frame*<br>*second variant: c.334A>G p.(Thr112Ala)*	1	nd	P<br>VOUS	y	Krabbe disease (#245200)	chr14_005094<br>GALC_00004
AR6	F	? / 8	paroxysmal dyskinesia	*KCTD7*	Intragenic	seq[GRCh37] 7q11.21q11.21(66103239_66108216)x1<br>NC_000007.13:g.(66098431_66103239)_(66108216_66236869)del<br>*exon 3-4 (NM_153033.4) Out-of-frame*<br>*no second variant*	1	nd	LP	uncertain	Epilepsy, progressive myoclonic 3, with or without intracellular inclusions (#611726)	chr7_006042
AR7	F	? / 53	pyramidal features, atrophy, muscle weakness, hyporeflexia legs	*PANK2*	~ 30	seq[GRCh37] 20p13p13(2815982_3955031)x1<br>NC_000020.10:g.(2797740_2815982)_(3955031_4053214)del<br>*no second variant*	1	nd	P	uncertain	Neurodegeneration with brain iron accumulation 1 (#234200)	chr20_002896
AR8	F	? / 10	spasticity, ataxia	*PDHX*	Intragenic	seq[GRCh37] 11p13p13(34978950_34982107)x1<br>NC_000011.9:g.(34969208_34978950)_(34982107_34988191)del<br>exon 4-5 (NM003477.3) Out-of-frame<br>*no second variant*	1	nd	P	uncertain	Lacticacidemia due to PDX1 deficiency (#245349)	chr11_007838
AR9	F	30 / 62	spasticity, dysarthria	*SPG11*	Intragenic	seq[GRCh37] 15q21.1q21.1(44877853_44896402)x3<br>NC_000015.9:g.(44876753_44877853)_(44896402_44898148)dupc<br>*exon 21-29 (NM_025137.4) Out-of-frame*<br>*second variant: c.7115_7123del p.(Leu2372_Lys2374del)*	3	nd	VOUS<br>VOUS	uncertain	Spastic paraplegia type 11 (#604360)	chr15_005983<br>SPG11_000106
AR10	M	15 / 24	spasticity and speech problems	*SPG11*	Intragenic	seq[GRCh37] 15q21.1q21.1(44862623_44867215)x1<br>NC_000015.9:g.(44861734_44862623)_(44867215_44876060)del<br>*exon 31-34 (NM_025137.4) Out-of-frame*<br>*second variant: c.5623C>T p.(Gln1875*)*	1	maternal<br>paternal	P<br>P	y	Spastic paraplegia type 11 (#604360)	chr15_005984<br>SPG11_000114
AR11	F	10 / 31	spastic paraplegia, swallowing and speech disorder	*SPG11*	Intragenic	seq[GRCh37] 15q21.1q21.1(44862666_44867274)x1<br>NC_000015.9:g.(44861719_44862666)_(44867274_44875972)delc<br>*exon 31-34 (NM_025137.4) Out-of-frame*<br>*second variant: c.4811dup p.(Leu1605fs)*	1	nd	P<br>P	y	Spastic paraplegia type 11 (#604360)	chr15_005985<br>SPG11_000119
AR12	M	? / 34	hereditary spastic paraplegia	*SPG11*	Intragenic	seq[GRCh37] 15q21.1q21.1(44887491_44889123)x1<br>NC_000015.9:g.(44884638_44887491)_(44889123_44890374)delc<br>*exon 24-26 (NM_025137.4) Out-of-frame*<br>*second variant: c.3075dup p.(Glu1026fs)*	1	maternal<br>paternal	P<br>P	y	Spastic paraplegia type 11 (#604360)	chr15_005986<br>SPG11_000019
AR13	F	? / 56	pyramidal features, spasticity, muscle atrophy, spinal contracture	*SPG11*	Intragenic	seq[GRCh37] 15q21.1q21.1(44862666_44867274)x1<br>NC_000015.9:g.(44861719_44862666)_(44867274_44875972)del<br>*exon 31-34 (NM_025137.4) Out-of-frame*<br>*no second variant*	1	nd	P	uncertain	Spastic paraplegia type 11 (#604360)	chr15_005985
AR14	M	? / 69	myelopathy and laryngospasm	*SPG11*	Intragenic	seq[GRCh37] 15q21.1q21.1(44862666_44867274)x1<br>NC_000015.9:g.(44861719_44862666)_(44867274_44875972)del<br>*exon 31-34 (NM_025137.4) Out-of-frame*<br>*no second variant*	1	nd	P	uncertain	Spastic paraplegia type 11 (#604360)	chr15_005985
AR15	F	10 / 56	cataract, pyramidal features, spasticity, hypotension	*SPG11*	Intragenic	seq[GRCh37] 15q21.1q21.1(44862666_44867274)x1<br>NC_000015.9:g.(44861719_44862666)_(44867274_44875972)del<br>*exon 31-34 (NM_025137.4) Out-of-frame*<br>*no second variant*	1	nd	P	uncertain	Spastic paraplegia type 11 (#604360)	chr15_005985
AR16	M	? / 2	ataxia, intellectual disability	*SPG7*	Intragenic	seq[GRCh37] 16q24.3q24.3(89595890_89620977)x1<br>NC_000016.9:g.(89592941_89595890)_(89620977_89623235)delc<br>*exon 6-16 (NM_003119.4) Out-of-frame*<br>*no second variant*	1	maternal	P	uncertain	Spastic paraplegia type 7 (#607259)	chr16_006615
AR17	M	? / 63	spastic paraparesis, ataxia, mild retardation, ophthalmoplegia	*SPG7*	Intragenic	seq[GRCh37] 16q24.3q24.3(89595890_89620977)x0<br>NC_000016.9:g.(89592941_89595890)_(89620977_89623235)del<br>*exon 6-16 (NM_003119.4) Out-of-frame*	0	nd	P	y	Spastic paraplegia type 7 (#607259)	chr16_006615
AR18	F	53 / 65	bilateral pyramidal syndrome	*SPG7*	Intragenic	seq[GRCh37] 16q24.3q24.3(89574039_89575481)x1<br>NC_000016.9:g.(89390951_89574039)_(89575481_89576906)del[c]<br>*exon 1 (NM_003119.4) In-frame*<br>*second variant: c.1045G>A p.(Glu349Ser)*	1	nd	P<br>LP	y	Spastic paraplegia type 7 (#607259)	chr16_006616
AR19	M	? / 25	ataxia	*SPG7*	Intragenic	seq[GRCh37] 16q24.3q24.3(89623235_89623441)x1<br>NC_000016.9:g.(89620977_89623235)_(89623441_89627434)del[c]<br>*exon 17 (NM_003119.4) In-frame*<br>*second variant: c.861dup p.(Asn288*)*	1	maternal<br>*paternal*	P<br>P	y	Spastic paraplegia type 7 (#607259)	chr16_006617<br>SPG7_000022
AR20	M	25 / 46	ataxia	*SYNE1*	Intragenic	seq[GRCh37] 6q25.2q25.2(152685922_152690810)x0<br>NC_000006.11:g.(152685045_152685922)_(152690810_152694177)del<br>*exon 61-64 (NM_182961.4) In-frame*	0	nd	P	y	Spinocerebellar ataxtia type 8 (#610743)	chr6_007448
AR21	M	12 / 17	developmental delay, dystonia, spasticity, drooling, ragged red fibres in muscle biopt	*TANGO2*	Intragenic	seq[GRCh37] 22q11.21q11.21(20030877_20050965)x0<br>NC_000022.10:g.(20024377_20030877)_(20050965_20073209)del<br>*exon 3-8 (NM_152906.7) In-frame*	0	maternal & paternal	P	y	Metabolic encephalomyopathic crises, recurrent, with rhabdomyolysis, cardiac arrhythmias, and neurodegeneration (#616878)	chr22_003032
AR22	F	? / 62	Parkinson, dystonia	*PRKN*	Intragenic	seq[GRCh37] 6q26q26(162394233_162622319)x1<br>NC_000006.11:g.(162206974_162394233)_(162622319_162683461)delc<br>*exon 4-6 (NM_004562.3) Out-of-frame*<br>*second variant: c.1289G>A p.(Gly430Asp)*	1	nd	P<br>LP	y	Juvenile Parkinson disease type 2 (#600116)	chr6_007449<br>PARK2_000061
AR23	F	45 / 52	Parkinson	*PRKN*	Intragenic	seq[GRCh37] 6q26q26(162394233_162475244)x1<br>NC_000006.11:g.(162206974_162394233)_(162475244_162622080)delc<br>*exon 5-6 (NM_004562.3) Out-of-frame*	1	nd	P	y	Juvenile Parkinson disease type 2 (#600116)	chr6_007450

						second variant: c.167T>A p.(Val56Glu)			VOUS			PARK2_000014
AR24	F	? / 39	Parkinson	*PRKN*	Intragenic	seq[GRCh37] 6q26q26(162394233_162394462)x4<br>NC_000006.9:g.(162206974_162394233)_(162394462_162475040)dupc<br>*exon 6 (NM_004562.3) Out-of-frame*	4	maternal & paternal	LP	y	Juvenile Parkinson disease type 2 (#600116)	chr6_007451
AR25	F	? / 41	Parkinson, cervical dystonia	*PRKN*	Intragenic	seq[GRCh37] 6q26q26(162622080_162683770)x1<br>NC_000006.11:g.(162475244_162622080)_(162683770_162864377)del[c]<br>*exon 3-4 (NM_004562.3) In-frame*	1	nd	P	y	Juvenile Parkinson disease type 2 (#600116)	chr6_007452
						second variant: c.719C>T p.(Thr240Met)			VOUS			chr6_007456
AR26	M	? / 42	spasticity, cataract, dystonia, parkinson	*PRKN*	Intragenic	seq[GRCh37] 6q26q26(162683461_162683770)x1<br>NC_000006.11:g.(162622319_162683461)_(162683770_162864377)delc<br>*exon 3 (NM_004562.3) Out-of-frame*	1	nd	P	y	Juvenile Parkinson disease type 2 (#600116)	chr6_007453
						second variant: c.101_102del p.(Gln34fs)			P			PARK2_000007
AR27	M	? / 63	extrapyramidal features	*PRKN*	Intragenic	seq[GRCh37] 6q26q26(162622080_162622319)x1<br>NC_000006.11:g.(162475244_162622080)_(162622319_162683461)delc<br>*exon 4 (NM_004562.3) Out-of-frame*	1	paternal	P	uncertain	Juvenile Parkinson disease type 2 (#600116)	chr6_007454
						no second variant		*nd*				

Genome assembly GRCh37 (hg19)

c= confirmed by MLPA/array/sequencing/deletion PCR, see suppl. File 2

*Based on ACMG guidelines. P=Pathogenic, LP=Likely Pathogenic, VOUS = Variant of Unknown Significance

[#] in some cases CNV's may extend into other sequences beyond the genes (i.e. intergenic or non-coding genes)

^Nomenclature according HGVS and/or ISCN (http://varnomen.hgvs.org/recommendations/DNA/variant/complex/). In case of intragenic CNVs, the predicted effect on the reading frame is indicated.
