# Supplementary material for: Copy number variants from 4800 exomes contribute to ~7% of genetic diagnoses in movement disorders, muscle disorders and neuropathies
Source: Eur J Hum Genet. 2023 Feb 13;31(6):654–62. doi: 10.1038/s41431-023-01312-0 (PMC10250492; doi:10.1038/s41431-023-01312-0)
Supplement: Supplementary file 5 — Sup. Table 3 [file 41431_2023_1312_MOESM5_ESM.pdf]

Individual number	Sex	age onset / age at WES request	Reason	Disease causing gene(s)	Intragenic /# of genes	Variant^	Copy number(s)	inheritance	Classification*	Likely diagnosis	OMIM disease	LOVD ID
**Inheritance pattern of the involved variant/gene: AD**												
BD1	M	? / 8	motor problems, reduced excercise tolerance, decreased muscle strength	contiguous gene duplication syndrome	> 50	seq[GRCh37] 22q11.21q11.21(18893961_21414845)x3<br>NC_000022.10:g.(18775422_18893961)_(21414845_21576183)dup	3	maternal	P	y	Chromosome 22q11.2 duplication syndroom (#608363)	chr22_003030
BD2	M	0 / 3	Delayed motor development, instability, weakness, drooling and protruding shoulder blade	contiguous gene deletion syndrome	> 50	seq[GRCh37] 22q11.21q11.21(18775072_21414845)x1<br>NC_000022.10:g.(18656690_18775072)_(21414845_21576183)del	1	de novo (paternity and maternity checked)	P	y	Chromosome 22q11.2 deletion syndroom (#611867)	chr22_003031
BD3	M	0 / 13	exercise tolerance, congenital / axial myopathy, PDDNOS	contiguous gene duplication syndrome	~ 20	seq[GRCh37] 16p13.11p13.11(14916710_16315604)x3<br>NC_000016.9:g.(14772727_14916710)_(16315604_16349511)dup	3	nd	P	y	Chromosome 16p11 duplication syndroom (PMID30287593)	chr16_006618
BD4	M	14 / 15	muscular dystrophy, limb-girdle and distal muscle weakness, finger and elbow contractures	*SMCHD1*	7	seq[GRCh37] 18p11.31p11.31(2771468_3277978)x1<br>NC_000018.9:g.(2770192_2771468)_(3277978_3447718)del<br>*DUX4 status unknown*	1	nd	LP	y	Digenic Fascioscapulohumeral muscular dystrophy 2 (#158901)	chr18_002480
BD5[%]	F	22 / 23	progressive exercise-related muscle pain and acidification, mild proximal hip weakness	*CAPN3* [%]	Intragenic	seq[GRCh37] 15q15.1q15.1(42676657_42684925)x1<br>NC_000015.9:g.(42652323_42676657)_(42684925_42686351)del<br>*exon 2-7 (NM_000070.2) In-frame*	1	nd	VOUS	uncertain	Limb-girdle muscular dystrophy type 1 and 4 (#253600 and 618129)	chr15_005987
BD6	F	2 / 35	congenital mypathy	*COL6A3*	Intragenic	seq[GRCh37] 2q37.3q37.3(238272947_238280992)x1<br>NC_000002.11:g.(238272110_238272947)_(238280992_238283014)del[c]<br>exon 9-13 (NM_004369.4) In-*frame*	1	de novo (paternity and maternity checked)	P	y	Bethlem myopathy 1 (#158810) and Ullrich congenital muscular dystrophy 1 (#254090)	chr2_020450
BD7[%]	F	? / 20	exercise-related muscle pain and hyperCKemia, rhabdomyolysis	*COL6A3* [%]	Intragenic	seq[GRCh37] 2q37.3q37.3(238267054_238268923)x3<br>NC_000002.11:g.(238266574_238267054)_(238268923_238269676)dup<br>exon 17-21 (NM_004369.4) *In-frame*	3	nd	VOUS	uncertain	Bethlem myopathy 1 (#158810) and Ullrich congenital muscular dystrophy 1 (#254090)	chr2_020451
BD8	M	? / 13	proximal paresis, exercise intolerance, areflexion	*PMP22*	7	seq[GRCh37] 17p12p12(14095305)_(15477522)x3<br>NC_000017.10:g.(14005599_14095305)_(15477522_15492232)dup	3	nd	P	y	Charcot-Marie-Tooth disease 1A (#1118220)	chr17_008701
**Inheritance: AR**												
BR1	M	? / 15	ataxia, epilepsy, tubulopathy, exercise intolerance, complex I / II deficiency	unknown, possibly *SLC18A3* , *CHAT or OGDHL*	~ 40	seq[GRCh37] 10q11.22q11.23(47409858_51633060)x1<br>NC_000010.10:g.(46967725_47409858)_(51633060_516448851)del<br>*no second variant in SLC18A3, CHAT, OGDHL*	1	paternal	P	uncertain	Congenital myasthenic syndrome 21 (#617239) Congenital myasthenic syndrome 6 (#254210), Yoon-Bellen neurodevelopmental syndrome (#619701)	chr10_005520
BR2	M	31 / 33	Muscle cramps and weakness. Mother has Pompe disease	*GAA*	Intragenic	seq[GRCh37] 17q25.3q25.3(78091938_78092166)x1<br>NC_000017.10:g.(78091555_78091938)_(78092166_78092370)del<br>*exon 18 (NM_000152.5) In-frame*<br>*no second variant*	1	nd	P	uncertain	Glycogen storage disease II (#232300)	chr17_008702
BR3	M	? / 38	muscle atrophy, contractures, arthrogryposis multiplex congenita, muscular dystrophy, ocular muscle weakness	*LARGE1*	Intragenic	seq[GRCh37] 22q12.3q12.3(33670431_33733814)x3<br>NC_000022.10:g.(33562856_33670431)_(33733814_33777909)dup<br>*exon 11-16 (NM_004737.6) In-frame*<br>*no second variant*	3	nd	VOUS	uncertain	Muscular dystrophy-dystroglycanopathy type 6 (#608840 and #613154)	chr22_003032
BR4	M	? / 10	facial weakness, limb weakness distal> proximal	*NEB*	Intragenic	seq[GRCh37] 2q23.3q23.3(152466318_152582063)x1<br>NC_000002.11:g.(152432808_152466318)_(152582063_152584191)del<br>*exon 6-81/105 (NM_004543.4) In-frame*<br>*no second variant*	1	maternal	P	uncertain	Nemaline myopathy 2 (#256030)	chr2_020452
BR5	F	10 / 11	developmental delay, muscle weakness limb-girdle pattern, muscle weakness distal, hyperCK emission	*SGCG*	Intragenic	seq[GRCh37] 13q12.12q12.12(23894775_23894899)x0<br>NC_000013.10:g.(23869626_23894775)_(23894899_23898506)del[c]<br>*exon 7 (NM_000231.3) Out-of-frame*	0	maternal & paternal	P	y	Limb-girdle muscular dystrophy type 5 (#253700)	chr13_002360
BR6	M	? / 42	congenital myopathy	*TTN*	Intragenic	seq[GRCh37] 2q31.2q31.2(179394967_179442912)x1<br>NC_000002.11:g.(179394843_179394967)_(179442912_179443337)del<br>*exon 273-307 (NM_133378.4) In-frame*	1	nd	P	uncertain	Limb-girdle muscular dystrophy (608807)	chr2_020453
						second variant: c.34625T>C p.(Val11542Ala)			VOUS			TTN_000252
BR7	F	? / 12	muscle weakness limb-girdle pattern, exercise intolerance, joint pain, fatigue, muscle biopsy abnormalities, bilateral sensorineural hearing loss	*TTN*	Intragenic	seq[GRCh37] 2q31.2q31.2(179556935_179601520)x3<br>NC_000002.11:g.(179556935)_(179601520)dup in tandem[c]<br>exon 51-122 (NM_1333784.4) *In-frame*	3	maternal	LP	y	Limb-girdle muscular dystrophy (608807)	chr2_020454
						second variant: c.12064C>T p.(Arg4022)*		*paternal*	P			TTN_005127
**Inheritance: XL**												
BX1	F	? / 57	muscle weakness facio-bulbar and limb-girdle pattern	*MTM1*	3	seq[GRCh37] Xq28q28(149760968_149998006)x1<br>NC_000023.10:g.(149681299_149760968)_(149998006_150154184)del	1	nd	P	y	X-linked myotubular myopathy (#310400)	chrX_018706
BX2	F	? / 25	exercise intolerance, myalgia, hyperCK emission, high ferritin, myopathic muscle biopsy	*DMD*	Intragenic	seq[GRCh37] Xp21.1p21.1(32429868_32717410)x1<br>NC_000023.10:g.(32408298_32429868)_(32717410_32827609)del<br>*exon 8-30 (NM_004006.2) Out-of-frame*	1	nd	P	y	Duchenne or Becker muscular dystrophy (#310200 and #300376)	chrX_018707

BX3	M	? / 14	muscle cramps, myalgia, hyperCK-emia	*DMD*	Intragenic	seq[GRCh37] Xp21.1p21.1(32456358_32662430)x0<br>NC_000023.10:g.(32430030_32456358)_(32662430_32663081)del[c]<br>*exon 11-29 (NM_004006.2) In-frame*	0	de novo (maternity checked)	P	y	Duchenne or Becker muscular dystrophy (#310200 and #300376)	chrX_018708
BX4	M	? / 50	muscle weakness limb-girdle pattern	*DMD*	Intragenic	seq[GRCh37] Xp21.1p21.1(31893307_31986631)x0<br>NC_000023.10:g.(31854939_31893307)_(31986631_32235032)del[c]<br>*exon 45-48 (NM_004006.2) In-frame*	0	nd	P	y	Duchenne or Becker muscular dystrophy (#310200 and #300376)	chrX_018709
BX5	F	? / 12	hip dysplasia, late milestones, ptosis, rapid choking, agenesis (> 10 elements), contractures of elbows and finger joints	*DMD*	Intragenic	seq[GRCh37] Xp21.1p21.1(30851433_31286044)x3<br>NC_000023.10:g.(30851433)_(31286044)dup[c]<br>*exon 63-79 (NM_004006.2) Out-of-frame*<br>and<br>seq[GRCh37] Xp21.1p21.1(31675215_32174025)x3<br>NC_000023.10:g.(31675215)_(32174025)dup[c]<br>*exon 45-54 (NM_004006.2) Out-of-frame*	3	nd	LP	y	Duchenne or Becker muscular dystrophy (#310200 and #300376)	chrX_018710 and chrX_018714
BX6	F	40 / 48	myopathy, muscular dystrophy, muscle weakness limb-girdle pattern	*DMD*	Intragenic	seq[GRCh37] Xp21.1p21.1(31496270_31950345)x1<br>NC_000023.10:g.(31462793_31496270)_(31950345_31986484)del[c]<br>*exon 46-59 (NM_004006.2) Out-of-frame*	1	nd	P	y	Duchenne or Becker muscular dystrophy (#310200 and #300376)	chrX_018711
BX7	M	? / 22	muscular dystrophy, limb-girdle muscle weakness, caridal symptoms, respiratory symptoms, suspicion Duchenne, intellectual disability	*DMD*	Intragenic	seq[GRCh37] Xp21.1p21.1(31838091_31986631)x0<br>NC_000023.10:g.(31792309_31838091)_(31986631_32235032)del[c]<br>*exon 45-50 (NM_004006.2) Out-of-frame*	0	nd	P	y	Duchenne or Becker muscular dystrophy (#310200 and #300376)	chrX_018712
BX8	M	? / 4	Duchenne muscular dystrophy, hyperCK-emia	*DMD*	Intragenic	seq[GRCh37] Xp21.1p21.1(31187487_31646026)x0<br>NC_000023.10:g.(31165638_31187487)_(31646026_31676121)del<br>*exon 55-74 (NM_004006.2) In-frame*	0	nd	P	y	Duchenne or Becker muscular dystrophy (#310200 and #300376)	chrX_018713

Genome assembly GRCh37 (hg19)

c= confirmed by MLPA/array/sequencing/deletion PCR, see suppl. File 2

*Based on ACMG guidelines. P=Pathogenic, LP=Likely Pathogenic, VOUS = Variant of Unknown Significance

[#] in some cases CNV's may extend into other sequences beyond the genes (i.e. intergenic or non-coding genes)

[%]these individuals may also have a recessive disorders since the genes are involved in both dominant and recessive disorders. No family history; no segregation has been performed.

^Nomenclature according HGVS and/or ISCN (http://varnomen.hgvs.org/recommendations/DNA/variant/complex/). In case of intragenic CNVs, the predicted effect on the reading frame is indicated.
