# Supplementary material for: Copy number variants from 4800 exomes contribute to ~7% of genetic diagnoses in movement disorders, muscle disorders and neuropathies
Source: Eur J Hum Genet. 2023 Feb 13;31(6):654–62. doi: 10.1038/s41431-023-01312-0 (PMC10250492; doi:10.1038/s41431-023-01312-0)
Supplement: Supplementary file 6 — Sup. Table 4 [file 41431_2023_1312_MOESM6_ESM.pdf]

Individual number	Sex	age onset / age at WES request	Reason	Disease causing gene(s)	Intragenic /# of genes	Variant^	Copy number(s)	inheritance	Classification*	Likely diagnosis	OMIM disease	LOVD ID
**Inheritance pattern of the involved variant/gene: AD**												
CD1	F	? / 44	neuropathy	*DCTN1*	> 20	seq[GRCh37] 2p13.2p13.2(74300675_74909185)x3<br>NC_000002.11:g.(74273449_74300675)_(74909185_75059781)dup	3	nd	VOUS	uncertain	Distal hereditary motor neuronopathy type VIIB (#607641)	chr2_020455
CD2	M	? / 72	neuropathy	*PMP22*	*7*	seq[GRCh37] 17p12p12(14095305)_(15477522)x1<br>NC_000017.10:g.(14005599_14095305)_(15477522_15492232)del	1	nd	P	y	Neuropathy, recurrent, with pressure palsies #162500)	chr1&_008703
CD3	F	? / 44	HMSN / HNPP	*PMP22*	*7*	seq[GRCh37] 17p12p12(14095305)_(15477522)x1<br>NC_000017.10:g.(14005599_14095305)_(15477522_15492232)del	1	nd	P	y	Neuropathy, recurrent, with pressure palsies #162500)	chr1&_008703
CD4	F	<10 / 64	diffuse paresis distal> proximal, pes cavus	*PMP22*	*7*	seq[GRCh37] 17p12p12(14095305)_(15477522)x3<br>NC_000017.10:g.(14005599_14095305)_(15477522_15492232)dup	3	nd	P	y	Charcot-Marie-Tooth disease 1A (#1118220)	chr17_008701
CD5	F	? / 60	neuropathy	*PMP22*	*7*	seq[GRCh37] 17p12p12(14095305)_(15477522)x3<br>NC_000017.10:g.(14005599_14095305)_(15477522_15492232)dup	3	nd	P	y	Charcot-Marie-Tooth disease 1A (#1118220)	chr17_008701
CD6	F	? / 61	Charcot-Marie-Tooth	*PMP22*	*7*	seq[GRCh37] 17p12p12(14095305)_(15477522)x3<br>NC_000017.10:g.(14005599_14095305)_(15477522_15492232)dup	3	nd	P	y	Charcot-Marie-Tooth disease 1A (#1118220)	chr17_008701
CD7	F	7 / 27	HMSN type I	*PMP22*	*7*	seq[GRCh37] 17p12p12(14095305)_(15477522)x3<br>NC_000017.10:g.(14005599_14095305)_(15477522_15492232)dup	3	nd	P	y	Charcot-Marie-Tooth disease 1A (#1118220)	chr17_008701
CD8	M	? / 68	HMSN type I	*PMP22*	*7*	seq[GRCh37] 17p12p12(14095305)_(15477522)x3<br>NC_000017.10:g.(14005599_14095305)_(15477522_15492232)dup[c]	3	nd	P	y	Charcot-Marie-Tooth disease 1A (#1118220)	chr17_008701
CD9	M	30 / 49	neuropathy, muscle atrophy, distal muscle weakness, hollow feet	*PMP22*	*7*	seq[GRCh37] 17p12p12(14095305)_(15477522)x3<br>NC_000017.10:g.(14005599_14095305)_(15477522_15492232)dup	3	nd	P	y	Charcot-Marie-Tooth disease 1A (#1118220)	chr17_008701
CD10	F	26 / 60	HMSN type II	*PMP22*	*7*	seq[GRCh37] 17p12p12(14095305)_(15477522)x3<br>NC_000017.10:g.(14005599_14095305)_(15477522_15492232)dup	3	nd	P	y	Charcot-Marie-Tooth disease 1A (#1118220)	chr17_008701
CD11	F	? / 50	polyneuropathy	*PMP22*	*7*	seq[GRCh37] 17p12p12(14095305)_(15477522)x3<br>NC_000017.10:g.(14005599_14095305)_(15477522_15492232)dup	3	nd	P	y	Charcot-Marie-Tooth disease 1A (#1118220)	chr17_008701
CD12	M	? / 5	HMSN type 1A, hypotonia, ptosis, ceroid lipofuscinosis	*PMP22*	*7*	seq[GRCh37] 17p12p12(14095305)_(15477522)x3<br>NC_000017.10:g.(14005599_14095305)_(15477522_15492232)dup	3	nd	P	y	Charcot-Marie-Tooth disease 1A (#1118220)	chr17_008701
CD13	M	? / 44	HMSN type I	*PMP22*	*7*	seq[GRCh37] 17p12p12(14095305)_(15477522)x3<br>NC_000017.10:g.(14005599_14095305)_(15477522_15492232)dup	3	nd	P	y	Charcot-Marie-Tooth disease 1A (#1118220)	chr17_008701
CD14	M	? / 64	neuropathy	*PMP22*	*7*	seq[GRCh37] 17p12p12(14095305)_(15477522)x3<br>NC_000017.10:g.(14005599_14095305)_(15477522_15492232)dup	3	nd	P	y	Charcot-Marie-Tooth disease 1A (#1118220)	chr17_008701
**Inheritance: AR**												
CR1	M	? / 7	neuropathy, motor developmental delay	*FARS2*	*2*	seq[GRCh37] 6p25.1p25.1(5216764_5545601)x1<br>NC_000006.11:g.(5144510_5216764)_(5545601_5613394)del<br>exon 1-5 (NM_006567.5)<br>*no second variant*	1	nd	LP	uncertain	autosomal recessive spastic paraplegia 77 (#617046)	chr6_007456

Genome assembly GRCh37 (hg19)

c= confirmed by MLPA/array/sequencing/deletion PCR, see suppl. File 2

*Based on ACMG guidelines. P=Pathogenic, LP=Likely Pathogenic, VOUS = Variant of Unknown Significance

[#] in some cases CNV's may extend into other sequences beyond the genes (i.e. intergenic or non-coding genes)

^Nomenclature according HGVS and/or ISCN (http://varnomen.hgvs.org/recommendations/DNA/variant/complex/). In case of intragenic CNVs, the predicted effect on the reading frame is indicated.
