# Supplementary material for: Copy number variants from 4800 exomes contribute to ~7% of genetic diagnoses in movement disorders, muscle disorders and neuropathies
Source: Eur J Hum Genet. 2023 Feb 13;31(6):654–62. doi: 10.1038/s41431-023-01312-0 (PMC10250492; doi:10.1038/s41431-023-01312-0)
Supplement: Supplementary file 7 — Description of Supplementary information [file 41431_2023_1312_MOESM7_ESM.docx]

**Supplementary Figure 1. Cohort breakdown per disorder.**

For each of the 3 sub cohorts (indicated) the number of probands with no variants detected (dark blue, lowest bars), matching single nucleotide variant (s) (*SNV(s);* blue/green, middle bars) or matching CNV(s) (light blue, top bars) are given. The number of probands per bar are indicated in the figure.

**Supplementary Figure 2. IGV plots and confirmations**

**Supplementary Table 1. Genes related to Parkinson disease panel**

**Supplementary Table 2. Overview of individual info for movement disorder panel**

**Supplementary Table 3. Overview of individual info for muscle disease panel**

**Supplementary Table 4. Overview of individual info for neuropathies panel**
